# Full pathogen characterisation: species identification including the detection of virulence factors and antibiotic resistance genes via multiplex DNA-assays

**DOI:** 10.1038/s41598-021-85438-5

**Published:** 2021-03-16

**Authors:** Noa Wolff, Michaela Hendling, Fabian Schroeder, Silvia Schönthaler, Andreas F. Geiss, Branka Bedenic, Ivan Barišić

**Affiliations:** 1 Molecular Diagnostics, AIT Austrian Institute of Technology, Giefinggasse 4, 1210 Vienna, Austria; 2 Ekorefugium, Slunj, Croatia; 3 University of Natural Resources and Life Sciences, Vienna, Austria; 4 Department of Microbiology, School of Medicine, University of Zagreb, Zagreb, Croatia

**Keywords:** Microbiology, Molecular biology, Diseases, Medical research, Molecular medicine

## Abstract

Antibiotic resistances progressively cause treatment failures, and their spreading dynamics reached an alarming level. Some strains have already been classified as highly critical, e.g. the ones summarised by the acronym ESKAPE (*Enterococcus faecium*, *Staphylococcus aureus*, *Klebsiella pneumoniae*, *Acinetobacter baumannii*, *Pseudomonas aeruginosa* and *Enterobacter* spp.). To restrain this trend and enable effective medication, as much information as possible must be obtained in the least possible time. Here, we present a DNA microarray-based assay that screens for the most important sepsis-relevant 44 pathogenic species, 360 virulence factors (mediate pathogenicity in otherwise non-pathogenic strains), and 409 antibiotic resistance genes in parallel. The assay was evaluated with 14 multidrug resistant strains, including all ESKAPE pathogens, mainly obtained from clinical isolates. We used a cost-efficient ligation-based detection platform designed to emulate the highly specific multiplex detection of padlock probes. Results could be obtained within one day, requiring approximately 4 h for amplification, application to the microarray, and detection.

## Introduction

Challenges associated to antibiotic treatment, indispensable in medicine, have been forecasted repeatedly in terms of antimicrobial resistances during the last two decades^1–3^. Potentially resistant pathogens limit the number of suitable measures and moreover retard treatment decisions, which cannot be longer made empirically^4^. Known examples for escalating resistance spreading are the extended spectrum β-lactamases and carbapenemases^5^, or the six pathogens summarised by the acronym ESKAPE (*Enterococcus faecium*, *Staphylococcus aureus*, *Klebsiella pneumoniae*, *Acinetobacter baumannii*, *Pseudomonas aeruginosa* and *Enterobacter* spp.), highlighted by the Infectious Diseases Society of America for being particularly critical in terms of antibiotic resistances (ABR)^6,7^. A precise antibiotic treatment is thus essential to contain the further spread of ABR mechanisms. The identification of the causative pathogen and their acquired ABR genes is of utmost importance^8^.

Further, addressing virulence factor (VF) genes is of increasing interest. Bacteria that have not turned to obligatory pathogens and can exist free-living or as commensals might turn into pathogens after acquiring certain VFs, which are frequently transferred on mobile genetic elements^9^. Those genes might, for instance, encode bacterial toxins or extracellular enzymes, which are directly involved in pathogenesis or cell surface components, such as membrane proteins and polysaccharides^10^. Detecting VFs allows e.g. the differentiation of harmless commensal *E. coli* strains from highly pathogenic ones^11^, for instance *E. coli* O157:H7, which demonstrates severe pathogenicity due to Shiga-like toxin genes received by bacteriophages^12^.

The gold standard in diagnostic microbiology is based on cultivation-dependent methods that are cost-effective, well established in the routine practice and diagnostically conclusive regarding the antibiotic susceptibility. Major drawbacks are that the cultivation of some pathogens is challenging and can last several weeks^13^. In addition, a previous treatment with antibiotics can cause false-negative results. To overcome these limitations, a wide variety of molecular diagnostic tests has been developed and is used for the pathogen identification.

Nevertheless, further technological advances are required to meet the clinical requirements^14,15^. A lot of mechanisms enable pathogens to protect themselves from antibiotic substances, including acquired ABR as well as intrinsic resistance mechanisms^16–18^. To investigate this multitude of mechanisms without relying on phenotypic observations, there is a large repertoire of molecular diagnostics technologies nowadays that already made their way to clinical everyday routine, as e.g. polymerase chain reaction (PCR)- and real time (RT)-PCR-based detection, matrix-assisted laser desorption/ionisation-time of flight mass spectrometry (MALDI-TOF MS), whole genome sequencing (WGS), and the microarray technique addressed here. The currently most popular and clinically used tests are based on real-time polymerase chain reactions (PCRs) due to their relatively good sensitivity, specificity and speed^19,20^. Using highly conserved ribosomal RNA (rRNA) genes as targets allows usually a higher sensitivity since multiple copies of this genes are present in the genome^21,22^. The application as well as the variability of PCR are multitudinous, offering RT-PCR, isothermal PCR, loop-mediated isothermal amplification, or the recombinase polymerase amplification (RPA). The most important in clinical diagnostics are the conventional and the real-time PCR. The RT-PCR, which allows to observe the amplification live using unspecifically intercalating fluorescence dyes or specific DNA sequences which give rise to a fluorescence signal only after hybridizing to the amplicon^23,24^. However, these tests can only identify a low number of targets because of the limited availability of differentiable fluorescence dyes. In addition, with an increasing degree of multiplexing, the sensitivity and specificity of PCRs is reduced due to unintended amplification products and primer dimer formation^25–27^.

A further tool in clinical microbiology is MALDI-TOF MS. This is usually employed for pathogen identification^28^. The detection principle is based on the evaporation and ionisation behaviour of bacterial cultures upon laser treatment. The resulting ions and the time they require to reach the detector are analysed by mass spectroscopy^29^. This time of flight (TOF) is characteristic for the respective pathogen and can hence be used to identify the organism by means of its deduced mass/charge ratio (m/z value). The resulting spectra are complex, but the spectral fingerprints vary enough to differentiate genera, as long as they exhibit the same growth conditions^30^. Since this method is cultivation-based and various pathogens exhibit different growth rates, the time until the bacterial culture is available for a MALDI-TOF MS analysis can vary significantly depending on the species. The method is also vulnerable to errors, especially regarding species differentiation, for example between *Streptococcus pneumoniae* and *Streptococcus mitis*, which might entail severe consequences^31–33^.

Irrespective of that, the usage of MALDI-TOF MS contributes well to the patients’ outcome rate, which is partially owed to the little time required for the identification^34^, partially to the further characterisation possibilities, especially regarding the ABR^35,36^. For instance, the presence of β-lactamase could be evidenced with the help of MALDI-TOF MS^37,38^, as well as other antibiotics^34^. However, MALDI-TOF MS identification is expression-dependent; not yet expressed proteins, induced by the antibiotic if present, cannot be detected at all^15,30^. In case of VFs, not all of them constitute expressed proteins. To integrate this technology in the clinical everyday routine, further studies concerning the detection of ABR and VFs owned by the pathogen are required. So far, ABR detection by MALDI-TOF MS is only addressed in research.

Regarding the content of information, WGS is superior to all other described methods, theoretically revealing the entireness of present phylogenetic marker genes, ABR genes, and VF genes. Since the invention of sequencing, there have been many and rapid developments in this field. The WGS costs, once the main limiting criterion, decreased dramatically within the last decade. After the sequence is obtained, no further detection setup is required; all the information is already contained by the sequence itself. Nevertheless, it still does not only require a lot of processing—and therefore time—to obtain the contiguous sequence and thereof the desired information out of the raw sequence snips, but also a strong bioinformatic expertise that exceeds the demands addressed to the commonly employed executive personnel by far. Trained experts, in turn, increase the costs again, after having been saved in terms of the sequencing reaction. In critical cases, the most limiting criterion might indeed be the time needed to analyse a genome. And for the clinical use, it is still too expensive.

An alternative method to identify and characterise pathogens that enable the detection of thousands of targets in parallel combines PCR with solid support-based detection systems, such as DNA microarrays and microbeads. While next generation sequencing platforms are currently replacing these hybridisation-based detection platforms in research facilities, they still remain attractive in clinical environments because of their relatively low price, short experimental run-times and manageable bioinformatic procedures^39,40^. In a sense, the microarray fills the gap between WGS and PCR^41^. Once developed for a comprehensive gene set (or with respect to the customer’s needs), a microarray chip can be made available ready-to-use with a standard procedure that easily fits in the clinical everyday routine, reducing time, effort, and required skills during application compared to WGS. On the other hand, a high number of genes can be detected simultaneously, since the spatial resolution of common fluorescence scanners allows high densities of loci carrying different probes. PCR-based detection, where either bands on a gel must be distinguishable or, in RT-PCR, different fluorescence dyes must be differentiated spectrally, which is problematic since dye spectra overlap, is more limited in terms of the maximum target gene number. A number of diagnostic microarrays using such platforms was already designed^42–46^.

The classical DNA microarray method is based on a hybridisation reaction between labelled target DNA and immobilised microarray probes^42,47^. A significant limitation of the microarray technology is the non-specific cross-hybridisation of amplification products to non-target microarray probes that massively impair the specificity^45,48,49^. Interestingly, the thermodynamic stability of the DNA double helix is less dependent on the base-pairing but rather on the base-stacking effects, making it difficult to avoid cross-hybridisations during a sequence-driven probe design process^50^. Also, the covalent linker of the microarray probe to the surface has an effect on the hybridisation interactions^51^. The yet poorly understood anomalous DNA behaviour can result even in thermodynamically more favourable intermediates comprising non-perfectly matching sequences in comparison to perfect-matching duplexes^52^.

**Figure 1:**
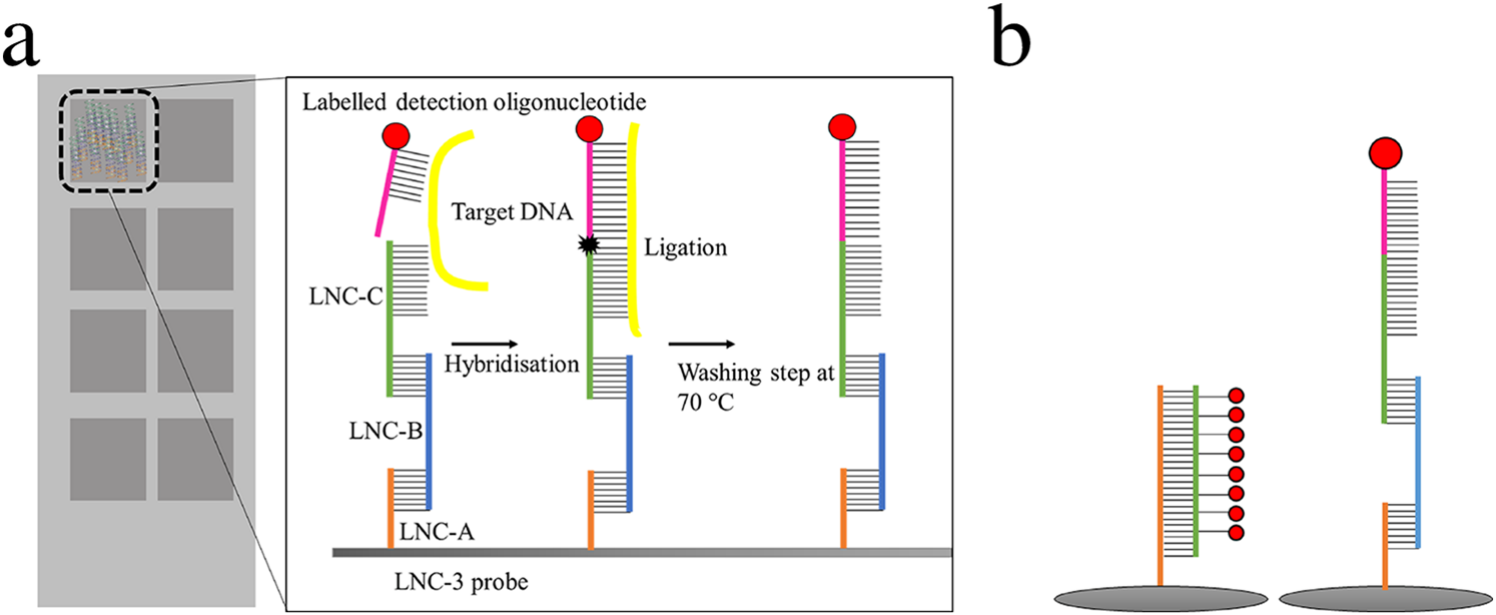
(**a**) Chip surface: Schematic representation of the 8 array chambers with the linear nucleotide chain (LNC-3) concept: To mimic liquid phase DNA hybridisation, three oligonucleotides, specified as LNC-A (orange), LNC-B (blue), and LNC-C (green), are immobilised on a functionalised glass surface (grey), the first one covalently, the others by hybridisation. The LNC-C terminal region is complementary to one part of the target DNA (yellow). The other part is complementary to the detection oligonucleotides (magenta), which are biotinylated and can be stained by fluorescence-labelled streptavidin (red). Once connected via the target DNA, LNC-C and detection oligonucleotide are ligated, thereby covalently linking the detection oligonucleotide to the immobilised probe. By washing at 70 °C, non-ligated detection oligonucleotides are removed. (**b**) **Comparison:** Identification via conventional, hybridisation-based detection (left) and LNC-3 technology (right).

As a consequence of this phenomenon, microarray detection protocols involving enzymes with proof-reading capability were developed to minimise non-specific cross-hybridisation effects. In addition, probe immobilisation concepts have been reported that focus on the emulation of the liquid phase DNA hybridisation behaviour to minimise surface-related effects^53^. Combining those advantages, we earlier introduced the linear nucleotide chain (LNC) concept (Fig. 1), which uses immobilised trimeric oligonucleotides in combination with a ligase reaction to provide a highly specific detection of target genes due to the proof-reading capability of the involved ligase and the emulation of the liquid phase hybridisation behaviour of detection oligonucleotides^54,55^.

In summary, plenty diagnostic techniques are available to characterise pathogens and ABR genes. However, an optimal diagnostic solution meeting the important criteria relevant for its application in clinical routine (low cost, high sensitivity, high specificity, low time-to-result, automatization, ABR and virulence characterisation) is still not available^56^. PCR and RT-PCR are overburdened by the mere number of target genes, MALDI-TOF is mainly used for pathogen identification, conventional microarrays suffer from poor specificity due to cross-hybridisations, and WGS is too expensive and time-consuming for a routine diagnostic method. In this study, we developed an assay that allows the full characterisation of pathogens causing sepsis. The microarray-based method identifies the 44 most sepsis-relevant bacterial pathogens, 360 VF genes, and 409 ABR genes at the same time to provide all information needed for a reasonable and effective treatment. The new assay was evaluated with 14 clinical strains including the initially mentioned ESKAPE pathogens.

## Materials and methods

Briefly summarised, all strains were cultivated and lysed, followed by DNA purification and PCR-based 45-plex amplification. The amplification products were applied to microarray chips that had been functionalised with DNA complementary to the genes of interest and complementary to their related mRNA (therefore constituting the base sequence of the non-coding strand). Fluorescence-labelled detection oligonucleotides, complementary to the adjacent region of the target DNA, were connected to the probes by the target DNA—if present—via hybridisation. If perfectly matching, the detection oligonucleotides were ligated to the immobilised DNA oligonucleotides. Non-ligated oligonucleotides, merely bound by hybridisation, were washed out. The chip readout occurred by means of a standard fluorescence-based microarray scanner. A conventional microarray platform comprising the identical detection sequences was used to showcase the specificity improvements realised by our detection concept. For the amplification of the DNA, application to the chip surface with subsequent ligation and washing steps, and the detection, approx. 4 h were required. Using the conventional microarray chip, ca. 4 h were needed as well, since an additional PCR step for the labelling was necessary.

### Bacterial cells and DNA purification

All reported strains were incubated at their optimal growth conditions overnight. A detailed description of media, growth conditions, and origin is given in Table S1. A total of 5 mL of each bacterial culture were washed in phosphate-buffered saline (PBS) buffer (1.05 mM KH_2_PO_4_, 155.17 mM NaCl, 2.97 mM Na_2_HPO_4_ 7 H_2_O, pH = 7.4) twice by spinning down (6000 g, 2 min) and resuspension, then finally resuspended in 1 mL of PBS buffer. Lysis was performed mechanically by a MagNa Lyser Instrument (Roche, Basel) at 6500 rpm for 0.5 min. A second treatment was performed after 5 min of incubation at room temperature (RT), followed by 10 min of incubation at 95 °C. After centrifuging at 16,000 *g* for 10 min, the supernatants were collected and used further.

To validate the microarray detection results, all strains were sequenced with the PGM Ion Torrent sequencer (Ion Personal Genome Machine System, Thermo Fisher Scientific, Waltham, MA, USA) as advised by the supplier. Raw reads were assembled de novo using Assembler SPAdes software^57^. The genome was annotated using the RAST (Rapid Annotations using Subsystems Technology) database^58,59^. The WGS data were screened for the presence of genes targeted by the assay using several published in silico tools (ResFinder^60^, Primeval^61^, resiDB^62^). The publicly available genome sequences were also analysed using these in silico tools.

### ***DNA*** amplification

After lysis and centrifugation, the supernatants were used directly as template DNA in a multi-primer PCR containing the primer pairs corresponding to all the genes screened for in case of ABR and VF genes, while for the 16S rRNA genes, a universal primer pair was designed covering approximately the whole gene. The 16S rRNA gene was used as our phylogenetic marker of choice because the genetic variability is low enough to use a single universal primer pair for the amplification of the DNA but high enough to differentiate clinically relevant species from each other. All primers are listed in the Supplementary Information, Tables 3–20. The PCR was conducted using the HotStartTaq DNA polymerase kit (Qiagen, Hilden, Germany) at final concentrations of 10 ng of template DNA, 5 µM of primer pairs (forward and reverse), and 3 mM of MgCl_2_ following the manufacturer’s instructions. The thermal cycling settings were: initial denaturation: 95 °C, 15 min; number of cycles: 40, comprising denaturation: 95 °C, 30 s; annealing: 55 °C, 30 s; elongation: 72 °C, 30 s; final elongation: 72 °C, 10 min; storage: 4 °C (Applied Biosystems GeneAmp PCR System 2700, Thermo Fischer Scientific, Waltham, MA, USA). Purification of the amplification products was done with the Stratec Invisorb Fragment Clean Up kit (Stratec Molecular GmbH, Berlin, Germany) following the manufacturer’s instructions, followed by 3 min of sonication (VWR Ultrasonic Cleaner USC-TH, PE, USA). The resulting amplified oligonucleotides served as target DNA for the detection reaction using the LNC-3 technology.

### DNA labelling for the conventional microarray

For the conventional microarray detection, 6 µL of the amplified DNA originating from the aforementioned PCR were labelled with Atto 532-labelled dCTP. The labelling reaction was conducted using 2 units of the Vent (exo-) DNA Polymerase kit (New England BioLabs, Ipswich, MA, USA), as it has no 3′ → 5′-proofreading exonuclease activity, and thus, facilitates the incorporation of the labelled dCTPs into the target DNA. To obtain the strand that was complementary to the immobilised probe only, an asymmetric PCR with the forward primer was implemented. The master mix of this PCR included 0.025 µM labelled dCTPs, 0.175 dNTPs mix, 0,9 µM forward primer and 1 mM MgSO_4_. The thermal cycling settings were: initial denaturation: 95 °C, 3 min; number of cycles: 25, comprising denaturation: 95 °C, 20 s; annealing: 55 °C, 20 s; elongation: 72 °C, 20 s; final elongation: 72 °C, 3 min; storage: 4 °C (Applied Biosystems GeneAmp PCR System 2700, Thermo Fischer Scientific, Waltham, MA, USA).

### Oligonucleotide design

First, a sequence database comprising all relevant genes was created using the tool ResiDB^62^. The tool allows the automatic creation and filtering of consensus sequences calculated based on user-defined parameters and publicly available gene entries. The consensus sequences were used to design the oligonucleotides with the Oli2go software, which uses thermodynamic calculations to facilitate multiplex detection applications^63^. The structure of the LNC oligonucleotides is summarised in Table 1 and listed completely in the Supplementary Information, Tables 3–20. The resulting oligonucleotides were obtained from Integrated DNA Technologies (Coralville, Iowa, USA), including Cy5-labelled LNC-A and Cy3-labelled LNC-B oligonucleotides serving as spotting and hybridisation controls.

**Table 1:** Microarray probe sequences and modifications.

Probe name	5′-mod	Sequence 5′-3′	Length
LNC-A	Thiol	TTTCGCTGCCGACCCTGCGCCGTGGCC	27 bp
LNC-B		CCCCGGCACGCGAGCCCACGCTGCTTTTTTGGCCACGGCGCAGGGTCGGCAGCG	54 bp
LNC-C		GCAGCGTGGGCTCGCGTGCCGGGGTTTTTTNNNNNNNNNNN	≈ 44 bp
Detection oligonucleotide		NNNNNNNNNNNNNTTT	≈ 23 bp
Hybridisation control		CCCCGGCACGCGAGCCCACGCTGCTTTTTTGGCCACGGCGCAGGGTCGGCAGCG/Cy3	54 bp
Spotting control	Thiol	TTTCGCTGCCGACCCTGCGCCGTGGCC/Cy5	27 bp

### Glass slide functionalisation

The glass slides intended for the LNC-3 probes were first cleaned by sonication in ddH_2_O, 100% ethanol, acetone, and 1 M NaOH for 10 min each, then finally immersed in 1 M HCl overnight. The next day, the slides were cleaned with ddH_2_O for 10 min twice, rinsed with 100% ethanol, and centrifuged to dry. The cleaned glass slides were immersed in 5% 3-aminopropyl-trimethoxysilane (ATS, Sigma-Aldrich, MO, USA) in dry acetone for 1 h to generate free amino groups on the top of the surface. Residual ATS was removed by washing with acetone three times for 5 min each, rinsing with 100% ethanol, and centrifuging to dry. Prior to succinimide functionalisation, the slides were baked at 90 °C for 50 min. Subsequently, 300 µL of 2 mM sulfo-m-maleimidobenzoyl-N-hydroxysulfosuccinimide (s-MBS, Thermo Fischer Scientific, Waltham, MA, USA) in PBS buffer was applied to the amino-functionalised surface. The reaction occurred in a humid surrounding overnight. The procedure was taken over from^64^.

### Spotting of the microarray probes

The LNC-3 probes were immobilised on the chip surface using an OmniGrid Contact Microarrayer (GenMachines, San Carlos, CA, USA) equipped with Stealth Micro Spotting Pins (ArrayIt, Microarray Technology, Sunnyvale, CA, USA). The spotting solution contained 5 µM of LNC-A, LNC-B, and LNC-C oligonucleotides each in sterile-filtered 2 × NaP_i_ spotting buffer (1 × NaP_i_: 0.1 M Na_2_HPO_4_, 0.15 M NaCl, pH = 6.5). Cy-5-labelled LNC-A oligonucleotides were used as spotting controls, Cy3-labelled LNC-B oligonucleotides as hybridisation controls. After spotting, the microarray chips remained in the spotting chamber overnight. The humidity was set to 60% during the entire process. The slides were immersed into 1 × NaP_i_ buffer repeatedly for 5 min to wash, then incubated in the same buffer additionally containing 10 mM β-mercaptoethanol for 1 h to inactivate residual reactive groups on the glass surface. To remove the β-mercaptoethanol, the NaP_i_ buffer washing step was repeated. To precipitate unbound LNC oligonucleotides, the slides were incubated in saline buffer (1.5 M NaCl, 0.01 M Na_2_HPO_4_, pH = 7) for 10 min. Another two washing steps were implemented with 5 × saline sodium citrate (SSC) buffer (1 × SSC buffer: 150 mM NaCl, 15 mM sodium citrate, pH = 7.0, obtained from Biorad, Hercules, California, USA) for five minutes each, the first with 0.1% Tween-20 (Sigma-Aldrich, MO, USA) and the second without. Finally, the slides were repeatedly rinsed with ddH_2_O, centrifuged to dry, and stored at − 20 °C.

### Spotting and blocking of the aldehyde-functionalised slides

The conventional microarray detection was carried out using aldehyde-modified slides (PolyAn, Berlin, Germany). The spotting was conducted analogously to the LNC-3 procedure with the same instruments but a spotting buffer comprising 3 M betaine and 6 × SSC buffer. The slides were immersed into a blocking solution containing 3 M urea and 0.1% SDS for 30 min. Afterwards, the slides were washed with PBS buffer containing 0.1% Tween-20 for 5 min. Finally, the slides were repeatedly rinsed with ddH_2_O, centrifuged to dry, and stored at − 20 °C.

### Detection oligonucleotide preparation

The biotin-modified detection oligonucleotides were ordered from Integrated DNA Technologies (Coralville, Iowa, USA) and then phosphorylated using the T4 polynucleotide kinase (Thermo Fischer Scientific, Waltham, MA, USA). For that, 50 U/reaction of the enzyme, 45 detection oligonucleotides at a concentration of 1.78 µM each, and 400 µM adenosine triphosphate (ATP) were allowed to react in a thermoshaker (Peqlab TS-100, VWR, Erlangen, Germany) at 37 °C overnight. The reaction was ended by 10 min of incubation at 75 °C. After establishing a protocol to save costs by self-biotinylating the detection oligonucleotides, a terminal deoxynucleotidyl transferase was used to elongate the detection oligonucleotides with biotinylated deoxynucleotide trisphosphates (dCTPs) without impairing their performance, which were subsequently bound by fluorescence-labelled, i.e. Alexa-647-conjugated, streptavidin. The procedure is outlined in detail in a recent study^55^.

### Solid-support-based ligation and detection

The ligation reaction, providing the required specificity, took place in gasket hybridisation chambers of a volume of 100 µL each (Agilent, Santa Clara, CA, USA). The ligation solution, comprising 5 U/reaction ampligase (Epicentre, Madison, WI, USA), 1.68 µM of each detection oligonucleotide, 100 nM of synthetic target DNA or 20 µL of PCR product, and 2 µg bovine serum albumin (BSA, New England BioLabs, Ipswich, MA, USA) in ampligase buffer (20 mM Tris-HCl, 25 mM KCl, 10 mM MgCl_2_, 0.5 mM nicotinamide adenine dinucleotide (NAD), 0.01% Triton X-100, pH = 8.3), was applied to the chamber. If PCR products were used, the mixture was heated up to 95 °C for 5 min followed by cooling down on ice prior to its application onto the microarray.

The ligation reaction was performed in a hybridisation oven (Microarray Hybridisation Chambers, Agilent, Santa Clara, CA, USA) at 55 °C for 1 h. The slides were subsequently washed repeatedly, first with 2 × SSC buffer containing 0.1% sodium dodecyl sulphate (SDS) for 5 min, second with 0.2 × SSC buffer for 2 min, third with ddH_2_O for 1 min, and finally with 70 °C ddH_2_O for 10 min to remove non-ligated hybridisation products. The slides were dried by centrifugation. Afterwards, the streptavidin-Alexa-647 conjugate (Jackson ImmunoResearch Laboratories Inc., West Grove, PA, USA) was diluted 1:1000 in sterile-filtered PBS buffer containing 0.1% Tween-20, applied to the slides, and incubated for 1 h at room temperature inside the hybridisation oven. Finally, the slides were washed with PBS buffer comprising 0.1% Tween-20 for 5 min, then twice with ddH_2_O. Again, the slides were centrifuged to dry.

### Conventional microarray detection

The conventional microarray hybridisation was performed in gasket hybridisation chambers of a volume of 100 μl each (Agilent, Santa Clara, CA, USA). The hybridisation mixture, comprising 20 μL of the fluorescence labelled amplification product, 40 μL hybridisation buffer (Roche Diagnostics GmbH, Rotkreuz, Switzerland), and 40 µL ddH_2_O, was applied to the hybridisation chambers. The reaction occurred in a hybridisation oven (Microarray Hybridisation Chambers, Agilent, Santa Clara, CA, USA) at 55 °C for 1 h. The slides were subsequently washed repeatedly, first with 2 × SSC buffer containing 0.1% sodium dodecyl sulphate (SDS) for 5 min, second with 0.2 × SSC buffer for 2 min, and third with ddH_2_O for 1 min. The slides were dried by centrifuging.

### Measurements and data evaluation

The slides were scanned with a Tecan PowerScanner (Männedorf, Switzerland). Slide coating and spotting efficiency were checked by Cy5-labelled LNC-A oligonucleotides, serving as spotting controls, and Cy3-labelled LNC-B oligonucleotides, serving as hybridisation controls, both spotted along with the probes at the same concentrations. The detection oligonucleotides carried biotin-modifications that allowed binding to Alexa-647-labelled streptavidin. The hybridisation controls were measured at 532 nm, all other samples at 647 nm. GenePix Pro 6.0 (Molecular Devices, Sunnyvale, CA, USA) was used for data analyses, including readout of fluorescence intensity counts (FIC), subtraction of the background (average FIC over the unspotted area), and median value calculation of the spotted areas. The graphs were compiled with Origin Lab 8.5 (OriginLab Corporation, Northampton, MA, USA). For the initial probe testing, an R script^65^ was written to assess the significance of the positive signals over all other ones. A z-score based on median values and median absolute deviations was used. For depiction, the lowest variable value was subtracted from all values, rendering all values positive. Therefore, the resulting scale represents standard deviations. The results were depicted as boxplot diagrams. For the subsequent analyses, a value was considered positive if surpassing the mean value of all signals plus one standard deviation.

## Results and discussion

In this work, we developed a DNA microarray-based assay that is capable of genetically identifying and characterising pathogens. The focus was put on the detection of the 44 most important sepsis-relevant bacterial pathogens and their ABR and VF genes. Our assay was compared to a conventional DNA-based microarray and its specificity was assessed analysing 14 sequenced reference strains of the 44 species. The main focus of this work is on the 6 ESKAPE species; all other strains are summed up in the Supplementary Information (Table S2).

### Evaluation of the probe performance

**Figure 2:**
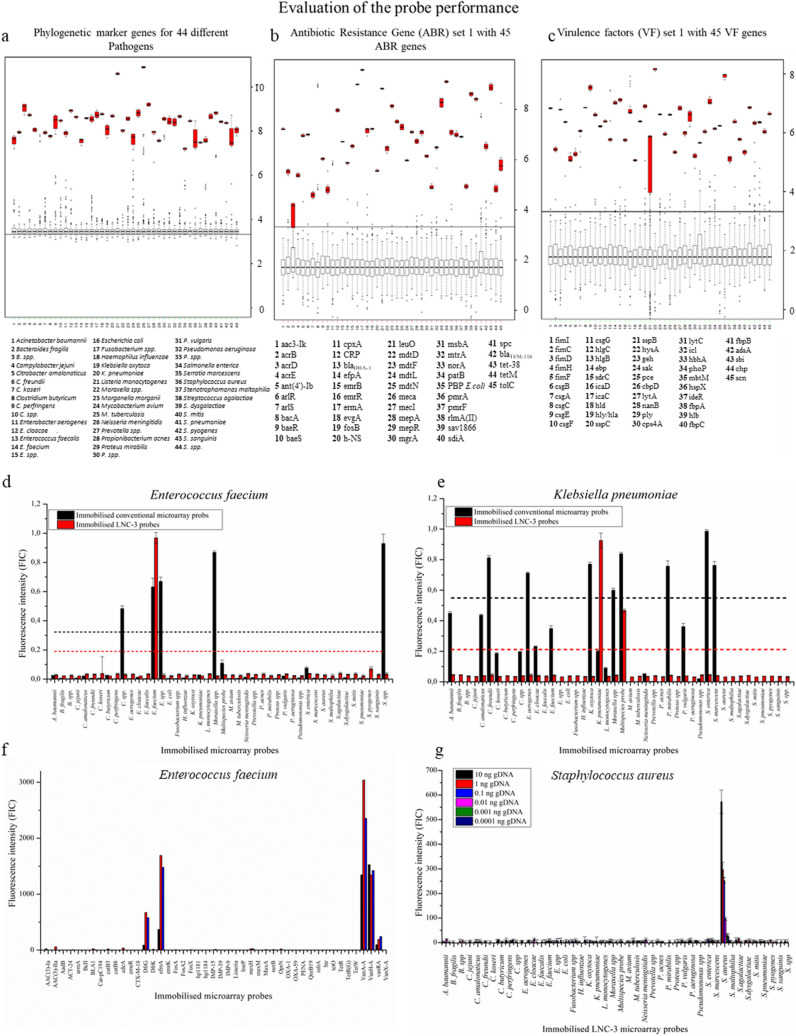
Performance of the LNC-3 probes: A set of 45 LNC-3-probes (and detection oligonucleotides) was tested in terms of specificity by applying complementary synthetic target DNA to the chip surface, the latter carrying all 45 specific parts of 16S rRNA genes serving as phylogenetic markers (**a**), ABR genes (**b**), and VF genes (**c**). All correct signals (red) were evaluated statistically against the signals of all non-matching probes (grey), using median-based z-scores (see Materials and Methods section for more detailed description). In the boxplots, all single values are located inside the boxes with associated error bars representing the standard deviation. The black bars represent the median values. The grey boxes summarise the values of the probes that did not match the gene fragment of interest, the red ones illustrate the positive signals. Statistical analyses and depiction were done with R: A Language and Environment for Statistical Computing^65^ **Identification** via conventional, hybridisation-based detection (black) and LNC-3 technology (red) of *E. faecium* (**d**) and *Klebsiella pneumoniae* (**e**). Black and red dotted lines show the thresholds for positive detection (mean value of all signals plus one standard deviation) in case of the conventional and LNC-3-based signals, respectively. **Reproducibility** of the ABR detection of *E. faecium* with the LNC-3 technique (**f**). **Sensitivity analysis:** Different amounts of target DNA were applied to the LNC-3-functionalised microarrays. 10 ng DNA corresponds to 10^6^ bacterial cells (**g**).

Upon the in silico design of the 814 oligonucleotides, they were first experimentally evaluated with synthetic target DNA to identify non-functional probes (Fig. 2a–c and Figs. S9–S22). The synthetic DNA was 100% complementary, single-stranded, and not dependent on any other factors, such as the upstream PCR amplification, therefore it represented the simplest case. The LNC-3 probes that generated weak fluorescence signals were subject to more detailed in silico secondary structure analyses and reordered to exclude shortcomings during the chemical synthesis. Along with, the detection oligonucleotides were scrutinised.

Figure 2a–c show exemplary results of the microarray probes corresponding to 16S rRNA genes (Fig. 2a), ABR genes (Fig. 2b), and VF genes (Fig. 2c), summarised in boxplot diagrams displaying the median values of four repetitions. The complete set of measurements is given in the Supplementary Information (Figs. S9–S22). The data were normalised to be comparable between different experiments. This was due to different individual factors, such as the in-house slide coating. Briefly, the positive signals, detected at the respective correct spots, are clearly contrasting from the non-matching probe signals, even without statistical evaluation. Having a look at the statistical boxplot analysis, a threshold can easily be drawn to separate positive values (red) from the counts emerging at non-matching spots (grey). All in all, the probes representing phylogenetic 16S rRNA-based markers, ABR genes and VF genes were shown to work well; the probes were sufficiently specific. Including the residual sets of phylogenetic markers, ABR genes, and VF factors, shown in Figs. S9–S21, 97.7% of the designed LNC-3 probes yielded acceptable signals (> tenfold standard deviation). A total of 2.2% of the probes produced fluorescence intensities that were lower than the tenfold standard deviation; however, they were still distinguishable from the background noise.

### Comparison between different microarray technologies

The LNC-3 approach was compared to a conventional DNA microarray method. The conventional method consisted of an immobilised oligonucleotide carrying a sequence complementary to the target DNA hybridising to the immobilised probe. In our alternative approach, the same sequence was split in two parts encoded on the LNC-3 probe and the detection oligonucleotide, so that the target DNA binds the detection oligonucleotide and immobilised probe via hybridisation. Only if subsequently ligated by a proofreading ligase, the detection oligonucleotides were not removed by stringent washing. The performance of those two methods was determined by identifying six different clinically relevant pathogens via their 16S rRNA genes. The results of two representative detection reactions targeting *E. faecium* and *K. pneumoniae* are shown in Fig. 2d,e. The residual results are given in the Supplementary Information (Fig. S7).

While the LNC-3-based microarray chip exhibited a single significant signal only in case of the *E. faecium* probe, the conventional chip surface presented five positive signals (mean value plus one standard deviation). Positive detection occurred for *E. faecium*, *Enterococcus* spp. (consensus sequence for all investigated *Enterococcus* spp.), *Clostridium* spp., *Moraxella* spp., and *Streptococcus* spp. probes (Fig. 2d).

Targeting *Klebsiella pneumoniae* (Fig. 2e), the LNC-3 method clearly identified the species by its corresponding probe and the multispecies probe. The latter LNC-C probe is perfectly matching to the 16S rRNA gene sequences of *Morganella morganii*, *Klebsiella pneumoniae*, *Citrobacter freundii*, *Enterobacter aerogenes, Escherichia coli*, *Pseudomonas aeruginosa* and *Proteus* spp. (explained in detail in Fig. S6), which are closely related and share large parts of their 16S rRNA genes. The signal intensities of the individual signals differ due to thermodynamic differences between species-specific probe/target DNA and multiple species probe/target DNA interactions, manifested in a lower ΔG value. The consequences of having two matching probes is discussed below in the sensitivity section. Concerning specificity, the LNC-3 responses were 100% correct. The conventional microarray analysis showed eight positive signals, including the correct ones. Further a lot of high signals were visible, attributed to unspecific binding, although not surpassing the threshold. The latter was extraordinarily high by the high standard deviation entailed by the unspecific signals. It was not possible to identify a phylogenetic correlation in the false positive signals created by the conventional microarray technique (Figs. S1–S5)^66,67^.

Attempting the identification of *Streptococcus pseudopneumoniae* (Fig. S7), probed together with *S. pneumoniae* at one spot because of > 99% sequence similarity of the 16S rRNA genes^68^, the conventional microarray resulted in one specific and three non-specific signals, namely at the correct *S. pneumoniae* and the related *Streptococcus* spp. spot, but also at the loci of *Moraxella* spp., *Streptococcus mitis*, and the multispecies probe (not containing *S. pseudopneumoniae*). In the LNC-3-based array, only the matching probes of *S. pneumoniae* and *S.* spp. were detected. The relatively high standard deviations were assigned to the in-house slide coating, which was prone to inaccuracies and caused single outlier values contributing to the median. We refrained from eliminating the outlier values to not erase data points. However, using a statistical method to eliminate outliers would be a further possibility to reduce deviations without notably changing the positive values. In general, standard deviations were expected to be reduced upon a future fully automated coating. A similar picture of correct LNC-3-based detection and many additional unspecific signals for the conventional microarray technique emerged for *Citrobacter freundii* (Fig. S7). In case of the *Bacteroides fragilis* probe, as well shown in Fig. S7, the conventional approach resulted in only one, however wrong, positive signal (*Listeria monocytogenes*). A special case was the detection of *Proteus penneri*, for which no perfectly matching probe besides multispecies and interspecies probe was presented on the microarray chip to test indirect detection of non-comprised pathogens by the ssp. probes. Indeed, the maximum signal was obtained at the multispecies probe, while positive signals were obtained for *P. mirabilis* and *P.* spp., which, strictly speaking, should not occur for *P. mirabilis* despite of the very close phylogenetic relationship (Fig. S1). In terms of testing the detection of non-comprised species by interspecies or multispecies probes, the concept was found rather impairing instead of supporting the assay performance in general, as is outlined in the sensitivity section below. The conventional microarray showed positive in case of *Clostridium* spp., the *Moraxella* spp., and the *Serratia marcescens* probes in addition, which are not very related. In conclusion, except from one additional signal of a closely related species (*P. mirabilis* using *P. penneri* target DNA), the LNC-3-based microarray showed correct responses in terms of pathogen identification in any case, while the conventional approach resulted in frequent false-positive signals, many of which at spots of completely unrelated pathogens.

### Reproducibility

The reproducibility of the microarray system was evaluated with this experiment as well. The reproducibility is a known problem with microarray technology^69–72^. In contrast to scientific research, there are no repetitions performed in clinical diagnostics usually. For that reason, the correct signals must be obtained reliably, not in the average of repeated measurements. In Fig. 2f, three repetitions of an ABR gene set detection of *E. faecium* is shown. Although the absolute intensities of the individual measurements differ greatly from one another due to the in-house slide coating, they exhibited significant signals towards their individual backgrounds and consequently generated a uniform statement about the resistance genes of the strain. As mentioned before, an automated slide coating could most likely reduce the standard deviations drastically and therefore further enhance the reproducibility.

### Sensitivity test of the LNC-3 microarray technology

The higher complexity of the employed reaction, comprising several additional steps, suggests that this gain of specificity could be accompanied by a loss of sensitivity. The total fluorescence signal intensity was found to be lower with the LNC-3 method than with the conventional microarray. For that reason, the sensitivity of the LNC-3-based microarray was determined using a target DNA dilution series (0.1 pg–10 ng) of *S. aureus* in the amplification reaction. The resulting PCR products were applied to the chip (Fig. 2g). 1 ng DNA corresponds to 10^5^ bacterial cells per mL (e.g. of blood). With 0.1 pg and 1 pg of target DNA, no signal differentiable from the background noise was obtained. 10 pg, corresponding to 10^3^ cells per mL, led to a significant signal compared to the background. 0.1, 1, and 10 ng resulted in higher signals. Hence, thousand pathogen cells could be detected, enabling the LNC-3 technology to compete with other pathogen detection formats^73,74^. The threshold of 10 pg DNA was achieved with other target DNA sources as well, such as *E. faecalis*. In two cases, the sensitivity was determined to be 100 pg, corresponding to 10^4^ cells per mL, i.e. *Escherichia coli* and *Enterococcus faecium*. (Fig. S8). This was assigned to the competitive reaction of the target DNA to two corresponding probes. In the case of *E. coli*, the target DNA might bind to the specific *E. coli* probe or to the multispecies probe. The target DNA of *Enterococcus faecium* was distributed between the specific *E. faecium* probe and the *E.* spp. probe. These results pointed out that the idea of interspecies probes to cover a broader pathogen range was counterproductive. While the multispecies probe showed the major signal, the signal at the specific *E. coli* spot was of critically low intensity. ΔG value analyses revealed that the multispecies probe exhibited a lower ΔG value for the multispecies target DNA/probe complex than for the *E. coli* probe/target DNA complex. This might well explain the higher multispecies probe signal but rendered the use of interspecies probes not reasonable. The same situation applied for the *E. faecium* sample. Here, more similar ΔG values led to a more equal signal distribution between the matching probes. However, it was shown that the LNC-3 approach is capable of detecting DNA amounts corresponding to 10^3^ cells per mL, whereat multispecies or interspecies probes impaired that sensitivity. Therefore, future chip generations will favour single matching probes over grouped ones.

### Full characterisation of bacterial isolates

**Figure 3:**
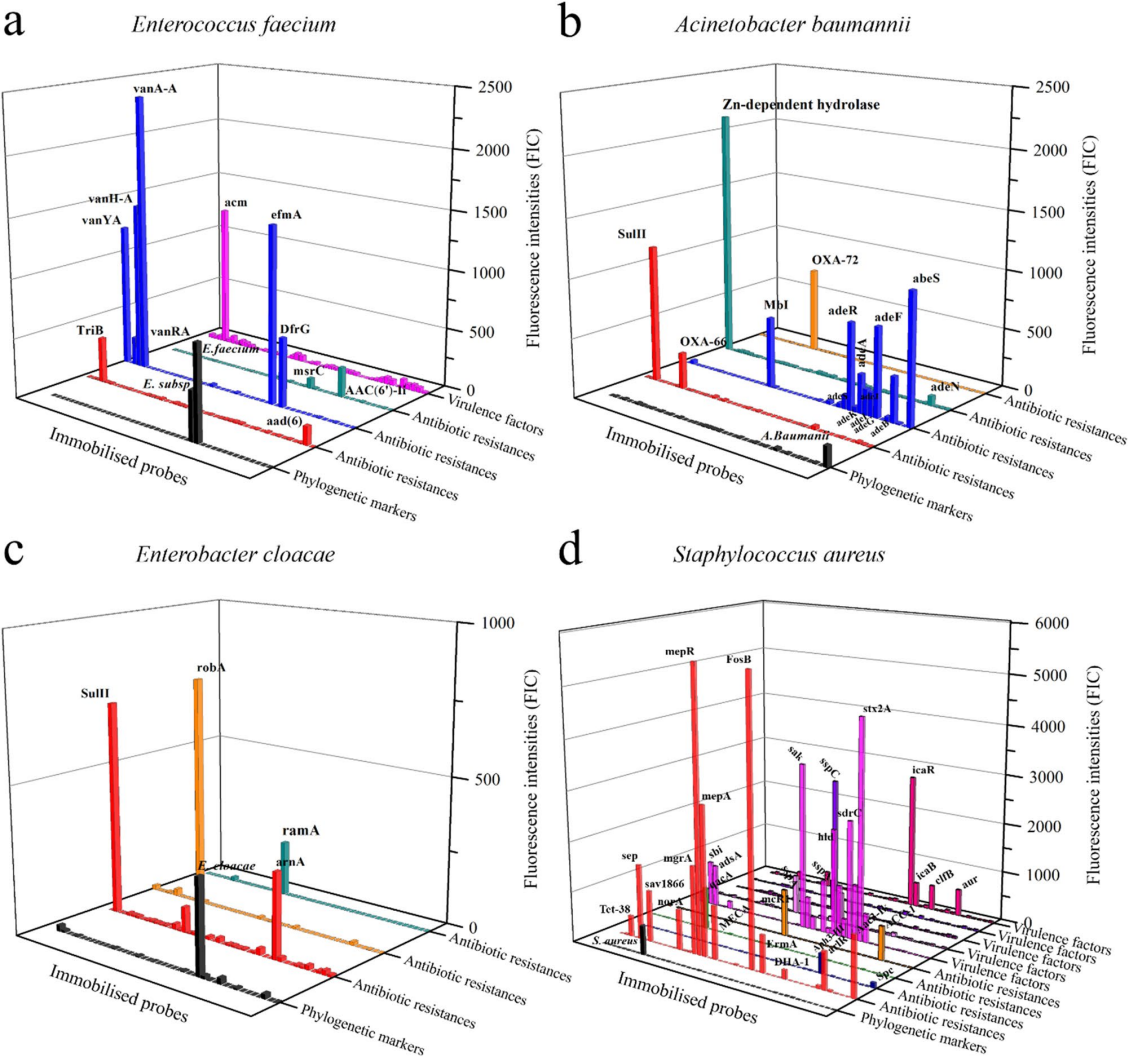
Full characterisation of *Enterococcus faecium* (**a**), *Acinetobacter baumannii* (**b**), *Enterococcus cloacae* (**c**), and *Staphylococcus aureus* (**d**) isolates, comprising identification (black) via 16S rRNA genes and characterisation in terms of ABR genes (red/blue/green/orange) and VFs (magenta and related colors). The DNA of a clinical isolate carrying the specified pathogen was purified and amplified by multiplex PCRs including the primer pairs of all investigated genes. The amplification products were applied to the microarray chips along with detection oligonucleotides. If matching, the detection oligonucleotides could be ligated to the probes and subsequently detected by a standard fluorescence-based microarray scanner.

The LNC-3 microarray platform was verified by characterisations of the six pathogens abbreviated with ESKAPE (Enterococcus faecium, Staphylococcus aureus, Klebsiella pneumoniae, Acinetobacter baumannii, Pseudomonas aeruginosa and Enterobacter cloacae). Those species have been particularly highlighted by the Infectious Diseases Society of America for being especially problematic in terms of ABR^6,7^. To validate the results, the genomes of the used ESKAPE strains were, if publicly not available, sequenced and bioinformatically assessed beforehand. The results are summarised in Table 2. In total, 14 pathogenic strains were characterised. Figure 3 shows the characterisations of clinical isolates from E. faecium (a), A. baumannii (b), E. cloacae (c), and S. aureus (d). Those data and the characterisation data of the two remaining ESKAPE pathogens K. pneumoniae and P. aeruginosa are additionally listed in Table 2. Expected genes whose corresponding probes did not exhibit sufficient signal intensities are highlighted in bold letters. Additionally identified ones are marked in italics. The residual characterisations are given in the Supplementary Information (Table S2).

**Table 2:** Characterisation of bacterial isolates using LNC-3 probes targeting ABR genes and VF genes. LNC-3-derived signals are compared with whole genome sequencing data. Genes found in sequence data but without LNC-3 response are indicated in bold letters, LNC-3 responses that were not found via sequencing are written in italics.

Pathogen	Detected pathogen	ABR genes		VF genes	
		Sequenced	LNC-3	Sequenced	LNC-3
*Acinetobacter baumannii*	*Acinetobacter baumannii*	**aadA**, OXA-66, SulI, Mbl abeS, adeC, adeJ, adeS, adeB, adeA, adeK, adeG, ADC-2, BlaA1, adeF, adeR, adeI, adeN, Zn-dependent hydrolase, **AAC(3)-Ia**, OXA-72, BlaA2	OXA-66, SulI, Mbl abeS, adeC, adeJ, adeS, adeB, adeA, adeK, adeG, ADC-2, BlaA1, adeF, adeR, adeI, adeN, Zn-dependent hydrolase, OXA-72, BlaA2		
*Enterobacter cloacae*	*Enterobacter cloacae*	ramA, SulII, robA	ramA, SulII, robA, *arnA*		
*Enterococcus faecium*	*Enterococcus faecium, Enterococcus spp.*	AAC(6′)-Ii, msrC, efmA	AAC(6′)-Ii, msrC, efmA, *DfrG, VanA-A, VanH-A, vanRA, vanYA, TriB, aad(6)*	acm	acm
*Klebsiella pneumoniae*	*Klebsiella pneumoniae*	oqxA, **FosA5**, **vgaC** acrA Klebsiella, oqxB	oqxA, acrA Klebsiella, oqxB	yagZ/ecpA, **east1_astA**	yagZ/ecpA
*Pseudomonas aeruginosa*	*Multispecies probe, Pseudomonas aeruginosa*	**PDC-1**, amrA, amrB **Aph3-IIb**, arnA, basS, **CatB7**, CpxR, **MexA**, MexB, MexD, **MexE**, **MexF**, mexG, mexI, mexJ, mexK, mexL, mexP, mexQ, mexV, mexW, **MuxB**, **MuxC**, OpmB, **opmD**, opmE, OpmH, OprJ, OprM, OXA-50, TriB, TriC, **OprN**, mexH, FosA, MuxA, mexM, **MexC**, **TriA**	amrA, amrB, arnA, basS, CpxR, MexB, MexD, mexG, mexI, mexJ, mexK, mexL, mexP, mexQ, mexV, OpmB, opmE, OpmH, OprJ, OprM, OXA-50, TriB, TriC, mexH, FosA, MuxA, mexM,	xcpA/pilD, algB, algQ, algZ, algU, alg8, alg44, algE, algX, algL, algF, algA, mucC, waaG, waaC, aprA, lasA, lasB, rhlI, lasI, plcH, xcpZ, xcpV, **xcpT**, xcpS, xcpP, xcpQ, pilY2, **pilS**, pilR, pilP, **pilM**, pilT, pilU, pilG, **pilH**, pilI, chpB, chpC, flgD, flgG, flgH, flgI, flgJ, fleQ, fleR, **fliE**, **fliG**, fliI, fliJ, fliM, fliN, fliP, fliQ, flhB, **flhA**	xcpA/pilD, algB, algQ, algZ, algU, alg8, alg44, algE, algX, algL, algF, algA, mucC, waaG, waaC, aprA, lasA, lasB, rhlI, lasI, plcH, xcpZ, xcpV, xcpS, xcpP, xcpQ, pilY2, pilR, pilP, pilT, pilU, pilG, pilI, chpB, chpC, flgD, flgG, flgH, flgI, flgJ, fleQ, fleR, , fliI, fliJ, fliM, fliN, fliP, fliQ, flhB,
*Staphylococcus aureus*	*Staphylococcus aureus*	Aac3-Ik, arlR, **arlS**, DHA-1, ErmA, FosB, MECA, mepA, mepR, mgrA, norA, sav1866, Spc, Tet-38, Aph3-III, qacA, mecR1	Aac3-Ik, arlR, DHA-1, ErmA, FosB, MECA, mepA, mepR, mgrA, norA, sav1866, Spc, Tet-38, Aph3-III, qacA, mecR1, *ACC-1*	hlgC, hlgB, ebp, sdrC, icaD, icaC, hld, hly/hla, sppC, sppB, hysA, geh, sak, hlb, adsA, scn, sdrD, sdrE, clfA, map, sea, sppA, icaA, fnbA, icaR, icaB, clfB, aur	hlgC, hlgB, ebp, sdrC, icaD, icaC, hld, hly/hla, sppC, sppB, hysA, geh, sak, hlb, adsA, scn, sdrD, sdrE, clfA, map, sea, sppA, icaA, fnbA, icaR, icaB, clfB, aur

During the identification of *E. faecium*, two positive signals were generated, one at the probe corresponding to *E.* *faecium* and one at the probe corresponding to *Enterococcus* spp. with a lower fluorescence signal (Fig. 3a: black)*.* Furthermore, the characterisation revealed the presence of the ABR genes *AAC(6′)-Ii, msrC, efmA, DfrG, VanA-A VanH-A, vanRA, vanYA, TriB,* and *aad(6)* (Fig. 3a: red, blue and green) and the VF gene *acm* (Fig. 3a: magenta). These results were compared with the shotgun sequencing data. It turned out that with the genes *DfrG, VanA-A VanH-A, vanRA, vanYA, TriB, aad(6)* had been identified in addition using the LNC-3 method. To ensure that the additional hits of ABR gens are not a result of measurement outliers, the measurement was repeated three times with the ABR gene panel comprising *DfrG* and the vancomycin-related resistance genes (shown in Fig. 2f and discussed in detail there) to exclude that. Those additional ABR genes were detected repeatedly. A number of possible explanations can be thought of, such as the binding of similar target DNA. However, *Enterococcus* spp. are known to frequently carry vancomycin resistance genes. A detection of the latter was hence not surprising. A conceivable mechanism of the vancomycin-related resistance genes escaping notice in whole genome shotgun sequencing data is their spread on transposable elements^75,76^. The assembly of larger sequence parts from contiguous motifs (contigs) may suffer from the direct repeats that enclose transposable elements and integrons, leading to sequences that are not assembled in silico correctly. In cases such as the vancomycin resistances, it was hence assumed that rather the WGS shotgun data were incomplete. Nevertheless, all genes revealed by WGS were found using the LNC-3 technique.

The probe corresponding to *A. baumannii*, albeit showing a relatively low absolute intensity, contrasted clearly from all other signals, which did not exceed the background noise (Fig. 3b). The sequence data did not reveal the presence of any VF genes, but 22 different ABR genes were identified (*aadA,* bla_*OXA-66*_*, sulI, mbl, abeS, adeC, adeJ, adeS, *adeB*, adeA, adeK, adeG adc-2, blaA1, adeF, adeR, adeI, adeN, Zn-dependent hydrolase, aac(3)-Ia,* bla_*OXA-72*_*, blaA2*). The signal intensities of the genes *aadA* (*aad* genes encode adenylases) and *aac(3)-Ia* (*aac* genes encode acetylases), both encoding enzymes to break down aminoglycosides, were not strong enough for an unequivocal response. All others were successfully detected. Regarding the VF genes, no false-positive signals were measured. An emerging pattern of non-detected ABR genes is discussed along with the *P. aeruginosa* results.

The probes corresponding to *Enterobacter cloacae* were detected with a signal significant towards the background, (Fig. 3c). *E. cloacae* lacked VF genes completely; the three ABR genes (*ramA, SulII, robA*) were detected along with an additional one (*arnA*).

In the case of the identification and characterisation of *S. aureus* (Fig. 3d), only the matching probe produced a signal. As mentioned before, the absolute signal strength was comparable only among the probes at one panel, not between different panels. Indeed, the signal of the phylogenetic probe detecting *S. aureus* was significant compared to the background, as it can be deduced from Fig. 2d as well. There, it was shown that the sensitivity was actually high enough down to a bacterial concentration comparable to other detection methods. According to the sequencing data, this strain carried 17 ABR genes and 28 VF genes. Only one ABR gene could not be detected with a significant signal strength: *arlS*. Although there was no straightforward reason that this single ABR gene was not detected, there are a number of possible explanations as well as strategies to prevent detection failures given in the *P. aeruginosa* section along with a more detailed discussion of (operon-)related genes.

A similar bigger picture emerged for *K. pneumoniae* (Table 2). *K. pneumoniae* was unequivocally identified, with several ABR and VF genes detected. However, of five ABR genes (*oqxA, vgaC, fosA5, acrA Klebsiella & oqxB*) and two VF genes (*yagZ/ecpA & east1_astA*) present according to the sequencing data, the signal intensities for the genes *fosA5, vgaC*, and *east1_astA* were not sufficient to be considered a positive signal.

For *P. aeruginosa* (Table 2), an approximately three-fold signal intensity at the *P. aeruginosa*, *P.* spp., and the multispecies probe loci indicated their presence. Regarding ABR genes, 40 were revealed by sequencing, while only 27 were detected using the LNC-3 chip. A total of 48 of 55 VFs could be detected. Questioning the low success rate, it was found that the undetected genes were mostly single-copy, genome-encoded genes, suggesting that the previous amplification was not efficient enough to provide the required amount of target DNA. Therefore, this issue must be addressed mainly via the upstream PCR. Since those genes mostly occur in operons or encode multidomain proteins, another option of improving the assay was conceived, which is described in the following.

In summary, 85% of all ABR genes and 83% of the VF genes present in the clinical isolates were identified using bacterial DNA extracts as templates. Only 1.6% false-positives were detected in case of the ABR genes, and none at all for the VF genes. This was mainly attributed to low amplification efficiencies of these individual genes in the multiplex PCR. Solutions to overcome this issue—since increasing the template DNA cannot trivially be done by increasing the overall target DNA amount, because too much DNA inhibits the PCR—could be employing a pre-PCR only amplifying those genes first. However, this could result in new amplification biases also heavily impacted by different background DNAs and would require extensive experimental optimisation. Also, unintended primer interactions in the multiplex reaction could be causative for the different amplification efficiencies. An approach to reduce such interactions using crosslinked primers and steric hindrance effects was published by our group recently^77^.

Another way to improve the interpretation of the microarray results is to consider the presence of ABR or VF operons. Many of those genes, especially efflux pumps, are encoded in operons, e.g. the MexAB-OprM operon, the MuxABC-OpmB, meaning that they are governed by a single promotor regulating their expression one after the other at a time^78–80^. Those genes are not only connected on the genetic level, but often encode related proteins that build, for instance, a single efflux pump, which might confer resistance to antibiotics of different classes if overexpressed. Hence, it might suffice to detect one gene per operon. Having detected mexA and oprM in the case of *P. aeruginosa* sufficed to deduce the presence of mexB as well. Another example was mentioned in the course of the *S. aureus* characterisation. The undetected *arlS* gene is located at the arlR-arlS locus, which encodes a regulatory system of two elements, regulating the *norA* efflux pump gene promoter amongst others^81,82^. Both, *norA* and *arlR*, were detected successfully indicating the presence of the operon. A similar picture emerged for the VFs. The genes that escaped detection encode mainly single components of multidomain flagellar and pili proteins. For instance, discussing the *P. aeruginosa* results, *pilM* is a part of the type IV pili among a lot others, such as *pilT* and *pilP*, which were detected^83^. *pilH* is embedded in an operon along with *pilG*, *pilI*, and *pilJ*^84^. The same applies for the flagellar proteins *fliE, fliG, fliI, fliJ, fliM, fliN* and more^85^.

Hence, the presence of several VF and ABR genes that escaped detection could be derived using operon-based microarray evaluations. Even simpler, the amount of target DNA per probe could be increased by collecting the probes that encode functional units at only one spot, capturing all the related target DNA molecules to generate one, stronger signal.

## Conclusion

It was shown that the LNC-3 concept is superior towards conventional microarray techniques regarding its specificity by using a highly specific ligase reaction instead of relying on hybridisation bonds that often lead to non-specific cross-hybridisation. By screening for 44 pathogens, 360 VF genes and 409 ABR genes at the same time, the number of genes that can be detected simultaneously could be tremendously increased, which is also owed to the combination with the Oli2go software, reducing critical interactions in upstream amplification reactions. The LNC-3 microarray technique was further employed to fully characterise 14 pathogens, so that the most effective treatment can be chosen without relying on empirical data, which become more and more uncertain due to the rapid dissemination of ABR genes, e.g. via horizontal gene transfer. Results could be obtained within one working day. In future studies, the sensitivity of the assay including the sample pre-processing steps has to be further improved in order to directly analyse clinical samples such as blood with pathogen concentrations in the range of 10^0^–10^3^ cells/mL.

## Supplementary Information

Supplementary Information
