# Supplementary material for: Full pathogen characterisation: species identification including the detection of virulence factors and antibiotic resistance genes via multiplex DNA-assays
Source: Sci Rep. 2021 Mar 16;11:6001. doi: 10.1038/s41598-021-85438-5 (PMC7966752; doi:10.1038/s41598-021-85438-5)
Supplement: Supplementary file 1 — Supplementary Information [file 41598_2021_85438_MOESM1_ESM.docx]

**Supplementary Information**

# Full pathogen characterisation: Species identification including the detection of virulence factors and antibiotic resistance genes via multiplex DNA-assays

Noa Wolff^1^*, Michaela Hendling^1^, Fabian Schroeder^2^, Silvia Schönthaler^1^_,_ Andreas F. Geiss^3^, Branka Bedenic^4^, Ivan Barišić^1^

^1^ Molecular Diagnostics, AIT Austrian Institute of Technology, Giefinggasse 4, Vienna, 1210 Austria

^2^ Ekorefugium, Slunj, Croatia

^3^ University of Natural Resources and Life Sciences, Vienna, Austria

^4^ Department of Microbiology, School of Medicine, University of Zagreb, Zagreb, Croatia

* To whom correspondence should be addressed. Tel: +43 664 88390643; Fax: +43 0 50550 4450; Email: [Noawolff@outlook.de](mailto:Noawolff@outlook.de)

Keywords: Antibiotic resistance; Pathogen identification; High-throughput detection; DNA microarray; Multiplex detection

Outline

[Strains and cultivation of bacterial cells 3](#_Toc52301420)

[‘The All-Species Living Tree’ Project (ARB-SILVA) 4](#_Toc52301421)

[Multispecies probe 8](#_Toc52301422)

[Comparison between the conventual microarray technique and the LNC-3 microarray technology 9](#_Toc52301423)

[Sensitivity test of the LNC-3 microarray technology 10](#_Toc52301424)

[Evaluation of the probe performance 11](#_Toc52301425)

[Characterisation of bacterial isolates 18](#_Toc52301426)

[Phylogenetic marker genes 20](#_Toc52301427)

[Antibiotic resistance genes (ABR) 24](#_Toc52301428)

[ABR-1 24](#_Toc52301429)

[ABR-2 31](#_Toc52301430)

[ABR-3 38](#_Toc52301431)

[ABR-4 45](#_Toc52301432)

[ABR-5 51](#_Toc52301433)

[ABR-6 58](#_Toc52301434)

[ABR-7 66](#_Toc52301435)

[ABR-8 73](#_Toc52301436)

[ABR-9 80](#_Toc52301437)

[Virulence factor genes (VF) – primers and probes 87](#_Toc52301438)

[VF-1 87](#_Toc52301439)

[VF-2 92](#_Toc52301440)

[VF-3 100](#_Toc52301441)

[VF-4 109](#_Toc52301442)

[VF-5 115](#_Toc52301443)

[VF-6 123](#_Toc52301444)

[VF-7 130](#_Toc52301445)

[VF-8 137](#_Toc52301446)

## Strains and cultivation of bacterial cells

Supplementary Table S1: A detailed description of media, conditions, and origin of the detected pathogens.

| **No.** | **Species** | **Strain** | **Medium** | **Condition** |
| --- | --- | --- | --- | --- |
| 1 | Acinetobacter baumannii | A_baumannii_5M | TSB | 37°C, shaking |
| 2 | Bacteroides fragilis | B_fragilis_M_7021 | TSB | 37°C, shaking |
| 3 | Enterobacter aerogenes | E_aerogenes_Pula_122664 | TSB | 37°C, shaking |
| 4 | Enterobacter cloacae | DSM30054 | TSB | 37°C, shaking |
| 5 | Enterococcus faecium | ATCC700221 | TSB | 37°C, shaking |
| 6 | Escherichia coli | DSM30083 | TSB | 37°C, shaking |
| 7 | Haemophilus influenzae | DSM4690 | BA | 37°C microaerophilic |
| 8 | Klebsiella pneumoniae | DSM30104 | TSB | 37°C, shaking |
| 9 | Prevotella bivia | DSM20514 | CDC Anaerobe agar | 37°C, anaerobe |
| 10 | Proteus mirabilis | P_mirabilis_P_1 | TSB | 37°C, shaking |
| 11 | Pseudomonas aeruginosa | DSM50071 | TSB | 37°C, shaking |
| 12 | Salmonella enterica sv. typhi | DSM554 | TSB | 37°C, shaking |
| 13 | Staphylococcus aureus | MRSA_18242 | TSB | 37°C, shaking |
| 14 | Streptococcus pseudopneumoniae | DSM18670 | TSBY | 37°C microaerophilic |

| CDC anaerobe agar: | BD CDC Anaerobe Agar with 5% Sheep Blood, Cat. No. 256506 |
| --- | --- |
| BA: | BD Columbia Agar with 5% Sheep Blood, Cat. No. 254005 |
| TSB: | Tryptic Soy Broth, Cat. No. 1.05459.0500, Merck KGaA |
| TSBY: | Tryptic Soy Broth, 3 g/L yeast extract |

## ‘The All-Species Living Tree’ Project (ARB-SILVA)


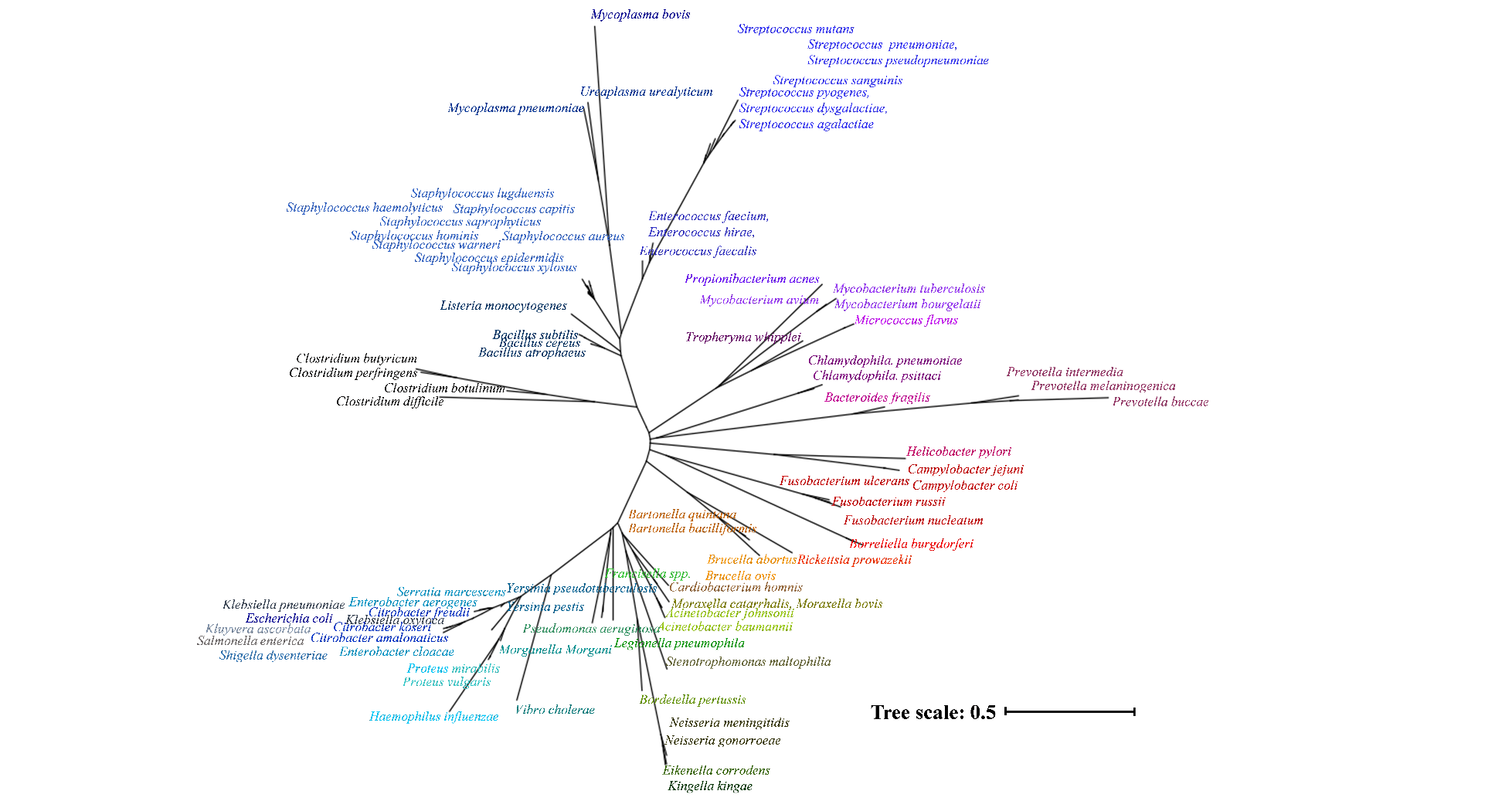


Supplementary Figure S1: ‘The All-Species Living Tree’ Project (ARB-SILVA^1^): Overview. The surrounded parts are magnified in the following figures.

^1^Yarza, P. *et al.* Update of the All-Species Living Tree Project based on 16S and 23S rRNA sequence analyses. Systematic and applied microbiology 33, 291–299 (2010).


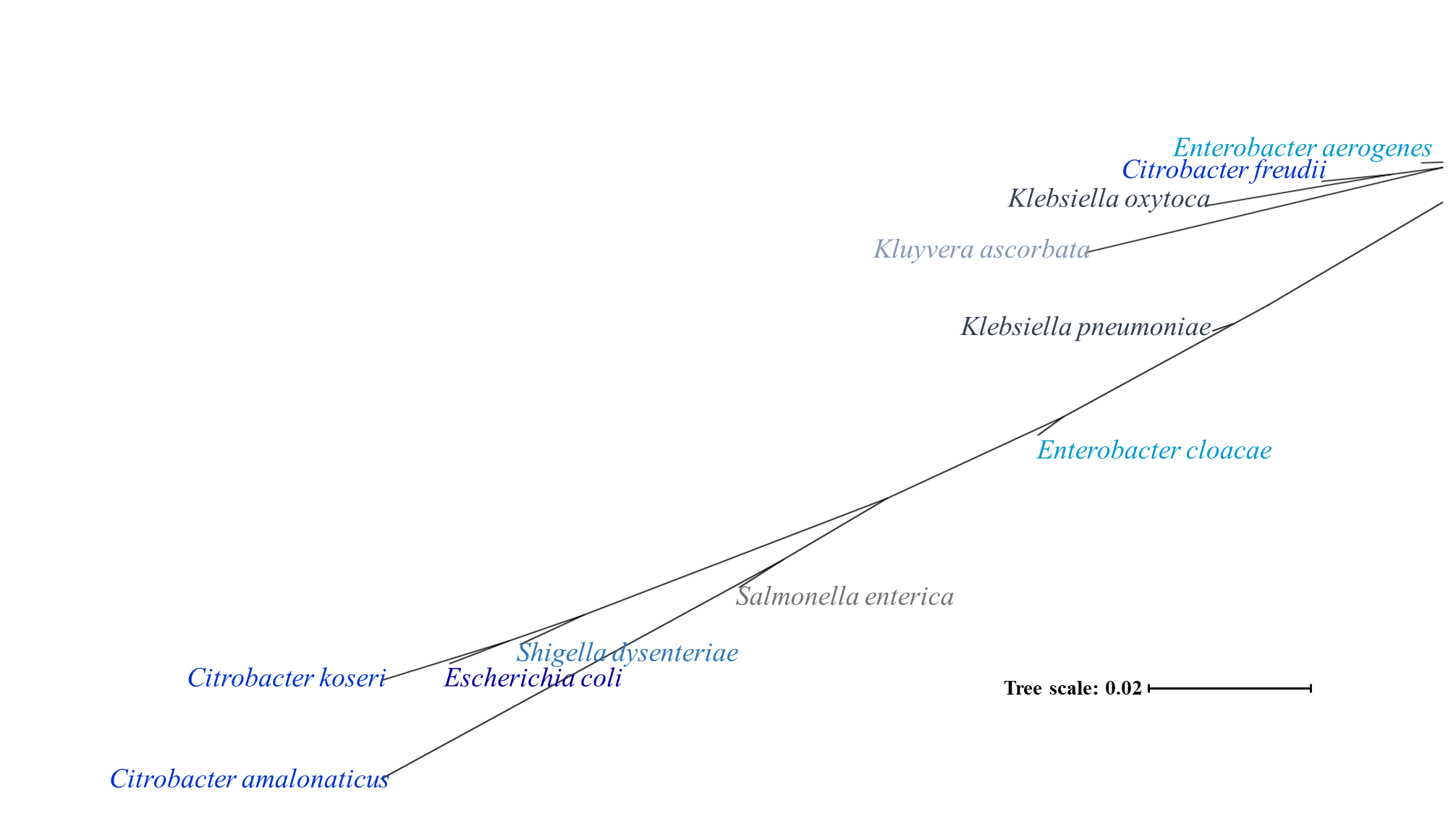


Supplementary Figure S2: ‘The All-Species Living Tree’ Project (ARB-SILVA^1^): Insert Enterobacteriaceae (Gammaproteobacteria).


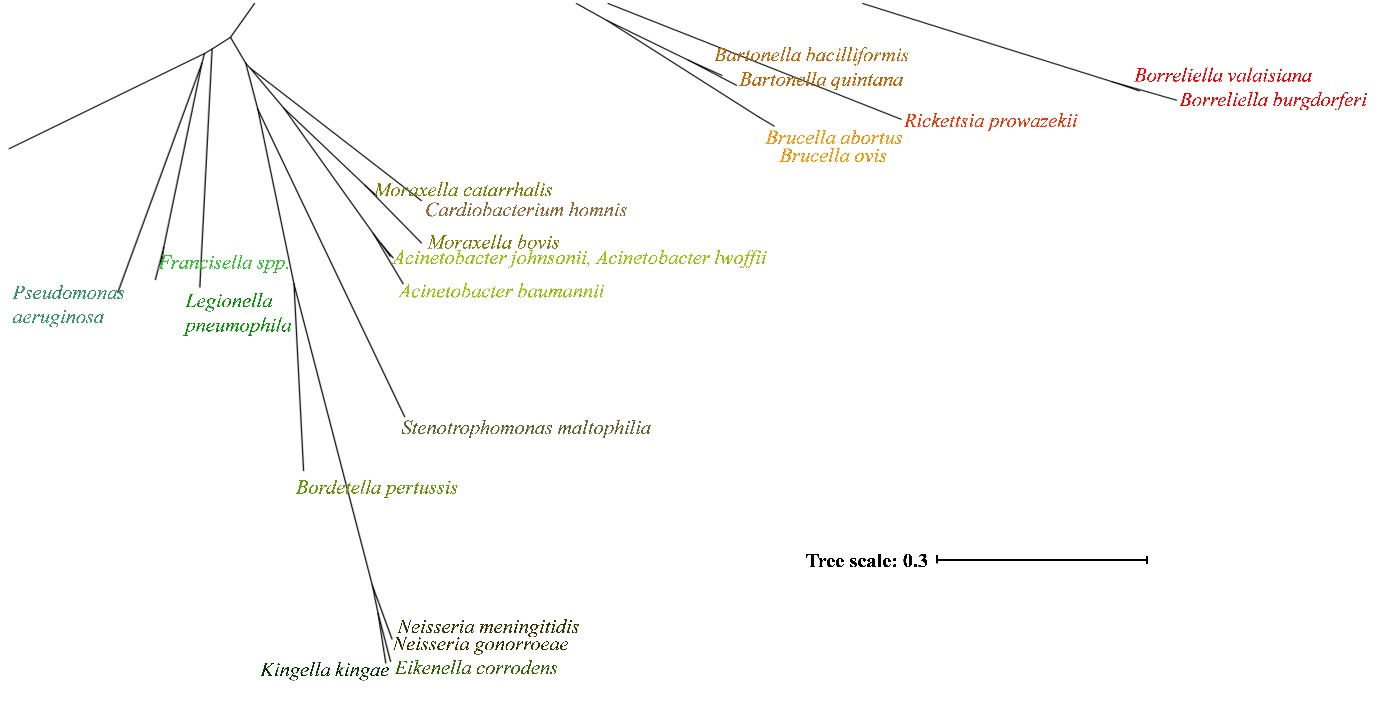


Supplementary Figure S3: ‘The All-Species Living Tree’ Project (ARB-SILVA^1^): Insert Gammaproteobacteria.

^1^Yarza, P. *et al.* Update of the All-Species Living Tree Project based on 16S and 23S rRNA sequence analyses. Systematic and applied microbiology 33, 291–299 (2010).


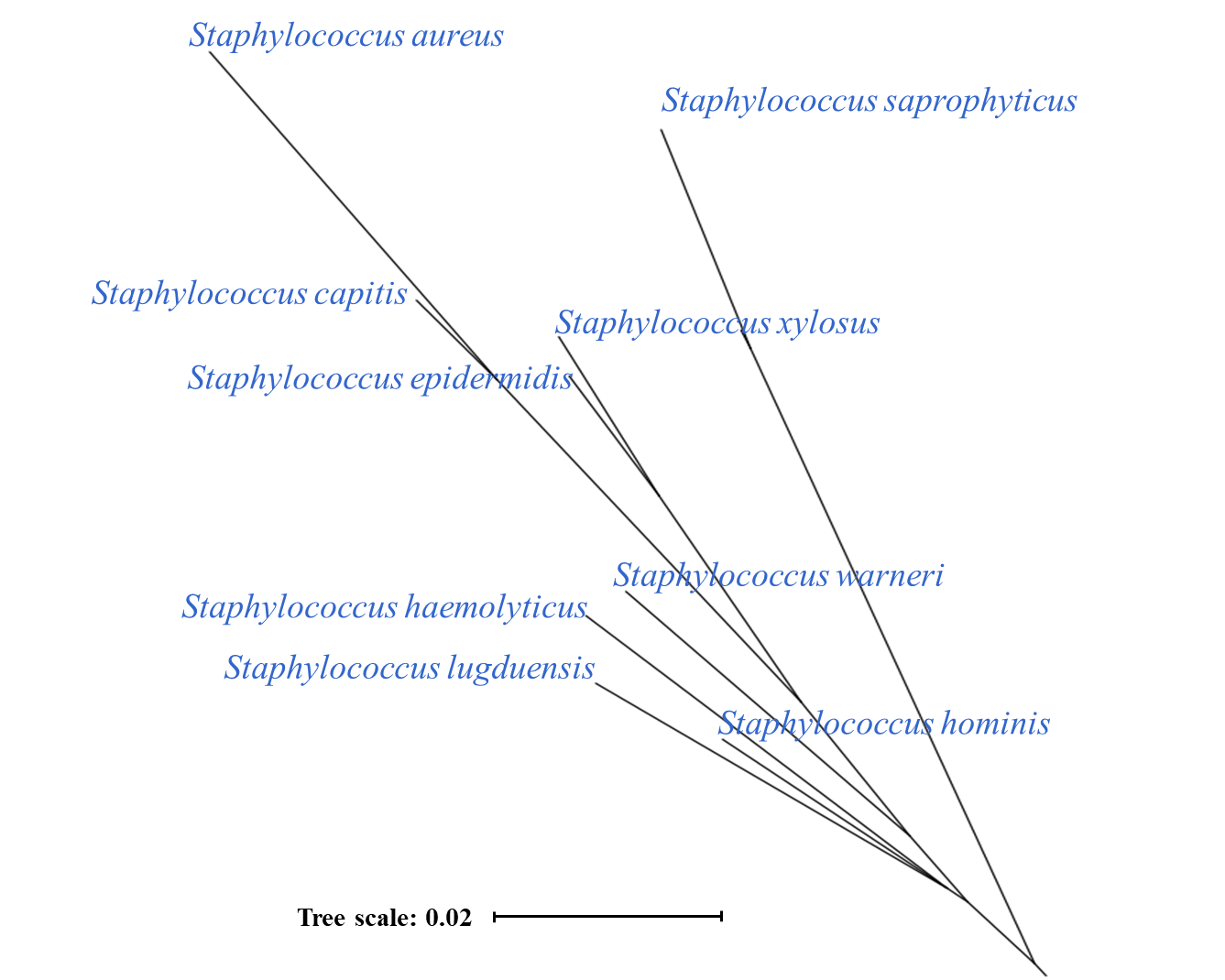


Supplementary Figure S4: ‘The All-Species Living Tree’ Project (ARB-SILVA^1^): Insert *Staphylococcus* (Bacilli).

^1^Yarza, P. *et al.* Update of the All-Species Living Tree Project based on 16S and 23S rRNA sequence analyses. Systematic and applied microbiology 33, 291–299 (2010).


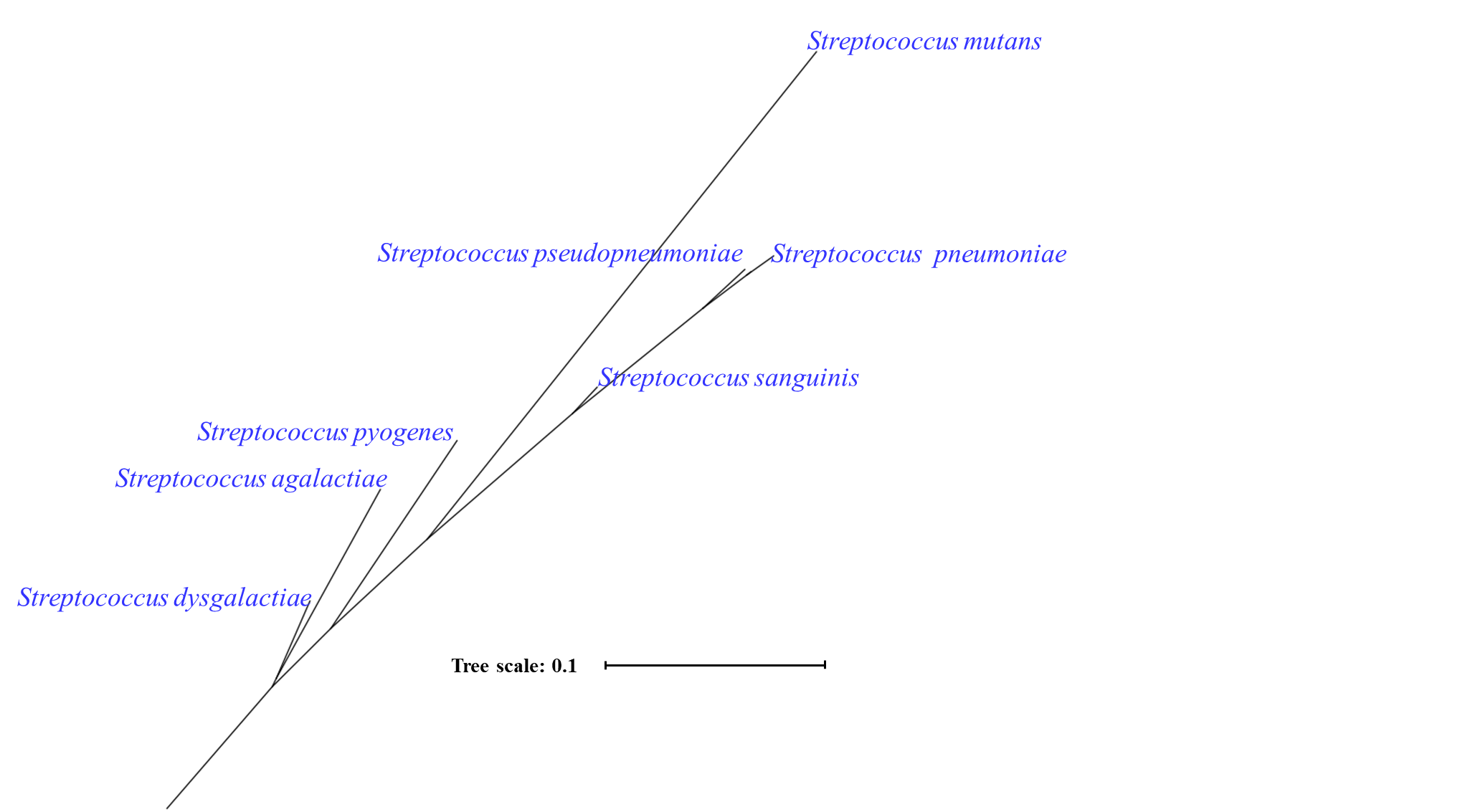


Supplementary Figure S5: ‘The All-Species Living Tree’ Project (ARB-SILVA^1^) insert *Streptococcus* (Bacilli).

^1^Yarza, P. *et al.* Update of the All-Species Living Tree Project based on 16S and 23S rRNA sequence analyses. Systematic and applied microbiology 33, 291–299 (2010).

## Multispecies probe


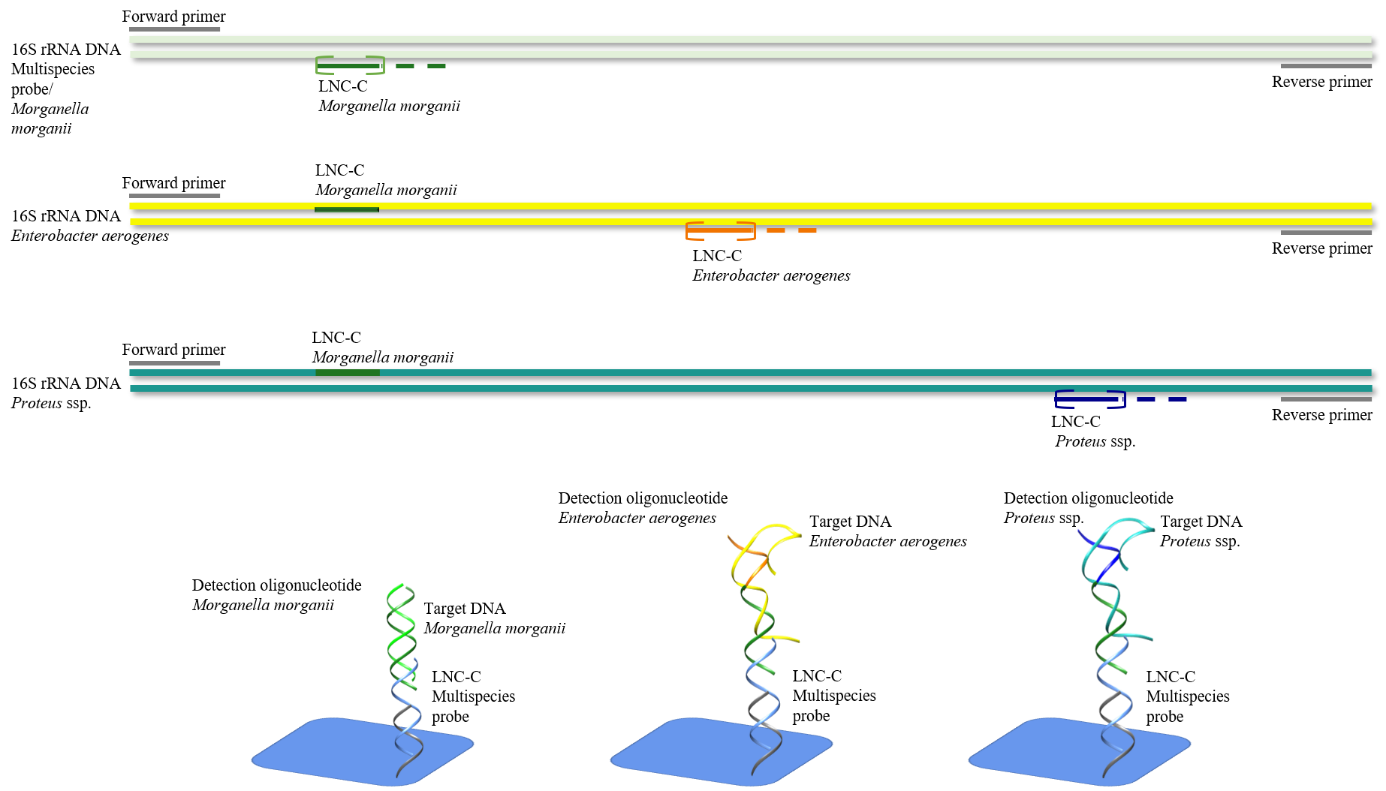


Supplementary Figure S6: Schematic depiction of the loop formation enabling several detection oligonucleotides to bind to the *M. morganii* probe. Top: A specific region of the 16S rRNA DNA was amplified by PCR using consensus sequences for the primers (grey). The amplicon (light green for *M. morganii*, yellow for *E. aerogenes*, turquoise for *Proteus* ssp.) carried species-specific parts (green for *M. morganii*, orange for *E. aerogenes*, blue for *Proteus* ssp.), of which the complementary sequence was chosen for the LNC-C probe (solid line) and the following section (dashed line) for the detection oligonucleotides. For example, the LNC-C part of *M. morganii* was also present at the other amplicons (not the detection oligonucleotide complementary sequence, so this was not influencing the probe testing using synthetic DNA). Bottom: For the given examples, it is shown how loop formation enables the detection oligonucleotides of all three to the *M. morganii* probe (even simplified by a lower ΔG value for the non-desired combinations). On the other hand, *Enterobacter aerogenes* and *Proteus* ssp. could bind to the respective other probes, making the latter a multispecies probe. The especially low ΔG value should be used to detect these bacteria with high sensitivity.

## Comparison between the conventual microarray technique and the LNC-3 microarray technology

*
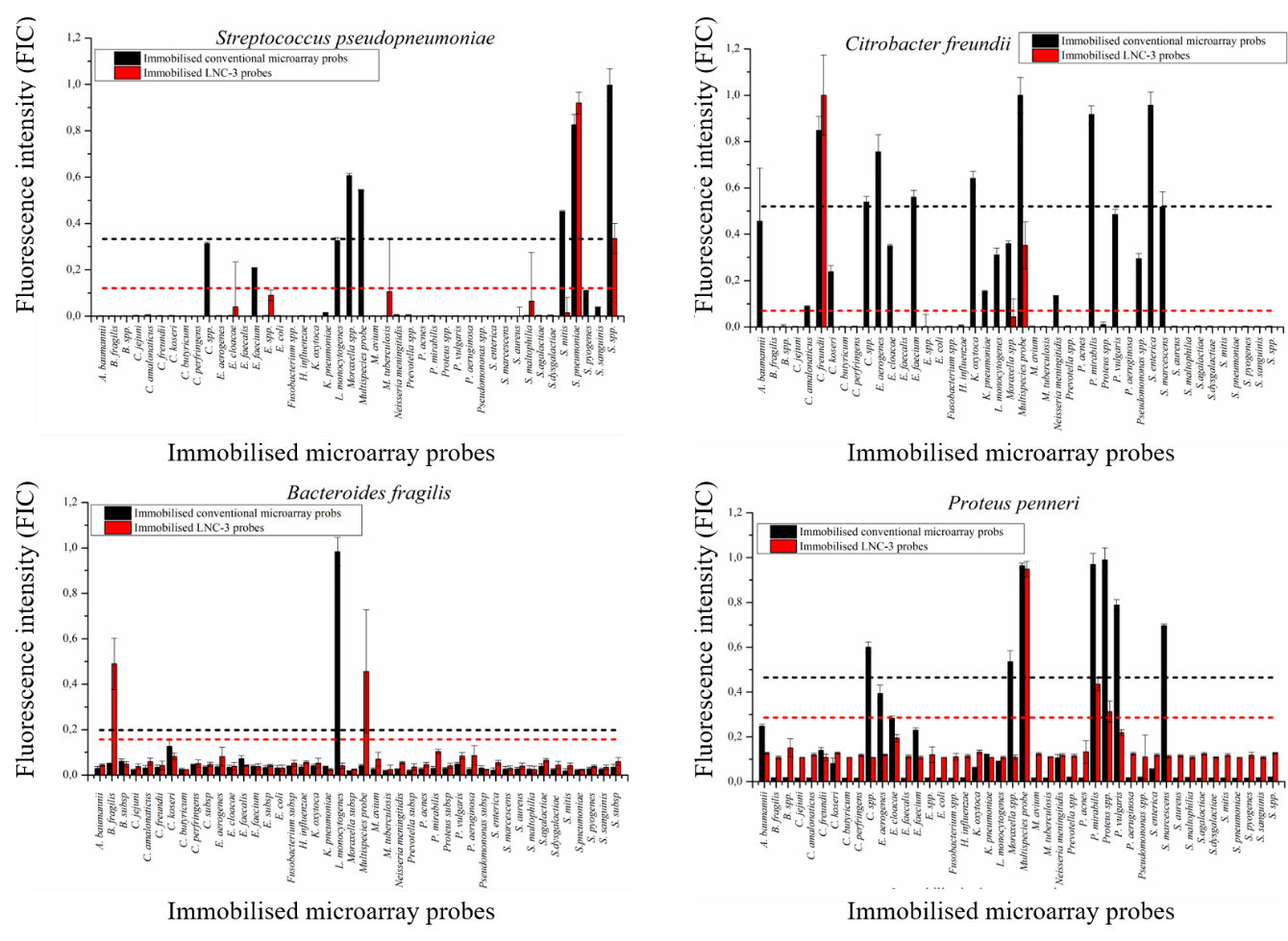
*

Supplementary Figure S7: Comparison between the conventual microarray technique and the LNC-3 microarray technology for the strains *C. freundii*, *B. fragilis*, *Proteus penneri*, and *Streptococcus pseudopneumoniae*. The threshold for positive detection, being the mean value of all probes plus one standard deviation, is depicted as black dotted line for the conventional microarray-based signals and red dotted line for the LNC-3-based ones.

## Sensitivity test of the LNC-3 microarray technology


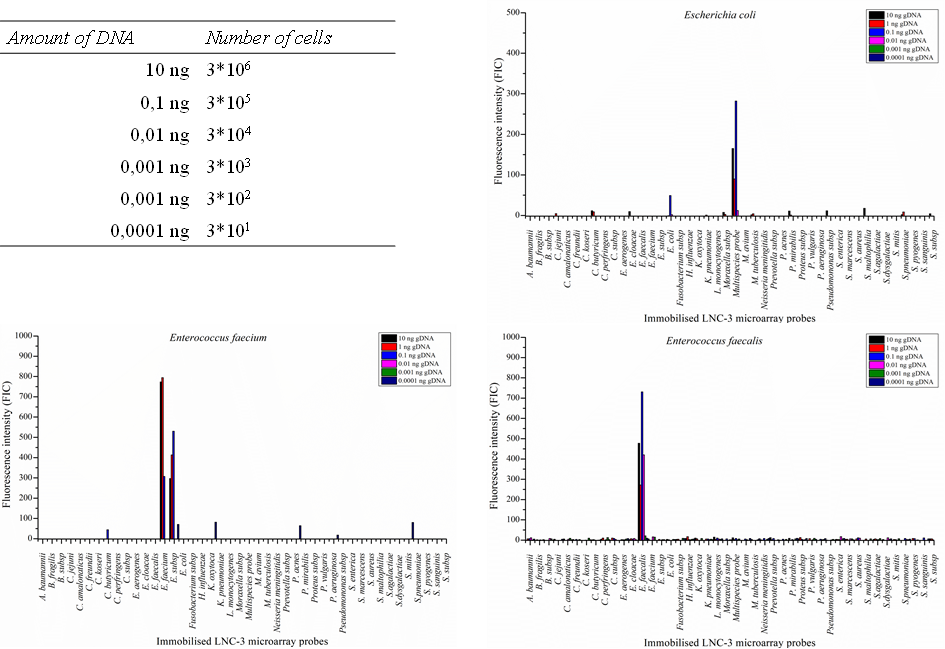


Supplementary Figure S8: Different amounts of target DNA were applied to the LNC-3-functionalized microarrays. 10 ng DNA correspond to 10^6^ bacterial cells. The analysed strains were *E. coli*, *Enterococcus faecium* and *Enterococcus faecalis*.

## Evaluation of the probe performance

Supplementary Figure S9: **Performance of the LNC-3 probes (ABR gene set 2):** On the left, a heatmap displays all measurements line by line. Each displayed value is the median value of four repetitions. On the right, all correct signals (red) were evaluated statistically against the signals of all non-matching probes (grey).

Supplementary Figure S10: **Performance of the LNC-3 probes (ABR gene set 3):** On the left, a heatmap displays all measurements line by line. Each displayed value is the median value of four repetitions. On the right, all correct signals (red) were evaluated statistically against the signals of all non-matching probes (grey).

Supplementary Figure S11: **Performance of the LNC-3 probes (ABR gene set 4):** On the left, a heatmap displays all measurements line by line. Each displayed value is the median value of four repetitions. On the right, all correct signals (red) were evaluated statistically against the signals of all non-matching probes (grey).

*
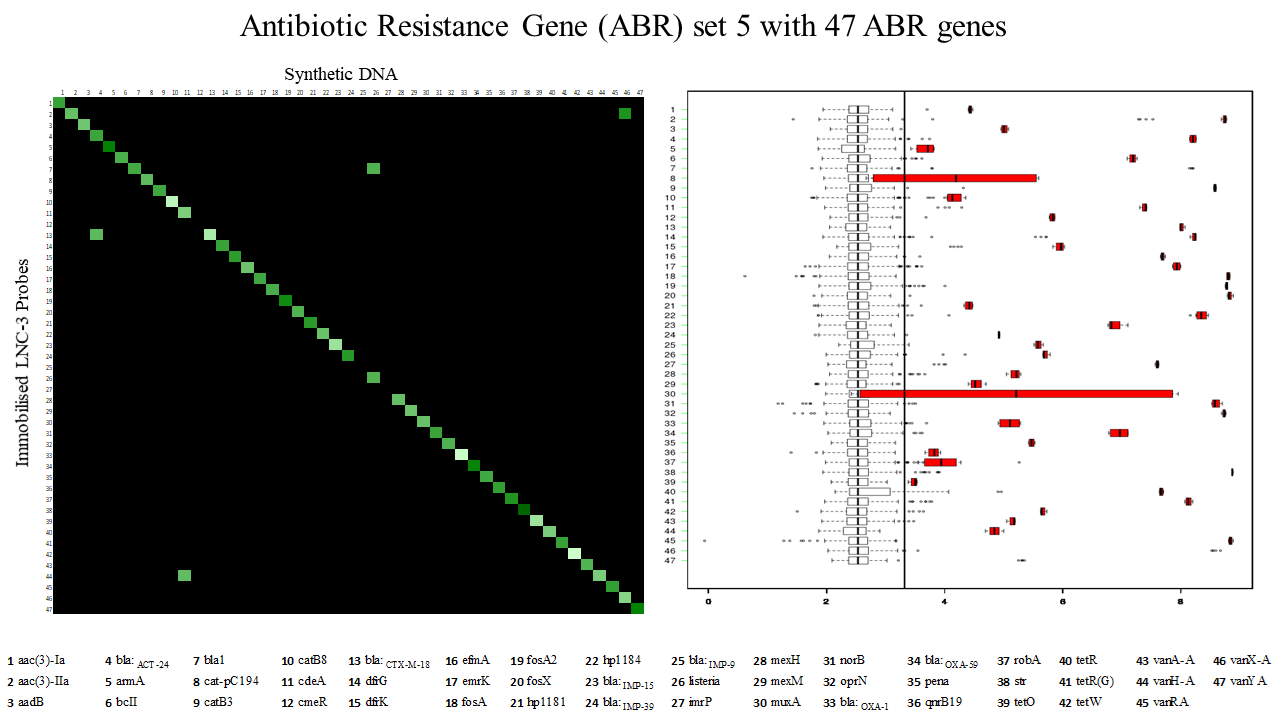
*

Supplementary Figure S12: **Performance of the LNC-3 probes (ABR gene set 5):** On the left, a heatmap displays all measurements line by line. Each displayed value is the median value of four repetitions. On the right, all correct signals (red) were evaluated statistically against the signals of all non-matching probes (grey).

Supplementary Figure S13: **Performance of the LNC-3 probes (ABR gene set 6):** On the left, a heatmap displays all measurements line by line. Each displayed value is the median value of four repetitions. On the right, all correct signals (red) were evaluated statistically against the signals of all non-matching probes (grey).

Supplementary Figure S14: **Performance of the LNC-3 probes (ABR gene set 7):** On the left, a heatmap displays all measurements line by line. Each displayed value is the median value of four repetitions. On the right, all correct signals (red) were evaluated statistically against the signals of all non-matching probes (grey).

Supplementary Figure S15: **Performance of the LNC-3 probes (ABR gene set 8):** On the left, a heatmap displays all measurements line by line. Each displayed value is the median value of four repetitions. On the right, all correct signals (red) were evaluated statistically against the signals of all non-matching probes (grey).

Supplementary Figure S16: **Performance of the LNC-3 probes (ABR gene set 9):** On the left, a heatmap displays all measurements line by line. Each displayed value is the median value of four repetitions. On the right, all correct signals (red) were evaluated statistically against the signals of all non-matching probes (grey).

Supplementary Figure S17: **Performance of the LNC-3 probes (VF gene set 2):** On the left, a heatmap displays all measurements line by line. Each displayed value is the median value of four repetitions. On the right, all correct signals (red) were evaluated statistically against the signals of all non-matching probes (grey).

Supplementary Figure S18: **Performance of the LNC-3 probes (VF gene set 3):** On the left, a heatmap displays all measurements line by line. Each displayed value is the median value of four repetitions. On the right, all correct signals (red) were evaluated statistically against the signals of all non-matching probes (grey).

Supplementary Figure S19: **Performance of the LNC-3 probes (VF gene set 4):** On the left, a heatmap displays all measurements line by line. Each displayed value is the median value of four repetitions. On the right, all correct signals (red) were evaluated statistically against the signals of all non-matching probes (grey).

Supplementary Figure S20: **Performance of the LNC-3 probes (VF gene set 5):** On the left, a heatmap displays all measurements line by line. Each displayed value is the median value of four repetitions. On the right, all correct signals (red) were evaluated statistically against the signals of all non-matching probes (grey).

Supplementary Figure S21: **Performance of the LNC-3 probes (VF gene set 6):** On the left, a heatmap displays all measurements line by line. Each displayed value is the median value of four repetitions. On the right, all correct signals (red) were evaluated statistically against the signals of all non-matching probes (grey).

*
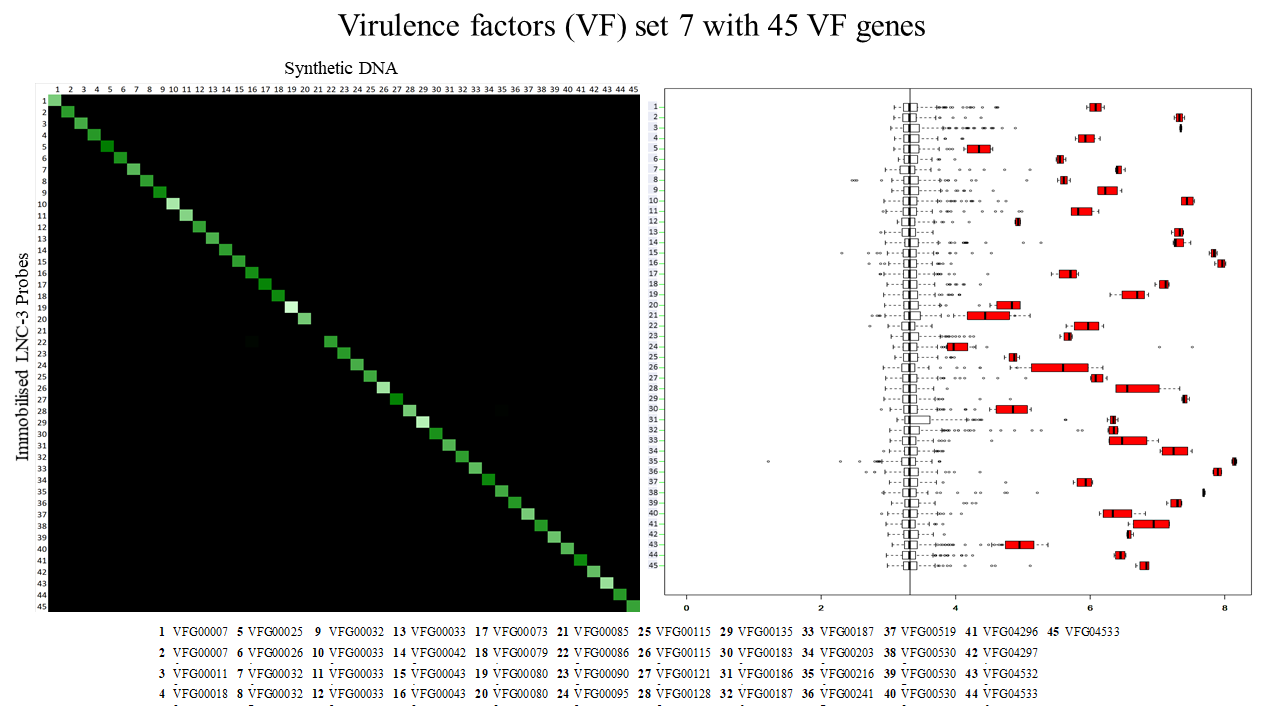
*

Supplementary Figure S22: **Performance of the LNC-3 probes (VF gene set 7):** On the left, a heatmap displays all measurements line by line. Each displayed value is the median value of four repetitions. On the right, all correct signals (red) were evaluated.

## Characterisation of bacterial isolates

Supplementary Table S2: Characterisation of bacterial isolates.

| **Pathogen / gene type** | **Phylogenetic marker genes** | **Antibiotic resistance genes** | | **Virulence factor genes** | |
| --- | --- | --- | --- | --- | --- |
|  |  | **Sequenced** | **LNC-3** | **Sequenced** | **LNC-3** |
| *Acinetobacter baumannii* | *Acinetobacter baumannii* | **aadA**, OXA-66, SulI, Mbl abeS, adeC, adeJ, adeS, adeB, adeA, adeK, adeG, ADC-2, BlaA1, adeF, adeR, adeI, adeN, Zn-dependent hydrolase, **AAC(3)-Ia**, OXA-72, BlaA2 | OXA-66, SulI, Mbl abeS, adeC, adeJ, adeS, adeB, adeA, adeK, adeG, ADC-2, BlaA1, adeF, adeR, adeI, adeN, Zn-dependent hydrolase, OXA-72, BlaA2 |  |  |
| *Bacteroides fragilis* | *Bacteroides fragilis* | tetX, ErmF, cfiA7 | tetX, ErmF, cfiA7 |  |  |
| *Escherichia coli* | *Multispecies probe, Escherichia coli* | acrB, acrD, acre, bacA, baeR, baeS, **CRP**, emrB, **emrR**, evgA, H-NS, leuO, mdtD, mdtF, **mdtL, mdtN**, msbA, Penicillin_Binding_Protein_Ecoli, PmrF, **tolC**, acrA_Escherichia, acrF, acrS, AmpC2_Ecoli, arnA, emrA, emrD, emrY, evgS, gadE, gadW, **gadX,** marA, mdfA, mdtA, **mdtB**, mdtE, mdtG, mdtH, mdtM, mdtO, mdtP, mfd, PmrC, **PmrE**, emrK | acrB, acrD, acre, bacA, baeR, baeS, , emrB, evgA, H-NS, leuO, mdtD, mdtF, msbA, Penicillin_Binding_Protein_Ecoli, PmrF, acrA_Escherichia, acrF, acrS, AmpC2_Ecoli, arnA, emrA, emrD, emrY, evgS, gadE, gadW, marA, mdfA, mdtA, mdtE, mdtG, mdtH, mdtM, mdtO, mdtP, mfd, PmrC, emrK | yagZ/ecpA, yagY/ecpB, yagX/ecpC, yagW/ecpD, yagV/ecpE, ykgK/ecpR | yagZ/ecpA, yagY/ecpB, yagX/ecpC, yagW/ecpD, yagV/ecpE, ykgK/ecpR |
| *Enterobacter aerogenes* | *Multispecies probe, Klebsiella pneumoniae, Enterobacter aerogenes* | TEM-116, Aac6-Ib, StrB, SulII, CTX-M-15, catB3, QnrB19, AAC(3)-IIa, OXA-1, DfrA14, **AmpC** | TEM-116, Aac6-Ib, StrB, SulII, CTX-M-15, catB3, QnrB19, AAC(3)-IIa, OXA-1, DfrA14 |  |  |
| *Enterobacter cloacae* | *Enterobacter cloacae* | ramA, SulII, robA | ramA, SulII, robA |  |  |
| *Enterococcus faecium* | *Enterococcus faecium, Enterococcus subspecies* | AAC(6')-Ii, msrC, efmA | AAC(6')-Ii, msrC, efmA, *DfrG, VanA-A VanH-A, vanRA, vanYA* | acm | acm |
| *Haemophilus influenzae* | *Haemophilus influenzae* | hmrM | hmrM | **hmw1B**, rfaD, licD, lic2A, **rfaE**, kdtA, **licA**, **licB**, **hmw1C** | rfaD, licD, lic2A, kdtA |
| *Klebsiella pneumoniae* | *Klebsiella pneumoniae* | oqxA, **FosA5, vgaC** acrA Klebsiella, oqxB | oqxA, acrA Klebsiella, oqxB | yagZ/ecpA, **east1_astA** | yagZ/ecpA |
| *Prevotella bivia* | *Multispecies probe, Prevotella bivia* | CfxA2 | CfxA2 |  |  |
| *Proteus mirabilis* | *Multispecies probe, Proteus mirabilis* | TEM-116, aadA, Aac6-Ib, SulI, CatA1, StrB, SulII, CMY-2, Sat-2A, MsrE, mphD, APH(3')-Ia, DfrA12**,** TetR, AAC(3)-Ia, armA, DfrA1, **TetA** | TEM-116, aadA, SulI, CatA1, StrB, SulII, CMY-2, Sat-2A, MsrE, mphD, APH(3')-Ia, DfrA12, TetR, AAC(3)-Ia, armA, DfrA1, *CatA2, CatA3, SulII* |  |  |
| *Pseudomonas aeruginosa* | *Multispecies probe, Pseudomonas aeruginosa* | **PDC-1**, amrA, amrB **Aph3-IIb**, arnA, basS, **CatB7**, CpxR, **MexA**, MexB, MexD, **MexE**, **MexF**, mexG, mexI, mexJ, mexK, mexL, mexP, mexQ, mexV, mexW, **MuxB**, **MuxC**, OpmB, **opmD**, opmE, OpmH, OprJ, OprM, OXA-50, TriB, TriC, **OprN**, mexH, FosA, MuxA, mexM, **MexC**, **TriA** | amrA, amrB, arnA, basS, CpxR, MexB, MexD, mexG, mexI, mexJ, mexK, mexL, mexP, mexQ, mexV, OpmB, opmE, OpmH, OprJ, OprM, OXA-50, TriB, TriC, mexH, FosA, MuxA, mexM, | xcpA/pilD, algB, algQ, algZ, algU, alg8, alg44, algE, algX, algL, algF, algA, mucC, waaG, waaC, aprA, lasA, lasB, rhlI, lasI, plcH, xcpZ, xcpV, **xcpT,** xcpS, xcpP, xcpQ, pilY2, **pilS,** pilR, pilP, **pilM**, pilT, pilU, pilG**, pilH**, pilI, chpB, chpC, flgD, flgG, flgH, flgI, flgJ, fleQ, fleR, **fliE, fliG**, fliI, fliJ, fliM, fliN, fliP, fliQ, flhB, **flhA** | xcpA/pilD, algB, algQ, algZ, algU, alg8, alg44, algE, algX, algL, algF, algA, mucC, waaG, waaC, aprA, lasA, lasB, rhlI, lasI, plcH, xcpZ, xcpV, xcpS, xcpP, xcpQ, pilY2, pilR, pilP, pilT, pilU, pilG, pilI, chpB, chpC, flgD, flgG, flgH, flgI, flgJ, fleQ, fleR, , fliI, fliJ, fliM, fliN, fliP, fliQ, flhB, |
| *Salmonella enterica* | *Multispecies probe, Klebsiella pneumoniae*  *Salmonella enterica* | sdiA, golS, Aac6-Iaa, mdsA, mdsC, mdsB | sdiA, golS, Aac6-Iaa, mdsA, mdsC, mdsB | fimI, fimC, fimD, **fimF**, csgB, **csgA**, csgC, **csgE**, csgF, csgG, sinH, lpfE, lpfD, lpfA, lpfC, lpfB, misL, **ratB** | fimI, fimC, fimD, csgB, csgC, csgF, csgG, sinH, lpfE, lpfD, lpfA, lpfC, lpfB, misL |
| *Staphylococcus aureus* | *Staphylococcus aureus* | Aac3-Ik, arlR, **arlS**, DHA-1, ErmA, FosB, MECA, mepA, mepR, mgrA, norA, sav1866, Spc, Tet-38, Aph3-III, qacA, mecR1 | Aac3-Ik, arlR, ErmA, FosB, MECA, mepA, mepR, mgrA, norA, sav1866, Spc, Tet-38, Aph3-III, qacA, mecR1, *ACC-1* | hlgC, hlgB, ebp, sdrC, icaD, icaC, hld, hly/hla, sspC, sspB, hysA, geh, sak, hlb, adsA, scn, sdrD, sdrE, clfA, map, sea, sspA, icaA, fnbA, icaR, icaB, clfB, aur | hlgC, hlgB, ebp, sdrC, icaD, icaC, hld, hly/hla, sspC, sspB, hysA, geh, sak, hlb, adsA, scn, sdrD, sdrE, clfA, map, sea, sspA, icaA, fnbA, icaR, icaB, clfB, aur |
| *Streptococcus pseudopneumoniae* | *Streptococcus pseudopneumoniae* | patB, RlmA(II), pmrA, tetM, MefA, mel | RlmA(II), pmrA, MefA, mel | **pce, ply**, lytB, **pavA**, **pfbA, nanA** | lytB |

## Phylogenetic marker genes

Supplementary Table S3: Primer and probe sequences of the phylogenetic marker/16S rRNA genes.

| Strain | Sequence |
| --- | --- |
| Acinetobacter baumannii | ATCCGACTTAATAAGCTTTTT |
|  | GCA GCG TGG GCT CGC GTG CCG GGG TTT TTT CTCGGGGATTTCAC |
| Bacteroides subsp. | AGCGGTGATTGCTTTTT |
|  | GCA GCG TGG GCT CGC GTG CCG GGG TTT TTT CAGCACCTTCAC |
| Bacteroides fragilis | CCGGAATCCTTTAATTTTTT |
|  | GCA GCG TGG GCT CGC GTG CCG GGG TTT TTT ATCCCCATCCTTTA |
| Citrobacter amalonaticus | GGATGTCAAGACCAGTTTTT |
|  | GCA GCG TGG GCT CGC GTG CCG GGG TTT TTT CTGCCAAGTTCTGT |
| Citrobacter freundii | GTTCCCAGGTTGAGTTTTT |
|  | GCA GCG TGG GCT CGC GTG CCG GGG TTT TTTCAGTTTCGGATGCA |
| Campylobacter jejuni | GAGTATTAGCAGTCGTTTTTTT |
|  | GCA GCG TGG GCT CGC GTG CCG GGG TTT TTT GTGTTAAGCAGGAGTATA |
| Citrobacter koseri | GCCAGTATCAGATGCTTTTT |
|  | GCA GCG TGG GCT CGC GTG CCG GGG TTT TTTCGAGACTCAAGCCT |
| Clostridium butyricum | TGCCACCGAAGTTTTTT |
|  | GCA GCG TGG GCT CGC GTG CCG GGG TTT TTTCCACCTGTCTTCC |
| Clostridium subsp. | AGGTTCTTCGCGTTTTTT |
|  | GCA GCG TGG GCT CGC GTG CCG GGG TTT TTTTGTCAAGTCTAGGTA |
| Clostridium perfringens | TAATGCAAGGGATGTTTTTT |
|  | GCA GCG TGG GCT CGC GTG CCG GGG TTT TTTTTTCCTCGATTAAGAG |
| Enterobacter cloacae | ACGTCAATTGCTGCTTTTT |
|  | GCA GCG TGG GCT CGC GTG CCG GGG TTT TTTTTCTTCTGCGGGTA |
| Escherichia coli | TTAACTTTACTCCCTTTTTT |
|  | GCA GCG TGG GCT CGC GTG CCG GGG TTT TTTAATGAGCAAAGGTA |
| Enterococcus faecium | CCACTCCTCTTTTTCTTTTT |
|  | GCA GCG TGG GCT CGC GTG CCG GGG TTT TTTTACTCACCCGTTCG |
| Enterococcus faecalis | GCGGCATAAACTGTTTTTT |
|  | GCA GCG TGG GCT CGC GTG CCG GGG TTT TTTTTCACTCTTATGCCAT |
| Enterobacter aerogenes | TCCTCGCTGAAAGTATTTTT |
|  | GCA GCG TGG GCT CGC GTG CCG GGG TTT TTTCCTTAACGCCTTCC |
| Enterococcus subsp. | CCTCGCGACTTCTTTTT |
|  | GCA GCG TGG GCT CGC GTG CCG GGG TTT TTTAAGAGATTAGCTTAG |
| Fusobacterium subsp. | AAGCTCTCTCACAGTTTTT |
|  | GCA GCG TGG GCT CGC GTG CCG GGG TTT TTTTAATGGGACGCA |
| Haemophilus influenzae | TTCCGATAATACGCGTTTTT |
|  | GCA GCG TGG GCT CGC GTG CCG GGG TTT TTTCCCGCACTTTCATC |
| Klebsiella oxytoca | CTGCCAGTTTCGATTTTT |
|  | GCA GCG TGG GCT CGC GTG CCG GGG TTT TTTACAAGACTCCAGC |
| Klebsiella pneumoniae | CTCGACTTGCATGTTTTTT |
|  | GCA GCG TGG GCT CGC GTG CCG GGG TTT TTTTCTCTGTGCTACCG |
| Listeria monocytogenes | TATCCATTGTAGCACGTTTTT |
|  | GCA GCG TGG GCT CGC GTG CCG GGG TTT TTTGCGACCCTTTGTAC |
| Mycobacterium avium | TATCCGGTATTAGACTTTTT |
|  | GCA GCG TGG GCT CGC GTG CCG GGG TTT TTTGTCTTGAGGTCC |
| Morganella morganii | TAGTTATCCCCCGCTTTTT |
|  | GCA GCG TGG GCT CGC GTG CCG GGG TTT TTTGCTACCGTTTCCAG |
| Moraxella subsp. | CGGCGTGGACTATTTTT |
|  | GCA GCG TGG GCT CGC GTG CCG GGG TTT TTTGGTAGACATCGTTTA |
| Mycobacterium tuberculosis | GACATGCATCCCGTTTTT |
|  | GCA GCG TGG GCT CGC GTG CCG GGG TTT TTTGCTTTCCACCACAA |
| Neisseria meningitidis | CTCTGACACACTCGTTTTT |
|  | GCA GCG TGG GCT CGC GTG CCG GGG TTT TTTGGAATTCTACCTCC |
| Propionibacterium acnes | AGCGGTTTACAATTTTT |
|  | GCA GCG TGG GCT CGC GTG CCG GGG TTT TTTGTCACAGGCGAA |
| Pseudomonoas subsp. | CAGCATGTCAAGGTTTTT |
|  | GCA GCG TGG GCT CGC GTG CCG GGG TTT TTTCTCTGGAAAGTTCT |
| Proteus mirabilis | CCCGCTGAAAGTACTTTTT |
|  | GCA GCG TGG GCT CGC GTG CCG GGG TTT TTTTTATCACCTTCCTC |
| Prevotella subsp. | CTTAAGCGTCAGTTTTTT |
|  | GCA GCG TGG GCT CGC GTG CCG GGG TTT TTTCGCACCTTCGAG |
| Proteus subsp. | GCAAGGTCCGAAGTTTTT |
|  | GCA GCG TGG GCT CGC GTG CCG GGG TTT TTTGTTCATCCGATAGC |
| Pseudomonas aeruginosa | TCTAGCTCAGTAGTTTTTT |
|  | GCA GCG TGG GCT CGC GTG CCG GGG TTT TTTACCCTCTACCGTAC |
| Proteus vulgaris | AGCTAATCCCATATTTTTT |
|  | GCA GCG TGG GCT CGC GTG CCG GGG TTT TTTTTACCTCACCTACT |
| Streptococcus agalactiae | AACGTTCTTCTCTAATTTTT |
|  | GCA GCG TGG GCT CGC GTG CCG GGG TTT TTTTTTCCACTCCTACC |
| Staphylococcus aureus | ACAGTTACTTACACATATGTTTTT |
|  | GCA GCG TGG GCT CGC GTG CCG GGG TTT TTTCCGTCAAGATGTGC |
| Streptococcus dysgalactiae subsp. equisimilis | CTATCTCTAGACCGTTTTT |
|  | GCA GCG TGG GCT CGC GTG CCG GGG TTT TTTCGAAGGGAAAGC |
| Serratia marcescens | GCTGAAAGTGCTTTTTTTT |
|  | GCA GCG TGG GCT CGC GTG CCG GGG TTT TTTCCACCTTCCTCCTC |
| Salmonella enterica | CCAGTTTCGAATGCTTTTT |
|  | GCA GCG TGG GCT CGC GTG CCG GGG TTT TTTAAGACTCAAGCCTG |
| Stenotrophomonas maltophilia | TGGGATTGGCTTACTTTTT |
|  | GCA GCG TGG GCT CGC GTG CCG GGG TTT TTTGACTGAGATAGGGTTTC |
| Streptococcus mitis | GGAGAAGCAAGCTCTTTTT |
|  | GCA GCG TGG GCT CGC GTG CCG GGG TTT TTTTTCGCAACTCATCC |
| Streptococcus pneumoniae | AAAGCCTACTATGGTTTTT |
|  | GCA GCG TGG GCT CGC GTG CCG GGG TTT TTTAGTTAAACAGTTTCC |
| Streptococcus pyogenes | GTACATTGGTTGAGCTTTTT |
|  | GCA GCG TGG GCT CGC GTG CCG GGG TTT TTTCCAGTTTCCAAAGC |
| Streptococcus sanguinis | AATTGCATCTTTCAATTTTT |
|  | GCA GCG TGG GCT CGC GTG CCG GGG TTT TTTATCTGGTAGTGATGC |
| Streptococcus subsp. | CACTCTCCCCTTCTTTTT |
|  | GCA GCG TGG GCT CGC GTG CCG GGG TTT TTTTACACATGGAATTC |

Antibiotic resistance genes (ABR)

### ABR-1

Supplementary Table S4: Primer and probe sequences and additional information of ABR-1

| **Gene** | **Primer/Probe** | **Hybridisation Sequence** | **Sequence** | **Spacer** | **Length** | **Product Size** | **Tm** | **Hairpin** | **Hairpin Tm** | **Hairpin delta G** |
| --- | --- | --- | --- | --- | --- | --- | --- | --- | --- | --- |
| CRP | CRP_fwd |  | GTACTACATCGTTAAAGGCTCTG |  | 23 | 151 | 62.88 | False | 0.00 | 0.00 |
|  | CRP_rev |  | TTTCGCACGTACCCATG |  | 17 | 151 | 60.62 | False | 0.00 | 0.00 |
|  | CRP_lnc3 | GCAGCGTGGGCTCGCGTGCCGGGGTTTTTT | TCCTCTCCTATCTGAA |  | 16 | 151 | 52.97 | False | 0.00 | 0.00 |
|  | CRP_detection |  | TCAGGGTGATTTTATT | TTT | 16 | 151 | 50.09 | False | 0.00 | 0.00 |
| acrE | acrE_fwd |  | GCAGCTCGATCCTATCTA |  | 18 | 126 | 58.49 | False | 0.00 | 0.00 |
|  | acrE_rev |  | TCCATGACCAACTCTACGT |  | 19 | 126 | 61.49 | False | 0.00 | 0.00 |
|  | acrE_detection |  | CGATTTTATGAGGCTG | TTT | 16 | 126 | 53.35 | False | 0.00 | 0.00 |
|  | acrE_lnc3 | GCAGCGTGGGCTCGCGTGCCGGGGTTTTTT | ACCCAATCCAGCAA |  | 14 | 126 | 54.80 | False | 0.00 | 0.00 |
| acrB | acrB_fwd |  | CCGGTCTGGGGATCAAG |  | 17 | 150 | 62.16 | False | 0.00 | 0.00 |
|  | acrB_rev |  | TAGAGATTTTCACGAACG |  | 18 | 150 | 55.21 | False | 0.00 | 0.00 |
|  | acrB_detection |  | TGGCGAAGATGGAA | TTT | 14 | 150 | 54.60 | False | 0.00 | 0.00 |
|  | acrB_lnc3 | GCAGCGTGGGCTCGCGTGCCGGGGTTTTTT | AATCCGTGCTGAAC |  | 14 | 150 | 53.54 | False | 0.00 | 0.00 |
| MECA | MECA_fwd |  | GAAAAAAGATGGCAAAGATA |  | 20 | 146 | 55.54 | False | 0.00 | 0.00 |
|  | MECA_rev |  | ATGAAGGTGTGCTTACAAGT |  | 20 | 146 | 61.04 | False | 0.00 | 0.00 |
|  | MECA_lnc3 | GCAGCGTGGGCTCGCGTGCCGGGGTTTTTT | ATTTATAACAACATGAAA |  | 18 | 146 | 48.36 | False | 0.00 | 0.00 |
|  | MECA_detection |  | AATGATTATGGCTCA | TTT | 15 | 146 | 48.94 | False | 0.00 | 0.00 |
| mdtN | mdtN_fwd |  | TGGAAAGTACGCCGAAA |  | 17 | 118 | 59.35 | False | 0.00 | 0.00 |
|  | mdtN_rev |  | TTAGTTGATGGCGCACTGT |  | 19 | 118 | 63.46 | False | 0.00 | 0.00 |
|  | mdtN_detection |  | CTGCTCTGTTAGTGG | TTT | 15 | 118 | 53.93 | False | 0.00 | 0.00 |
|  | mdtN_lnc3 | GCAGCGTGGGCTCGCGTGCCGGGGTTTTTT | CTCGCAGTAAATTCC |  | 15 | 118 | 52.13 | False | 0.00 | 0.00 |
| msbA | msbA_fwd |  | ATGAGTTGCAGAAAAACCG |  | 19 | 103 | 60.17 | False | 0.00 | 0.00 |
|  | msbA_rev |  | TCCACAATGACACCATC |  | 17 | 103 | 56.86 | False | 35.35 | 113.51 |
|  | msbA_lnc3 | GCAGCGTGGGCTCGCGTGCCGGGGTTTTTT | CTCTGGTGATTGCC |  | 14 | 103 | 53.61 | False | 0.00 | 0.00 |
|  | msbA_detection |  | CACCGCTTGTCTAC | TTT | 14 | 103 | 53.77 | False | 0.00 | 0.00 |
| Penicillin_Binding_Protein_Ecoli | Penicillin_Binding_Protein_Ecoli_fwd |  | GAAAACTAACCTGACCGAAGTACA |  | 24 | 147 | 64.92 | False | 0.00 | 0.00 |
|  | Penicillin_Binding_Protein_Ecoli_rev |  | TTGATTTTCGACACATAGCC |  | 20 | 147 | 60.01 | False | 0.00 | 0.00 |
|  | Penicillin_Binding_Protein_Ecoli_detection |  | CGTCGTTACTATCCTT | TTT | 16 | 147 | 53.55 | False | 0.00 | 0.00 |
|  | Penicillin_Binding_Protein_Ecoli_lnc3 | GCAGCGTGGGCTCGCGTGCCGGGGTTTTTT | GAAGTTAAAGGCTATAAA |  | 18 | 147 | 50.70 | False | 0.00 | 0.00 |
| RlmA(II) | RlmA(II)_fwd |  | AAGGAAAATTTTCAAAACCGT |  | 21 | 130 | 59.09 | False | 42.43 | -429.92 |
|  | RlmA(II)_rev |  | CTTCACCACAACCGATATCC |  | 20 | 130 | 61.96 | False | 0.00 | 0.00 |
|  | RlmA(II)_detection |  | CTGACTTGCTTGCA | TTT | 14 | 130 | 53.84 | False | 0.00 | 0.00 |
|  | RlmA(II)_lnc3 | GCAGCGTGGGCTCGCGTGCCGGGGTTTTTT | TATCTTAGATGCTGTAT |  | 17 | 130 | 49.45 | False | 0.00 | 0.00 |
| evgA | evgA_fwd |  | AATAATTATTGATGACCATCC |  | 21 | 120 | 55.03 | False | 40.55 | -278.04 |
|  | evgA_rev |  | GTTTCCACCCGCTGAAC |  | 17 | 120 | 61.61 | False | 0.00 | 0.00 |
|  | evgA_lnc3 | GCAGCGTGGGCTCGCGTGCCGGGGTTTTTT | TATCGCAGCAATTC |  | 14 | 120 | 50.32 | False | 0.00 | 0.00 |
|  | evgA_detection |  | GTAATTTATTGATCAAAA | TTT | 18 | 120 | 46.91 | False | 0.00 | 0.00 |
| mtrA | mtrA_fwd |  | GTAGAAATCGACGTACCG |  | 18 | 126 | 58.18 | False | 0.00 | 0.00 |
|  | mtrA_rev |  | CACATCACGAGTAAACACC |  | 19 | 126 | 59.65 | False | 0.00 | 0.00 |
|  | mtrA_detection |  | CACCGCTGGAATTC | TTT | 14 | 126 | 54.63 | False | 0.00 | 0.00 |
|  | mtrA_lnc3 | GCAGCGTGGGCTCGCGTGCCGGGGTTTTTT | AGCAGATCTCGTTGA |  | 15 | 126 | 54.97 | False | 0.00 | 0.00 |
| tetM | tetM_fwd |  | GTACAAGCACAAACTCG |  | 17 | 113 | 56.35 | False | 0.00 | 0.00 |
|  | tetM_rev |  | TGATAAACCGTTGATAAATCA |  | 21 | 113 | 56.98 | False | 36.60 | 25.54 |
|  | tetM_lnc3 | GCAGCGTGGGCTCGCGTGCCGGGGTTTTTT | CAATCTTTTTTATCAAT |  | 17 | 113 | 45.80 | False | 0.00 | 0.00 |
|  | tetM_detection |  | AAGATTGACCAAAAT | TTT | 15 | 113 | 46.95 | False | 0.00 | 0.00 |
| emrB | emrB_fwd |  | GTATCAGCCTCGCGTAT |  | 17 | 146 | 59.11 | False | 0.00 | 0.00 |
|  | emrB_rev |  | GATAATCGGCGACAGGAT |  | 18 | 146 | 59.86 | False | 0.00 | 0.00 |
|  | emrB_detection |  | GGTCTACGGTTACA | TTT | 14 | 146 | 50.76 | False | 0.00 | 0.00 |
|  | emrB_lnc3 | GCAGCGTGGGCTCGCGTGCCGGGGTTTTTT | CAGTTGTTGCAGGA |  | 14 | 146 | 53.26 | False | 0.00 | 0.00 |
| mepR | mepR_fwd |  | ATACGTTAGGTTATCTTTATGCAC |  | 24 | 134 | 61.03 | False | 0.00 | 0.00 |
|  | mepR_rev |  | GCGATAGATCAGCTTTTTACGTTC |  | 24 | 134 | 64.61 | False | 39.76 | -84.60 |
|  | mepR_lnc3 | GCAGCGTGGGCTCGCGTGCCGGGGTTTTTT | CTGACACAAAATGATATTG |  | 19 | 134 | 54.04 | False | 0.00 | 0.00 |
|  | mepR_detection |  | CTAAAGCATTACAACGAA | TTT | 18 | 134 | 54.70 | False | 0.00 | 0.00 |
| bacA | bacA_fwd |  | CTGATAGCGGCAATATTGG |  | 19 | 83 | 59.64 | False | 0.00 | 0.00 |
|  | bacA_rev |  | CCGACAATAATCATATGGCC |  | 20 | 83 | 60.01 | False | 0.00 | 0.00 |
|  | bacA_detection |  | AATTTCTGCCGGTAT | TTT | 15 | 83 | 52.81 | False | 0.00 | 0.00 |
|  | bacA_lnc3 | GCAGCGTGGGCTCGCGTGCCGGGGTTTTTT | TCGAAGGATTGACAG |  | 15 | 83 | 53.00 | False | 0.00 | 0.00 |
| mdtD | mdtD_fwd |  | TGACAGAWCTTCCCGAC |  | 17 | 94 | 58.71 | False | 0.00 | 0.00 |
|  | mdtD_rev |  | GCGGTGTTDACGATGGT |  | 17 | 94 | 61.56 | False | 0.00 | 0.00 |
|  | mdtD_detection |  | GGCTTCTTTATGCAG | TTT | 15 | 94 | 52.42 | False | 0.00 | 0.00 |
|  | mdtD_lnc3 | GCAGCGTGGGCTCGCGTGCCGGGGTTTTTT | GGATTGTGGCTTTC |  | 14 | 94 | 51.39 | False | 0.00 | 0.00 |
| sav1866 | sav1866_fwd |  | GGGTTAATGAATATTTGGTTAG |  | 22 | 126 | 56.72 | False | 0.00 | 0.00 |
|  | sav1866_rev |  | GTACACCGTTAAAATGTAAAA |  | 21 | 126 | 56.35 | False | 35.80 | 99.03 |
|  | sav1866_lnc3 | GCAGCGTGGGCTCGCGTGCCGGGGTTTTTT | AATTATTATTGCACTATCC |  | 19 | 126 | 51.99 | False | 0.00 | 0.00 |
|  | sav1866_detection |  | ATAATGTTCTTTTTAGATGT | TTT | 20 | 126 | 52.29 | False | 0.00 | 0.00 |
| baeS | baeS_fwd |  | TTTGAACGTTTTTATCGCA |  | 19 | 163 | 57.53 | False | 0.00 | 0.00 |
|  | baeS_rev |  | CCAGCGGTAACTCTACT |  | 17 | 163 | 58.07 | False | 0.00 | 0.00 |
|  | baeS_lnc3 | GCAGCGTGGGCTCGCGTGCCGGGGTTTTTT | CGATTTGCCTGAAC |  | 14 | 163 | 52.08 | False | 0.00 | 0.00 |
|  | baeS_detection |  | ATTGTTGAAGCACAT | TTT | 15 | 163 | 51.00 | False | 0.00 | 0.00 |
| H-NS | H-NS_fwd |  | GACGAAAACGGCGAAACTAAAA |  | 22 | 114 | 64.37 | False | 0.00 | 0.00 |
|  | H-NS_rev |  | TTATTGCTTGATCAGGAAATC |  | 21 | 114 | 57.97 | False | 42.26 | -361.68 |
|  | H-NS_detection |  | AGCAAGGTAAATCC | TTT | 14 | 114 | 48.81 | False | 0.00 | 0.00 |
|  | H-NS_lnc3 | GCAGCGTGGGCTCGCGTGCCGGGGTTTTTT | AAAAGCAATGGATG |  | 14 | 114 | 47.58 | False | 0.00 | 0.00 |
| tolC | tolC_fwd |  | TTCTTATCGGCCTGAGC |  | 17 | 146 | 59.27 | False | 38.76 | -92.31 |
|  | tolC_rev |  | TTCATTAATTTTTTCAAAGGCA |  | 22 | 146 | 57.50 | False | 0.00 | 0.00 |
|  | tolC_detection |  | CCTTAGTAACCCGGA | TTT | 15 | 146 | 54.51 | False | 0.00 | 0.00 |
|  | tolC_lnc3 | GCAGCGTGGGCTCGCGTGCCGGGGTTTTTT | TTATCAGCAAGCACG |  | 15 | 146 | 54.83 | False | 0.00 | 0.00 |
| Tet-38 | Tet-38_fwd |  | TTACGTTGCCATTCCTATTTA |  | 21 | 149 | 59.08 | False | 0.00 | 0.00 |
|  | Tet-38_rev |  | ACCAATCATATATAACCAATTAAAG |  | 25 | 149 | 57.70 | False | 0.00 | 0.00 |
|  | Tet-38_lnc3 | GCAGCGTGGGCTCGCGTGCCGGGGTTTTTT | TAAAGCTCATTTAGAT |  | 16 | 149 | 46.71 | False | 34.72 | 170.13 |
|  | Tet-38_detection |  | TTTGTTGGCTTAAT | TTT | 14 | 149 | 45.74 | False | 0.00 | 0.00 |
| PmrF | PmrF_fwd |  | AATATATCGGCAGGATCTACA |  | 21 | 92 | 59.64 | False | 40.17 | -241.76 |
|  | PmrF_rev |  | TTATTCATTTTCCTTGCTGG |  | 20 | 92 | 57.73 | False | 37.10 | -5.85 |
|  | PmrF_lnc3 | GCAGCGTGGGCTCGCGTGCCGGGGTTTTTT | CCCCGCTATTTTGT |  | 14 | 92 | 53.00 | False | 0.00 | 0.00 |
|  | PmrF_detection |  | TCAGCAAGTTATCCG | TTT | 15 | 92 | 53.51 | False | 0.00 | 0.00 |
| mdtL | mdtL_fwd |  | GAATACGCCACCATTATG |  | 18 | 162 | 56.46 | False | 0.00 | 0.00 |
|  | mdtL_rev |  | CGCATGGGAAGGTGAAAC |  | 18 | 162 | 62.32 | False | 0.00 | 0.00 |
|  | mdtL_detection |  | TAAGCCACGTACGT | TTT | 14 | 162 | 54.32 | False | 0.00 | 0.00 |
|  | mdtL_lnc3 | GCAGCGTGGGCTCGCGTGCCGGGGTTTTTT | GCGCTGGGAATTTT |  | 14 | 162 | 54.85 | False | 0.00 | 0.00 |
| FosB | FosB_fwd |  | TAGATGACAGCGAATTTAA |  | 19 | 155 | 55.00 | False | 0.00 | 0.00 |
|  | FosB_rev |  | CTCAAGTGTGCCAGTATGTAA |  | 21 | 155 | 61.74 | False | 0.00 | 0.00 |
|  | FosB_lnc3 | GCAGCGTGGGCTCGCGTGCCGGGGTTTTTT | AGAGTTAGAGATATTAGAGA |  | 20 | 155 | 53.33 | False | 0.00 | 0.00 |
|  | FosB_detection |  | TAGACAATCAATTTACTTTA | TTT | 20 | 155 | 51.63 | False | 0.00 | 0.00 |
| acrD | acrD_fwd |  | CTTCCGTCGATAAGCAG |  | 17 | 81 | 57.65 | False | 0.00 | 0.00 |
|  | acrD_rev |  | TGATATCGCGGAACTGT |  | 17 | 81 | 58.50 | False | 0.00 | 0.00 |
|  | acrD_lnc3 | GCAGCGTGGGCTCGCGTGCCGGGGTTTTTT | ACCATTAACGCCCA |  | 14 | 81 | 54.99 | False | 0.00 | 0.00 |
|  | acrD_detection |  | GTCACTGCTGCAAA | TTT | 14 | 81 | 54.27 | False | 0.00 | 0.00 |
| TEM-116 | TEM-116_fwd |  | CCGCATACACTATTCTCAG |  | 19 | 114 | 58.59 | False | 0.00 | 0.00 |
|  | TEM-116_rev |  | TCACTCATGGTTATGGCAG |  | 19 | 114 | 60.63 | False | 38.35 | -109.62 |
|  | TEM-116_lnc3 | GCAGCGTGGGCTCGCGTGCCGGGGTTTTTT | ACTCACCAGTCACA |  | 14 | 114 | 53.39 | False | 0.00 | 0.00 |
|  | TEM-116_detection |  | GAAAAGCATCTTACG | TTT | 15 | 114 | 49.75 | False | 0.00 | 0.00 |
| Aac3-Ik | Aac3-Ik_fwd |  | GAGTATTGCTCTTAGGTACGA |  | 21 | 134 | 60.42 | False | 44.25 | -619.39 |
|  | Aac3-Ik_rev |  | TAACCATACTTTTTTCATTACT |  | 22 | 134 | 55.23 | False | 0.00 | 0.00 |
|  | Aac3-Ik_detection |  | TATATGAAAATGAAGGT | TTT | 17 | 134 | 48.08 | False | 0.00 | 0.00 |
|  | Aac3-Ik_lnc3 | GCAGCGTGGGCTCGCGTGCCGGGGTTTTTT | ACAAATGATAAAATTTATA |  | 19 | 134 | 46.58 | False | 0.00 | 0.00 |
| ANT(4')-Ib | ANT(4')-Ib_fwd |  | GCGTAATATTCGTGTGCAA |  | 19 | 132 | 60.10 | False | 0.00 | 0.00 |
|  | ANT(4')-Ib_rev |  | TCAGTTAAGACCGAAGC |  | 17 | 132 | 56.57 | False | 0.00 | 0.00 |
|  | ANT(4')-Ib_lnc3 | GCAGCGTGGGCTCGCGTGCCGGGGTTTTTT | GTGCCATGTTGATTG |  | 15 | 132 | 53.61 | False | 0.00 | 0.00 |
|  | ANT(4')-Ib_detection |  | GTCTGCATCATCGC | TTT | 14 | 132 | 54.92 | False | 0.00 | 0.00 |
| cpxA | cpxA_fwd |  | GTTACCCAAGCTCGATT |  | 17 | 79 | 56.97 | False | 0.00 | 0.00 |
|  | cpxA_rev |  | ATGCTGCTCAATCATCAG |  | 18 | 79 | 58.33 | False | 0.00 | 0.00 |
|  | cpxA_detection |  | TGGATAGCGAACAG | TTT | 14 | 79 | 51.85 | False | 0.00 | 0.00 |
|  | cpxA_lnc3 | GCAGCGTGGGCTCGCGTGCCGGGGTTTTTT | GATGACCGAGCTTC |  | 14 | 79 | 53.44 | False | 0.00 | 0.00 |
| patB | patB_fwd |  | GGAACTCAAGCAAGTCTTTGAA |  | 22 | 151 | 63.25 | False | 40.34 | -231.42 |
|  | patB_rev |  | CTCTTGCAATGTTTCATCAGTC |  | 22 | 151 | 62.03 | False | 0.00 | 0.00 |
|  | patB_lnc3 | GCAGCGTGGGCTCGCGTGCCGGGGTTTTTT | ATTTGGAAAAGAGACT |  | 16 | 151 | 50.43 | False | 0.00 | 0.00 |
|  | patB_detection |  | AAAATCAAGGTCATG | TTT | 15 | 151 | 48.21 | False | 0.00 | 0.00 |
| arlS | arlS_fwd |  | AGAAGAATCGTTAAATATTTCT |  | 22 | 162 | 55.13 | False | 0.00 | 0.00 |
|  | arlS_rev |  | TGTTTTAATGAGTGTATTCG |  | 20 | 162 | 55.03 | False | 0.00 | 0.00 |
|  | arlS_detection |  | AGGAGATGTAAATGA | TTT | 15 | 162 | 47.47 | False | 0.00 | 0.00 |
|  | arlS_lnc3 | GCAGCGTGGGCTCGCGTGCCGGGGTTTTTT | ACTTGAATTGACTAA |  | 15 | 162 | 45.74 | False | 0.00 | 0.00 |
| arlR | arlR_fwd |  | ATCATTATAATTACAGCGAAAAGTG |  | 25 | 113 | 60.51 | False | 0.00 | 0.00 |
|  | arlR_rev |  | ATTCTTGCTAAAAGTTCTTCAA |  | 22 | 113 | 58.38 | False | 0.00 | 0.00 |
|  | arlR_lnc3 | GCAGCGTGGGCTCGCGTGCCGGGGTTTTTT | TGACAAAGTTGCTG |  | 14 | 113 | 50.66 | False | 0.00 | 0.00 |
|  | arlR_detection |  | GGCTTGATTACGGT | TTT | 14 | 113 | 52.64 | False | 0.00 | 0.00 |
| emrR | emrR_fwd |  | GAAAACCACAGTATTCAGCCT |  | 21 | 168 | 62.31 | False | 0.00 | 0.00 |
|  | emrR_rev |  | CTCGTGACCTTTTTCCGTTAAT |  | 22 | 168 | 63.09 | False | 0.00 | 0.00 |
|  | emrR_detection |  | GAAAAACGCGGTTG | TTT | 14 | 168 | 53.82 | False | 0.00 | 0.00 |
|  | emrR_lnc3 | GCAGCGTGGGCTCGCGTGCCGGGGTTTTTT | TTGCCGATGAACTG |  | 14 | 168 | 53.55 | False | 0.00 | 0.00 |
| pmrA | pmrA_fwd |  | AGCAAAATGACTCCCAAA |  | 18 | 119 | 57.88 | False | 0.00 | 0.00 |
|  | pmrA_rev |  | TAGCCAAATTGACCTGCTAC |  | 20 | 119 | 61.55 | False | 36.96 | 3.64 |
|  | pmrA_detection |  | TCTGGGAGGTGTTG | TTT | 14 | 119 | 54.47 | False | 0.00 | 0.00 |
|  | pmrA_lnc3 | GCAGCGTGGGCTCGCGTGCCGGGGTTTTTT | AATCAGGTATTCTTTTA |  | 17 | 119 | 48.00 | False | 0.00 | 0.00 |
| ErmA | ErmA_fwd |  | CAAGACAACGTAATAGAAATC |  | 21 | 127 | 56.33 | False | 0.00 | 0.00 |
|  | ErmA_rev |  | TTACCGCTTCTTTAGTCA |  | 18 | 127 | 56.28 | False | 0.00 | 0.00 |
|  | ErmA_lnc3 | GCAGCGTGGGCTCGCGTGCCGGGGTTTTTT | TTTTACCAAAGAGCT |  | 15 | 127 | 49.51 | False | 0.00 | 0.00 |
|  | ErmA_detection |  | AGTCAAAATGAGTCG | TTT | 15 | 127 | 50.95 | False | 0.00 | 0.00 |
| efpA | efpA_fwd |  | TACGGCTCATTTTTCATG |  | 18 | 117 | 56.19 | False | 0.00 | 0.00 |
|  | efpA_rev |  | GATCGCCGACAGAGTCAG |  | 18 | 117 | 63.14 | False | 35.83 | 85.13 |
|  | efpA_lnc3 | GCAGCGTGGGCTCGCGTGCCGGGGTTTTTT | TGTGCCCTACTTCC |  | 14 | 117 | 54.59 | False | 0.00 | 0.00 |
|  | efpA_detection |  | CCAACCTGGTCATG | TTT | 14 | 117 | 53.46 | False | 34.69 | 140.44 |
| mepA | mepA_fwd |  | TTAATGAGTATCTTAATGGGATT |  | 23 | 122 | 57.02 | False | 38.06 | -46.44 |
|  | mepA_rev |  | ATAAGTTCTGGAAATTGCACAAG |  | 23 | 122 | 61.91 | False | 35.26 | 156.93 |
|  | mepA_lnc3 | GCAGCGTGGGCTCGCGTGCCGGGGTTTTTT | ACATTATGGAAACTTCG |  | 17 | 122 | 53.33 | False | 0.00 | 0.00 |
|  | mepA_detection |  | CGATTGCAAGTTATGG | TTT | 16 | 122 | 54.09 | False | 0.00 | 0.00 |
| norA | norA_fwd |  | GTATGAGTGCTGGTATGGT |  | 19 | 156 | 60.01 | False | 0.00 | 0.00 |
|  | norA_rev |  | AAACTTCTGCCATAAATCCAC |  | 21 | 156 | 60.56 | False | 0.00 | 0.00 |
|  | norA_lnc3 | GCAGCGTGGGCTCGCGTGCCGGGGTTTTTT | CAAGCCATCAAAAAG |  | 15 | 156 | 50.62 | False | 0.00 | 0.00 |
|  | norA_detection |  | CAAAAAACTTTGGCT | TTT | 15 | 156 | 50.19 | False | 0.00 | 0.00 |
| sdiA | sdiA_fwd |  | AATGATTTCTTCACCTGG |  | 18 | 96 | 55.27 | False | 41.77 | -283.29 |
|  | sdiA_rev |  | CTGTGTCTGATATTGCAATT |  | 20 | 96 | 57.53 | False | 0.00 | 0.00 |
|  | sdiA_lnc3 | GCAGCGTGGGCTCGCGTGCCGGGGTTTTTT | CGCAATGTTGTTAC |  | 14 | 96 | 49.59 | False | 0.00 | 0.00 |
|  | sdiA_detection |  | GCTTTCAGGAGATG | TTT | 14 | 96 | 50.15 | False | 0.00 | 0.00 |
| mgrA | mgrA_fwd |  | CGATCAACGTGAAGTATTTATT |  | 22 | 146 | 58.76 | False | 39.10 | -154.35 |
|  | mgrA_rev |  | TGACTTTACCTAATAAGCGATTAAG |  | 25 | 146 | 61.22 | False | 34.64 | 181.55 |
|  | mgrA_lnc3 | GCAGCGTGGGCTCGCGTGCCGGGGTTTTTT | AACTATTAGACCAGA |  | 15 | 146 | 46.54 | False | 0.00 | 0.00 |
|  | mgrA_detection |  | ATTAAGTAATGCATCT | TTT | 16 | 146 | 47.18 | False | 0.00 | 0.00 |
| Dha1 | Dha1_fwd |  | ACGCTGGTATTGGTGTT |  | 17 | 115 | 59.34 | False | 0.00 | 0.00 |
|  | Dha1_rev |  | AAAACAACGATTAATCCTAAT |  | 21 | 115 | 55.12 | False | 0.00 | 0.00 |
|  | Dha1_lnc3 | GCAGCGTGGGCTCGCGTGCCGGGGTTTTTT | GGTCATGACACATG |  | 14 | 115 | 50.61 | False | 0.00 | 0.00 |
|  | Dha1_detection |  | TTTCTGTTCAAGCT | TTT | 14 | 115 | 48.49 | False | 0.00 | 0.00 |
| baeR | baeR_fwd |  | AGTTACCAATCGACGAAAACAC |  | 22 | 87 | 63.33 | False | 0.00 | 0.00 |
|  | baeR_rev |  | CACGCAGATAATCAATGAG |  | 19 | 87 | 57.33 | False | 0.00 | 0.00 |
|  | baeR_detection |  | ATGAACCGAAGCTG | TTT | 14 | 87 | 53.10 | False | 0.00 | 0.00 |
|  | baeR_lnc3 | GCAGCGTGGGCTCGCGTGCCGGGGTTTTTT | TTTTGATCGTGGAAG |  | 15 | 87 | 51.30 | False | 0.00 | 0.00 |
| mdtF | mdtF_fwd |  | CTGCAAACGCCGGAAGA |  | 17 | 113 | 63.77 | False | 0.00 | 0.00 |
|  | mdtF_rev |  | GTGGAATAATCTTCCGC |  | 17 | 113 | 55.43 | False | 45.00 | -628.41 |
|  | mdtF_lnc3 | GCAGCGTGGGCTCGCGTGCCGGGGTTTTTT | CAAAATCCTGTTGAAA |  | 16 | 113 | 50.34 | False | 0.00 | 0.00 |
|  | mdtF_detection |  | GTTCAGCAAGATGG | TTT | 14 | 113 | 51.02 | False | 0.00 | 0.00 |
| mecI | mecI_fwd |  | TAGAAGAAATACAAATGCAAA |  | 21 | 104 | 55.34 | False | 0.00 | 0.00 |
|  | mecI_rev |  | TTTATTGTCTTTTTTACGATCT |  | 22 | 104 | 55.90 | False | 0.00 | 0.00 |
|  | mecI_lnc3 | GCAGCGTGGGCTCGCGTGCCGGGGTTTTTT | CAAAAACCATTCGTA |  | 15 | 104 | 48.83 | False | 0.00 | 0.00 |
|  | mecI_detection |  | CACTTATAACGAGAT | TTT | 15 | 104 | 46.11 | False | 0.00 | 0.00 |
| Spc | Spc_fwd |  | TTGGTTCAGCAGTAAATGG |  | 19 | 141 | 59.17 | False | 0.00 | 0.00 |
|  | Spc_rev |  | CCGTATTTCCAATCTTTCCTGAT |  | 23 | 141 | 62.88 | False | 0.00 | 0.00 |
|  | Spc_lnc3 | GCAGCGTGGGCTCGCGTGCCGGGGTTTTTT | CATAGTTTACCTCAATTAAC |  | 20 | 141 | 53.60 | False | 0.00 | 0.00 |
|  | Spc_detection |  | TCGAAAAAAACTAACAGAA | TTT | 19 | 141 | 54.87 | False | 0.00 | 0.00 |
| leuO | leuO_fwd |  | ACCTGCGGTCAGTAACG |  | 17 | 110 | 62.37 | False | 42.28 | -405.29 |
|  | leuO_rev |  | CAAAAAGTTGAAATGCGC |  | 18 | 110 | 57.25 | False | 41.08 | -265.06 |
|  | leuO_detection |  | TTTTGTTCGTTATGG | TTT | 15 | 110 | 48.83 | False | 0.00 | 0.00 |
|  | leuO_lnc3 | GCAGCGTGGGCTCGCGTGCCGGGGTTTTTT | TTTAATGACGAGCT |  | 14 | 110 | 47.97 | False | 0.00 | 0.00 |

### ABR-2

Supplementary Table S5: Primer and probe sequences and additional information of ABR-2.

| Gene | Primer/Probe | Hybridisation Sequence | Sequence | Spacer | Length | Product Size | Tm | Hairpin | Hairpin Tm | Hairpin delta G |
| --- | --- | --- | --- | --- | --- | --- | --- | --- | --- | --- |
| mdtP | mdtP_fwd |  | CGCAGCTTTATTACAGTAT |  | 19 | 114 | 56.22 | False | 0.00 | 0.00 |
|  | mdtP_rev |  | GACCGTGCGCCACTTTA |  | 17 | 114 | 63.43 | False | 35.36 | 110.32 |
|  | mdtP_detection |  | TCGATCTGTTAGAAC | TTT | 15 | 114 | 49.38 | False | 0.00 | 0.00 |
|  | mdtP_lnc3 | GCAGCGTGGGCTCGCGTGCCGGGGTTTTTT | CAGCTATCAGATGC |  | 14 | 114 | 50.02 | False | 0.00 | 0.00 |
| emrD | emrD_fwd |  | TCCAGCTATTGCCGATAT |  | 18 | 111 | 58.70 | False | 43.18 | -363.18 |
|  | emrD_rev |  | GGGCCATAAAACAGCTG |  | 17 | 111 | 58.85 | False | 0.00 | 0.00 |
|  | emrD_detection |  | CGCTTATCTGCTGA | TTT | 14 | 111 | 52.44 | False | 0.00 | 0.00 |
|  | emrD_lnc3 | GCAGCGTGGGCTCGCGTGCCGGGGTTTTTT | CAGAGCGTAATGGG |  | 14 | 111 | 53.38 | False | 0.00 | 0.00 |
| mdsA | mdsA_fwd |  | TTCCAGATCGATCCGCG |  | 17 | 109 | 62.27 | False | 42.69 | -340.62 |
|  | mdsA_rev |  | GAATGCGATCGAAATCC |  | 17 | 109 | 56.34 | False | 0.00 | 0.00 |
|  | mdsA_lnc3 | GCAGCGTGGGCTCGCGTGCCGGGGTTTTTT | CGCAATTACGTCAG |  | 14 | 109 | 51.94 | False | 0.00 | 0.00 |
|  | mdsA_detection |  | GCTGAAGTACTGGC | TTT | 14 | 109 | 53.69 | False | 0.00 | 0.00 |
| Aac6-Iaa | Aac6-Iaa_fwd |  | GGCATCATTTRTTGCGAT |  | 18 | 122 | 58.38 | False | 44.65 | -794.02 |
|  | Aac6-Iaa_rev |  | GAACAAAAATACCTTCAAGRAA |  | 22 | 122 | 58.13 | False | 43.25 | -310.36 |
|  | Aac6-Iaa_lnc3 | GCAGCGTGGGCTCGCGTGCCGGGGTTTTTT | CGGATGCCTCAATC |  | 14 | 122 | 53.89 | False | 0.00 | 0.00 |
|  | Aac6-Iaa_detection |  | CGCCACGATTATGT | TTT | 14 | 122 | 53.41 | False | 0.00 | 0.00 |
| CatA1 | CatA1_fwd |  | CAATCCCTGGGTGAGTTTC |  | 19 | 114 | 61.55 | False | 0.00 | 0.00 |
|  | CatA1_rev |  | ATCAGCACCTTGTCGCC |  | 17 | 114 | 63.15 | False | 0.00 | 0.00 |
|  | CatA1_detection |  | TATGGACAACTTCTTC | TTT | 16 | 114 | 50.83 | False | 0.00 | 0.00 |
|  | CatA1_lnc3 | GCAGCGTGGGCTCGCGTGCCGGGGTTTTTT | TTTAAACGTGGCCAA |  | 15 | 114 | 54.29 | False | 0.00 | 0.00 |
| MphC | MphC_fwd |  | ACGAATGAAAGAGCATATAATAG |  | 23 | 127 | 58.40 | False | 0.00 | 0.00 |
|  | MphC_rev |  | CTACTCTTTCATACCTAACTC |  | 21 | 127 | 56.24 | False | 0.00 | 0.00 |
|  | MphC_lnc3 | GCAGCGTGGGCTCGCGTGCCGGGGTTTTTT | TATCGCTGAGTTTG |  | 14 | 127 | 49.26 | False | 0.00 | 0.00 |
|  | MphC_detection |  | CTATGGAATCAGGAG | TTT | 15 | 127 | 49.40 | False | 0.00 | 0.00 |
| gadW | gadW_fwd |  | AAAAATTTGGCAGACGATTTT |  | 21 | 157 | 59.51 | False | 38.23 | -126.63 |
|  | gadW_rev |  | GGGGKGACTGGTTAATTAA |  | 19 | 157 | 58.84 | False | 0.00 | 0.00 |
|  | gadW_detection |  | ACTTACAAAATATTCGT | TTT | 17 | 157 | 49.41 | False | 0.00 | 0.00 |
|  | gadW_lnc3 | GCAGCGTGGGCTCGCGTGCCGGGGTTTTTT | GACAGTTTGCCATT |  | 14 | 157 | 50.66 | False | 0.00 | 0.00 |
| acrF | acrF_fwd |  | AGAGGGTAAAGGTGTTGT |  | 18 | 120 | 58.52 | False | 0.00 | 0.00 |
|  | acrF_rev |  | GCMCCGTTACTGATAGC |  | 17 | 120 | 59.04 | False | 0.00 | 0.00 |
|  | acrF_lnc3 | GCAGCGTGGGCTCGCGTGCCGGGGTTTTTT | CCTCTCTCGCCTTT |  | 14 | 120 | 54.77 | False | 0.00 | 0.00 |
|  | acrF_detection |  | ATTCTCGGCGTATT | TTT | 14 | 120 | 51.35 | False | 0.00 | 0.00 |
| golS | golS_fwd |  | ATGAACATCGGTAAAGCAGC |  | 20 | 131 | 62.53 | False | 0.00 | 0.00 |
|  | golS_rev |  | ACATCAGCCTGGGTATAG |  | 18 | 131 | 58.62 | False | 0.00 | 0.00 |
|  | golS_detection |  | GAACAGATTGGTCT | TTT | 14 | 131 | 48.35 | False | 34.40 | 167.24 |
|  | golS_lnc3 | GCAGCGTGGGCTCGCGTGCCGGGGTTTTTT | TGATTCGCTACTAT |  | 14 | 131 | 46.29 | False | 0.00 | 0.00 |
| PmrE | PmrE_fwd |  | TTCTAATCGCWCAAAATCA |  | 19 | 80 | 55.94 | False | 0.00 | 0.00 |
|  | PmrE_rev |  | AGATATCCGATCATTCAGCATAG |  | 23 | 80 | 61.79 | False | 0.00 | 0.00 |
|  | PmrE_lnc3 | GCAGCGTGGGCTCGCGTGCCGGGGTTTTTT | TTGTGGCATTAGAT |  | 14 | 80 | 47.70 | False | 0.00 | 0.00 |
|  | PmrE_detection |  | ATTTTACCGTCACG | TTT | 14 | 80 | 50.16 | False | 0.00 | 0.00 |
| SulII | SulII_fwd |  | TATTCGCGGTTTTCCAGAC |  | 19 | 88 | 61.60 | False | 43.09 | -356.22 |
|  | SulII_rev |  | CCCGTCTTGCACCGAATG |  | 18 | 88 | 64.71 | False | 0.00 | 0.00 |
|  | SulII_detection |  | CGAAATCATCTGCC | TTT | 14 | 88 | 51.33 | False | 0.00 | 0.00 |
|  | SulII_lnc3 | GCAGCGTGGGCTCGCGTGCCGGGGTTTTTT | CTATCCGCAATTGG |  | 14 | 88 | 50.83 | False | 0.00 | 0.00 |
| Aph3-III | Aph3-III_fwd |  | ATCACCGGAATTGAAAAAACTGA |  | 23 | 116 | 63.46 | False | 0.00 | 0.00 |
|  | Aph3-III_rev |  | TTTTTAAATATAGGTTTTCATTTTC |  | 25 | 116 | 55.48 | False | 0.00 | 0.00 |
|  | Aph3-III_detection |  | CTGCTAAGGTATATAA | TTT | 16 | 116 | 46.26 | False | 0.00 | 0.00 |
|  | Aph3-III_lnc3 | GCAGCGTGGGCTCGCGTGCCGGGGTTTTTT | GGAAGGAATGTCTC |  | 14 | 116 | 49.11 | False | 0.00 | 0.00 |
| AmpC1_Ecoli | AmpC1_Ecoli_fwd |  | TGGAGATGATCAAGCGCAC |  | 19 | 141 | 63.94 | False | 0.00 | 0.00 |
|  | AmpC1_Ecoli_rev |  | AAATCGACTCTTCAACATAG |  | 20 | 141 | 56.60 | False | 0.00 | 0.00 |
|  | AmpC1_Ecoli_detection |  | ATTTATCGTCGAGTCG | TTT | 16 | 141 | 54.56 | False | 0.00 | 0.00 |
|  | AmpC1_Ecoli_lnc3 | GCAGCGTGGGCTCGCGTGCCGGGGTTTTTT | GATTATATGCTGCTTGG |  | 17 | 141 | 54.05 | False | 0.00 | 0.00 |
| evgS | evgS_fwd |  | GTGACCATTGACACATG |  | 17 | 163 | 55.93 | False | 41.27 | -431.43 |
|  | evgS_rev |  | GGAATGATATTTGGTTTTCTAA |  | 22 | 163 | 56.28 | False | 0.00 | 0.00 |
|  | evgS_lnc3 | GCAGCGTGGGCTCGCGTGCCGGGGTTTTTT | TATTAGTTGGCAGTAG |  | 16 | 163 | 50.16 | False | 0.00 | 0.00 |
|  | evgS_detection |  | CCTTTTATGGGGATT | TTT | 15 | 163 | 49.69 | False | 0.00 | 0.00 |
| marA | marA_fwd |  | TTTAAAAAAGAAACCGGTCATTC |  | 23 | 150 | 60.44 | False | 0.00 | 0.00 |
|  | marA_rev |  | GGTTCGGGTCAGAGTTT |  | 17 | 150 | 59.35 | False | 0.00 | 0.00 |
|  | marA_lnc3 | GCAGCGTGGGCTCGCGTGCCGGGGTTTTTT | AGCTGAAGGAAAGTA |  | 15 | 150 | 51.26 | False | 0.00 | 0.00 |
|  | marA_detection |  | ACGAGCCGATACTC | TTT | 14 | 150 | 54.69 | False | 0.00 | 0.00 |
| arnA | arnA_fwd |  | AAGTACCGTAAGCGAATCATC |  | 21 | 102 | 61.92 | False | 43.72 | -394.19 |
|  | arnA_rev |  | CGGGCCGACGATTAAATTAG |  | 20 | 102 | 62.70 | False | 37.95 | -52.56 |
|  | arnA_detection |  | AATACTTCGATGAGGA | TTT | 16 | 102 | 52.01 | False | 0.00 | 0.00 |
|  | arnA_lnc3 | GCAGCGTGGGCTCGCGTGCCGGGGTTTTTT | GGATGTGTAGCGATA |  | 15 | 102 | 52.43 | False | 0.00 | 0.00 |
| SulI | SulI_fwd |  | GCCGATGAGATCAGACGTA |  | 19 | 143 | 62.43 | False | 0.00 | 0.00 |
|  | SulI_rev |  | AATCCTTGGATATCGTTCAGGT |  | 22 | 143 | 63.26 | False | 0.00 | 0.00 |
|  | SulI_detection |  | CGTGTTTCAATCGA | TTT | 14 | 143 | 50.67 | False | 0.00 | 0.00 |
|  | SulI_lnc3 | GCAGCGTGGGCTCGCGTGCCGGGGTTTTTT | CCGATCAGATGCAC |  | 14 | 143 | 53.95 | False | 0.00 | 0.00 |
| ErmB | ErmB_fwd |  | GGTAACGTCTATTGAATTAGA |  | 21 | 125 | 56.52 | False | 0.00 | 0.00 |
|  | ErmB_rev |  | ACCTCTGTTTGTTAGGGAATT |  | 21 | 125 | 61.00 | False | 38.25 | -91.57 |
|  | ErmB_detection |  | CACCAAGATATTCTACA | TTT | 17 | 125 | 51.38 | False | 0.00 | 0.00 |
|  | ErmB_lnc3 | GCAGCGTGGGCTCGCGTGCCGGGGTTTTTT | TCGTGTCACTTTAATT |  | 16 | 125 | 51.15 | False | 0.00 | 0.00 |
| Aac6-Aph2 | Aac6-Aph2_fwd |  | CAAAAGAAAAAGCAATATATAATTT |  | 25 | 127 | 55.84 | False | 0.00 | 0.00 |
|  | Aac6-Aph2_rev |  | GTTCCTTTAATTTCTTTATAACC |  | 23 | 127 | 55.72 | False | 0.00 | 0.00 |
|  | Aac6-Aph2_lnc3 | GCAGCGTGGGCTCGCGTGCCGGGGTTTTTT | ACAAATTTAGAAACTAA |  | 17 | 127 | 46.47 | False | 0.00 | 0.00 |
|  | Aac6-Aph2_detection |  | TGTAAAAATTCCTAATAT | TTT | 18 | 127 | 46.85 | False | 0.00 | 0.00 |
| Aac6-Ib | Aac6-Ib_fwd |  | CTAAATCGATCTCATATCGTCG |  | 22 | 146 | 60.20 | False | 44.63 | -749.47 |
|  | Aac6-Ib_rev |  | CCAATCGGCTCTCCATTCA |  | 19 | 146 | 63.32 | False | 0.00 | 0.00 |
|  | Aac6-Ib_detection |  | AGGAACAGTACTTGC | TTT | 15 | 146 | 53.24 | False | 0.00 | 0.00 |
|  | Aac6-Ib_lnc3 | GCAGCGTGGGCTCGCGTGCCGGGGTTTTTT | ACTTGCTGACGTAC |  | 14 | 146 | 52.68 | False | 0.00 | 0.00 |
| aadA | aadA_fwd |  | CGTTATCCRGCTAAGCG |  | 17 | 92 | 59.50 | False | 0.00 | 0.00 |
|  | aadA_rev |  | TCAATGTCGATCRTGGCTG |  | 19 | 92 | 62.65 | False | 40.53 | -262.06 |
|  | aadA_lnc3 | GCAGCGTGGGCTCGCGTGCCGGGGTTTTTT | GCAATTTGGAGAATGG |  | 16 | 92 | 54.25 | False | 0.00 | 0.00 |
|  | aadA_detection |  | CAGCGCAATGACAT | TTT | 14 | 92 | 54.62 | False | 0.00 | 0.00 |
| mdtB | mdtB_fwd |  | TKTTTATTATGCGTCCTGT |  | 19 | 111 | 56.92 | False | 0.00 | 0.00 |
|  | mdtB_rev |  | GATARTCCACTTCCGGCAG |  | 19 | 111 | 62.40 | False | 0.00 | 0.00 |
|  | mdtB_lnc3 | GCAGCGTGGGCTCGCGTGCCGGGGTTTTTT | CGATCTTACTCGCC |  | 14 | 111 | 53.21 | False | 0.00 | 0.00 |
|  | mdtB_detection |  | GGGATTATCGGTTATC | TTT | 16 | 111 | 51.38 | False | 0.00 | 0.00 |
| StrB | StrB_fwd |  | CGACTATCTGGTATGGCGC |  | 19 | 113 | 63.81 | False | 0.00 | 0.00 |
|  | StrB_rev |  | CGGCAACGATGTGAGAG |  | 17 | 113 | 61.10 | True | 76.85 | -2151.90 |
|  | StrB_lnc3 | GCAGCGTGGGCTCGCGTGCCGGGGTTTTTT | AATCTGATGTTGCTC |  | 15 | 113 | 50.94 | False | 0.00 | 0.00 |
|  | StrB_detection |  | GAATATGCCGGGGA | TTT | 14 | 113 | 54.92 | False | 0.00 | 0.00 |
| mdtA | mdtA_fwd |  | ACAARGTCAGCAAACATCT |  | 19 | 82 | 59.71 | False | 0.00 | 0.00 |
|  | mdtA_rev |  | TCGCCCGCAGAAATACCT |  | 18 | 82 | 64.79 | False | 0.00 | 0.00 |
|  | mdtA_detection |  | TCAGAAAGTGGTGATC | TTT | 16 | 82 | 53.97 | False | 0.00 | 0.00 |
|  | mdtA_lnc3 | GCAGCGTGGGCTCGCGTGCCGGGGTTTTTT | GGCATTCAGGACAG |  | 14 | 82 | 53.61 | False | 0.00 | 0.00 |
| mfd | mfd_fwd |  | GAGCGCGAACTGGAACG |  | 17 | 88 | 64.53 | False | 0.00 | 0.00 |
|  | mfd_rev |  | CGATCCCGGTTTCGATAATG |  | 20 | 88 | 62.42 | False | 41.29 | -317.88 |
|  | mfd_detection |  | TTTCAACGTGCTGG | TTT | 14 | 88 | 54.29 | False | 0.00 | 0.00 |
|  | mfd_lnc3 | GCAGCGTGGGCTCGCGTGCCGGGGTTTTTT | TTTCCATCATCAACG |  | 15 | 88 | 51.40 | False | 0.00 | 0.00 |
| mdsC | mdsC_fwd |  | CCAGCGCGTCTCTTAGC |  | 17 | 141 | 63.62 | False | 0.00 | 0.00 |
|  | mdsC_rev |  | CGATTTCAATCTGTTTTTGGACAT |  | 24 | 141 | 62.90 | False | 36.72 | 21.97 |
|  | mdsC_lnc3 | GCAGCGTGGGCTCGCGTGCCGGGGTTTTTT | GGCGTTTTCTACCG |  | 14 | 141 | 54.72 | False | 0.00 | 0.00 |
|  | mdsC_detection |  | CAAATCACCTTGCCT | TTT | 15 | 141 | 54.47 | False | 0.00 | 0.00 |
| acrS | acrS_fwd |  | CCAAAATTCCCCGCCAG |  | 17 | 100 | 61.72 | False | 0.00 | 0.00 |
|  | acrS_rev |  | CCCATCTTTTCGCGTATCAC |  | 20 | 100 | 62.92 | False | 0.00 | 0.00 |
|  | acrS_lnc3 | GCAGCGTGGGCTCGCGTGCCGGGGTTTTTT | GAAAATCTTATATCACA |  | 17 | 100 | 46.64 | False | 0.00 | 0.00 |
|  | acrS_detection |  | AATGTGAATTTAATGA | TTT | 16 | 100 | 45.47 | False | 0.00 | 0.00 |
| mdtE | mdtE_fwd |  | CGCACTCGTTACCGCTAAT |  | 19 | 119 | 63.94 | False | 0.00 | 0.00 |
|  | mdtE_rev |  | TGGCGACCTCTTCTTTCAT |  | 19 | 119 | 62.49 | False | 0.00 | 0.00 |
|  | mdtE_detection |  | GTCTGGACCCGATT | TTT | 14 | 119 | 54.74 | False | 0.00 | 0.00 |
|  | mdtE_lnc3 | GCAGCGTGGGCTCGCGTGCCGGGGTTTTTT | TGGTTACCGTACAAC |  | 15 | 119 | 53.31 | False | 0.00 | 0.00 |
| emrA | emrA_fwd |  | GAGACTCAAACCCCGCAG |  | 18 | 107 | 63.67 | False | 0.00 | 0.00 |
|  | emrA_rev |  | TAAATCCCTATCGCTACGG |  | 19 | 107 | 59.56 | False | 0.00 | 0.00 |
|  | emrA_detection |  | TCACCTTGCTCTTTAT | TTT | 16 | 107 | 53.00 | False | 0.00 | 0.00 |
|  | emrA_lnc3 | GCAGCGTGGGCTCGCGTGCCGGGGTTTTTT | TCTGCTCCTCCTTC |  | 14 | 107 | 53.75 | False | 0.00 | 0.00 |
| emrY | emrY_fwd |  | CGGTTTGCTATTACTGGCG |  | 19 | 116 | 62.69 | False | 0.00 | 0.00 |
|  | emrY_rev |  | TGTTAAGGGTAAAAAGAAACA |  | 21 | 116 | 57.23 | False | 0.00 | 0.00 |
|  | emrY_lnc3 | GCAGCGTGGGCTCGCGTGCCGGGGTTTTTT | GACATTTATGCCAA |  | 14 | 116 | 46.77 | False | 0.00 | 0.00 |
|  | emrY_detection |  | CGATTGATTTTACAG | TTT | 15 | 116 | 46.48 | False | 0.00 | 0.00 |
| gadX | gadX_fwd |  | TAYGCAAGACATAAATATATTCT |  | 23 | 95 | 56.35 | False | 43.73 | -518.45 |
|  | gadX_rev |  | YGAATTTGGCTTGCATC |  | 17 | 95 | 56.89 | False | 0.00 | 0.00 |
|  | gadX_detection |  | TATCGCTATTTTAATG | TTT | 16 | 95 | 45.86 | False | 0.00 | 0.00 |
|  | gadX_lnc3 | GCAGCGTGGGCTCGCGTGCCGGGGTTTTTT | TGGTTAATGGTGAA |  | 14 | 95 | 47.44 | False | 0.00 | 0.00 |
| mdtH | mdtH_fwd |  | GTTGATAATGTCATTAAGCATG |  | 22 | 126 | 57.70 | False | 40.79 | -246.45 |
|  | mdtH_rev |  | GCACTTAAGGTTTCACGCG |  | 19 | 126 | 63.46 | False | 0.00 | 0.00 |
|  | mdtH_detection |  | TTTGTCTGTTTTATATC | TTT | 17 | 126 | 47.31 | False | 0.00 | 0.00 |
|  | mdtH_lnc3 | GCAGCGTGGGCTCGCGTGCCGGGGTTTTTT | ACTTTTCACCCTGA |  | 14 | 126 | 50.53 | False | 0.00 | 0.00 |
| AmpC2_Ecoli | AmpC2_Ecoli_fwd |  | CGATATTGTGCATCGCA |  | 17 | 158 | 58.39 | False | 43.52 | -586.50 |
|  | AmpC2_Ecoli_rev |  | ACTCAAACAACGTTTGC |  | 17 | 158 | 56.80 | False | 0.00 | 0.00 |
|  | AmpC2_Ecoli_lnc3 | GCAGCGTGGGCTCGCGTGCCGGGGTTTTTT | CGGTAATTTATCAGGGT |  | 17 | 158 | 54.78 | False | 0.00 | 0.00 |
|  | AmpC2_Ecoli_detection |  | AAACCTTATTACTTTACCT | TTT | 19 | 158 | 52.77 | False | 0.00 | 0.00 |
| OXA-66 | OXA-66_fwd |  | GGTACCCAAGTCGATAATTT |  | 20 | 160 | 58.76 | False | 0.00 | 0.00 |
|  | OXA-66_rev |  | TTCCATTCTTTTCTTCTATGAAT |  | 23 | 160 | 58.03 | False | 0.00 | 0.00 |
|  | OXA-66_detection |  | AATAAAACGCTTCC | TTT | 14 | 160 | 47.35 | False | 0.00 | 0.00 |
|  | OXA-66_lnc3 | GCAGCGTGGGCTCGCGTGCCGGGGTTTTTT | CTTACAAGCTAGCT |  | 14 | 160 | 48.13 | False | 0.00 | 0.00 |
| gadE | gadE_fwd |  | TATGGTAAACACTTGCCCCAT |  | 21 | 107 | 63.58 | False | 41.69 | -375.59 |
|  | gadE_rev |  | GACGTGATATTGCTTTCATTT |  | 21 | 107 | 58.91 | False | 0.00 | 0.00 |
|  | gadE_detection |  | TCAAAATTATGCAACTGA | TTT | 18 | 107 | 54.06 | False | 0.00 | 0.00 |
|  | gadE_lnc3 | GCAGCGTGGGCTCGCGTGCCGGGGTTTTTT | TGTTTTTCACATAATCAGT |  | 19 | 107 | 54.57 | False | 0.00 | 0.00 |
| acrA_Escherichia | acrA_Escherichia_fwd |  | TCAAAACTGAACCTCTGCA |  | 19 | 120 | 60.63 | False | 0.00 | 0.00 |
|  | acrA_Escherichia_rev |  | TACCTTCTTTGAAATTACGCT |  | 21 | 120 | 59.30 | False | 0.00 | 0.00 |
|  | acrA_Escherichia_lnc3 | GCAGCGTGGGCTCGCGTGCCGGGGTTTTTT | GCAGAAGTTCGTCC |  | 14 | 120 | 54.18 | False | 0.00 | 0.00 |
|  | acrA_Escherichia_detection |  | TCAAGTTAGCGGGAT | TTT | 15 | 120 | 54.90 | False | 0.00 | 0.00 |
| mdtG | mdtG_fwd |  | TCACCCTGTGRAAATGA |  | 17 | 76 | 56.96 | True | 45.48 | -622.58 |
|  | mdtG_rev |  | CSGTAAGAAAACAGCCTAG |  | 19 | 76 | 59.04 | False | 43.02 | -184.60 |
|  | mdtG_detection |  | AAACCTGATCGTCG | TTT | 14 | 76 | 52.78 | False | 0.00 | 0.00 |
|  | mdtG_lnc3 | GCAGCGTGGGCTCGCGTGCCGGGGTTTTTT | CTATAAACTGGAAACG |  | 16 | 76 | 49.75 | False | 0.00 | 0.00 |
| tet(A) | tet(A)_fwd |  | TTTGCTCCTTGGCTTGGAA |  | 19 | 110 | 63.68 | False | 36.50 | 35.31 |
|  | tet(A)_rev |  | GCACTTGAAAAAGCCAGCAAT |  | 21 | 110 | 64.62 | False | 0.00 | 0.00 |
|  | tet(A)_lnc3 | GCAGCGTGGGCTCGCGTGCCGGGGTTTTTT | CCAGTGCTGTTGTT |  | 14 | 110 | 53.68 | False | 0.00 | 0.00 |
|  | tet(A)_detection |  | GTCATTAATAGGCGC | TTT | 15 | 110 | 52.33 | False | 0.00 | 0.00 |
| mdfA | mdfA_fwd |  | CAGTCGCCGATTATCAT |  | 17 | 143 | 56.93 | False | 0.00 | 0.00 |
|  | mdfA_rev |  | ATCAGCGAACGTACGGT |  | 17 | 143 | 61.71 | True | 46.45 | -736.21 |
|  | mdfA_lnc3 | GCAGCGTGGGCTCGCGTGCCGGGGTTTTTT | CTATTTTCGGGGCG |  | 14 | 143 | 54.34 | False | 0.00 | 0.00 |
|  | mdfA_detection |  | TTAATTGCGGGTAACT | TTT | 16 | 143 | 53.91 | False | 0.00 | 0.00 |
| PmrC | PmrC_fwd |  | ACGTCGACTATATTGTTGAT |  | 20 | 123 | 57.90 | False | 37.80 | -75.31 |
|  | PmrC_rev |  | GCAGATAGATGCCATTTTC |  | 19 | 123 | 57.59 | False | 44.22 | -532.64 |
|  | PmrC_detection |  | TTTACCACCAGCCT | TTT | 14 | 123 | 53.82 | False | 0.00 | 0.00 |
|  | PmrC_lnc3 | GCAGCGTGGGCTCGCGTGCCGGGGTTTTTT | AGAACATCAGGATAAA |  | 16 | 123 | 49.37 | False | 0.00 | 0.00 |
| mdsB | mdsB_fwd |  | CGCCTATGTCATTACTCT |  | 18 | 128 | 56.64 | False | 0.00 | 0.00 |
|  | mdsB_rev |  | CTCGACAATCAAAATGGC |  | 18 | 128 | 57.67 | False | 0.00 | 0.00 |
|  | mdsB_detection |  | CAATATCTTTACGCAG | TTT | 16 | 128 | 50.37 | False | 0.00 | 0.00 |
|  | mdsB_lnc3 | GCAGCGTGGGCTCGCGTGCCGGGGTTTTTT | TCTGGCGGAGATAA |  | 14 | 128 | 52.86 | False | 0.00 | 0.00 |
| SHV-12 | SHV-12_fwd |  | RTCTGAGCGCCCGTTC |  | 16 | 113 | 62.79 | False | 0.00 | 0.00 |
|  | SHV-12_rev |  | GGTCTTATCGGCGATAAAC |  | 19 | 113 | 59.27 | False | 40.40 | -324.09 |
|  | SHV-12_detection |  | GTGGACGATCGG | TTT | 12 | 113 | 50.70 | False | 0.00 | 0.00 |
|  | SHV-12_lnc3 | GCAGCGTGGGCTCGCGTGCCGGGGTTTTTT | CTGCAGTGGATG |  | 12 | 113 | 47.31 | False | 0.00 | 0.00 |
| mdtO | mdtO_fwd |  | CAACTGGACAGCGAAGA |  | 17 | 121 | 59.78 | False | 0.00 | 0.00 |
|  | mdtO_rev |  | CGTTATCCAGCGCCTGC |  | 17 | 121 | 64.36 | False | 43.24 | -341.00 |
|  | mdtO_lnc3 | GCAGCGTGGGCTCGCGTGCCGGGGTTTTTT | CTGATTGAACGTTC |  | 14 | 121 | 48.20 | False | 0.00 | 0.00 |
|  | mdtO_detection |  | GCAAACGGTTATTC | TTT | 14 | 121 | 49.08 | False | 0.00 | 0.00 |
| MsrA | MsrA_fwd |  | GTAAAGCTAAACGAAATCAAGCG |  | 23 | 148 | 63.07 | False | 41.57 | -379.26 |
|  | MsrA_rev |  | TTTTCAATATGCTTAGCTTGTT |  | 22 | 148 | 58.79 | False | 0.00 | 0.00 |
|  | MsrA_lnc3 | GCAGCGTGGGCTCGCGTGCCGGGGTTTTTT | AAAAGTATAGCACCA |  | 15 | 148 | 48.78 | False | 0.00 | 0.00 |
|  | MsrA_detection |  | GATCGTTTAAGTGCA | TTT | 15 | 148 | 51.49 | False | 0.00 | 0.00 |
| mdtM | mdtM_fwd |  | GGTTATGTCACGGTGCA |  | 17 | 148 | 60.20 | False | 0.00 | 0.00 |
|  | mdtM_rev |  | CAAAAAGGACTTTCCAGTG |  | 19 | 148 | 57.82 | False | 44.45 | -429.16 |
|  | mdtM_detection |  | CCTCCATCGTACTGA | TTT | 15 | 148 | 54.69 | False | 0.00 | 0.00 |
|  | mdtM_lnc3 | GCAGCGTGGGCTCGCGTGCCGGGGTTTTTT | TGATGGCGATTATCA |  | 15 | 148 | 52.18 | False | 34.89 | 175.39 |

### ABR-3

Supplementary Table S6: Primer and probe sequences and additional information of ABR-3.

| Gene | Primer/Probe | Hybridisation Sequence | Sequence | Spacer | Length | Product Size | Tm | Hairpin | Hairpin Tm | Hairpin delta G |
| --- | --- | --- | --- | --- | --- | --- | --- | --- | --- | --- |
| mel | mel_fwd |  | GCCATGACCGCTATTTTCTTG |  | 21 | 123 | 63.95 | False | 0.00 | 0.00 |
|  | mel_rev |  | TCTTACGTTCTTCCTCTTT |  | 19 | 123 | 56.75 | False | 0.00 | 0.00 |
|  | mel_lnc3 | GCAGCGTGGGCTCGCGTGCCGGGGTTTTTT | ACTGAGTATTGGGGAA |  | 16 | 123 | 54.88 | False | 0.00 | 0.00 |
|  | mel_detection |  | ACTATTCTGATTATCTTC | TTT | 18 | 123 | 49.41 | False | 0.00 | 0.00 |
| adeI | adeI_fwd |  | AAGATGGTTCTACCTATCC |  | 19 | 107 | 56.31 | False | 43.94 | -444.20 |
|  | adeI_rev |  | ATTCGGGTTAGAGAATACG |  | 19 | 107 | 57.50 | False | 40.20 | -283.49 |
|  | adeI_lnc3 | GCAGCGTGGGCTCGCGTGCCGGGGTTTTTT | CAACTTGCTTTCTCT |  | 15 | 107 | 51.15 | False | 0.00 | 0.00 |
|  | adeI_detection |  | GACGCTTCTGTAAA | TTT | 14 | 107 | 49.62 | False | 0.00 | 0.00 |
| tet(D) | tet(D)_fwd |  | TAACATCCAATTAAGATATGATG |  | 23 | 89 | 56.11 | False | 0.00 | 0.00 |
|  | tet(D)_rev |  | ACTGATCTAAATTATGTTCAAT |  | 22 | 89 | 55.09 | False | 0.00 | 0.00 |
|  | tet(D)_lnc3 | GCAGCGTGGGCTCGCGTGCCGGGGTTTTTT | AGTTTCATACAACGG |  | 15 | 89 | 50.91 | False | 0.00 | 0.00 |
|  | tet(D)_detection |  | TTATCAAAGATGTTCTAT | TTT | 18 | 89 | 49.11 | False | 0.00 | 0.00 |
| adeJ | adeJ_fwd |  | TGTACTYGGTGGCGTAC |  | 17 | 123 | 60.57 | False | 39.83 | -296.28 |
|  | adeJ_rev |  | GATTTATGCTCCTGAGTGTTTA |  | 22 | 123 | 60.06 | False | 0.00 | 0.00 |
|  | adeJ_detection |  | GTGGATTCGTAGTAT | TTT | 15 | 123 | 48.98 | False | 0.00 | 0.00 |
|  | adeJ_lnc3 | GCAGCGTGGGCTCGCGTGCCGGGGTTTTTT | CCCTGTATTCTATGT |  | 15 | 123 | 48.21 | False | 0.00 | 0.00 |
| oqxA | oqxA_fwd |  | CCGCTAAGGTGCTGGTG |  | 17 | 116 | 63.53 | False | 36.82 | 10.51 |
|  | oqxA_rev |  | ATTCACTTTATCAATGTATCCC |  | 22 | 116 | 57.83 | False | 0.00 | 0.00 |
|  | oqxA_lnc3 | GCAGCGTGGGCTCGCGTGCCGGGGTTTTTT | GATCAGTCAGTGGG |  | 14 | 116 | 52.24 | False | 0.00 | 0.00 |
|  | oqxA_detection |  | ATAGTTTTAACGGTCG | TTT | 16 | 116 | 51.96 | False | 0.00 | 0.00 |
| farB | farB_fwd |  | GTCGTATTTTATTGTTTGGGAAT |  | 23 | 129 | 59.80 | False | 0.00 | 0.00 |
|  | farB_rev |  | AGCGTCCCCATATACACCA |  | 19 | 129 | 64.01 | False | 0.00 | 0.00 |
|  | farB_detection |  | GATTTATCGCTGTT | TTT | 14 | 129 | 46.67 | False | 0.00 | 0.00 |
|  | farB_lnc3 | GCAGCGTGGGCTCGCGTGCCGGGGTTTTTT | AATATCCGATTGTC |  | 14 | 129 | 45.22 | False | 0.00 | 0.00 |
| ADC-2 | ADC-2_fwd |  | TCTATGAATAAACCTTTCGA |  | 20 | 135 | 55.19 | False | 0.00 | 0.00 |
|  | ADC-2_rev |  | CGGCTGATTTTCTTGGTTATA |  | 21 | 135 | 60.07 | False | 0.00 | 0.00 |
|  | ADC-2_lnc3 | GCAGCGTGGGCTCGCGTGCCGGGGTTTTTT | CAATTTTTCCGGCC |  | 14 | 135 | 52.78 | False | 0.00 | 0.00 |
|  | ADC-2_detection |  | CTTGGCTTAAAACATAG | TTT | 17 | 135 | 51.58 | False | 0.00 | 0.00 |
| adeS | adeS_fwd |  | CGATTTATTTAAGCCTTTCT |  | 20 | 142 | 55.05 | False | 0.00 | 0.00 |
|  | adeS_rev |  | ACTTTTCGAGCCTTGATT |  | 18 | 142 | 57.66 | False | 0.00 | 0.00 |
|  | adeS_detection |  | TTGCTGTTGTACAT | TTT | 14 | 142 | 48.63 | False | 0.00 | 0.00 |
|  | adeS_lnc3 | GCAGCGTGGGCTCGCGTGCCGGGGTTTTTT | CACAGGTTTAGGTC |  | 14 | 142 | 49.48 | False | 0.00 | 0.00 |
| MphD | MphD_fwd |  | TCAGATGAATATGGCTTATGT |  | 21 | 98 | 58.29 | False | 0.00 | 0.00 |
|  | MphD_rev |  | CAAGATGCCGTTTTAAAG |  | 18 | 98 | 55.23 | False | 0.00 | 0.00 |
|  | MphD_detection |  | TATATCCTCAAAAAGAAG | TTT | 18 | 98 | 49.97 | False | 0.00 | 0.00 |
|  | MphD_lnc3 | GCAGCGTGGGCTCGCGTGCCGGGGTTTTTT | ATCCCTATTTTAAAGAAAT |  | 19 | 98 | 50.81 | False | 0.00 | 0.00 |
| LsaA | LsaA_fwd |  | AAAAATTAAGAGATGCTTTTGAA |  | 23 | 98 | 57.67 | False | 41.58 | -359.43 |
|  | LsaA_rev |  | CGACCATTCCGCTTTTTTA |  | 19 | 98 | 59.94 | False | 0.00 | 0.00 |
|  | LsaA_lnc3 | GCAGCGTGGGCTCGCGTGCCGGGGTTTTTT | AAAAATCAAAAAAGAAGTC |  | 19 | 98 | 51.96 | False | 0.00 | 0.00 |
|  | LsaA_detection |  | AATCGCTTGAAAGAAAC | TTT | 17 | 98 | 54.77 | False | 0.00 | 0.00 |
| macB | macB_fwd |  | GATCCGTTGGGTAAAACC |  | 18 | 117 | 58.91 | False | 40.00 | -173.33 |
|  | macB_rev |  | ATAGGGCGACCAAAGCA |  | 17 | 117 | 61.67 | True | 63.30 | -1406.97 |
|  | macB_detection |  | CCTTGACCGTCATC | TTT | 14 | 117 | 53.28 | False | 0.00 | 0.00 |
|  | macB_lnc3 | GCAGCGTGGGCTCGCGTGCCGGGGTTTTTT | GTTCAGGAAACGCC |  | 14 | 117 | 54.96 | False | 0.00 | 0.00 |
| vgaC | vgaC_fwd |  | GGAGATCGTCCGGGAAGG |  | 18 | 102 | 64.90 | False | 42.97 | -340.06 |
|  | vgaC_rev |  | TCCGCCATAAAGTCCGG |  | 17 | 102 | 62.15 | False | 43.65 | -555.95 |
|  | vgaC_detection |  | CTCGTTCGCGC | TTT | 11 | 102 | 52.11 | True | 42.58 | -381.68 |
|  | vgaC_lnc3 | GCAGCGTGGGCTCGCGTGCCGGGGTTTTTT | CCGACCTGGGG |  | 11 | 102 | 51.57 | False | 0.00 | 0.00 |
| mtrE | mtrE_fwd |  | AGCTGATCGACATCGCAC |  | 18 | 109 | 63.47 | False | 41.64 | -316.75 |
|  | mtrE_rev |  | GTGGGCAGGAGGTTGTTG |  | 18 | 109 | 64.27 | False | 0.00 | 0.00 |
|  | mtrE_lnc3 | GCAGCGTGGGCTCGCGTGCCGGGGTTTTTT | TGAACAGCGAAATC |  | 14 | 109 | 50.55 | False | 0.00 | 0.00 |
|  | mtrE_detection |  | TACCGCAAACAATA | TTT | 14 | 109 | 48.38 | False | 0.00 | 0.00 |
| adeG | adeG_fwd |  | GTCCAATGTTCTCAAGTTAT |  | 20 | 135 | 56.78 | False | 0.00 | 0.00 |
|  | adeG_rev |  | CGTACTGAGAACCTAAATAAATCT |  | 24 | 135 | 60.42 | False | 0.00 | 0.00 |
|  | adeG_lnc3 | GCAGCGTGGGCTCGCGTGCCGGGGTTTTTT | GTAGATCTGGACCGT |  | 15 | 135 | 54.68 | False | 0.00 | 0.00 |
|  | adeG_detection |  | GTAAAAGCTAAACAGC | TTT | 16 | 135 | 51.24 | False | 0.00 | 0.00 |
| mtrC | mtrC_fwd |  | GGAATCGCTGCGTACCG |  | 17 | 130 | 64.00 | False | 0.00 | 0.00 |
|  | mtrC_rev |  | TGCTTCATAAGTGGAACTGT |  | 20 | 130 | 60.75 | True | 62.58 | -997.95 |
|  | mtrC_detection |  | GGCAGTTATGTCCG | TTT | 14 | 130 | 53.81 | False | 0.00 | 0.00 |
|  | mtrC_lnc3 | GCAGCGTGGGCTCGCGTGCCGGGGTTTTTT | GCCTGTTCCAAGAA |  | 14 | 130 | 52.77 | False | 0.00 | 0.00 |
| dfrC | dfrC_fwd |  | TCACTAACCAAGCTTCATT |  | 19 | 134 | 57.75 | False | 0.00 | 0.00 |
|  | dfrC_rev |  | ATCTACCTGGTCAATCAT |  | 18 | 134 | 55.24 | False | 0.00 | 0.00 |
|  | dfrC_lnc3 | GCAGCGTGGGCTCGCGTGCCGGGGTTTTTT | TATCTGGTCATGTTT |  | 15 | 134 | 48.34 | False | 0.00 | 0.00 |
|  | dfrC_detection |  | TTATATTTGGAGGAC | TTT | 15 | 134 | 45.65 | False | 0.00 | 0.00 |
| adeB | adeB_fwd |  | GGATTAACTCTCCAAATAATC |  | 21 | 140 | 55.39 | False | 38.60 | -192.05 |
|  | adeB_rev |  | CCAAATACGCATAGCTTT |  | 18 | 140 | 56.26 | False | 0.00 | 0.00 |
|  | adeB_detection |  | GTGTCGAAGGTGTAG | TTT | 15 | 140 | 54.02 | False | 0.00 | 0.00 |
|  | adeB_lnc3 | GCAGCGTGGGCTCGCGTGCCGGGGTTTTTT | TGTAGAAGAGCTAAAAC |  | 17 | 140 | 52.09 | False | 0.00 | 0.00 |
| MsrE | MsrE_fwd |  | ACTGGGCGGAATCGAAATA |  | 19 | 113 | 62.64 | False | 41.38 | -256.22 |
|  | MsrE_rev |  | TTTTTGCACGAGTTTCCT |  | 18 | 113 | 58.57 | False | 0.00 | 0.00 |
|  | MsrE_lnc3 | GCAGCGTGGGCTCGCGTGCCGGGGTTTTTT | GGGCAATGTTATCTC |  | 15 | 113 | 51.57 | False | 0.00 | 0.00 |
|  | MsrE_detection |  | GCCTTGGTGTTTCC | TTT | 14 | 113 | 54.75 | False | 0.00 | 0.00 |
| CTX-M-15 | CTX-M-15_fwd |  | CTGTTGTTAGGAAGTGTGCC |  | 20 | 140 | 62.67 | False | 0.00 | 0.00 |
|  | CTX-M-15_rev |  | GCACGATAAAGTATTTGCGAAT |  | 22 | 140 | 61.89 | False | 44.91 | -417.88 |
|  | CTX-M-15_detection |  | ACTTGCCGAATTAGAG | TTT | 16 | 140 | 54.58 | False | 0.00 | 0.00 |
|  | CTX-M-15_lnc3 | GCAGCGTGGGCTCGCGTGCCGGGGTTTTTT | GGACGTACAGCAAAA |  | 15 | 140 | 54.62 | False | 0.00 | 0.00 |
| floR | floR_fwd |  | TCGGATTCAGCTTTGCCT |  | 18 | 96 | 62.63 | False | 0.00 | 0.00 |
|  | floR_rev |  | ATCCTGCGATGCCCCAT |  | 17 | 96 | 64.48 | False | 0.00 | 0.00 |
|  | floR_detection |  | GATCGTGACAACCC | TTT | 14 | 96 | 53.72 | False | 0.00 | 0.00 |
|  | floR_lnc3 | GCAGCGTGGGCTCGCGTGCCGGGGTTTTTT | GTCGCGCTTGTAAT |  | 14 | 96 | 54.30 | False | 0.00 | 0.00 |
| adeC | adeC_fwd |  | TTGGGGAACAAGAAAAAC |  | 18 | 126 | 56.44 | False | 37.70 | -42.13 |
|  | adeC_rev |  | TGYGCATGTGTAGCAAG |  | 17 | 126 | 59.69 | True | 48.73 | -812.81 |
|  | adeC_detection |  | TCAATCAGCTTTTCGT | TTT | 16 | 126 | 54.73 | False | 0.00 | 0.00 |
|  | adeC_lnc3 | GCAGCGTGGGCTCGCGTGCCGGGGTTTTTT | CTAAATATGAAAAAGCCAT |  | 19 | 126 | 52.87 | False | 0.00 | 0.00 |
| KPC-3 | KPC-3_fwd |  | GCTGTCTTGTCTCTCAT |  | 17 | 147 | 56.20 | False | 0.00 | 0.00 |
|  | KPC-3_rev |  | CTTACAGTTGCGCCTGA |  | 17 | 147 | 60.20 | False | 39.46 | -214.60 |
|  | KPC-3_lnc3 | GCAGCGTGGGCTCGCGTGCCGGGGTTTTTT | GGAACCATTCGCTA |  | 14 | 147 | 52.22 | False | 0.00 | 0.00 |
|  | KPC-3_detection |  | AACTCGAACAGGAC | TTT | 14 | 147 | 52.07 | False | 0.00 | 0.00 |
| macA | macA_fwd |  | TATGCCCGTTCGTTTGTG |  | 18 | 126 | 61.60 | False | 0.00 | 0.00 |
|  | macA_rev |  | GCGATTTTTCACGGTCAG |  | 18 | 126 | 60.34 | False | 0.00 | 0.00 |
|  | macA_lnc3 | GCAGCGTGGGCTCGCGTGCCGGGGTTTTTT | ACGACGCAGAATAC |  | 14 | 126 | 52.96 | False | 0.00 | 0.00 |
|  | macA_detection |  | GGTTGAAATCGACG | TTT | 14 | 126 | 51.77 | False | 0.00 | 0.00 |
| adeF | adeF_fwd |  | GTATTCAACGTCTCATTCA |  | 19 | 114 | 56.08 | False | 0.00 | 0.00 |
|  | adeF_rev |  | ATTGGACAGCAGCTCTAG |  | 18 | 114 | 59.62 | False | 0.00 | 0.00 |
|  | adeF_lnc3 | GCAGCGTGGGCTCGCGTGCCGGGGTTTTTT | AAGAACTGGATTTAGC |  | 16 | 114 | 51.66 | False | 0.00 | 0.00 |
|  | adeF_detection |  | CGAAAATGATGCACG | TTT | 15 | 114 | 54.36 | False | 0.00 | 0.00 |
| mphD | mphD_fwd |  | AAATCCATAGTATTCCTGAAAAA |  | 23 | 110 | 58.02 | False | 0.00 | 0.00 |
|  | mphD_rev |  | TTCAGATTTTACTAACTGCAAAT |  | 23 | 110 | 58.83 | False | 0.00 | 0.00 |
|  | mphD_lnc3 | GCAGCGTGGGCTCGCGTGCCGGGGTTTTTT | ATTTGAAAATTATGAAA |  | 17 | 110 | 45.06 | False | 0.00 | 0.00 |
|  | mphD_detection |  | CCTTCAGATTTAAGA | TTT | 15 | 110 | 46.05 | False | 0.00 | 0.00 |
| mtrD | mtrD_fwd |  | CGTCCGATTATCATGACCTC |  | 20 | 117 | 61.51 | False | 0.00 | 0.00 |
|  | mtrD_rev |  | CATCCCCCAGAATACGGT |  | 18 | 117 | 61.75 | False | 0.00 | 0.00 |
|  | mtrD_lnc3 | GCAGCGTGGGCTCGCGTGCCGGGGTTTTTT | TTATTTTGGGCGTG |  | 14 | 117 | 50.83 | False | 0.00 | 0.00 |
|  | mtrD_detection |  | GTTCCCCTGTATATT | TTT | 15 | 117 | 48.55 | False | 0.00 | 0.00 |
| BlaA1 | BlaA1_fwd |  | CTTCATGCTTGGGACGA |  | 17 | 134 | 59.86 | False | 0.00 | 0.00 |
|  | BlaA1_rev |  | GAACGCCACCAATAATTTTCA |  | 21 | 134 | 61.45 | False | 0.00 | 0.00 |
|  | BlaA1_lnc3 | GCAGCGTGGGCTCGCGTGCCGGGGTTTTTT | CTTTGGCTCAACAA |  | 14 | 134 | 50.57 | False | 0.00 | 0.00 |
|  | BlaA1_detection |  | AATAATTTAGCGATTAG | TTT | 17 | 134 | 47.29 | False | 0.00 | 0.00 |
| mtrR | mtrR_fwd |  | CTGGCGCGAGAAAATTAC |  | 18 | 121 | 60.11 | False | 0.00 | 0.00 |
|  | mtrR_rev |  | CCAAATCAGCCCGTCCAA |  | 18 | 121 | 63.66 | False | 0.00 | 0.00 |
|  | mtrR_detection |  | ATTTGGACAAGGAAAC | TTT | 16 | 121 | 52.66 | False | 0.00 | 0.00 |
|  | mtrR_lnc3 | GCAGCGTGGGCTCGCGTGCCGGGGTTTTTT | GGATTTGGCTGACG |  | 14 | 121 | 54.63 | False | 0.00 | 0.00 |
| Sat-2A | Sat-2A_fwd |  | ATGAAGATTTCGGTGATCCCTG |  | 22 | 123 | 64.42 | False | 0.00 | 0.00 |
|  | Sat-2A_rev |  | ACTTCTGGTAGATAGTTCAA |  | 20 | 123 | 56.63 | False | 0.00 | 0.00 |
|  | Sat-2A_lnc3 | GCAGCGTGGGCTCGCGTGCCGGGGTTTTTT | CGGAAACATTGGAT |  | 14 | 123 | 49.91 | False | 0.00 | 0.00 |
|  | Sat-2A_detection |  | GCTGAGAACCATTT | TTT | 14 | 123 | 49.77 | False | 0.00 | 0.00 |
| CMY-2 | CMY-2_fwd |  | CGTTGAGGTAAACCCGCC |  | 18 | 111 | 64.34 | False | 41.32 | -233.29 |
|  | CMY-2_rev |  | CCAAGGTTTTTTTCTGGAACGAA |  | 23 | 111 | 64.44 | False | 0.00 | 0.00 |
|  | CMY-2_detection |  | TGGGTGCATAAAA | TTT | 13 | 111 | 46.69 | False | 0.00 | 0.00 |
|  | CMY-2_lnc3 | GCAGCGTGGGCTCGCGTGCCGGGGTTTTTT | GTGAAAGCCTCA |  | 12 | 111 | 45.71 | False | 0.00 | 0.00 |
| OXA-23 | OXA-23_fwd |  | AGAATATGTGCCAGCCTCTA |  | 20 | 96 | 61.95 | False | 0.00 | 0.00 |
|  | OXA-23_rev |  | CCCTTCCATTTAAATATTTCAT |  | 22 | 96 | 56.32 | False | 0.00 | 0.00 |
|  | OXA-23_lnc3 | GCAGCGTGGGCTCGCGTGCCGGGGTTTTTT | TGTTGAATGCCCTG |  | 14 | 96 | 53.30 | False | 0.00 | 0.00 |
|  | OXA-23_detection |  | ATCGGATTGGAGAAC | TTT | 15 | 96 | 53.03 | False | 0.00 | 0.00 |
| Mbl | Mbl_fwd |  | CACGTYTAGGTCAGAATCG |  | 19 | 123 | 60.10 | False | 38.92 | -110.55 |
|  | Mbl_rev |  | GTGAARTTGAGCCGCAA |  | 17 | 123 | 60.16 | False | 43.61 | -446.27 |
|  | Mbl_lnc3 | GCAGCGTGGGCTCGCGTGCCGGGGTTTTTT | ATTAATGAATAACTTAAATC |  | 20 | 123 | 48.43 | False | 0.00 | 0.00 |
|  | Mbl_detection |  | TGCCATATCCAAAAC | TTT | 15 | 123 | 51.24 | False | 0.00 | 0.00 |
| adeK | adeK_fwd |  | CAAAAGCACGTTTATTCCC |  | 19 | 139 | 58.67 | False | 0.00 | 0.00 |
|  | adeK_rev |  | CCCCAGTCAAAGATTGG |  | 17 | 139 | 57.63 | False | 43.16 | -596.09 |
|  | adeK_lnc3 | GCAGCGTGGGCTCGCGTGCCGGGGTTTTTT | TTATGCATCAACTGA |  | 15 | 139 | 49.39 | False | 0.00 | 0.00 |
|  | adeK_detection |  | CTTAAGTGATCTATTTAA | TTT | 18 | 139 | 47.22 | False | 0.00 | 0.00 |
| oqxB | oqxB_fwd |  | CCCTACCCGGCTGATCG |  | 17 | 87 | 64.82 | False | 0.00 | 0.00 |
|  | oqxB_rev |  | CCCTGATAGCCGTTCGA |  | 17 | 87 | 61.47 | False | 0.00 | 0.00 |
|  | oqxB_lnc3 | GCAGCGTGGGCTCGCGTGCCGGGGTTTTTT | TTTTCCGTCCGTTTA |  | 15 | 87 | 53.16 | False | 0.00 | 0.00 |
|  | oqxB_detection |  | ACCGCTTTTTCCTG | TTT | 14 | 87 | 53.80 | False | 0.00 | 0.00 |
| Mrx | Mrx_fwd |  | CGACCTTTCTCTTCCGGAT |  | 19 | 128 | 62.61 | False | 0.00 | 0.00 |
|  | Mrx_rev |  | GATGATGGTCGCGATGA |  | 17 | 128 | 59.78 | False | 0.00 | 0.00 |
|  | Mrx_detection |  | ATACAAAGAGCGCG | TTT | 14 | 128 | 53.87 | False | 0.00 | 0.00 |
|  | Mrx_lnc3 | GCAGCGTGGGCTCGCGTGCCGGGGTTTTTT | GTTCGTCCTGTCTC |  | 14 | 128 | 52.84 | False | 0.00 | 0.00 |
| ErmC | ErmC_fwd |  | TTTTAATGGCAGAAGTTGA |  | 19 | 82 | 56.04 | False | 0.00 | 0.00 |
|  | ErmC_rev |  | GAGCTATTCACTTTAGGTTT |  | 20 | 82 | 56.48 | False | 0.00 | 0.00 |
|  | ErmC_detection |  | CCAAGAGAATATTTTC | TTT | 16 | 82 | 46.85 | False | 0.00 | 0.00 |
|  | ErmC_lnc3 | GCAGCGTGGGCTCGCGTGCCGGGGTTTTTT | CTATATTAAGTATGGTT |  | 17 | 82 | 45.03 | False | 0.00 | 0.00 |
| APH(3')-Ia | APH(3')-Ia_fwd |  | AACGGTTTGGTTGATGC |  | 17 | 106 | 59.02 | False | 0.00 | 0.00 |
|  | APH(3')-Ia_rev |  | CTGAATCCGGTGAGAATGGC |  | 20 | 106 | 64.76 | False | 0.00 | 0.00 |
|  | APH(3')-Ia_detection |  | CTGGAAAGAAATGC | TTT | 14 | 106 | 48.36 | False | 0.00 | 0.00 |
|  | APH(3')-Ia_lnc3 | GCAGCGTGGGCTCGCGTGCCGGGGTTTTTT | CCTGTTGAACAAGT |  | 14 | 106 | 49.58 | False | 0.00 | 0.00 |
| abeS | abeS_fwd |  | ATCGGGATTGCCTATGCC |  | 18 | 97 | 62.95 | False | 44.46 | -629.54 |
|  | abeS_rev |  | AGGCAGCYAAGTCTAAAT |  | 18 | 97 | 57.70 | False | 0.00 | 0.00 |
|  | abeS_lnc3 | GCAGCGTGGGCTCGCGTGCCGGGGTTTTTT | AGGTATTATTTTAATTTCTG |  | 20 | 97 | 50.55 | False | 0.00 | 0.00 |
|  | abeS_detection |  | CAATTGGCTGGATATTTT | TTT | 18 | 97 | 54.96 | False | 0.00 | 0.00 |
| MefA | MefA_fwd |  | GGTCTTGTCTATGGCTTCAYTA |  | 22 | 129 | 62.65 | False | 35.85 | 0.00 |
|  | MefA_rev |  | GCTGCTGCGATAATTAAATC |  | 20 | 129 | 59.09 | False | 0.00 | 0.00 |
|  | MefA_lnc3 | GCAGCGTGGGCTCGCGTGCCGGGGTTTTTT | AGTGGATCGTCATGA |  | 15 | 129 | 54.53 | False | 0.00 | 0.00 |
|  | MefA_detection |  | TAGGAAGAAGATAATGA | TTT | 17 | 129 | 49.20 | False | 0.00 | 0.00 |
| FosA5 | FosA5_fwd |  | ACTGAATCACCTGACCC |  | 17 | 121 | 58.64 | False | 0.00 | 0.00 |
|  | FosA5_rev |  | ATCGCCGCAGGAGAGATA |  | 18 | 121 | 63.59 | True | 70.40 | -2245.51 |
|  | FosA5_lnc3 | GCAGCGTGGGCTCGCGTGCCGGGGTTTTTT | GTTTTATCAGCAGC |  | 14 | 121 | 48.60 | False | 0.00 | 0.00 |
|  | FosA5_detection |  | TGCTGGGCATGA | TTT | 12 | 121 | 52.24 | True | 35.60 | 73.15 |
| adeA | adeA_fwd |  | CACATTCCGTATTGAAGTTAATAAC |  | 25 | 139 | 60.97 | False | 0.00 | 0.00 |
|  | adeA_rev |  | CTCGCCACTGATATTACGTTG |  | 21 | 139 | 62.84 | False | 0.00 | 0.00 |
|  | adeA_detection |  | CAAGCGCTATTGGT | TTT | 14 | 139 | 53.64 | False | 0.00 | 0.00 |
|  | adeA_lnc3 | GCAGCGTGGGCTCGCGTGCCGGGGTTTTTT | CGTGCTTCTATTCCT |  | 15 | 139 | 53.09 | False | 0.00 | 0.00 |
| acrA_Klebsiella | acrA_Klebsiella_fwd |  | GGCCTGCAAAAAATCAAAC |  | 19 | 96 | 59.87 | False | 0.00 | 0.00 |
|  | acrA_Klebsiella_rev |  | TTGTTCTGATGGCGCGT |  | 17 | 96 | 63.05 | False | 0.00 | 0.00 |
|  | acrA_Klebsiella_detection |  | GTAGCTTCTGATGAT | TTT | 15 | 96 | 49.31 | False | 0.00 | 0.00 |
|  | acrA_Klebsiella_lnc3 | GCAGCGTGGGCTCGCGTGCCGGGGTTTTTT | TAAAAGCGCAGGAA |  | 14 | 96 | 52.38 | False | 0.00 | 0.00 |
| adeR | adeR_fwd |  | CGGCGCTAGATCAAGATA |  | 18 | 108 | 59.55 | False | 0.00 | 0.00 |
|  | adeR_rev |  | CTGCCTGAACTCTAGCGA |  | 18 | 108 | 62.02 | False | 0.00 | 0.00 |
|  | adeR_detection |  | CAGATGACTTTGTGG | TTT | 15 | 108 | 51.83 | False | 0.00 | 0.00 |
|  | adeR_lnc3 | GCAGCGTGGGCTCGCGTGCCGGGGTTTTTT | CATTACGCATAGGTG |  | 15 | 108 | 51.84 | False | 0.00 | 0.00 |
| PDC-1 | PDC-1_fwd |  | GGTGATGAAGGCCAATGAC |  | 19 | 93 | 62.30 | False | 0.00 | 0.00 |
|  | PDC-1_rev |  | TCTTTCGAGGCCAGCCC |  | 17 | 93 | 64.98 | False | 39.02 | -114.49 |
|  | PDC-1_lnc3 | GCAGCGTGGGCTCGCGTGCCGGGGTTTTTT | CCATCAGCCTGAAA |  | 14 | 93 | 52.42 | False | 0.00 | 0.00 |
|  | PDC-1_detection |  | GGAGAACCGCATTA | TTT | 14 | 93 | 52.22 | False | 0.00 | 0.00 |
| mphA | mphA_fwd |  | CCAACTGTACGCACTTGCA |  | 19 | 120 | 64.66 | False | 44.64 | -442.31 |
|  | mphA_rev |  | ACCCACCGACGTCCATC |  | 17 | 120 | 64.60 | False | 0.00 | 0.00 |
|  | mphA_detection |  | CTTGGGCTCGACTA | TTT | 14 | 120 | 54.38 | False | 0.00 | 0.00 |
|  | mphA_lnc3 | GCAGCGTGGGCTCGCGTGCCGGGGTTTTTT | CTGACTGTCAATGAG |  | 15 | 120 | 51.09 | False | 0.00 | 0.00 |

### ABR-4

Supplementary Table S7: Primer and probe sequences and additional information of ABR-4.

| Gene | Primer/Probe | Hybridisation Sequence | Sequence | Spacer | Length | Product Size | Tm | Hairpin | Hairpin Tm | Hairpin delta G |
| --- | --- | --- | --- | --- | --- | --- | --- | --- | --- | --- |
| MexA | MexA_fwd |  | AACAACGAGCTGCTGCC |  | 17 | 116 | 64.11 | False | 43.18 | -363.18 |
|  | MexA_rev |  | GTAGCCTGGCCCTTGAG |  | 17 | 116 | 62.50 | False | 0.00 | 0.00 |
|  | MexA_lnc3 | GCAGCGTGGGCTCGCGTGCCGGGGTTTTTT | CAGTTGCAGGAAGG |  | 14 | 116 | 53.93 | False | 0.00 | 0.00 |
|  | MexA_detection |  | CGTCAAGCAGAAGG | TTT | 14 | 116 | 54.16 | False | 0.00 | 0.00 |
| Zn-dependent_hydrolase | Zn-dependent_hydrolase_fwd |  | CACATTCTTTACAGTTTGAT |  | 20 | 120 | 55.19 | False | 0.00 | 0.00 |
|  | Zn-dependent_hydrolase_rev |  | CTACCCATTGTTGCTTATTT |  | 20 | 120 | 57.43 | False | 0.00 | 0.00 |
|  | Zn-dependent_hydrolase_detection |  | GCAGGCAATTAATA | TTT | 14 | 120 | 46.43 | False | 0.00 | 0.00 |
|  | Zn-dependent_hydrolase_lnc3 | GCAGCGTGGGCTCGCGTGCCGGGGTTTTTT | TTGATGTAAATTCAGA |  | 16 | 120 | 47.45 | False | 0.00 | 0.00 |
| aad(6) | aad(6)_fwd |  | TATTGAAAGGTATATATCCGAGGA |  | 24 | 160 | 60.55 | False | 0.00 | 0.00 |
|  | aad(6)_rev |  | CTCCGGATAGGCATAATGAA |  | 20 | 160 | 60.52 | False | 43.12 | -365.44 |
|  | aad(6)_lnc3 | GCAGCGTGGGCTCGCGTGCCGGGGTTTTTT | AACATATGGGAAGC |  | 14 | 160 | 48.91 | False | 0.00 | 0.00 |
|  | aad(6)_detection |  | ATTATTTCTATGCCAT | TTT | 16 | 160 | 47.16 | False | 0.00 | 0.00 |
| mexK | mexK_fwd |  | CATGATCATGCGCAACTC |  | 18 | 89 | 60.18 | False | 35.21 | 126.86 |
|  | mexK_rev |  | CCTCGATGATGGCGTGC |  | 17 | 89 | 64.05 | False | 0.00 | 0.00 |
|  | mexK_lnc3 | GCAGCGTGGGCTCGCGTGCCGGGGTTTTTT | CAGATCGAACAGGA |  | 14 | 89 | 51.30 | False | 0.00 | 0.00 |
|  | mexK_detection |  | CATCAGCCATGGAC | TTT | 14 | 89 | 53.71 | False | 0.00 | 0.00 |
| CpxR | CpxR_fwd |  | CAGATGCAACTGGGCGAC |  | 18 | 110 | 64.72 | False | 38.74 | -97.48 |
|  | CpxR_rev |  | AGGAGCGCTTCGAGGAT |  | 17 | 110 | 63.62 | False | 0.00 | 0.00 |
|  | CpxR_lnc3 | GCAGCGTGGGCTCGCGTGCCGGGGTTTTTT | CGGCCAGGAGATC |  | 13 | 110 | 54.84 | False | 0.00 | 0.00 |
|  | CpxR_detection |  | AGCCTGACCCTTTC | TTT | 14 | 110 | 54.99 | False | 0.00 | 0.00 |
| MexE | MexE_fwd |  | GGAAACCACCCAAGGCAT |  | 18 | 144 | 63.64 | False | 0.00 | 0.00 |
|  | MexE_rev |  | TCGATGTAGCCCGAYAC |  | 17 | 144 | 60.07 | False | 35.03 | 0.00 |
|  | MexE_lnc3 | GCAGCGTGGGCTCGCGTGCCGGGGTTTTTT | AACAACCGCTGAAC |  | 14 | 144 | 54.29 | False | 0.00 | 0.00 |
|  | MexE_detection |  | GAGTGGGACGAATT | TTT | 14 | 144 | 52.09 | False | 0.00 | 0.00 |
| AAC(6')-Ii | AAC(6')-Ii_fwd |  | TCAATTACTTAGAAAAAGAAGT |  | 22 | 153 | 55.18 | False | 42.10 | -498.34 |
|  | AAC(6')-Ii_rev |  | GATGTTCACGAAGGTTCT |  | 18 | 153 | 57.78 | False | 0.00 | 0.00 |
|  | AAC(6')-Ii_lnc3 | GCAGCGTGGGCTCGCGTGCCGGGGTTTTTT | TGATTTAGACCATGGA |  | 16 | 153 | 52.18 | False | 0.00 | 0.00 |
|  | AAC(6')-Ii_detection |  | ACAACGTTAAGTCAA | TTT | 15 | 153 | 50.16 | False | 0.00 | 0.00 |
| MexF | MexF_fwd |  | GGCATCACCGTCGACAAG |  | 18 | 126 | 64.37 | False | 0.00 | 0.00 |
|  | MexF_rev |  | GGCCAGTTCGTCCTTCAC |  | 18 | 126 | 63.97 | False | 0.00 | 0.00 |
|  | MexF_lnc3 | GCAGCGTGGGCTCGCGTGCCGGGGTTTTTT | AACCGCTACGACAT |  | 14 | 126 | 54.81 | False | 0.00 | 0.00 |
|  | MexF_detection |  | GCTCTACCTGTCGA | TTT | 14 | 126 | 54.01 | False | 0.00 | 0.00 |
| NDM-1 | NDM-1_fwd |  | TGACAATATCACCGTTGG |  | 18 | 115 | 57.85 | False | 35.10 | 100.73 |
|  | NDM-1_rev |  | GTAGTGCTCAGTGTCGGC |  | 18 | 115 | 63.69 | False | 0.00 | 0.00 |
|  | NDM-1_detection |  | GCTGCCTGATCAAG | TTT | 14 | 115 | 54.18 | False | 0.00 | 0.00 |
|  | NDM-1_lnc3 | GCAGCGTGGGCTCGCGTGCCGGGGTTTTTT | CATCGCTTTTGGTG |  | 14 | 115 | 52.49 | False | 0.00 | 0.00 |
| AadA5 | AadA5_fwd |  | GTCGGATTGGAAGGGTG |  | 17 | 121 | 60.29 | False | 0.00 | 0.00 |
|  | AadA5_rev |  | CAAACGCTCCGATACCCA |  | 18 | 121 | 63.05 | False | 0.00 | 0.00 |
|  | AadA5_lnc3 | GCAGCGTGGGCTCGCGTGCCGGGGTTTTTT | GTCGTTCTTGCTCTT |  | 15 | 121 | 54.24 | False | 0.00 | 0.00 |
|  | AadA5_detection |  | GCTCGCATTTGGTA | TTT | 14 | 121 | 53.66 | False | 0.00 | 0.00 |
| mexW | mexW_fwd |  | TGATCATGGTCACCGTG |  | 17 | 144 | 59.52 | False | 44.85 | -669.02 |
|  | mexW_rev |  | ACTCGACGATCAGGATGC |  | 18 | 144 | 62.48 | False | 38.12 | -45.88 |
|  | mexW_detection |  | CTATACCCAGGTCG | TTT | 14 | 144 | 51.10 | False | 0.00 | 0.00 |
|  | mexW_lnc3 | GCAGCGTGGGCTCGCGTGCCGGGGTTTTTT | TCGAGCCTGAACAT |  | 14 | 144 | 54.20 | False | 0.00 | 0.00 |
| mexP | mexP_fwd |  | TGTCGGGCATATCCAGG |  | 17 | 114 | 61.49 | False | 38.77 | -95.32 |
|  | mexP_rev |  | GTGCGCTCGTCGACATC |  | 17 | 114 | 64.26 | False | 44.20 | -399.08 |
|  | mexP_detection |  | CACCAATGGCGTC | TTT | 13 | 114 | 53.87 | False | 0.00 | 0.00 |
|  | mexP_lnc3 | GCAGCGTGGGCTCGCGTGCCGGGGTTTTTT | AGGGCAACTACGT |  | 13 | 114 | 52.74 | False | 0.00 | 0.00 |
| OXA-9 | OXA-9_fwd |  | TTCCGCCACTCTCCCAAT |  | 18 | 90 | 64.39 | False | 0.00 | 0.00 |
|  | OXA-9_rev |  | GTATTTCCGGTAATGGCG |  | 18 | 90 | 59.16 | False | 0.00 | 0.00 |
|  | OXA-9_lnc3 | GCAGCGTGGGCTCGCGTGCCGGGGTTTTTT | CGTGGCTTCTGATG |  | 14 | 90 | 54.26 | False | 0.00 | 0.00 |
|  | OXA-9_detection |  | AGGTTGAAACGCTT | TTT | 14 | 90 | 52.67 | False | 0.00 | 0.00 |
| TriC | TriC_fwd |  | ATGAACAGCGTGGTGACC |  | 18 | 129 | 63.61 | False | 0.00 | 0.00 |
|  | TriC_rev |  | GACGATCTGCAGGCTCT |  | 17 | 129 | 61.80 | False | 0.00 | 0.00 |
|  | TriC_detection |  | CTGGTGAACGTCATC | TTT | 15 | 129 | 54.87 | False | 0.00 | 0.00 |
|  | TriC_lnc3 | GCAGCGTGGGCTCGCGTGCCGGGGTTTTTT | GCGACGACATCTAC |  | 14 | 129 | 53.69 | False | 0.00 | 0.00 |
| mexG | mexG_fwd |  | GGACCGCAAGCTATGGC |  | 17 | 106 | 64.02 | False | 39.06 | -132.54 |
|  | mexG_rev |  | CAGCTTGGCTTCGACGC |  | 17 | 106 | 64.89 | False | 42.28 | -247.76 |
|  | mexG_lnc3 | GCAGCGTGGGCTCGCGTGCCGGGGTTTTTT | TGCTGACCATCCTC |  | 14 | 106 | 54.73 | False | 0.00 | 0.00 |
|  | mexG_detection |  | ATCGTCCACACCTT | TTT | 14 | 106 | 54.04 | False | 0.00 | 0.00 |
| mexQ | mexQ_fwd |  | ATCGTTCTTTCGGTGCTG |  | 18 | 135 | 61.29 | False | 0.00 | 0.00 |
|  | mexQ_rev |  | GATCACGTCGGGGTTGG |  | 17 | 135 | 63.19 | False | 0.00 | 0.00 |
|  | mexQ_lnc3 | GCAGCGTGGGCTCGCGTGCCGGGGTTTTTT | TTCTTCCAGTTGCC |  | 14 | 135 | 52.77 | False | 0.00 | 0.00 |
|  | mexQ_detection |  | GCTGAGCGAATACC | TTT | 14 | 135 | 53.96 | False | 0.00 | 0.00 |
| OprJ | OprJ_fwd |  | CAGCAGCTACCAGGTCG |  | 17 | 76 | 62.86 | False | 0.00 | 0.00 |
|  | OprJ_rev |  | GTCGGTCAGGCTCTTGAC |  | 18 | 76 | 63.33 | True | 48.11 | -1113.66 |
|  | OprJ_lnc3 | GCAGCGTGGGCTCGCGTGCCGGGGTTTTTT | CCTGCCGGAGTAC |  | 13 | 76 | 54.75 | False | 0.00 | 0.00 |
|  | OprJ_detection |  | GAACTGGACCTCT | TTT | 13 | 76 | 48.87 | False | 0.00 | 0.00 |
| MexB | MexB_fwd |  | CGTGGTCCAGGTGATCG |  | 17 | 80 | 62.85 | False | 0.00 | 0.00 |
|  | MexB_rev |  | TGCCGTCGGAGTTACTCT |  | 18 | 80 | 63.65 | False | 0.00 | 0.00 |
|  | MexB_lnc3 | GCAGCGTGGGCTCGCGTGCCGGGGTTTTTT | CAGATGAACGGGATC |  | 15 | 80 | 54.13 | False | 0.00 | 0.00 |
|  | MexB_detection |  | GACAATCTGCGCTA | TTT | 14 | 80 | 52.88 | False | 0.00 | 0.00 |
| mexV | mexV_fwd |  | GTTGATCATGCTCGCCG |  | 17 | 148 | 61.91 | False | 0.00 | 0.00 |
|  | mexV_rev |  | CTGCCAGGGACGCTTTTC |  | 18 | 148 | 64.68 | False | 0.00 | 0.00 |
|  | mexV_lnc3 | GCAGCGTGGGCTCGCGTGCCGGGGTTTTTT | ACTCCATCCGTCAG |  | 14 | 148 | 54.35 | False | 0.00 | 0.00 |
|  | mexV_detection |  | CAGATCGCCCTTTT | TTT | 14 | 148 | 53.04 | False | 0.00 | 0.00 |
| MuxB | MuxB_fwd |  | CTGTTCATGGGCGACGT |  | 17 | 147 | 63.12 | False | 0.00 | 0.00 |
|  | MuxB_rev |  | CTGCTGGTCCTCGTCGA |  | 17 | 147 | 64.21 | False | 0.00 | 0.00 |
|  | MuxB_detection |  | CTTCGTCTCCCTGA | TTT | 14 | 147 | 53.83 | False | 0.00 | 0.00 |
|  | MuxB_lnc3 | GCAGCGTGGGCTCGCGTGCCGGGGTTTTTT | GATCCTGATTTCCGG |  | 15 | 147 | 54.08 | False | 0.00 | 0.00 |
| TriB | TriB_fwd |  | GACGGCAAGACCCAGGT |  | 17 | 71 | 64.89 | False | 36.44 | 24.05 |
|  | TriB_rev |  | AGCACCTGTACCTCGCG |  | 17 | 71 | 64.95 | False | 0.00 | 0.00 |
|  | TriB_lnc3 | GCAGCGTGGGCTCGCGTGCCGGGGTTTTTT | TCGTCGATGGGAA |  | 13 | 71 | 52.50 | False | 0.00 | 0.00 |
|  | TriB_detection |  | ACAGTCCAGCGTG | TTT | 13 | 71 | 54.96 | False | 0.00 | 0.00 |
| CatB7 | CatB7_fwd |  | AGCTGCTCTCGGAACAG |  | 17 | 141 | 62.05 | False | 36.21 | 63.13 |
|  | CatB7_rev |  | TGACCAGCTTGTCCACGT |  | 18 | 141 | 64.88 | False | 37.96 | -70.61 |
|  | CatB7_detection |  | CCGGCTACTATCAC | TTT | 14 | 141 | 52.14 | False | 0.00 | 0.00 |
|  | CatB7_lnc3 | GCAGCGTGGGCTCGCGTGCCGGGGTTTTTT | CTACAGCTACTACT |  | 14 | 141 | 45.83 | False | 0.00 | 0.00 |
| CARB-8 | CARB-8_fwd |  | TACTGATTTTTTAAGACAAATTGGG |  | 25 | 113 | 60.75 | False | 0.00 | 0.00 |
|  | CARB-8_rev |  | TTGCCTTAGGAGTTGTCGTAT |  | 21 | 113 | 62.96 | False | 0.00 | 0.00 |
|  | CARB-8_lnc3 | GCAGCGTGGGCTCGCGTGCCGGGGTTTTTT | GCCTGATTTAAATGAAG |  | 17 | 113 | 52.01 | False | 0.00 | 0.00 |
|  | CARB-8_detection |  | GTAAGCTCGGTGATT | TTT | 15 | 113 | 53.50 | False | 0.00 | 0.00 |
| adeN | adeN_fwd |  | GATGTCCAAAATACAATTGC |  | 20 | 131 | 56.99 | False | 0.00 | 0.00 |
|  | adeN_rev |  | ATAATCATTCGCCATTCAATATC |  | 23 | 131 | 59.29 | False | 0.00 | 0.00 |
|  | adeN_detection |  | CAATCAGGTGAAATTA | TTT | 16 | 131 | 48.89 | False | 0.00 | 0.00 |
|  | adeN_lnc3 | GCAGCGTGGGCTCGCGTGCCGGGGTTTTTT | CCTTACTCATATCTCAT |  | 17 | 131 | 50.33 | False | 0.00 | 0.00 |
| msrC | msrC_fwd |  | GAGGGGAAGAACGAAAG |  | 17 | 97 | 57.33 | False | 0.00 | 0.00 |
|  | msrC_rev |  | CTTTTTCTATCTAGATGGGT |  | 20 | 97 | 55.23 | False | 35.35 | 113.51 |
|  | msrC_lnc3 | GCAGCGTGGGCTCGCGTGCCGGGGTTTTTT | TCTTATGAACAAGGG |  | 15 | 97 | 49.38 | False | 0.00 | 0.00 |
|  | msrC_detection |  | ATGCTTCTAGACGA | TTT | 14 | 97 | 49.81 | False | 0.00 | 0.00 |
| amrA | amrA_fwd |  | CTGGTCGGCGAGGACTC |  | 17 | 131 | 65.00 | False | 40.33 | -286.78 |
|  | amrA_rev |  | CCCTTCACCTGGCCTTC |  | 17 | 131 | 62.77 | False | 0.00 | 0.00 |
|  | amrA_lnc3 | GCAGCGTGGGCTCGCGTGCCGGGGTTTTTT | TCGATCCGATCTAC |  | 14 | 131 | 50.35 | False | 0.00 | 0.00 |
|  | amrA_detection |  | GTGAACTTCTCCCA | TTT | 14 | 131 | 51.37 | False | 0.00 | 0.00 |
| OpmH | OpmH_fwd |  | CCGTGCGCGACTACAAC |  | 17 | 114 | 64.51 | False | 43.72 | -394.19 |
|  | OpmH_rev |  | TCAGGTAGGCGCTGAGC |  | 17 | 114 | 64.66 | True | 47.49 | -644.38 |
|  | OpmH_lnc3 | GCAGCGTGGGCTCGCGTGCCGGGGTTTTTT | CCGCTACGACTACA |  | 14 | 114 | 54.62 | False | 0.00 | 0.00 |
|  | OpmH_detection |  | TCCTCGATACCCTG | TTT | 14 | 114 | 53.02 | False | 0.00 | 0.00 |
| OXA-50 | OXA-50_fwd |  | GTCTCGCGCCTGGGTTA |  | 17 | 140 | 64.97 | False | 0.00 | 0.00 |
|  | OXA-50_rev |  | GCGGGGAATGGCAATTCT |  | 18 | 140 | 64.15 | True | 45.89 | -900.41 |
|  | OXA-50_detection |  | GACCGCTGAAGATC | TTT | 14 | 140 | 53.44 | False | 0.00 | 0.00 |
|  | OXA-50_lnc3 | GCAGCGTGGGCTCGCGTGCCGGGGTTTTTT | CTTCTGGTTGGTGG |  | 14 | 140 | 53.36 | False | 0.00 | 0.00 |
| OpmB | OpmB_fwd |  | CTGCTCAACGACACGGT |  | 17 | 127 | 63.01 | False | 0.00 | 0.00 |
|  | OpmB_rev |  | GGGCCTGGGTGCTTTTC |  | 17 | 127 | 64.20 | False | 0.00 | 0.00 |
|  | OpmB_detection |  | GCCGAGAACAAATAC | TTT | 15 | 127 | 52.53 | False | 0.00 | 0.00 |
|  | OpmB_lnc3 | GCAGCGTGGGCTCGCGTGCCGGGGTTTTTT | GTTCGCTGAAGGTG |  | 14 | 127 | 54.58 | False | 0.00 | 0.00 |
| mexJ | mexJ_fwd |  | AGACGGTCTTCAGCCTG |  | 17 | 119 | 61.64 | False | 0.00 | 0.00 |
|  | mexJ_rev |  | GCGTTGCGACCAGAGTTC |  | 18 | 119 | 64.94 | False | 0.00 | 0.00 |
|  | mexJ_lnc3 | GCAGCGTGGGCTCGCGTGCCGGGGTTTTTT | GAACACAGCTTCGAA |  | 15 | 119 | 54.65 | False | 0.00 | 0.00 |
|  | mexJ_detection |  | CGTTTCCGCATCG | TTT | 13 | 119 | 54.71 | False | 0.00 | 0.00 |
| OprM | OprM_fwd |  | TCGATCAACCTGCCGATCT |  | 19 | 101 | 64.62 | False | 40.98 | -381.42 |
|  | OprM_rev |  | ATCGCCTTCTCGTACTG |  | 17 | 101 | 58.95 | False | 0.00 | 0.00 |
|  | OprM_detection |  | CAGAAGGACATCAAC | TTT | 15 | 101 | 51.43 | False | 0.00 | 0.00 |
|  | OprM_lnc3 | GCAGCGTGGGCTCGCGTGCCGGGGTTTTTT | ACTACGCGAAGATC |  | 14 | 101 | 52.12 | False | 0.00 | 0.00 |
| opmD | opmD_fwd |  | CTGTTCGACGAYCGCTG |  | 17 | 158 | 62.49 | False | 44.33 | -496.64 |
|  | opmD_rev |  | AGGTCGATTTCCCACTGC |  | 18 | 158 | 63.01 | False | 0.00 | 0.00 |
|  | opmD_detection |  | AGCTGGACTACGAC | TTT | 14 | 158 | 54.42 | False | 0.00 | 0.00 |
|  | opmD_lnc3 | GCAGCGTGGGCTCGCGTGCCGGGGTTTTTT | CAGCATCGAACAAC |  | 14 | 158 | 52.15 | False | 0.00 | 0.00 |
| mexI | mexI_fwd |  | GACCATCCGCATGCTGC |  | 17 | 135 | 64.73 | False | 42.89 | -374.46 |
|  | mexI_rev |  | GTGGTCTCGCCGGAAGA |  | 17 | 135 | 64.56 | False | 43.71 | -375.02 |
|  | mexI_detection |  | GCCAAGGTCAACTC | TTT | 14 | 135 | 53.95 | False | 0.00 | 0.00 |
|  | mexI_lnc3 | GCAGCGTGGGCTCGCGTGCCGGGGTTTTTT | TGACCGAGACCATG |  | 14 | 135 | 54.80 | False | 0.00 | 0.00 |
| arnA | arnA_fwd |  | TCGAGTACACGCGCAAC |  | 17 | 117 | 63.43 | False | 42.37 | -444.39 |
|  | arnA_rev |  | CCTCGGAGGTGGAAGGG |  | 17 | 117 | 64.32 | False | 0.00 | 0.00 |
|  | arnA_detection |  | GAGGAAAACCTGCG | TTT | 14 | 117 | 54.53 | False | 0.00 | 0.00 |
|  | arnA_lnc3 | GCAGCGTGGGCTCGCGTGCCGGGGTTTTTT | TTCGAACTGGACTTC |  | 15 | 117 | 53.32 | False | 0.00 | 0.00 |
| amrB | amrB_fwd |  | TCGATCAGGAAGGTGGTC |  | 18 | 148 | 61.64 | False | 0.00 | 0.00 |
|  | amrB_rev |  | CGAGCATCACCGTGAAGG |  | 18 | 148 | 64.08 | True | 48.36 | -717.13 |
|  | amrB_lnc3 | GCAGCGTGGGCTCGCGTGCCGGGGTTTTTT | CCGTGATGTACCTG |  | 14 | 148 | 52.88 | False | 0.00 | 0.00 |
|  | amrB_detection |  | TTCATGCAGAACTTC | TTT | 15 | 148 | 51.27 | False | 0.00 | 0.00 |
| DfrA12 | DfrA12_fwd |  | TAGTTGTTTCAACGCTGTCG |  | 20 | 152 | 62.63 | False | 42.43 | -374.84 |
|  | DfrA12_rev |  | GTCACCCTCGAAGGTTTGAT |  | 20 | 152 | 63.66 | False | 43.79 | -348.98 |
|  | DfrA12_lnc3 | GCAGCGTGGGCTCGCGTGCCGGGGTTTTTT | CGGAGCTGAGATATA |  | 15 | 152 | 51.19 | False | 0.00 | 0.00 |
|  | DfrA12_detection |  | CACTCTGGCACTAC | TTT | 14 | 152 | 52.75 | False | 0.00 | 0.00 |
| farA | farA_fwd |  | GGCATATGAAAATCGGAC |  | 18 | 124 | 56.78 | False | 0.00 | 0.00 |
|  | farA_rev |  | GCCGGAATCAGCGAAAA |  | 17 | 124 | 61.37 | False | 0.00 | 0.00 |
|  | farA_detection |  | TACGGCAAACAAAT | TTT | 14 | 124 | 49.66 | False | 0.00 | 0.00 |
|  | farA_lnc3 | GCAGCGTGGGCTCGCGTGCCGGGGTTTTTT | TGGTGTCCGATTTG |  | 14 | 124 | 52.97 | False | 0.00 | 0.00 |
| Aph3-IIb | Aph3-IIb_fwd |  | CTGCCCGCCGAAATCG |  | 16 | 107 | 64.17 | False | 39.44 | -164.12 |
|  | Aph3-IIb_rev |  | CTCATCAGCAGCCATTG |  | 17 | 107 | 58.61 | False | 0.00 | 0.00 |
|  | Aph3-IIb_detection |  | AACCCAGAGCGAC | TTT | 13 | 107 | 54.46 | False | 0.00 | 0.00 |
|  | Aph3-IIb_lnc3 | GCAGCGTGGGCTCGCGTGCCGGGGTTTTTT | AGGTGCTGAACGA |  | 13 | 107 | 53.27 | False | 0.00 | 0.00 |
| VIM-2 | VIM-2_fwd |  | ATTGTCCGTGATGGTGA |  | 17 | 119 | 58.65 | False | 41.81 | -293.26 |
|  | VIM-2_rev |  | GAGACTGCACGCGTTAC |  | 17 | 119 | 61.41 | False | 0.00 | 0.00 |
|  | VIM-2_lnc3 | GCAGCGTGGGCTCGCGTGCCGGGGTTTTTT | GATTGATACAGCGTG |  | 15 | 119 | 52.28 | False | 0.00 | 0.00 |
|  | VIM-2_detection |  | GGGTGCGAAAAACA | TTT | 14 | 119 | 54.67 | False | 0.00 | 0.00 |
| MuxC | MuxC_fwd |  | CGACCATCTTCAACCCGC |  | 18 | 99 | 64.40 | False | 0.00 | 0.00 |
|  | MuxC_rev |  | CGATCACCTGGACCTGG |  | 17 | 99 | 62.15 | False | 0.00 | 0.00 |
|  | MuxC_lnc3 | GCAGCGTGGGCTCGCGTGCCGGGGTTTTTT | TGGTGATGGAGGTC |  | 14 | 99 | 54.14 | False | 0.00 | 0.00 |
|  | MuxC_detection |  | GACCAGCAGTACCA | TTT | 14 | 99 | 54.64 | False | 0.00 | 0.00 |
| opmE | opmE_fwd |  | GAGGTACGCGGCTCGAT |  | 17 | 95 | 64.74 | False | 44.20 | -399.08 |
|  | opmE_rev |  | CAGCGAATCACCGGGTT |  | 17 | 95 | 63.14 | False | 0.00 | 0.00 |
|  | opmE_detection |  | CGAGAGTGGTACGT | TTT | 14 | 95 | 54.50 | False | 0.00 | 0.00 |
|  | opmE_lnc3 | GCAGCGTGGGCTCGCGTGCCGGGGTTTTTT | CTCGATGCTTTGGA |  | 14 | 95 | 52.69 | False | 0.00 | 0.00 |
| basS | basS_fwd |  | AGATCTGGATCAGCGAAAA |  | 19 | 123 | 59.94 | False | 43.66 | -540.63 |
|  | basS_rev |  | CAACGCCGAACCAGACC |  | 17 | 123 | 64.14 | False | 0.00 | 0.00 |
|  | basS_detection |  | CTGCTGCTGTTCTA | TTT | 14 | 123 | 52.14 | False | 0.00 | 0.00 |
|  | basS_lnc3 | GCAGCGTGGGCTCGCGTGCCGGGGTTTTTT | AGCACACCATGAAC |  | 14 | 123 | 53.36 | False | 0.00 | 0.00 |
| MexD | MexD_fwd |  | CTGGTGGACTCCGTCAC |  | 17 | 114 | 62.38 | False | 37.83 | -54.91 |
|  | MexD_rev |  | GAAGGTGACGACGATCTC |  | 18 | 114 | 60.42 | False | 0.00 | 0.00 |
|  | MexD_detection |  | GAGTCGACCAACAA | TTT | 14 | 114 | 52.52 | False | 0.00 | 0.00 |
|  | MexD_lnc3 | GCAGCGTGGGCTCGCGTGCCGGGGTTTTTT | GCCTGCTCTACTTC |  | 14 | 114 | 52.84 | False | 0.00 | 0.00 |
| AphA6 | AphA6_fwd |  | CGAAAATGTTGAGTTGGCTC |  | 20 | 107 | 61.50 | False | 43.63 | -561.31 |
|  | AphA6_rev |  | TTTTGCATTGATCGCTTT |  | 18 | 107 | 57.05 | False | 0.00 | 0.00 |
|  | AphA6_detection |  | CTCATCATGACTTTTC | TTT | 16 | 107 | 50.06 | False | 0.00 | 0.00 |
|  | AphA6_lnc3 | GCAGCGTGGGCTCGCGTGCCGGGGTTTTTT | TTAAAGGTGCCTGAA |  | 15 | 107 | 52.47 | False | 0.00 | 0.00 |
| mexL | mexL_fwd |  | AAGCCATCGCCTCGGAA |  | 17 | 127 | 64.64 | False | 37.49 | -22.95 |
|  | mexL_rev |  | TGGAAGTACAGGGCGGG |  | 17 | 127 | 64.25 | False | 0.00 | 0.00 |
|  | mexL_lnc3 | GCAGCGTGGGCTCGCGTGCCGGGGTTTTTT | CTACAGCCACTTCAC |  | 15 | 127 | 54.33 | False | 0.00 | 0.00 |
|  | mexL_detection |  | CGACAAGGAAACCC | TTT | 14 | 127 | 53.97 | False | 0.00 | 0.00 |

### ABR-5

Supplementary Table S8: Primer and probe sequences and additional information of ABR-5.

| Gene | Primer/Probe | Hybridisation Sequence | Sequence | Spacer | Length | Product Size | Tm | Hairpin | Hairpin Tm | Hairpin delta G |
| --- | --- | --- | --- | --- | --- | --- | --- | --- | --- | --- |
| Str | Str_fwd |  | TAATATTTTACGTCCTGAATTATT |  | 24 | 130 | 56.43 | False | 0.00 | 0.00 |
|  | Str_rev |  | CATATTGAATTCTTTATCAGTTAAA |  | 25 | 130 | 56.37 | False | 0.00 | 0.00 |
|  | Str_lnc3 | GCAGCGTGGGCTCGCGTGCCGGGGTTTTTT | TTCTTGGTATATTGGCT |  | 17 | 130 | 53.77 | False | 0.00 | 0.00 |
|  | Str_detection |  | TTAATAGGGGTTTTGATTT | TTT | 19 | 130 | 53.80 | False | 0.00 | 0.00 |
| FosX | FosX_fwd |  | CTTTAATTGTGAAAGATTTGAAT |  | 23 | 151 | 56.15 | False | 40.91 | -344.77 |
|  | FosX_rev |  | CCTTCCATAATGCAAATCC |  | 19 | 151 | 57.48 | False | 0.00 | 0.00 |
|  | FosX_lnc3 | GCAGCGTGGGCTCGCGTGCCGGGGTTTTTT | AATGCAGAAGAAATC |  | 15 | 151 | 48.40 | False | 0.00 | 0.00 |
|  | FosX_detection |  | TATTCTAGTGGCGA | TTT | 14 | 151 | 49.32 | False | 0.00 | 0.00 |
| efmA | efmA_fwd |  | TTTCTCCTGAAATATTGGAAGTT |  | 23 | 114 | 60.18 | False | 41.96 | -439.32 |
|  | efmA_rev |  | CAAATGCACCATTGATCGTTAT |  | 22 | 114 | 61.85 | False | 39.43 | -185.18 |
|  | efmA_detection |  | CTGCAGCAATTGAT | TTT | 14 | 114 | 50.91 | False | 0.00 | 0.00 |
|  | efmA_lnc3 | GCAGCGTGGGCTCGCGTGCCGGGGTTTTTT | GATGCAAAATTTGGG |  | 15 | 114 | 51.06 | False | 0.00 | 0.00 |
| VanA-A | VanA-A_fwd |  | TATAACCGTTCCCGCAGAC |  | 19 | 125 | 62.97 | False | 0.00 | 0.00 |
|  | VanA-A_rev |  | CGTTATCTTGTAAAAACATATCCA |  | 24 | 125 | 59.62 | False | 0.00 | 0.00 |
|  | VanA-A_detection |  | ACGGCAAAAAAAATAT | TTT | 16 | 125 | 50.38 | False | 0.00 | 0.00 |
|  | VanA-A_lnc3 | GCAGCGTGGGCTCGCGTGCCGGGGTTTTTT | GACGGATACAGGAA |  | 14 | 125 | 50.83 | False | 0.00 | 0.00 |
| catB8 | catB8_fwd |  | CATTTGATGAATGCGCGC |  | 18 | 114 | 61.87 | False | 43.61 | -572.02 |
|  | catB8_rev |  | GATTGCCAGCCATGATGAA |  | 19 | 114 | 62.01 | False | 0.00 | 0.00 |
|  | catB8_lnc3 | GCAGCGTGGGCTCGCGTGCCGGGGTTTTTT | GACGTTGATAAATTGATC |  | 18 | 114 | 52.94 | False | 0.00 | 0.00 |
|  | catB8_detection |  | ATTGGCAGCTTTTGT | TTT | 15 | 114 | 54.72 | False | 0.00 | 0.00 |
| emrK | emrK_fwd |  | GGGTTATTTCAAAAGAARCGCT |  | 22 | 129 | 62.94 | False | 43.40 | -681.43 |
|  | emrK_rev |  | GACGGTTTAATGGTGTGTTCAT |  | 22 | 129 | 63.37 | False | 0.00 | 0.00 |
|  | emrK_detection |  | ATAAGTAGCAAAGCG | TTT | 15 | 129 | 51.16 | False | 0.00 | 0.00 |
|  | emrK_lnc3 | GCAGCGTGGGCTCGCGTGCCGGGGTTTTTT | TACCAAAGATACGTTA |  | 16 | 129 | 49.18 | False | 0.00 | 0.00 |
| mexH | mexH_fwd |  | CACCTCGGCCAGTACCT |  | 17 | 139 | 63.89 | False | 0.00 | 0.00 |
|  | mexH_rev |  | CGTCGACCAGGACCTCG |  | 17 | 139 | 64.95 | False | 37.07 | -4.82 |
|  | mexH_lnc3 | GCAGCGTGGGCTCGCGTGCCGGGGTTTTTT | AGCAATTTCTCCCTG |  | 15 | 139 | 53.65 | False | 0.00 | 0.00 |
|  | mexH_detection |  | GACGAAAGCACCAG | TTT | 14 | 139 | 54.58 | False | 0.00 | 0.00 |
| FosA | FosA_fwd |  | CTCAATCACCTGACCCTG |  | 18 | 114 | 60.27 | False | 0.00 | 0.00 |
|  | FosA_rev |  | CAGTTCGAGATAGGCGC |  | 17 | 114 | 60.53 | False | 0.00 | 0.00 |
|  | FosA_detection |  | TACCGCGATCTTCT | TTT | 14 | 114 | 53.54 | False | 0.00 | 0.00 |
|  | FosA_lnc3 | GCAGCGTGGGCTCGCGTGCCGGGGTTTTTT | CAGCATCGCCTTC |  | 13 | 114 | 53.57 | False | 0.00 | 0.00 |
| DfrG | DfrG_fwd |  | GAATTATGAGGAATGGAATGAGGT |  | 24 | 106 | 62.85 | False | 0.00 | 0.00 |
|  | DfrG_rev |  | ACTCAATAAGTTTTTTCTTTCA |  | 22 | 106 | 56.28 | False | 39.21 | -97.57 |
|  | DfrG_lnc3 | GCAGCGTGGGCTCGCGTGCCGGGGTTTTTT | AATGATAAAAATCCG |  | 15 | 106 | 45.19 | False | 0.00 | 0.00 |
|  | DfrG_detection |  | TATAACTACTATTTTCAT | TTT | 18 | 106 | 45.52 | False | 0.00 | 0.00 |
| catB3 | catB3_fwd |  | GCATCATCTTTCCCGTTCT |  | 19 | 154 | 61.32 | False | 0.00 | 0.00 |
|  | catB3_rev |  | CACCGTGCCCGATCTTG |  | 17 | 154 | 63.91 | False | 38.36 | -68.44 |
|  | catB3_detection |  | AATACTGTCATTGGC | TTT | 15 | 154 | 50.89 | False | 0.00 | 0.00 |
|  | catB3_lnc3 | GCAGCGTGGGCTCGCGTGCCGGGGTTTTTT | TCCAAAAAGCAGGT |  | 14 | 154 | 51.97 | False | 0.00 | 0.00 |
| cdeA | cdeA_fwd |  | TTTATGTACRCCATTTTATATTTT |  | 24 | 157 | 56.49 | False | 38.52 | -113.94 |
|  | cdeA_rev |  | TAAAAGACCCATTCCAAAATATTTA |  | 25 | 157 | 59.49 | False | 0.00 | 0.00 |
|  | cdeA_detection |  | GTCATTGGTGGTGTAA | TTT | 16 | 157 | 54.71 | False | 0.00 | 0.00 |
|  | cdeA_lnc3 | GCAGCGTGGGCTCGCGTGCCGGGGTTTTTT | AATTTAGTTTATTTCTATCA |  | 20 | 157 | 49.40 | False | 0.00 | 0.00 |
| VanH-A | VanH-A_fwd |  | GAACAAATACAGAGAATGAAGCAAG |  | 25 | 136 | 63.12 | False | 0.00 | 0.00 |
|  | VanH-A_rev |  | CTCCTTCCAATACATCCAATGC |  | 22 | 136 | 63.32 | False | 0.00 | 0.00 |
|  | VanH-A_lnc3 | GCAGCGTGGGCTCGCGTGCCGGGGTTTTTT | TATCAATACTGGGCG |  | 15 | 136 | 52.76 | False | 0.00 | 0.00 |
|  | VanH-A_detection |  | CGGTCCACTTGTAG | TTT | 14 | 136 | 52.78 | False | 0.00 | 0.00 |
| Listeria | Listeria_fwd |  | TGGTTTCAAAAAAATAGTACC |  | 21 | 133 | 56.22 | False | 0.00 | 0.00 |
|  | Listeria_rev |  | TTTGAGAAGTTAAATTTTCTTTTA |  | 24 | 133 | 55.83 | False | 38.90 | -158.30 |
|  | Listeria_lnc3 | GCAGCGTGGGCTCGCGTGCCGGGGTTTTTT | TGTTATTTTTGAAATTAT |  | 18 | 133 | 46.18 | False | 0.00 | 0.00 |
|  | Listeria_detection |  | TAATATTGCGACGG | TTT | 14 | 133 | 49.53 | False | 0.00 | 0.00 |
| BLA1 | BLA1_fwd |  | AGAAAAAATTTGATGCTCGAK |  | 21 | 115 | 58.79 | False | 0.00 | 0.00 |
|  | BLA1_rev |  | GCTAAAGCCTTGTARGTTG |  | 19 | 115 | 58.66 | False | 42.28 | -326.53 |
|  | BLA1_detection |  | GGTACAAATCAAACAAT | TTT | 17 | 115 | 51.29 | False | 0.00 | 0.00 |
|  | BLA1_lnc3 | GCAGCGTGGGCTCGCGTGCCGGGGTTTTTT | ATATGCGATTGATACT |  | 16 | 115 | 49.93 | False | 0.00 | 0.00 |
| AAC(3)-IIa | AAC(3)-IIa_fwd |  | CGGCATTCTCGATTGCTTT |  | 19 | 129 | 62.64 | False | 41.30 | -496.28 |
|  | AAC(3)-IIa_rev |  | GCGTCGAACAGGTAGCA |  | 17 | 129 | 62.76 | False | 0.00 | 0.00 |
|  | AAC(3)-IIa_lnc3 | GCAGCGTGGGCTCGCGTGCCGGGGTTTTTT | GAAACTATAGCAAATG |  | 16 | 129 | 47.46 | False | 0.00 | 0.00 |
|  | AAC(3)-IIa_detection |  | CTTACGTGAAGCTC | TTT | 14 | 129 | 50.40 | False | 0.00 | 0.00 |
| VanX-A | VanX-A_fwd |  | GACACGGGTGAGCTTGTA |  | 18 | 123 | 62.58 | False | 0.00 | 0.00 |
|  | VanX-A_rev |  | CATGATGGAGCGCAAAC |  | 17 | 123 | 59.71 | False | 0.00 | 0.00 |
|  | VanX-A_detection |  | AACGCTCTCATCAT | TTT | 14 | 123 | 51.19 | False | 0.00 | 0.00 |
|  | VanX-A_lnc3 | GCAGCGTGGGCTCGCGTGCCGGGGTTTTTT | TTTGATTTTATGGATG |  | 16 | 123 | 46.57 | False | 0.00 | 0.00 |
| tetO | tetO_fwd |  | CAAATCCTTTCTGGGCTTC |  | 19 | 123 | 59.94 | False | 38.37 | -86.86 |
|  | tetO_rev |  | CCATAACCGCATTTTGGA |  | 18 | 123 | 58.89 | True | 46.62 | -926.35 |
|  | tetO_detection |  | TTCACTTGGATATTTAAA | TTT | 18 | 123 | 50.04 | False | 0.00 | 0.00 |
|  | tetO_lnc3 | GCAGCGTGGGCTCGCGTGCCGGGGTTTTTT | AGTATGAAAGCAGAGT |  | 16 | 123 | 52.66 | False | 0.00 | 0.00 |
| mexM | mexM_fwd |  | TCGAGCAACAGGTCAGC |  | 17 | 79 | 62.71 | False | 0.00 | 0.00 |
|  | mexM_rev |  | AACTGGCCGTCGATCTG |  | 17 | 79 | 62.46 | False | 0.00 | 0.00 |
|  | mexM_detection |  | CAACGTGGTGATCC | TTT | 14 | 79 | 54.13 | False | 0.00 | 0.00 |
|  | mexM_lnc3 | GCAGCGTGGGCTCGCGTGCCGGGGTTTTTT | GTGACTTCGTTGCA |  | 14 | 79 | 53.94 | False | 0.00 | 0.00 |
| ACT-24 | ACT-24_fwd |  | GTTGCYGATGCCTCACT |  | 17 | 101 | 61.67 | False | 40.99 | -242.88 |
|  | ACT-24_rev |  | CAGTTGAGCATCTCCCAGC |  | 19 | 101 | 64.25 | False | 38.06 | -46.44 |
|  | ACT-24_lnc3 | GCAGCGTGGGCTCGCGTGCCGGGGTTTTTT | GCTACTGGCGTATC |  | 14 | 101 | 53.15 | False | 0.00 | 0.00 |
|  | ACT-24_detection |  | GGGTCAATGTATCAG | TTT | 15 | 101 | 50.66 | False | 0.00 | 0.00 |
| CTX-M-18 | CTX-M-18_fwd |  | GCCGCGTTGCAGTACAG |  | 17 | 110 | 64.88 | False | 0.00 | 0.00 |
|  | CTX-M-18_rev |  | CGAAACGTCTCATCGCC |  | 17 | 110 | 61.49 | False | 0.00 | 0.00 |
|  | CTX-M-18_detection |  | AAATTGATTGCCCAG | TTT | 15 | 110 | 52.01 | False | 0.00 | 0.00 |
|  | CTX-M-18_lnc3 | GCAGCGTGGGCTCGCGTGCCGGGGTTTTTT | ATACCGCCATGAAC |  | 14 | 110 | 52.74 | False | 0.00 | 0.00 |
| OprN | OprN_fwd |  | AAGCCCTACGACCGCA |  | 16 | 174 | 63.65 | False | 0.00 | 0.00 |
|  | OprN_rev |  | CGGGAAGCGATCGTTG |  | 16 | 174 | 60.82 | False | 0.00 | 0.00 |
|  | OprN_detection |  | GAAACAGTTCGACG | TTT | 14 | 174 | 51.75 | False | 0.00 | 0.00 |
|  | OprN_lnc3 | GCAGCGTGGGCTCGCGTGCCGGGGTTTTTT | GAAAGCCTGTGGTG |  | 14 | 174 | 54.37 | False | 0.00 | 0.00 |
| hp1184 | hp1184_fwd |  | GCGTATGAATTGCTTTTTGGTT |  | 22 | 113 | 62.82 | False | 0.00 | 0.00 |
|  | hp1184_rev |  | GCGACAAAATAAAACACGC |  | 19 | 113 | 60.02 | False | 39.56 | -232.18 |
|  | hp1184_detection |  | TAGGGCTAGGCTTG | TTT | 14 | 113 | 53.82 | False | 0.00 | 0.00 |
|  | hp1184_lnc3 | GCAGCGTGGGCTCGCGTGCCGGGGTTTTTT | GTCAAAATAAAACCCA |  | 16 | 113 | 49.91 | False | 0.00 | 0.00 |
| MuxA | MuxA_fwd |  | CACGCTGATGCAGAACC |  | 17 | 107 | 61.76 | False | 42.01 | -294.19 |
|  | MuxA_rev |  | GGGTATCCAGGGTCTGC |  | 17 | 107 | 61.84 | False | 0.00 | 0.00 |
|  | MuxA_detection |  | GAGATCGACCTGCA | TTT | 14 | 107 | 54.94 | False | 0.00 | 0.00 |
|  | MuxA_lnc3 | GCAGCGTGGGCTCGCGTGCCGGGGTTTTTT | AACTGAAGAACGCC |  | 14 | 107 | 53.44 | False | 0.00 | 0.00 |
| AadB | AadB_fwd |  | TTGAAATGCTCGGCGGG |  | 17 | 124 | 63.88 | False | 0.00 | 0.00 |
|  | AadB_rev |  | GCCTCCGCGATTTCATAC |  | 18 | 124 | 61.90 | False | 0.00 | 0.00 |
|  | AadB_lnc3 | GCAGCGTGGGCTCGCGTGCCGGGGTTTTTT | GAGTTGGACTATGGA |  | 15 | 124 | 51.55 | False | 0.00 | 0.00 |
|  | AadB_detection |  | TTCTTAGCGGAGAT | TTT | 14 | 124 | 49.71 | False | 0.00 | 0.00 |
| TetR(G) | TetR(G)_fwd |  | TCAGTCACTATGTGGTCG |  | 18 | 104 | 59.22 | False | 37.63 | -45.89 |
|  | TetR(G)_rev |  | GAAGGACGACGGTGCTTG |  | 18 | 104 | 64.30 | True | 58.20 | -1996.27 |
|  | TetR(G)_lnc3 | GCAGCGTGGGCTCGCGTGCCGGGGTTTTTT | CATCTGATGCCGAT |  | 14 | 104 | 52.44 | False | 0.00 | 0.00 |
|  | TetR(G)_detection |  | GAGAGAGTTCCGGA | TTT | 14 | 104 | 53.41 | False | 0.00 | 0.00 |
| hp1181 | hp1181_fwd |  | CATGCAATCTTTAGCGTC |  | 18 | 105 | 57.45 | False | 0.00 | 0.00 |
|  | hp1181_rev |  | CTCACGCCCCCAACAAA |  | 17 | 105 | 63.35 | False | 0.00 | 0.00 |
|  | hp1181_detection |  | TTAGGGCAATTCAC | TTT | 14 | 105 | 49.29 | False | 0.00 | 0.00 |
|  | hp1181_lnc3 | GCAGCGTGGGCTCGCGTGCCGGGGTTTTTT | AAAAAGGCAAGGTT |  | 14 | 105 | 49.62 | False | 0.00 | 0.00 |
| OXA-59 | OXA-59_fwd |  | AGAACAACGGCCTCGATC |  | 18 | 146 | 63.04 | False | 42.05 | -362.24 |
|  | OXA-59_rev |  | CGATTCGACGATCCGCT |  | 17 | 146 | 62.65 | False | 37.15 | -9.89 |
|  | OXA-59_detection |  | GAATTCCTCGGCAA | TTT | 14 | 146 | 53.06 | False | 0.00 | 0.00 |
|  | OXA-59_lnc3 | GCAGCGTGGGCTCGCGTGCCGGGGTTTTTT | CGCTCGAACAATTG |  | 14 | 146 | 52.74 | False | 0.00 | 0.00 |
| robA | robA_fwd |  | CACTGGATAATGTGGCGG |  | 18 | 80 | 61.45 | False | 0.00 | 0.00 |
|  | robA_rev |  | AGCATGACCGGTGACAT |  | 17 | 80 | 61.67 | False | 0.00 | 0.00 |
|  | robA_lnc3 | GCAGCGTGGGCTCGCGTGCCGGGGTTTTTT | GCTATTCCAAGTGG |  | 14 | 80 | 50.10 | False | 0.00 | 0.00 |
|  | robA_detection |  | CATCTGCAAAGGAT | TTT | 14 | 80 | 49.41 | False | 0.00 | 0.00 |
| Cat-pC194 | Cat-pC194_fwd |  | GGAAGAGAAAAGAGATATTTAATC |  | 24 | 92 | 57.35 | False | 0.00 | 0.00 |
|  | Cat-pC194_rev |  | GTTTCGGTATAAAACACTAATATC |  | 24 | 92 | 57.93 | False | 40.73 | -389.32 |
|  | Cat-pC194_detection |  | TTTTAGTATAACCACAG | TTT | 17 | 92 | 49.05 | False | 0.00 | 0.00 |
|  | Cat-pC194_lnc3 | GCAGCGTGGGCTCGCGTGCCGGGGTTTTTT | AACCAACAAACGAC |  | 14 | 92 | 51.13 | False | 0.00 | 0.00 |
| PENA | PENA_fwd |  | TTCGTCCTGATGGCAAT |  | 17 | 120 | 59.08 | False | 0.00 | 0.00 |
|  | PENA_rev |  | TTGAGTCCGCACAATCC |  | 17 | 120 | 60.20 | False | 0.00 | 0.00 |
|  | PENA_lnc3 | GCAGCGTGGGCTCGCGTGCCGGGGTTTTTT | CTGCAGACGGTAAC |  | 14 | 120 | 53.77 | False | 0.00 | 0.00 |
|  | PENA_detection |  | GTATAACTTTTTGAAAGA | TTT | 18 | 120 | 48.89 | False | 0.00 | 0.00 |
| AAC(3)-Ia | AAC(3)-Ia_fwd |  | GCATCATTCGCACATGTAG |  | 19 | 139 | 60.54 | False | 0.00 | 0.00 |
|  | AAC(3)-Ia_rev |  | AGCAAGTTCCCGAGGTAAT |  | 19 | 139 | 62.20 | False | 0.00 | 0.00 |
|  | AAC(3)-Ia_detection |  | TTTTCGGTCGTGAG | TTT | 14 | 139 | 53.12 | False | 0.00 | 0.00 |
|  | AAC(3)-Ia_lnc3 | GCAGCGTGGGCTCGCGTGCCGGGGTTTTTT | GGCTGCTCTTGATC |  | 14 | 139 | 53.77 | False | 0.00 | 0.00 |
| norB | norB_fwd |  | CTGGCGCTGGATTTTCATTATT |  | 22 | 145 | 63.84 | False | 0.00 | 0.00 |
|  | norB_rev |  | AACCATTGCGATAACAAAAA |  | 20 | 145 | 58.26 | False | 36.38 | 41.24 |
|  | norB_detection |  | AAGTCGTTCAAAATAC | TTT | 16 | 145 | 49.87 | False | 0.00 | 0.00 |
|  | norB_lnc3 | GCAGCGTGGGCTCGCGTGCCGGGGTTTTTT | GTACTCCAGAAAGTA |  | 15 | 145 | 48.17 | False | 0.00 | 0.00 |
| armA | armA_fwd |  | GGTCTTACTATTCTGCCTATC |  | 21 | 120 | 58.75 | False | 0.00 | 0.00 |
|  | armA_rev |  | ATGTTGCGACTCTTTCATTCG |  | 21 | 120 | 63.26 | False | 37.55 | -43.83 |
|  | armA_detection |  | AGTTATCAATAGAAGATT | TTT | 18 | 120 | 48.72 | False | 0.00 | 0.00 |
|  | armA_lnc3 | GCAGCGTGGGCTCGCGTGCCGGGGTTTTTT | GTACAATCAGGGGC |  | 14 | 120 | 53.16 | False | 0.00 | 0.00 |
| lmrP | lmrP_fwd |  | ATTGATTTGACTGATGAGAGT |  | 21 | 87 | 58.72 | False | 0.00 | 0.00 |
|  | lmrP_rev |  | CCCTGCACCAAACATGAC |  | 18 | 87 | 62.26 | False | 0.00 | 0.00 |
|  | lmrP_lnc3 | GCAGCGTGGGCTCGCGTGCCGGGGTTTTTT | GATTTGTTTACACCAT |  | 16 | 87 | 49.67 | False | 0.00 | 0.00 |
|  | lmrP_detection |  | CAATTATTGGTTTATCAA | TTT | 18 | 87 | 49.31 | False | 0.00 | 0.00 |
| cmeR | cmeR_fwd |  | CTTTGAAATTTTAGATGACATATGT |  | 25 | 139 | 58.62 | False | 0.00 | 0.00 |
|  | cmeR_rev |  | TTCTGGTTGATTRAAAATTTCT |  | 22 | 139 | 57.48 | False | 0.00 | 0.00 |
|  | cmeR_detection |  | AAAACACAAGAAATT | TTT | 15 | 139 | 45.24 | False | 0.00 | 0.00 |
|  | cmeR_lnc3 | GCAGCGTGGGCTCGCGTGCCGGGGTTTTTT | TTCATCTTATTTATTCC |  | 17 | 139 | 46.53 | False | 0.00 | 0.00 |
| vanRA | vanRA_fwd |  | AATTGCCGATTTGGTTGA |  | 18 | 134 | 58.72 | False | 43.71 | -375.02 |
|  | vanRA_rev |  | CGGGAAGCATGATRTCCA |  | 18 | 134 | 61.43 | False | 38.51 | -112.43 |
|  | vanRA_detection |  | CTATACCGCCAAAG | TTT | 14 | 134 | 49.90 | False | 0.00 | 0.00 |
|  | vanRA_lnc3 | GCAGCGTGGGCTCGCGTGCCGGGGTTTTTT | TACGGTTTTCAAATA |  | 15 | 134 | 46.39 | False | 0.00 | 0.00 |
| OXA-1 | OXA-1_fwd |  | ACGGATGGTTTGAAGGGT |  | 18 | 114 | 62.17 | False | 0.00 | 0.00 |
|  | OXA-1_rev |  | TTTTCTTGGCTTTTATGCTTGATG |  | 24 | 114 | 63.16 | False | 0.00 | 0.00 |
|  | OXA-1_lnc3 | GCAGCGTGGGCTCGCGTGCCGGGGTTTTTT | AGCAAATCAGGACATA |  | 16 | 114 | 53.09 | False | 0.00 | 0.00 |
|  | OXA-1_detection |  | AATATGTTTTTGTGTCC | TTT | 17 | 114 | 51.29 | False | 0.00 | 0.00 |
| TetW | TetW_fwd |  | TTTTCAAAACGCTGTCA |  | 17 | 158 | 55.56 | False | 0.00 | 0.00 |
|  | TetW_rev |  | CCAATACAATCGGGGCCAA |  | 19 | 158 | 63.96 | False | 0.00 | 0.00 |
|  | TetW_detection |  | AGATTTGCTTTGAATAC | TTT | 17 | 158 | 51.06 | False | 0.00 | 0.00 |
|  | TetW_lnc3 | GCAGCGTGGGCTCGCGTGCCGGGGTTTTTT | ACGTAACGGACTGTA |  | 15 | 158 | 54.54 | False | 0.00 | 0.00 |
| QnrB19 | QnrB19_fwd |  | CGTTCAGTGGTTCAGATCT |  | 19 | 137 | 60.55 | False | 0.00 | 0.00 |
|  | QnrB19_rev |  | CTTAACGCCTTGTAAATCA |  | 19 | 137 | 56.67 | False | 0.00 | 0.00 |
|  | QnrB19_lnc3 | GCAGCGTGGGCTCGCGTGCCGGGGTTTTTT | AACTTCACACATTGC |  | 15 | 137 | 52.47 | False | 0.00 | 0.00 |
|  | QnrB19_detection |  | GATCTGACCAATTCG | TTT | 15 | 137 | 51.72 | False | 0.00 | 0.00 |
| TetR | TetR_fwd |  | AGACGCCTTCGACGAAG |  | 17 | 127 | 62.42 | False | 42.28 | -473.34 |
|  | TetR_rev |  | TCAATCGTCACCCTTTCTCG |  | 20 | 127 | 63.68 | False | 0.00 | 0.00 |
|  | TetR_detection |  | TTGGCGAAAAGGAG | TTT | 14 | 127 | 53.38 | False | 0.00 | 0.00 |
|  | TetR_lnc3 | GCAGCGTGGGCTCGCGTGCCGGGGTTTTTT | GTGATTGTCGATGGA |  | 15 | 127 | 53.51 | False | 0.00 | 0.00 |
| DfrK | DfrK_fwd |  | AGAGGAAATTTTCATTTTCGG |  | 21 | 128 | 58.75 | False | 39.63 | -233.11 |
|  | DfrK_rev |  | CATAATTCACTTCTGGAAAG |  | 20 | 128 | 55.36 | False | 0.00 | 0.00 |
|  | DfrK_lnc3 | GCAGCGTGGGCTCGCGTGCCGGGGTTTTTT | ATATGTTGAGAAAATGTAC |  | 19 | 128 | 52.21 | False | 0.00 | 0.00 |
|  | DfrK_detection |  | ATTACAAAAATTCATTACG | TTT | 19 | 128 | 51.32 | False | 0.00 | 0.00 |
| vanYA | vanYA_fwd |  | TCTCGAGGAATATATGGATTA |  | 21 | 117 | 56.56 | False | 0.00 | 0.00 |
|  | vanYA_rev |  | GGCACATGAATGGTGGT |  | 17 | 117 | 60.55 | False | 40.11 | -256.80 |
|  | vanYA_detection |  | TTTTATTATCCTGTTAC | TTT | 17 | 117 | 46.49 | False | 0.00 | 0.00 |
|  | vanYA_lnc3 | GCAGCGTGGGCTCGCGTGCCGGGGTTTTTT | GGAAAAATATGAGATC |  | 16 | 117 | 46.56 | False | 0.00 | 0.00 |
| FosA2 | FosA2_fwd |  | TTTCTCGACCCGGACGG |  | 17 | 67 | 64.55 | False | 37.81 | -42.69 |
|  | FosA2_rev |  | ACGCCGCCAGCCG |  | 13 | 67 | 64.80 | False | 41.78 | -221.45 |
|  | FosA2_lnc3 | GCAGCGTGGGCTCGCGTGCCGGGGTTTTTT | GCTGGAGCTGCA |  | 12 | 67 | 54.19 | True | 39.75 | -184.87 |
|  | FosA2_detection |  | CGTGGGCAGCC | TTT | 11 | 67 | 54.63 | False | 0.00 | 0.00 |
| IMP-39 | IMP-39_fwd |  | TGATGAAGGCGTTTATGT |  | 18 | 122 | 57.42 | False | 0.00 | 0.00 |
|  | IMP-39_rev |  | CCGTAAATGGAGTGTCAATTA |  | 21 | 122 | 59.73 | False | 0.00 | 0.00 |
|  | IMP-39_lnc3 | GCAGCGTGGGCTCGCGTGCCGGGGTTTTTT | TTCGTTTGAAGAAG |  | 14 | 122 | 46.82 | False | 0.00 | 0.00 |
|  | IMP-39_detection |  | TTAACGGGTGGGG | TTT | 13 | 122 | 53.75 | False | 0.00 | 0.00 |
| IMP-9 | IMP-9_fwd |  | AGTCTTTGCCAGATTTAAA |  | 19 | 135 | 55.71 | False | 37.30 | -17.50 |
|  | IMP-9_rev |  | TCAGATAGGCATCAGTATTTA |  | 21 | 135 | 57.35 | False | 37.06 | -4.16 |
|  | IMP-9_lnc3 | GCAGCGTGGGCTCGCGTGCCGGGGTTTTTT | GAAGAAGTTAACGGT |  | 15 | 135 | 50.42 | False | 0.00 | 0.00 |
|  | IMP-9_detection |  | TGGGGTGTTATTCCT | TTT | 15 | 135 | 54.13 | False | 0.00 | 0.00 |
| IMP-15 | IMP-15_fwd |  | AGTATYTCCTCTCATTTTCATA |  | 22 | 123 | 57.11 | False | 0.00 | 0.00 |
|  | IMP-15_rev |  | TTGTACYTTACCGTCTTTT |  | 19 | 123 | 56.37 | False | 0.00 | 0.00 |
|  | IMP-15_lnc3 | GCAGCGTGGGCTCGCGTGCCGGGGTTTTTT | GGGCGGAATAGAGT |  | 14 | 123 | 54.42 | False | 0.00 | 0.00 |
|  | IMP-15_detection |  | GGCTTAATTCTCAATCT | TTT | 17 | 123 | 52.52 | False | 0.00 | 0.00 |
| BcII | BcII_lnc3 | GCAGCGTGGGCTCGCGTGCCGGGGTTTTTT | TAGCAAAGAAAAATGGA |  | 17 | 115 | 52.54 | False | 0.00 | 0.00 |
|  | BcII_detection |  | TATGAAGAACCACTTG | TTT | 16 | 115 | 51.21 | True | 42.03 | -360.32 |
|  | BcII_fwd |  | GGCATTAAAGCGCATAGTACA |  | 21 | 115 | 62.82 | False | 37.02 | -1.24 |
|  | BcII_rev |  | CTTTCATATTTCCAAACTTCAA |  | 22 | 115 | 57.05 | False | 0.00 | 0.00 |

### ABR-6

Supplementary Table S9: Primer and probe sequences and additional information of ABR-6.

| Gene | Primer/Probe | Hybridisation Sequence | Sequence | Spacer | Length | Product Size | Tm | Hairpin | Hairpin Tm | Hairpin delta G |
| --- | --- | --- | --- | --- | --- | --- | --- | --- | --- | --- |
| TetA | TetA_fwd |  | CCTTTCCTTTGGGTTCTCTAT |  | 21 | 155 | 61.14 | False | 0.00 | 0.00 |
|  | TetA_rev |  | ACCATCCCGAACCCGAAA |  | 18 | 155 | 64.69 | False | 40.68 | -207.54 |
|  | TetA_lnc3 | GCAGCGTGGGCTCGCGTGCCGGGGTTTTTT | CCGGCGCTTATATT |  | 14 | 155 | 53.03 | False | 0.00 | 0.00 |
|  | TetA_detection |  | GCCGATATCACTGAT | TTT | 15 | 155 | 52.88 | False | 0.00 | 0.00 |
| ErmF | ErmF_fwd |  | CCCGAAATGTTCAAGTTGT |  | 19 | 123 | 59.79 | False | 0.00 | 0.00 |
|  | ErmF_rev |  | CAAACATCAGGATTTTGAAAATATC |  | 25 | 123 | 59.93 | False | 37.57 | -50.22 |
|  | ErmF_lnc3 | GCAGCGTGGGCTCGCGTGCCGGGGTTTTTT | GTTCCGAAATTTCC |  | 14 | 123 | 48.43 | False | 0.00 | 0.00 |
|  | ErmF_detection |  | TTTCAAAGTGGTGT | TTT | 14 | 123 | 48.76 | False | 0.00 | 0.00 |
| GES-7 | GES-7_fwd |  | TCGGAAACCAAACGGGA |  | 17 | 128 | 61.83 | False | 43.59 | -378.78 |
|  | GES-7_rev |  | TCTCTCCTGGGCTTTAAAAAAA |  | 22 | 128 | 62.11 | False | 0.00 | 0.00 |
|  | GES-7_lnc3 | GCAGCGTGGGCTCGCGTGCCGGGGTTTTTT | GTTTTCCTAAAGATTG |  | 16 | 128 | 47.56 | False | 0.00 | 0.00 |
|  | GES-7_detection |  | GGTTGTTGGAGAGA | TTT | 14 | 128 | 51.37 | False | 0.00 | 0.00 |
| rosB | rosB_fwd |  | ACCGCTACGTGATGCAT |  | 17 | 150 | 62.14 | False | 0.00 | 0.00 |
|  | rosB_rev |  | CGCACTAATATAAACGCT |  | 18 | 150 | 55.77 | False | 0.00 | 0.00 |
|  | rosB_detection |  | ACCATTAGCTGTAC | TTT | 14 | 150 | 48.06 | False | 0.00 | 0.00 |
|  | rosB_lnc3 | GCAGCGTGGGCTCGCGTGCCGGGGTTTTTT | ATTTTGCTACGTGA |  | 14 | 150 | 48.87 | False | 0.00 | 0.00 |
| OXA-10 | OXA-10_fwd |  | CGGCCTAGAAACTGGTGT |  | 18 | 141 | 62.60 | False | 0.00 | 0.00 |
|  | OXA-10_rev |  | ATTTGTTGAAATACGGGAA |  | 19 | 141 | 56.13 | False | 35.00 | 146.41 |
|  | OXA-10_lnc3 | GCAGCGTGGGCTCGCGTGCCGGGGTTTTTT | AATGGGACGGAAAG |  | 14 | 141 | 52.45 | False | 0.00 | 0.00 |
|  | OXA-10_detection |  | CCAAGAGCCATGAA | TTT | 14 | 141 | 52.42 | False | 0.00 | 0.00 |
| OXA-72 | OXA-72_fwd |  | AAATGAGATTTTCAAATGGGA |  | 21 | 145 | 57.71 | False | 44.02 | -460.36 |
|  | OXA-72_rev |  | TTTCTGCATTAGCTCTAGGC |  | 20 | 145 | 61.32 | False | 44.96 | -505.47 |
|  | OXA-72_lnc3 | GCAGCGTGGGCTCGCGTGCCGGGGTTTTTT | ACTTTAGGTGAGGC |  | 14 | 145 | 51.46 | False | 0.00 | 0.00 |
|  | OXA-72_detection |  | AATGGCATTGTCAG | TTT | 14 | 145 | 50.30 | False | 0.00 | 0.00 |
| cmeB | cmeB_fwd |  | ACGATTCAACCTTTTCCCA |  | 19 | 102 | 60.71 | False | 0.00 | 0.00 |
|  | cmeB_rev |  | GTTTCTTTTGCTACTTGAG |  | 19 | 102 | 55.36 | False | 0.00 | 0.00 |
|  | cmeB_lnc3 | GCAGCGTGGGCTCGCGTGCCGGGGTTTTTT | GTTCAAGGTCAACC |  | 14 | 102 | 50.77 | False | 0.00 | 0.00 |
|  | cmeB_detection |  | CGCACCAGGTTATA | TTT | 14 | 102 | 51.81 | False | 0.00 | 0.00 |
| ErmT | ErmT_fwd |  | ATTTTTAATGACAGAAGTTGA |  | 21 | 102 | 55.26 | False | 0.00 | 0.00 |
|  | ErmT_rev |  | CTTTTTAATACAATTAACGAGCT |  | 23 | 102 | 57.65 | False | 0.00 | 0.00 |
|  | ErmT_detection |  | TCATCCAAAACCTA | TTT | 14 | 102 | 46.50 | False | 0.00 | 0.00 |
|  | ErmT_lnc3 | GCAGCGTGGGCTCGCGTGCCGGGGTTTTTT | CCCTAGAGAATACTT |  | 15 | 102 | 47.25 | False | 0.00 | 0.00 |
| murA | murA_fwd |  | CCGCGCATTTTATTGTCCAAG |  | 21 | 110 | 64.78 | False | 0.00 | 0.00 |
|  | murA_rev |  | GGAACAAAAATCCCATAAGG |  | 20 | 110 | 58.10 | False | 0.00 | 0.00 |
|  | murA_lnc3 | GCAGCGTGGGCTCGCGTGCCGGGGTTTTTT | AATTTTCTTGTGTTAATCG |  | 19 | 110 | 53.87 | False | 0.00 | 0.00 |
|  | murA_detection |  | GATTCCTATTTTGCTGTTA | TTT | 19 | 110 | 54.84 | False | 0.00 | 0.00 |
| CARB-22 | CARB-22_fwd |  | CTCAACTGTTAGTTACGC |  | 18 | 123 | 56.28 | False | 42.87 | -354.53 |
|  | CARB-22_rev |  | CCGCGATAATCCCAACG |  | 17 | 123 | 61.27 | False | 0.00 | 0.00 |
|  | CARB-22_lnc3 | GCAGCGTGGGCTCGCGTGCCGGGGTTTTTT | CATCGAGAAACAAAC |  | 15 | 123 | 50.04 | False | 0.00 | 0.00 |
|  | CARB-22_detection |  | ATCTGGGCGAATTG | TTT | 14 | 123 | 53.14 | False | 0.00 | 0.00 |
| IMP-19 | IMP-19_fwd |  | GGTGACGCAAATTTAGA |  | 17 | 92 | 55.15 | False | 0.00 | 0.00 |
|  | IMP-19_rev |  | TCACTATGACTTGAAACAA |  | 19 | 92 | 55.04 | False | 0.00 | 0.00 |
|  | IMP-19_lnc3 | GCAGCGTGGGCTCGCGTGCCGGGGTTTTTT | CAAAGTCCGCCAA |  | 13 | 92 | 52.52 | False | 0.00 | 0.00 |
|  | IMP-19_detection |  | AATATTAATGTCTAAATATG | TTT | 20 | 92 | 47.56 | False | 34.42 | 270.35 |
| DfrA1 | DfrA1_fwd |  | AGCCTGATCGATCAAGTAG |  | 19 | 104 | 59.79 | False | 40.28 | -220.70 |
|  | DfrA1_rev |  | AAAACTGGCCTAAAATTGC |  | 19 | 104 | 58.17 | False | 0.00 | 0.00 |
|  | DfrA1_lnc3 | GCAGCGTGGGCTCGCGTGCCGGGGTTTTTT | TACAATAGACATCGAGC |  | 17 | 104 | 54.64 | False | 0.00 | 0.00 |
|  | DfrA1_detection |  | CGGAAGGTGATGTTTA | TTT | 16 | 104 | 54.47 | False | 0.00 | 0.00 |
| norA | norA_fwd |  | TTGAATTCAACTTTTACCAGTAT |  | 23 | 116 | 58.50 | False | 0.00 | 0.00 |
|  | norA_rev |  | CCACTTAATGAAACCGCAATAGC |  | 23 | 116 | 64.74 | False | 37.42 | -24.46 |
|  | norA_lnc3 | GCAGCGTGGGCTCGCGTGCCGGGGTTTTTT | GGTGCATTATTCGAT |  | 15 | 116 | 51.18 | False | 0.00 | 0.00 |
|  | norA_detection |  | GTTAATTTAGAGTTTCCT | TTT | 18 | 116 | 50.59 | False | 0.00 | 0.00 |
| IMP-11 | IMP-11_fwd |  | TTTGCCYGATTTAAAAATTGA |  | 21 | 102 | 57.71 | False | 37.17 | -15.26 |
|  | IMP-11_rev |  | ACCAAACCGTGTTTAGW |  | 17 | 102 | 56.41 | False | 0.00 | 0.00 |
|  | IMP-11_lnc3 | GCAGCGTGGGCTCGCGTGCCGGGGTTTTTT | GGTGTTTATGTTCAT |  | 15 | 102 | 47.84 | False | 0.00 | 0.00 |
|  | IMP-11_detection |  | ACATCGTTTGAAGAA | TTT | 15 | 102 | 50.22 | False | 0.00 | 0.00 |
| TetD | TetD_fwd |  | TCAGCCTGACCAATCTGA |  | 18 | 122 | 61.18 | False | 36.75 | 21.87 |
|  | TetD_rev |  | CAGCAGACCGTACAGTG |  | 17 | 122 | 60.28 | False | 0.00 | 0.00 |
|  | TetD_detection |  | AGTCAGACACAGCA | TTT | 14 | 122 | 53.98 | False | 0.00 | 0.00 |
|  | TetD_lnc3 | GCAGCGTGGGCTCGCGTGCCGGGGTTTTTT | TGTTTGCTTTTATTTTC |  | 17 | 122 | 49.85 | False | 0.00 | 0.00 |
| y56 | y56_fwd |  | TGGCTGAAAGGAAAYACCAC |  | 20 | 122 | 63.27 | False | 44.24 | -462.81 |
|  | y56_rev |  | CAAAGTACCGCAATATCA |  | 18 | 122 | 55.54 | False | 0.00 | 0.00 |
|  | y56_detection |  | TGTGGGTGACAAAA | TTT | 14 | 122 | 51.91 | False | 0.00 | 0.00 |
|  | y56_lnc3 | GCAGCGTGGGCTCGCGTGCCGGGGTTTTTT | CCTACTGACTGGAT |  | 14 | 122 | 49.67 | False | 0.00 | 0.00 |
| TetC | TetC_fwd |  | GTTCTCGGAGCACTGTCC |  | 18 | 106 | 63.33 | False | 0.00 | 0.00 |
|  | TetC_rev |  | ACAGGACGGGTGTGGTC |  | 17 | 106 | 64.47 | False | 0.00 | 0.00 |
|  | TetC_lnc3 | GCAGCGTGGGCTCGCGTGCCGGGGTTTTTT | GCTTCGCTACTTGG |  | 14 | 106 | 54.27 | False | 0.00 | 0.00 |
|  | TetC_detection |  | AGCCACTATCGACTA | TTT | 15 | 106 | 53.33 | False | 0.00 | 0.00 |
| cat | cat_fwd |  | ACCTGAAAACACAGTTCC |  | 18 | 110 | 58.24 | False | 39.46 | -214.60 |
|  | cat_rev |  | TACCYATTGTAATAATTGGCAATA |  | 24 | 110 | 59.35 | False | 37.79 | -86.60 |
|  | cat_lnc3 | GCAGCGTGGGCTCGCGTGCCGGGGTTTTTT | GATTTTAGTTCATTTAA |  | 17 | 110 | 45.04 | False | 0.00 | 0.00 |
|  | cat_detection |  | TTTAAATATTGGTAATAAT | TTT | 19 | 110 | 45.82 | False | 0.00 | 0.00 |
| vatF | vatF_fwd |  | CTTTATCAAAAATACCACACA |  | 21 | 157 | 56.02 | False | 0.00 | 0.00 |
|  | vatF_rev |  | AGCTAATGCACAGAATTT |  | 18 | 157 | 55.27 | False | 44.96 | -505.47 |
|  | vatF_lnc3 | GCAGCGTGGGCTCGCGTGCCGGGGTTTTTT | ATTCTGAAAACTTTGAA |  | 17 | 157 | 50.21 | False | 0.00 | 0.00 |
|  | vatF_detection |  | CGTAACGTGCTTTA | TTT | 14 | 157 | 50.29 | False | 0.00 | 0.00 |
| OXA-61 | OXA-61_fwd |  | AGACTTTCACAAAATAGCT |  | 19 | 102 | 55.41 | False | 0.00 | 0.00 |
|  | OXA-61_rev |  | TTTTCCAAAAAGCTCTAAATTTT |  | 23 | 102 | 58.11 | False | 0.00 | 0.00 |
|  | OXA-61_lnc3 | GCAGCGTGGGCTCGCGTGCCGGGGTTTTTT | TTCTCAAGAAGCAATGA |  | 17 | 102 | 54.85 | False | 0.00 | 0.00 |
|  | OXA-61_detection |  | ATAGTGTTAAGGAAATGA | TTT | 18 | 102 | 51.86 | False | 0.00 | 0.00 |
| cmeC | cmeC_fwd |  | ATTACCTTAAAGCTTTAAAAATTTT |  | 25 | 147 | 57.10 | False | 37.37 | -32.36 |
|  | cmeC_rev |  | CAATATCTGGACGTTGAAG |  | 19 | 147 | 56.86 | False | 0.00 | 0.00 |
|  | cmeC_lnc3 | GCAGCGTGGGCTCGCGTGCCGGGGTTTTTT | ATCAAGTTTTTAATCTTAA |  | 19 | 147 | 49.15 | False | 0.00 | 0.00 |
|  | cmeC_detection |  | AGAATTTGACATTCCA | TTT | 16 | 147 | 50.94 | False | 0.00 | 0.00 |
| rosA | rosA_fwd |  | CCTCATTATTACAGCCACTTAT |  | 22 | 105 | 59.59 | False | 0.00 | 0.00 |
|  | rosA_rev |  | CAAGCAGCAAGATACCT |  | 17 | 105 | 56.95 | False | 0.00 | 0.00 |
|  | rosA_detection |  | GCAGCCCTATTCAC | TTT | 14 | 105 | 53.73 | False | 0.00 | 0.00 |
|  | rosA_lnc3 | GCAGCGTGGGCTCGCGTGCCGGGGTTTTTT | ACCGATAAGCATCC |  | 14 | 105 | 51.87 | False | 0.00 | 0.00 |
| OXA-2 | OXA-2_fwd |  | AACATCAGCGCTTGGTCA |  | 18 | 117 | 63.24 | False | 0.00 | 0.00 |
|  | OXA-2_rev |  | GCCACTCAACCCATCCTAC |  | 19 | 117 | 63.61 | False | 0.00 | 0.00 |
|  | OXA-2_detection |  | GTCGCAACTGGATA | TTT | 14 | 117 | 52.30 | False | 0.00 | 0.00 |
|  | OXA-2_lnc3 | GCAGCGTGGGCTCGCGTGCCGGGGTTTTTT | GATTGTGGAAGCCG |  | 14 | 117 | 54.63 | False | 0.00 | 0.00 |
| OKP-A-8 | OKP-A-8_fwd |  | GCAGTGGATGGTKGACG |  | 17 | 116 | 62.45 | False | 0.00 | 0.00 |
|  | OKP-A-8_rev |  | CGACAATGCCRCGTGAG |  | 17 | 116 | 63.33 | False | 0.00 | 0.00 |
|  | OKP-A-8_lnc3 | GCAGCGTGGGCTCGCGTGCCGGGGTTTTTT | GCTGGTTTATCGCC |  | 14 | 116 | 54.74 | False | 0.00 | 0.00 |
|  | OKP-A-8_detection |  | GATAAAACCGGGGC | TTT | 14 | 116 | 54.13 | False | 0.00 | 0.00 |
| Arr3 | Arr3_fwd |  | TTTCCCGGTAATCCAACACAG |  | 21 | 110 | 64.40 | False | 0.00 | 0.00 |
|  | Arr3_rev |  | TCCAACATTCCCCTTATTAA |  | 20 | 110 | 57.57 | False | 0.00 | 0.00 |
|  | Arr3_lnc3 | GCAGCGTGGGCTCGCGTGCCGGGGTTTTTT | CGAACCCTTGAGAA |  | 14 | 110 | 52.00 | False | 0.00 | 0.00 |
|  | Arr3_detection |  | TTGTTGGCGTTGTT | TTT | 14 | 110 | 53.99 | False | 0.00 | 0.00 |
| IMP-29 | IMP-29_fwd |  | TTGCCAGATTTAAAAATTGAGA |  | 22 | 116 | 58.71 | False | 0.00 | 0.00 |
|  | IMP-29_rev |  | GTATTTACAAGAACCACCAA |  | 20 | 116 | 57.00 | False | 0.00 | 0.00 |
|  | IMP-29_detection |  | AGAAGTTAACGGTT | TTT | 14 | 116 | 47.65 | False | 0.00 | 0.00 |
|  | IMP-29_lnc3 | GCAGCGTGGGCTCGCGTGCCGGGGTTTTTT | CATACTTCGTTTGA |  | 14 | 116 | 46.08 | False | 0.00 | 0.00 |
| blmS | blmS_fwd |  | CCTCGACCCAGCTACGA |  | 17 | 105 | 63.57 | False | 36.38 | 57.11 |
|  | blmS_rev |  | CCGGTGCTTTTTTCCTCG |  | 18 | 105 | 62.61 | False | 0.00 | 0.00 |
|  | blmS_detection |  | CCATGGTGGCATTG | TTT | 14 | 105 | 54.93 | False | 0.00 | 0.00 |
|  | blmS_lnc3 | GCAGCGTGGGCTCGCGTGCCGGGGTTTTTT | TGGATGATCTCGATG |  | 15 | 105 | 52.38 | False | 0.00 | 0.00 |
| SulIII | SulIII_fwd |  | AGATTGCAAACTTGTGTT |  | 18 | 123 | 56.12 | False | 40.33 | -212.43 |
|  | SulIII_rev |  | AAAGCAGCAATTCTTTCTTTAAA |  | 23 | 123 | 60.12 | False | 0.00 | 0.00 |
|  | SulIII_detection |  | CGGAAGAGGTTTTTAC | TTT | 16 | 123 | 53.05 | False | 0.00 | 0.00 |
|  | SulIII_lnc3 | GCAGCGTGGGCTCGCGTGCCGGGGTTTTTT | TAAAGTTGAAACGAATC |  | 17 | 123 | 51.10 | False | 0.00 | 0.00 |
| lnuB | lnuB_fwd |  | AAGAAATTAGCCTTGAAAATTA |  | 22 | 145 | 56.12 | False | 0.00 | 0.00 |
|  | lnuB_rev |  | ACTTTTAAGTTGTATTGTTCA |  | 21 | 145 | 55.21 | False | 40.57 | -183.10 |
|  | lnuB_lnc3 | GCAGCGTGGGCTCGCGTGCCGGGGTTTTTT | CTCGATTAGATAAGGTA |  | 17 | 145 | 50.11 | False | 0.00 | 0.00 |
|  | lnuB_detection |  | GAATTATTTGAAGCCT | TTT | 16 | 145 | 49.35 | False | 0.00 | 0.00 |
| FexA | FexA_fwd |  | AAAACGTGGGGGAGCTTT |  | 18 | 104 | 63.14 | False | 0.00 | 0.00 |
|  | FexA_rev |  | CATTCCACCCTAAATATTGACCA |  | 23 | 104 | 62.59 | False | 0.00 | 0.00 |
|  | FexA_lnc3 | GCAGCGTGGGCTCGCGTGCCGGGGTTTTTT | GCAGGAAGTATTGG |  | 14 | 104 | 50.10 | False | 0.00 | 0.00 |
|  | FexA_detection |  | TGTTGGAACTGCTG | TTT | 14 | 104 | 53.26 | False | 0.00 | 0.00 |
| mph | mph_fwd |  | ATTCTTACTGCTAATGAATTAA |  | 22 | 100 | 55.10 | False | 0.00 | 0.00 |
|  | mph_rev |  | CTTGCCAACGATCCCATA |  | 18 | 100 | 60.35 | False | 0.00 | 0.00 |
|  | mph_detection |  | AGTGAAGGAACAATAC | TTT | 16 | 100 | 51.20 | False | 0.00 | 0.00 |
|  | mph_lnc3 | GCAGCGTGGGCTCGCGTGCCGGGGTTTTTT | ACAAAGGATGAATCG |  | 15 | 100 | 50.97 | False | 0.00 | 0.00 |
| VanX-B | VanX-B_fwd |  | GATTTTATGGACGAACGGT |  | 19 | 134 | 59.03 | False | 0.00 | 0.00 |
|  | VanX-B_rev |  | TTATAGTGCCACCATTCA |  | 18 | 134 | 56.29 | False | 0.00 | 0.00 |
|  | VanX-B_detection |  | ATCATGGAAAGCAGC | TTT | 15 | 134 | 54.70 | False | 0.00 | 0.00 |
|  | VanX-B_lnc3 | GCAGCGTGGGCTCGCGTGCCGGGGTTTTTT | AGATGCTTGCGTAAA |  | 15 | 134 | 53.75 | False | 0.00 | 0.00 |
| catB9 | catB9_fwd |  | TCGCAGTGATTGGATAAGTAC |  | 21 | 116 | 61.59 | False | 0.00 | 0.00 |
|  | catB9_rev |  | TCCACACATCATGACCAATAATTG |  | 24 | 116 | 63.80 | False | 0.00 | 0.00 |
|  | catB9_detection |  | CACGCGATGGTTTT | TTT | 14 | 116 | 54.96 | False | 0.00 | 0.00 |
|  | catB9_lnc3 | GCAGCGTGGGCTCGCGTGCCGGGGTTTTTT | ATGATAATTTTGCAGATG |  | 18 | 116 | 52.31 | False | 0.00 | 0.00 |
| qacA | qacA_fwd |  | GCTTTAGCATTAATTTTAGTTG |  | 22 | 119 | 56.20 | False | 43.64 | -454.72 |
|  | qacA_rev |  | ATAGACTCTTCAACAGCAGC |  | 20 | 119 | 61.25 | False | 0.00 | 0.00 |
|  | qacA_lnc3 | GCAGCGTGGGCTCGCGTGCCGGGGTTTTTT | CTTCACTAGCAGTTG |  | 15 | 119 | 51.51 | False | 0.00 | 0.00 |
|  | qacA_detection |  | CATCTGCTCTAATAAT | TTT | 16 | 119 | 47.56 | False | 0.00 | 0.00 |
| Qnr-S1 | Qnr-S1_fwd |  | TGTTTGAAAATCGCTGGAT |  | 19 | 134 | 59.17 | False | 0.00 | 0.00 |
|  | Qnr-S1_rev |  | TTCGGCGTGRCATAAATT |  | 18 | 134 | 59.74 | False | 0.00 | 0.00 |
|  | Qnr-S1_lnc3 | GCAGCGTGGGCTCGCGTGCCGGGGTTTTTT | AGAGTCAGACTTAAG |  | 15 | 134 | 48.19 | False | 0.00 | 0.00 |
|  | Qnr-S1_detection |  | TCGAGGTGTTTTTT | TTT | 14 | 134 | 49.00 | False | 0.00 | 0.00 |
| cepA | cepA_fwd |  | ATAAGTATCTTCATTCATTGGGAA |  | 24 | 138 | 60.11 | False | 42.59 | -366.57 |
|  | cepA_rev |  | TGCGAAATATTTCCAGTAAT |  | 20 | 138 | 56.54 | False | 37.34 | -25.59 |
|  | cepA_detection |  | CCAAAACTGGACTA | TTT | 14 | 138 | 48.19 | False | 0.00 | 0.00 |
|  | cepA_lnc3 | GCAGCGTGGGCTCGCGTGCCGGGGTTTTTT | CTGGAGTTCTGTTA |  | 14 | 138 | 47.40 | False | 0.00 | 0.00 |
| cmeA | cmeA_fwd |  | AGGAGATGCTTTAGTTAATATAGG |  | 24 | 167 | 59.73 | False | 0.00 | 0.00 |
|  | cmeA_rev |  | GAATGCTGTCTAARTCCCA |  | 19 | 167 | 59.28 | False | 0.00 | 0.00 |
|  | cmeA_lnc3 | GCAGCGTGGGCTCGCGTGCCGGGGTTTTTT | TTTAAATCCTATTTACG |  | 17 | 167 | 47.07 | False | 0.00 | 0.00 |
|  | cmeA_detection |  | CAGATTTCTTTATTTCA | TTT | 17 | 167 | 47.64 | False | 0.00 | 0.00 |
| EreA2 | EreA2_fwd |  | GTATGGCTCAGTGCTGA |  | 17 | 130 | 59.18 | False | 44.38 | -522.77 |
|  | EreA2_rev |  | GATAATTTCGGCCAATTG |  | 18 | 130 | 55.03 | False | 0.00 | 0.00 |
|  | EreA2_lnc3 | GCAGCGTGGGCTCGCGTGCCGGGGTTTTTT | GAATCAGGAAGAAAAC |  | 16 | 130 | 50.29 | False | 0.00 | 0.00 |
|  | EreA2_detection |  | TGCAGTTAGTCGGA | TTT | 14 | 130 | 53.70 | False | 0.00 | 0.00 |
| VanB | VanB_fwd |  | TGATTTGATTGTCGGCGA |  | 18 | 132 | 60.58 | False | 0.00 | 0.00 |
|  | VanB_rev |  | TCCTCGACCGGAATGTC |  | 17 | 132 | 61.38 | False | 0.00 | 0.00 |
|  | VanB_lnc3 | GCAGCGTGGGCTCGCGTGCCGGGGTTTTTT | ATCTTCCGCATCCA |  | 14 | 132 | 54.25 | False | 0.00 | 0.00 |
|  | VanB_detection |  | TCAGGAAAACGAGC | TTT | 14 | 132 | 53.03 | False | 0.00 | 0.00 |
| DHA-1 | DHA-1_fwd |  | TTTATTCCGGAAAAACAGG |  | 19 | 93 | 56.86 | False | 36.92 | 6.55 |
|  | DHA-1_rev |  | ACTCAAAATAGCCTGTGC |  | 18 | 93 | 58.49 | False | 37.95 | -51.15 |
|  | DHA-1_detection |  | ATACCGAAAGAGTC | TTT | 14 | 93 | 47.74 | False | 0.00 | 0.00 |
|  | DHA-1_lnc3 | GCAGCGTGGGCTCGCGTGCCGGGGTTTTTT | TAAAAACTACCCGA |  | 14 | 93 | 46.72 | False | 0.00 | 0.00 |
| DfrA14 | DfrA14_fwd |  | TTGTATTTCAGTCAATCGA |  | 19 | 126 | 55.24 | False | 0.00 | 0.00 |
|  | DfrA14_rev |  | TCGATAAGTGGAGCGTAG |  | 18 | 126 | 59.07 | False | 0.00 | 0.00 |
|  | DfrA14_detection |  | GGCGGAGAAATTTAC | TTT | 15 | 126 | 52.47 | False | 0.00 | 0.00 |
|  | DfrA14_lnc3 | GCAGCGTGGGCTCGCGTGCCGGGGTTTTTT | CGTTATAGTGTCTGGT |  | 16 | 126 | 53.39 | False | 0.00 | 0.00 |
| CTX-M-2 | CTX-M-2_fwd |  | GGCGCAGACCCTGAAAAA |  | 18 | 154 | 64.26 | False | 37.03 | -1.90 |
|  | CTX-M-2_rev |  | GCTGCCGGTTTTATCGC |  | 17 | 154 | 62.56 | False | 0.00 | 0.00 |
|  | CTX-M-2_detection |  | GGCAATACTACCGG | TTT | 14 | 154 | 52.93 | False | 0.00 | 0.00 |
|  | CTX-M-2_lnc3 | GCAGCGTGGGCTCGCGTGCCGGGGTTTTTT | TGACGTGGCTTAAG |  | 14 | 154 | 52.62 | False | 0.00 | 0.00 |
| ANT(6)-Ia | ANT(6)-Ia_fwd |  | TGGAATGTAACACCTTATG |  | 19 | 139 | 55.59 | False | 0.00 | 0.00 |
|  | ANT(6)-Ia_rev |  | ATGAAAATTCTGTCTTTATCCC |  | 22 | 139 | 58.31 | False | 0.00 | 0.00 |
|  | ANT(6)-Ia_lnc3 | GCAGCGTGGGCTCGCGTGCCGGGGTTTTTT | CGCAAAGAGATACTGT |  | 16 | 139 | 54.63 | False | 0.00 | 0.00 |
|  | ANT(6)-Ia_detection |  | TTGCAATCGATCATCT | TTT | 16 | 139 | 54.15 | False | 0.00 | 0.00 |
| Aph4-Ia | Aph4-Ia_fwd |  | CCGATCTTAGCCAGACG |  | 17 | 135 | 59.79 | False | 38.04 | -54.34 |
|  | Aph4-Ia_rev |  | CGGTGTCGTCCATCACAG |  | 18 | 135 | 63.69 | False | 39.49 | -224.47 |
|  | Aph4-Ia_lnc3 | GCAGCGTGGGCTCGCGTGCCGGGGTTTTTT | GGAATCGGTCAATAC |  | 15 | 135 | 51.28 | False | 0.00 | 0.00 |
|  | Aph4-Ia_detection |  | ACTACATGGCGTGA | TTT | 14 | 135 | 54.22 | False | 0.00 | 0.00 |
| ACT-1 | ACT-1_fwd |  | ATGCTGGATCTGGCAAC |  | 17 | 161 | 60.29 | True | 45.37 | -677.85 |
|  | ACT-1_rev |  | CCAAAAAGACCGATGCTG |  | 18 | 161 | 59.95 | False | 0.00 | 0.00 |
|  | ACT-1_lnc3 | GCAGCGTGGGCTCGCGTGCCGGGGTTTTTT | TGCGCTTTTATCA |  | 13 | 161 | 47.80 | False | 0.00 | 0.00 |
|  | ACT-1_detection |  | AAACTGGCAGCC | TTT | 12 | 161 | 50.87 | False | 0.00 | 0.00 |
| Enterobacter_acrA | Enterobacter_acrA_fwd |  | CAGGCGATTGGSGATAAA |  | 18 | 174 | 60.90 | True | 49.34 | -833.86 |
|  | Enterobacter_acrA_rev |  | TTAAGACTTGGTTTGTTCTGAC |  | 22 | 174 | 60.66 | False | 0.00 | 0.00 |
|  | Enterobacter_acrA_lnc3 | GCAGCGTGGGCTCGCGTGCCGGGGTTTTTT | GGTCTGAAAGATGGC |  | 15 | 174 | 54.76 | True | 53.97 | -861.07 |
|  | Enterobacter_acrA_detection | GATCGCGTGATTGT | TTT | 14 | 174 | 53.87 | True | 37.60 | -34.94 |  |
| ArmR | ArmR_fwd |  | ATGTCCCTGAACACTCCG |  | 18 | 144 | 62.29 | False | 34.89 | 131.65 |
|  | ArmR_rev |  | GTAGAGGTCCCAGGCATT |  | 18 | 144 | 61.67 | False | 0.00 | 0.00 |
|  | ArmR_lnc3 | GCAGCGTGGGCTCGCGTGCCGGGGTTTTTT | CAAACCGTCCCG |  | 12 | 144 | 51.95 | False | 0.00 | 0.00 |
|  | ArmR_detection |  | CACCGAGACCGAA | TTT | 13 | 144 | 54.10 | True | 40.05 | -172.46 |

### ABR-7

Supplementary Table S10: Primer and probe sequences and additional information of ABR-7.

| Gene | Primer/Probe | Hybridisation Sequence | Sequence | Spacer | Length | Product Size | Tm | Hairpin | Hairpin Tm | Hairpin delta G |
| --- | --- | --- | --- | --- | --- | --- | --- | --- | --- | --- |
| lsaE | lsaE_fwd |  | TCCTGCTTATTGATGAACCTA |  | 21 | 106 | 60.28 | False | 0.00 | 0.00 |
|  | lsaE_rev |  | CTATCATGAGATACCAAAATAAAC |  | 24 | 106 | 57.66 | False | 39.19 | -171.08 |
|  | lsaE_lnc3 | GCAGCGTGGGCTCGCGTGCCGGGGTTTTTT | ACGTAATGTAGTGC |  | 14 | 106 | 49.29 | False | 0.00 | 0.00 |
|  | lsaE_detection |  | AAAACTATTTGAAACG | TTT | 16 | 106 | 47.89 | False | 0.00 | 0.00 |
| CfxA2 | CfxA2_fwd |  | AACTATACATCTCCTCTTGGTG |  | 22 | 124 | 60.94 | False | 36.20 | 55.43 |
|  | CfxA2_rev |  | TATCTACACCTGTTTTGCAT |  | 20 | 124 | 58.30 | False | 0.00 | 0.00 |
|  | CfxA2_lnc3 | GCAGCGTGGGCTCGCGTGCCGGGGTTTTTT | CGATGATGAGAAAC |  | 14 | 124 | 47.42 | False | 0.00 | 0.00 |
|  | CfxA2_detection |  | AAAGTTTCATTAAGAA | TTT | 16 | 124 | 45.29 | False | 0.00 | 0.00 |
| ErmX | ErmX_fwd |  | GTGGTCCATGATGATTTC |  | 18 | 154 | 56.25 | False | 0.00 | 0.00 |
|  | ErmX_rev |  | CGACTTCCCACTGCATGAG |  | 19 | 154 | 64.22 | False | 0.00 | 0.00 |
|  | ErmX_detection |  | TGCCATTCTTCGAAA | TTT | 15 | 154 | 53.61 | False | 0.00 | 0.00 |
|  | ErmX_lnc3 | GCAGCGTGGGCTCGCGTGCCGGGGTTTTTT | CTTTCACCTCACCAC |  | 15 | 154 | 54.58 | False | 0.00 | 0.00 |
| OXA-48 | OXA-48_fwd |  | AGCAAGGATTTACCAATAATC |  | 21 | 137 | 57.67 | False | 44.24 | -462.81 |
|  | OXA-48_rev |  | TCCATCCCACTTAAAGACT |  | 19 | 137 | 58.74 | False | 0.00 | 0.00 |
|  | OXA-48_detection |  | AAATTCCCAATAGCTT | TTT | 16 | 137 | 50.95 | False | 0.00 | 0.00 |
|  | OXA-48_lnc3 | GCAGCGTGGGCTCGCGTGCCGGGGTTTTTT | CCGCATCTACCTTTA |  | 15 | 137 | 52.66 | False | 0.00 | 0.00 |
| QnrD | QnrD_fwd |  | GGAAAAGCACTTTATCAATGAAAA |  | 24 | 104 | 61.03 | False | 35.42 | 112.95 |
|  | QnrD_rev |  | TTAAATCAACCCCTGAAAAATCAC |  | 24 | 104 | 62.33 | False | 42.22 | -318.07 |
|  | QnrD_lnc3 | GCAGCGTGGGCTCGCGTGCCGGGGTTTTTT | AATTTACGGGGAATAGA |  | 17 | 104 | 53.43 | False | 0.00 | 0.00 |
|  | QnrD_detection |  | GTTAAAAATATTGCCTTT | TTT | 18 | 104 | 50.34 | False | 0.00 | 0.00 |
| dfrA19 | dfrA19_fwd |  | GAATACCGATCAACGCAT |  | 18 | 108 | 58.44 | False | 43.04 | -429.73 |
|  | dfrA19_rev |  | CAGTCGAACTCTCTTGGAATT |  | 21 | 108 | 61.74 | False | 0.00 | 0.00 |
|  | dfrA19_detection |  | GTACATTCAAGCTCT | TTT | 15 | 108 | 50.05 | False | 0.00 | 0.00 |
|  | dfrA19_lnc3 | GCAGCGTGGGCTCGCGTGCCGGGGTTTTTT | GGTGGGGAGAAGTT |  | 14 | 108 | 54.41 | False | 0.00 | 0.00 |
| BlaA | BlaA_fwd |  | GATAAAACTGGAACTTGTCAA |  | 21 | 103 | 58.05 | False | 0.00 | 0.00 |
|  | BlaA_rev |  | GCGATTGGGTATAAAAAATTGCC |  | 23 | 103 | 63.45 | False | 39.49 | -280.96 |
|  | BlaA_lnc3 | GCAGCGTGGGCTCGCGTGCCGGGGTTTTTT | AAATGATGTTGCTATTAT |  | 18 | 103 | 50.66 | False | 0.00 | 0.00 |
|  | BlaA_detection |  | TTGGCCTGATGATAAT | TTT | 16 | 103 | 53.13 | False | 0.00 | 0.00 |
| VanS-B | VanS-B_fwd |  | ACAGCAATTTGTGTCTTATTTCA |  | 23 | 136 | 61.53 | False | 0.00 | 0.00 |
|  | VanS-B_rev |  | GTTATTGTAGTGAAACAGC |  | 19 | 136 | 55.14 | True | 45.15 | -307.34 |
|  | VanS-B_lnc3 | GCAGCGTGGGCTCGCGTGCCGGGGTTTTTT | ACAGCAAACAGTAAAAT |  | 17 | 136 | 53.30 | False | 0.00 | 0.00 |
|  | VanS-B_detection |  | CCTATCAGCCATTGG | TTT | 15 | 136 | 54.01 | False | 0.00 | 0.00 |
| CatA2 | CatA2_fwd |  | CTGACCACCAAACTCGATATT |  | 21 | 118 | 61.81 | False | 0.00 | 0.00 |
|  | CatA2_rev |  | GGAACTCCGGAAACTGATT |  | 19 | 118 | 60.86 | False | 0.00 | 0.00 |
|  | CatA2_detection |  | TACCTGATCTCCCG | TTT | 14 | 118 | 53.02 | False | 0.00 | 0.00 |
|  | CatA2_lnc3 | GCAGCGTGGGCTCGCGTGCCGGGGTTTTTT | ATCCGCTGATGATT |  | 14 | 118 | 51.20 | False | 0.00 | 0.00 |
| Qnr-A1 | Qnr-A1_fwd |  | TATCAGTGTGACTTCAGC |  | 18 | 127 | 57.13 | False | 0.00 | 0.00 |
|  | Qnr-A1_rev |  | CCTTGAAACTGGCATCGC |  | 18 | 127 | 62.69 | False | 0.00 | 0.00 |
|  | Qnr-A1_lnc3 | GCAGCGTGGGCTCGCGTGCCGGGGTTTTTT | GATGCCAGTTTCGA |  | 14 | 127 | 53.13 | False | 0.00 | 0.00 |
|  | Qnr-A1_detection |  | GGATTGCAGTTTCAT | TTT | 15 | 127 | 51.68 | False | 0.00 | 0.00 |
| OXA-58 | OXA-58_fwd |  | TGTTCAAGCGCTTTTTAATGA |  | 21 | 119 | 61.04 | False | 0.00 | 0.00 |
|  | OXA-58_rev |  | ATGCAGGAATATAAGCTGT |  | 19 | 119 | 57.34 | False | 0.00 | 0.00 |
|  | OXA-58_detection |  | GCATTTAGACCGAG | TTT | 14 | 119 | 50.40 | False | 0.00 | 0.00 |
|  | OXA-58_lnc3 | GCAGCGTGGGCTCGCGTGCCGGGGTTTTTT | TATTAAAAAATATGGCAC |  | 18 | 119 | 49.41 | False | 0.00 | 0.00 |
| VanR-B | VanR-B_fwd |  | CCCCTGACACGAAAAGAAT |  | 19 | 107 | 61.17 | False | 0.00 | 0.00 |
|  | VanR-B_rev |  | TCAAAGTCATATCCCCATA |  | 19 | 107 | 55.86 | False | 0.00 | 0.00 |
|  | VanR-B_detection |  | CAGAACAAAGGCAGA | TTT | 15 | 107 | 54.03 | False | 0.00 | 0.00 |
|  | VanR-B_lnc3 | GCAGCGTGGGCTCGCGTGCCGGGGTTTTTT | TTTTGCTGCTGGTG |  | 14 | 107 | 54.63 | False | 0.00 | 0.00 |
| FOX-5 | FOX-5_fwd |  | TCAAGGMGTATCGGATCC |  | 18 | 126 | 60.25 | False | 38.14 | -41.55 |
|  | FOX-5_rev |  | TCTCGAACAGSGTCTGC |  | 17 | 126 | 62.19 | False | 43.79 | -477.85 |
|  | FOX-5_lnc3 | GCAGCGTGGGCTCGCGTGCCGGGGTTTTTT | GCCCACTATTTCAAC |  | 15 | 126 | 52.29 | False | 0.00 | 0.00 |
|  | FOX-5_detection |  | TATGGGGTTGCCAA | TTT | 14 | 126 | 54.33 | False | 0.00 | 0.00 |
| bmr | bmr_fwd |  | GCGGTATGAATTCAATGTTT |  | 20 | 106 | 58.49 | False | 0.00 | 0.00 |
|  | bmr_rev |  | ACCGTTGCAAAGTAGAA |  | 17 | 106 | 56.70 | False | 0.00 | 0.00 |
|  | bmr_lnc3 | GCAGCGTGGGCTCGCGTGCCGGGGTTTTTT | CCTATTATCGGCGG |  | 14 | 106 | 52.78 | False | 0.00 | 0.00 |
|  | bmr_detection |  | AATGCTGTTCGATAT | TTT | 15 | 106 | 49.66 | False | 0.00 | 0.00 |
| blt | blt_fwd |  | GTCTACTGTTATGTCAGGA |  | 19 | 109 | 56.61 | False | 42.59 | -501.25 |
|  | blt_rev |  | GGCCATATTGGATAAATGAG |  | 20 | 109 | 57.44 | True | 46.41 | -483.10 |
|  | blt_lnc3 | GCAGCGTGGGCTCGCGTGCCGGGGTTTTTT | ACTGTTTTGCTTGTA |  | 15 | 109 | 50.44 | False | 0.00 | 0.00 |
|  | blt_detection |  | ACTTGTTTTATTTTTCT | TTT | 17 | 109 | 47.55 | False | 0.00 | 0.00 |
| ANT(6)-Ib | ANT(6)-Ib_fwd |  | ATGAGATCAGAAAAGGAAG |  | 19 | 103 | 55.27 | False | 0.00 | 0.00 |
|  | ANT(6)-Ib_rev |  | TATTTGTATTTGTTCTAGATCCT |  | 23 | 103 | 56.97 | False | 0.00 | 0.00 |
|  | ANT(6)-Ib_detection |  | GCAAAAACAGACAAA | TTT | 15 | 103 | 50.31 | False | 0.00 | 0.00 |
|  | ANT(6)-Ib_lnc3 | GCAGCGTGGGCTCGCGTGCCGGGGTTTTTT | GATATTGTTTTGAATTTT |  | 18 | 103 | 47.92 | False | 0.00 | 0.00 |
| AAC(6')-IIc | AAC(6')-IIc_fwd |  | GATGAACGCCCAACTCTTGA |  | 20 | 137 | 64.28 | False | 0.00 | 0.00 |
|  | AAC(6')-IIc_rev |  | CTTCCAAGTGCGATGTAGGAT |  | 21 | 137 | 63.96 | False | 0.00 | 0.00 |
|  | AAC(6')-IIc_detection |  | AGCAAGCTGTAGTG | TTT | 14 | 137 | 52.56 | False | 0.00 | 0.00 |
|  | AAC(6')-IIc_lnc3 | GCAGCGTGGGCTCGCGTGCCGGGGTTTTTT | CGAAGTTCTGGCAA |  | 14 | 137 | 53.45 | False | 0.00 | 0.00 |
| aadK | aadK_fwd |  | CATTTAGTATGGGGAAGAACTATA |  | 24 | 147 | 59.67 | False | 0.00 | 0.00 |
|  | aadK_rev |  | AATACTTTCTAAATAATGCATAGCA |  | 25 | 147 | 59.44 | True | 47.25 | -608.10 |
|  | aadK_detection |  | AGGAACTCATGTCTAC | TTT | 16 | 147 | 52.83 | False | 0.00 | 0.00 |
|  | aadK_lnc3 | GCAGCGTGGGCTCGCGTGCCGGGGTTTTTT | TTTCAAATAAAGAATGGG |  | 18 | 147 | 51.99 | False | 0.00 | 0.00 |
| APH(3')-IIa | APH(3')-IIa_fwd |  | ATCTCGTCGTGACCCAT |  | 17 | 84 | 60.63 | False | 0.00 | 0.00 |
|  | APH(3')-IIa_rev |  | GGCCACAGTCGATGAATC |  | 18 | 84 | 61.45 | False | 0.00 | 0.00 |
|  | APH(3')-IIa_lnc3 | GCAGCGTGGGCTCGCGTGCCGGGGTTTTTT | TGCTTGCCGAATATC |  | 15 | 84 | 54.49 | False | 0.00 | 0.00 |
|  | APH(3')-IIa_detection |  | ATGGTGGAAAATGGC | TTT | 15 | 84 | 54.93 | False | 0.00 | 0.00 |
| VEB-1a | VEB-1a_fwd |  | CAATCAAAGCAAACGAAGAACAAA |  | 24 | 164 | 63.77 | False | 0.00 | 0.00 |
|  | VEB-1a_rev |  | TTCTCTCATAATTTTCCAAATAA |  | 23 | 164 | 56.07 | False | 0.00 | 0.00 |
|  | VEB-1a_detection |  | CAAACTGTTAATAGATACT | TTT | 19 | 164 | 51.17 | False | 0.00 | 0.00 |
|  | VEB-1a_lnc3 | GCAGCGTGGGCTCGCGTGCCGGGGTTTTTT | CCAACAGCGATGAA |  | 14 | 164 | 53.55 | False | 0.00 | 0.00 |
| VanY-B | VanY-B_fwd |  | AGATGGCTGGATGAAAACAG |  | 20 | 126 | 61.85 | False | 39.43 | -201.72 |
|  | VanY-B_rev |  | TGTGGCAGCTTCGATGC |  | 17 | 126 | 63.86 | False | 44.90 | -613.75 |
|  | VanY-B_lnc3 | GCAGCGTGGGCTCGCGTGCCGGGGTTTTTT | AGACAGAGATAACCG |  | 15 | 126 | 51.79 | False | 0.00 | 0.00 |
|  | VanY-B_detection |  | GTGTGAGCAACGAG | TTT | 14 | 126 | 54.63 | False | 0.00 | 0.00 |
| AmpC | AmpC_fwd |  | GTGAAACCTTCCGGCAT |  | 17 | 125 | 60.54 | False | 0.00 | 0.00 |
|  | AmpC_rev |  | CGATAACCCCAGGCRTA |  | 17 | 125 | 60.02 | False | 0.00 | 0.00 |
|  | AmpC_detection |  | CACACCTGGATTAA | TTT | 14 | 125 | 48.29 | False | 0.00 | 0.00 |
|  | AmpC_lnc3 | GCAGCGTGGGCTCGCGTGCCGGGGTTTTTT | CGTTAAAACTGAGC |  | 14 | 125 | 48.64 | False | 0.00 | 0.00 |
| ROB-1 | ROB-1_fwd |  | GCAGATGAACGCTTTGC |  | 17 | 149 | 60.36 | False | 0.00 | 0.00 |
|  | ROB-1_rev |  | ACGTATTTTTGGGTTTCGGG |  | 20 | 149 | 62.69 | False | 0.00 | 0.00 |
|  | ROB-1_detection |  | GATTTAAATCGTACCATT | TTT | 18 | 149 | 51.61 | False | 0.00 | 0.00 |
|  | ROB-1_lnc3 | GCAGCGTGGGCTCGCGTGCCGGGGTTTTTT | CGCTGCCTGAAAAA |  | 14 | 149 | 54.80 | False | 0.00 | 0.00 |
| cfrA | cfrA_fwd |  | GGTAAACATGAAGTATAAAGC |  | 21 | 130 | 56.28 | False | 0.00 | 0.00 |
|  | cfrA_rev |  | TATCTCATCTACAGTTAGGTT |  | 21 | 130 | 56.63 | False | 0.00 | 0.00 |
|  | cfrA_lnc3 | GCAGCGTGGGCTCGCGTGCCGGGGTTTTTT | TTGTATATCATCACAATG |  | 18 | 130 | 50.91 | False | 33.65 | 271.56 |
|  | cfrA_detection |  | CGGATGTAATTTTGGG | TTT | 16 | 130 | 53.54 | False | 0.00 | 0.00 |
| QnrB4 | QnrB4_fwd |  | TCAGATTTTCGCGGCGC |  | 17 | 137 | 64.59 | False | 0.00 | 0.00 |
|  | QnrB4_rev |  | TTTTCCCACAGCTCGCAC |  | 18 | 137 | 64.23 | False | 0.00 | 0.00 |
|  | QnrB4_lnc3 | GCAGCGTGGGCTCGCGTGCCGGGGTTTTTT | ATATCACCAATACCAAC |  | 17 | 137 | 52.13 | False | 0.00 | 0.00 |
|  | QnrB4_detection |  | TTAAGCTACGCCAAC | TTT | 15 | 137 | 54.32 | False | 0.00 | 0.00 |
| cfiA7 | cfiA7_fwd |  | TTATCCTTATCTCCATGCTT |  | 20 | 122 | 57.14 | False | 0.00 | 0.00 |
|  | cfiA7_rev |  | TTCGGCGAGGGATACATAAGT |  | 21 | 122 | 64.92 | False | 39.74 | -88.36 |
|  | cfiA7_lnc3 | GCAGCGTGGGCTCGCGTGCCGGGGTTTTTT | GTCGCAGTTATGGC |  | 14 | 122 | 54.79 | False | 0.00 | 0.00 |
|  | cfiA7_detection |  | ACAGAAAAGCGTAAAAA | TTT | 17 | 122 | 53.74 | False | 0.00 | 0.00 |
| VanW-B | VanW-B_fwd |  | GCGGGATTTATGAATATGCC |  | 20 | 94 | 60.66 | False | 0.00 | 0.00 |
|  | VanW-B_rev |  | CATTTGTTTGTATAAAGCAGC |  | 21 | 94 | 58.03 | False | 0.00 | 0.00 |
|  | VanW-B_lnc3 | GCAGCGTGGGCTCGCGTGCCGGGGTTTTTT | ACGGATGCAAGTTG |  | 14 | 94 | 53.96 | False | 0.00 | 0.00 |
|  | VanW-B_detection |  | CCTTAGGTACCGGG | TTT | 14 | 94 | 54.44 | False | 0.00 | 0.00 |
| hmrM | hmrM_fwd |  | GTGTTAGGYGTGAGTATTCCTTT |  | 23 | 160 | 63.69 | False | 0.00 | 0.00 |
|  | hmrM_rev |  | ARCGAAAATTAATCAGCATCA |  | 21 | 160 | 59.27 | False | 0.00 | 0.00 |
|  | hmrM_lnc3 | GCAGCGTGGGCTCGCGTGCCGGGGTTTTTT | ATGGAAAGCAAAATG |  | 15 | 160 | 49.54 | False | 0.00 | 0.00 |
|  | hmrM_detection |  | TCAGATTTAGCACG | TTT | 14 | 160 | 49.26 | False | 0.00 | 0.00 |
| spd | spd_fwd |  | AATTATTTTATTCCAGAAAAGAATA |  | 25 | 154 | 55.69 | False | 43.79 | -822.22 |
|  | spd_rev |  | TTTCCTTTATAGCTTTTTGTAA |  | 22 | 154 | 55.79 | False | 0.00 | 0.00 |
|  | spd_lnc3 | GCAGCGTGGGCTCGCGTGCCGGGGTTTTTT | ATTATATCAAGCCAAGC |  | 17 | 154 | 53.07 | False | 0.00 | 0.00 |
|  | spd_detection |  | TTTCTTCAATATCAATTTAT | TTT | 20 | 154 | 50.09 | False | 0.00 | 0.00 |
| vanHB | vanHB_fwd |  | GGATGTGTTGGAGGGCG |  | 17 | 120 | 63.56 | False | 0.00 | 0.00 |
|  | vanHB_rev |  | GCCGTATGGGGTGTGATG |  | 18 | 120 | 63.84 | False | 38.31 | -71.26 |
|  | vanHB_detection |  | CATCCCTTTTTGTC | TTT | 14 | 120 | 47.73 | False | 0.00 | 0.00 |
|  | vanHB_lnc3 | GCAGCGTGGGCTCGCGTGCCGGGGTTTTTT | AGAAAGTGCTTGAC |  | 14 | 120 | 49.77 | False | 0.00 | 0.00 |
| tmrB | tmrB_fwd |  | AATACTTCAATGAGATCATCG |  | 21 | 124 | 57.22 | False | 39.55 | -157.54 |
|  | tmrB_rev |  | GAGTTTTTTCCTTCTGCTCT |  | 20 | 124 | 59.50 | False | 0.00 | 0.00 |
|  | tmrB_detection |  | GAAACCTTGTTAAAA | TTT | 15 | 124 | 45.56 | False | 0.00 | 0.00 |
|  | tmrB_lnc3 | GCAGCGTGGGCTCGCGTGCCGGGGTTTTTT | TAATGGCTTCAAAG |  | 14 | 124 | 46.20 | False | 0.00 | 0.00 |
| DfrA8 | DfrA8_fwd |  | TGAATTGACCTTTGTTAAGCGAC |  | 23 | 145 | 64.02 | False | 0.00 | 0.00 |
|  | DfrA8_rev |  | TCATTCTGTAAGCTCCTTTTTAC |  | 23 | 145 | 60.82 | False | 0.00 | 0.00 |
|  | DfrA8_detection |  | ACTACGAGCAGAAT | TTT | 14 | 145 | 50.23 | False | 0.00 | 0.00 |
|  | DfrA8_lnc3 | GCAGCGTGGGCTCGCGTGCCGGGGTTTTTT | CGAAATGGTCAAAG |  | 14 | 145 | 48.51 | False | 0.00 | 0.00 |
| Tet-32 | Tet-32_fwd |  | ACCATACCTTTCCTTTACCCTT |  | 22 | 146 | 63.11 | False | 0.00 | 0.00 |
|  | Tet-32_rev |  | TACAACGGGCAGGGATT |  | 17 | 146 | 61.25 | False | 0.00 | 0.00 |
|  | Tet-32_lnc3 | GCAGCGTGGGCTCGCGTGCCGGGGTTTTTT | CAAAGTATTGCGCA |  | 14 | 146 | 51.51 | False | 0.00 | 0.00 |
|  | Tet-32_detection |  | ATCATTGAATCAACCA | TTT | 16 | 146 | 51.04 | False | 0.00 | 0.00 |
| LnuA | LnuA_fwd |  | TGGGGBGTAGATGTATTAAC |  | 20 | 137 | 59.41 | False | 0.00 | 0.00 |
|  | LnuA_rev |  | GGCATCCAATCAACTTCTATTT |  | 22 | 137 | 60.94 | False | 0.00 | 0.00 |
|  | LnuA_detection |  | ATAGATATAGATTTTGAC | TTT | 18 | 137 | 46.64 | False | 0.00 | 0.00 |
|  | LnuA_lnc3 | GCAGCGTGGGCTCGCGTGCCGGGGTTTTTT | AGAGAACACAGAGAT |  | 15 | 137 | 50.46 | False | 0.00 | 0.00 |
| cat | cat_fwd |  | AGAAAAGAATATTTTGAACACTA |  | 23 | 149 | 56.29 | False | 0.00 | 0.00 |
|  | cat_rev |  | TTTACAACTTCCATAATTGCATAA |  | 24 | 149 | 59.49 | False | 0.00 | 0.00 |
|  | cat_detection |  | TTGATATTACTTTGTTT | TTT | 17 | 149 | 46.55 | False | 0.00 | 0.00 |
|  | cat_lnc3 | GCAGCGTGGGCTCGCGTGCCGGGGTTTTTT | TAGCATTACTAAAGAAA |  | 17 | 149 | 48.13 | False | 0.00 | 0.00 |
| OXY2-5 | OXY2-5_fwd |  | GTGCAGCACCAGTAAAG |  | 17 | 92 | 58.79 | False | 0.00 | 0.00 |
|  | OXY2-5_rev |  | CGTTAATCTCCAGCCTTT |  | 18 | 92 | 57.84 | False | 0.00 | 0.00 |
|  | OXY2-5_detection |  | GCAATAAAGAGGTGGT | TTT | 16 | 92 | 54.77 | False | 0.00 | 0.00 |
|  | OXY2-5_lnc3 | GCAGCGTGGGCTCGCGTGCCGGGGTTTTTT | TATTAAAACAGAGCGAAA |  | 18 | 92 | 53.46 | False | 0.00 | 0.00 |
| MexC | MexC_fwd |  | AAGCGCTTCGAGGAGGG |  | 17 | 83 | 65.00 | False | 0.00 | 0.00 |
|  | MexC_rev |  | GACACCGCAGCCTTCAG |  | 17 | 83 | 63.84 | False | 0.00 | 0.00 |
|  | MexC_lnc3 | GCAGCGTGGGCTCGCGTGCCGGGGTTTTTT | ACGTCAAGGCTGG |  | 13 | 83 | 54.91 | False | 0.00 | 0.00 |
|  | MexC_detection |  | CGACCTGCTGTTC | TTT | 13 | 83 | 52.93 | False | 0.00 | 0.00 |
| CMY-10 | CMY-10_fwd |  | GTGGTGGATGCCAGCAT |  | 17 | 149 | 63.20 | True | 63.16 | -2753.61 |
|  | CMY-10_rev |  | AACAGGGTCTGCTCGCT |  | 17 | 149 | 64.15 | False | 38.75 | -109.42 |
|  | CMY-10_lnc3 | GCAGCGTGGGCTCGCGTGCCGGGGTTTTTT | GGCCGTGCTCAA |  | 12 | 149 | 54.71 | False | 0.00 | 0.00 |
|  | CMY-10_detection |  | GGATGGCAAGGC | TTT | 12 | 149 | 51.82 | False | 0.00 | 0.00 |
| OXY6-4 | OXY6-4_fwd |  | GGTTCATTATGGGCCAGT |  | 18 | 230 | 60.27 | False | 0.00 | 0.00 |
|  | OXY6-4_rev |  | ATCTCCAGCCTTTTATTCAC |  | 20 | 230 | 59.05 | False | 0.00 | 0.00 |
|  | OXY6-4_lnc3 | GCAGCGTGGGCTCGCGTGCCGGGGTTTTTT | TTTGCCATGTGCAG |  | 14 | 230 | 54.74 | True | 56.73 | -1345.85 |
|  | OXY6-4_detection |  | CACCGGTAAAGTGAT | TTT | 15 | 230 | 53.36 | True | 40.41 | -235.70 |
| LsaC | LsaC_fwd |  | GTTGATGAAACATTGTGYAA |  | 20 | 248 | 57.51 | False | 42.32 | -414.50 |
|  | LsaC_rev |  | TTATCGTGTTCCACAAATA |  | 19 | 248 | 55.22 | False | 37.76 | -47.39 |
|  | LsaC_lnc3 | GCAGCGTGGGCTCGCGTGCCGGGGTTTTTT | TAGATGTAATATCAAGA |  | 17 | 248 | 46.32 | False | 0.00 | 0.00 |
|  | LsaC_detection |  | ATACAGATTGAGGAA | TTT | 15 | 248 | 47.47 | False | 0.00 | 0.00 |
| ykkC | ykkC_fwd |  | TTAACATGGAGCGGCACT |  | 18 | 261 | 62.62 | False | 0.00 | 0.00 |
|  | ykkC_rev |  | TTATGCCTCGCCTCCT |  | 16 | 261 | 59.85 | False | 0.00 | 0.00 |
|  | ykkC_lnc3 | GCAGCGTGGGCTCGCGTGCCGGGGTTTTTT | TTGAAGCTTGTGAC |  | 14 | 261 | 50.23 | False | 0.00 | 0.00 |
|  | ykkC_detection |  | ACAGGATGAGACAGA | TTT | 15 | 261 | 53.44 | True | 43.01 | -188.24 |
| ykkD | ykkD_fwd |  | ATGCTGCACTGGATCAGTTTA |  | 21 | 122 | 63.88 | True | 49.54 | -761.49 |
|  | ykkD_rev |  | GCAAAACCAACAATGATCAACAG |  | 23 | 122 | 63.74 | False | 0.00 | 0.00 |
|  | ykkD_lnc3 | GCAGCGTGGGCTCGCGTGCCGGGGTTTTTT | CGTGGCCCTTAT |  | 12 | 122 | 49.01 | False | 0.00 | 0.00 |
|  | ykkD_detection |  | GAATCAATATGCGAA | TTT | 15 | 122 | 48.37 | False | 0.00 | 0.00 |
| cphA4 | cphA4_fwd |  | GCGAGCTGCACAAGCT |  | 16 | 192 | 63.62 | True | 69.25 | -2514.50 |
|  | cphA4_rev |  | CCTTGCGGGTAAAGGC |  | 16 | 192 | 60.66 | False | 42.96 | -495.52 |
|  | cphA4_lnc3 | GCAGCGTGGGCTCGCGTGCCGGGGTTTTTT | TGGAGGTGATCAAC |  | 14 | 192 | 51.47 | False | 0.00 | 0.00 |
|  | cphA4_detection |  | ACCAACTACCACAC | TTT | 14 | 192 | 51.82 | False | 0.00 | 0.00 |
| OXA-213 | OXA-213_lnc3 | GCAGCGTGGGCTCGCGTGCCGGGGTTTTTT | TGGGTCGTTCAAC |  | 13 | 146 | 51.52 | False | 0.00 | 0.00 |
|  | OXA-213_detection |  | CACAAGGAGAAATTGT | TTT | 16 | 146 | 52.33 | True | 44.18 | -554.50 |
|  | OXA-213_fwd |  | GGKTGGGGATGGGAT |  | 15 | 146 | 58.04 | False | 0.00 | 0.00 |
|  | OXA-213_rev |  | CCTTTATAAGCAATTTCTTTK |  | 21 | 146 | 54.63 | False | 40.25 | -247.96 |
| ceoA | ceoA_fwd |  | TTCAAGGACGGCGCG |  | 15 | 281 | 62.63 | False | 0.00 | 0.00 |
|  | ceoA_rev |  | TATAGCCGAGRTTGATGCG |  | 19 | 281 | 61.87 | False | 0.00 | 0.00 |
|  | ceoA_lnc3 | GCAGCGTGGGCTCGCGTGCCGGGGTTTTTT | GCTGATCGGCG |  | 11 | 281 | 50.87 | False | 0.00 | 0.00 |
|  | ceoA_detection |  | ACAACGCGATCG | TTT | 12 | 281 | 51.76 | True | 42.43 | -374.84 |

### ABR-8

Supplementary Table S11: Primer and probe sequences and additional information of ABR-8.

| Gene | Primer/Probe | Hybridisation Sequence | Sequence | Spacer | Length | Product Size | Tm | Hairpin | Hairpin Tm | Hairpin delta G |
| --- | --- | --- | --- | --- | --- | --- | --- | --- | --- | --- |
| tet(H) | tet(H)_fwd |  | CATCTTGCTGTTTTCCCTTTT |  | 21 | 105 | 61.33 | False | 0.00 | 0.00 |
|  | tet(H)_rev |  | GTGATCCCCGCAATGAT |  | 17 | 105 | 60.03 | False | 0.00 | 0.00 |
|  | tet(H)_detection |  | CACACTTTGGATGCT | TTT | 15 | 105 | 54.52 | False | 0.00 | 0.00 |
|  | tet(H)_lnc3 | GCAGCGTGGGCTCGCGTGCCGGGGTTTTTT | TTAATGGCATTCTCAAC |  | 17 | 105 | 53.63 | False | 0.00 | 0.00 |
| pgpB | pgpB_fwd |  | CCTCTTCTTTTTTCAAGCCTTTC |  | 23 | 129 | 62.65 | False | 0.00 | 0.00 |
|  | pgpB_rev |  | TCGTCGTATGCCCTGAGAT |  | 19 | 129 | 64.01 | False | 0.00 | 0.00 |
|  | pgpB_detection |  | ACTATGTGAAGACGG | TTT | 15 | 129 | 52.61 | False | 0.00 | 0.00 |
|  | pgpB_lnc3 | GCAGCGTGGGCTCGCGTGCCGGGGTTTTTT | ACCCTGATTTTATCG |  | 15 | 129 | 50.09 | False | 0.00 | 0.00 |
| MefB | MefB_fwd |  | CTTATGTTCAAGAAAGCATTGC |  | 22 | 116 | 60.65 | False | 0.00 | 0.00 |
|  | MefB_rev |  | CTCAACAACCGGACCTG |  | 17 | 116 | 60.54 | False | 0.00 | 0.00 |
|  | MefB_detection |  | CGCCATGACTCTTT | TTT | 14 | 116 | 53.10 | False | 0.00 | 0.00 |
|  | MefB_lnc3 | GCAGCGTGGGCTCGCGTGCCGGGGTTTTTT | TTTCCCTTTTGATGAC |  | 16 | 116 | 52.29 | False | 0.00 | 0.00 |
| AadC | AadC_fwd |  | TAGATAAGCTATACCGTAAACAAA |  | 24 | 156 | 59.61 | False | 0.00 | 0.00 |
|  | AadC_rev |  | TTGCTATCATTGTGTTATTAC |  | 21 | 156 | 55.62 | False | 0.00 | 0.00 |
|  | AadC_detection |  | TAGGCGGTAAAAAGC | TTT | 15 | 156 | 54.27 | False | 0.00 | 0.00 |
|  | AadC_lnc3 | GCAGCGTGGGCTCGCGTGCCGGGGTTTTTT | ATAAGTATGTTAGATATGA |  | 19 | 156 | 48.71 | False | 0.00 | 0.00 |
| tet(40) | tet(40)_fwd |  | GGTGTTATCAGTCTTATCG |  | 19 | 97 | 55.94 | False | 0.00 | 0.00 |
|  | tet(40)_rev |  | CACTGGTTCGGCTGACA |  | 17 | 97 | 62.64 | False | 0.00 | 0.00 |
|  | tet(40)_lnc3 | GCAGCGTGGGCTCGCGTGCCGGGGTTTTTT | TAGGTATTCTGGCT |  | 14 | 97 | 48.48 | False | 0.00 | 0.00 |
|  | tet(40)_detection |  | TCTCAGTTGCTCAT | TTT | 14 | 97 | 50.44 | False | 0.00 | 0.00 |
| DfrA31 | DfrA31_fwd |  | TTTCTGGTGGTGGTGAAATW |  | 20 | 96 | 61.13 | False | 0.00 | 0.00 |
|  | DfrA31_rev |  | AAAACACRTCGCCAGAGA |  | 18 | 96 | 61.46 | False | 0.00 | 0.00 |
|  | DfrA31_detection |  | ATCTTTCAGTGATTC | TTT | 15 | 96 | 47.06 | False | 0.00 | 0.00 |
|  | DfrA31_lnc3 | GCAGCGTGGGCTCGCGTGCCGGGGTTTTTT | AGCAGATGTTATCC |  | 14 | 96 | 48.12 | False | 0.00 | 0.00 |
| clbA | clbA_fwd |  | TGCAACAGGAGATATTGGTTT |  | 21 | 126 | 61.70 | False | 0.00 | 0.00 |
|  | clbA_rev |  | AATGCTTCTCCCATTCCCAT |  | 20 | 126 | 63.18 | False | 0.00 | 0.00 |
|  | clbA_lnc3 | GCAGCGTGGGCTCGCGTGCCGGGGTTTTTT | ACTGACCAAATTTT |  | 14 | 126 | 45.58 | False | 0.00 | 0.00 |
|  | clbA_detection |  | GTACTTTCACTTAAAA | TTT | 16 | 126 | 46.08 | False | 0.00 | 0.00 |
| lmrD | lmrD_fwd |  | CATGCAAATTTCATCAAACTTTGG |  | 24 | 168 | 62.64 | False | 0.00 | 0.00 |
|  | lmrD_rev |  | GGAAGATATTCAAAATCAAGATT |  | 23 | 168 | 57.09 | False | 0.00 | 0.00 |
|  | lmrD_lnc3 | GCAGCGTGGGCTCGCGTGCCGGGGTTTTTT | GTTTGATGAACCAGAA |  | 16 | 168 | 52.35 | False | 34.62 | 173.89 |
|  | lmrD_detection |  | GAAGTTCGTCCTGAA | TTT | 15 | 168 | 53.32 | False | 0.00 | 0.00 |
| FosA | FosA_fwd |  | GCTGCAGGGATTGAATCATC |  | 20 | 129 | 62.97 | False | 0.00 | 0.00 |
|  | FosA_rev |  | GCCCCACAGGAGAGATAGG |  | 19 | 129 | 64.75 | False | 41.66 | -189.30 |
|  | FosA_lnc3 | GCAGCGTGGGCTCGCGTGCCGGGGTTTTTT | CTGGCATTTTATCAGC |  | 16 | 129 | 54.02 | False | 0.00 | 0.00 |
|  | FosA_detection |  | AGTTACCTGGAATGC | TTT | 15 | 129 | 53.27 | False | 0.00 | 0.00 |
| Aac6-Ic | Aac6-Ic_fwd |  | GACGGCRCTTTCGTT |  | 15 | 160 | 58.38 | False | 45.00 | -593.07 |
|  | Aac6-Ic_rev |  | CCCACTCCTGYACCTG |  | 16 | 160 | 59.98 | False | 0.00 | 0.00 |
|  | Aac6-Ic_lnc3 | GCAGCGTGGGCTCGCGTGCCGGGGTTTTTT | GTCAACGGCTGC |  | 12 | 160 | 53.07 | False | 0.00 | 0.00 |
|  | Aac6-Ic_detection |  | GAATCGTCGCCG | TTT | 12 | 160 | 52.54 | False | 0.00 | 0.00 |
| CatB2 | CatB2_fwd |  | AGTTATAGGAAGTGATGTGTG |  | 21 | 120 | 58.81 | False | 0.00 | 0.00 |
|  | CatB2_rev |  | GTGTAGGGTTCCACGTCTT |  | 19 | 120 | 62.77 | False | 0.00 | 0.00 |
|  | CatB2_detection |  | ATGGAGCGGTGATA | TTT | 14 | 120 | 53.39 | False | 0.00 | 0.00 |
|  | CatB2_lnc3 | GCAGCGTGGGCTCGCGTGCCGGGGTTTTTT | GATCAAGATCGGGC |  | 14 | 120 | 53.48 | False | 0.00 | 0.00 |
| QnrVC1 | QnrVC1_fwd |  | ATATAATCAAGTGAACTTCTCA |  | 22 | 131 | 56.52 | False | 37.60 | -36.67 |
|  | QnrVC1_rev |  | GCTCTATGAAAGTACAGTTAAT |  | 22 | 131 | 57.52 | False | 0.00 | 0.00 |
|  | QnrVC1_lnc3 | GCAGCGTGGGCTCGCGTGCCGGGGTTTTTT | ATATCTTTAGCAATTGTA |  | 18 | 131 | 49.56 | False | 0.00 | 0.00 |
|  | QnrVC1_detection |  | CTTTTATACATTGTAATTT | TTT | 19 | 131 | 47.83 | False | 0.00 | 0.00 |
| vgaE | vgaE_fwd |  | GTGAGGATAGGTTATTTTTCCCA |  | 23 | 122 | 62.27 | False | 0.00 | 0.00 |
|  | vgaE_rev |  | ATTCTTGCTAAAACTGTTCTAATTA |  | 25 | 122 | 59.50 | False | 0.00 | 0.00 |
|  | vgaE_detection |  | GATAAAACAATCATAGAA | TTT | 18 | 122 | 48.34 | False | 0.00 | 0.00 |
|  | vgaE_lnc3 | GCAGCGTGGGCTCGCGTGCCGGGGTTTTTT | CACAATTCTCGATGAT |  | 16 | 122 | 51.98 | False | 0.00 | 0.00 |
| tetA(P) | tetA(P)_fwd |  | GTCGTAAACTATCTATTGTTATTGG |  | 25 | 114 | 60.04 | False | 0.00 | 0.00 |
|  | tetA(P)_rev |  | CTAATCCCCATACAATCTGTGC |  | 22 | 114 | 62.79 | False | 0.00 | 0.00 |
|  | tetA(P)_detection |  | GAAGGTTCTATTTCTA | TTT | 16 | 114 | 46.85 | False | 0.00 | 0.00 |
|  | tetA(P)_lnc3 | GCAGCGTGGGCTCGCGTGCCGGGGTTTTTT | AGTGGGATTTATTTTA |  | 16 | 114 | 46.83 | False | 0.00 | 0.00 |
| Aac3-VIa | Aac3-VIa_fwd |  | TCACCGCGCTCCATTATG |  | 18 | 147 | 63.50 | False | 0.00 | 0.00 |
|  | Aac3-VIa_rev |  | CGGCATATTCGTCGAGGATG |  | 20 | 147 | 64.59 | False | 40.85 | -200.86 |
|  | Aac3-VIa_detection |  | GATGCCCTTACTGC | TTT | 14 | 147 | 53.73 | False | 0.00 | 0.00 |
|  | Aac3-VIa_lnc3 | GCAGCGTGGGCTCGCGTGCCGGGGTTTTTT | CGCGTCACTTATTC |  | 14 | 147 | 51.54 | False | 0.00 | 0.00 |
| OXA-235 | OXA-235_fwd |  | CAATGAAATYTTTAAATGGGAT |  | 22 | 99 | 56.28 | False | 38.51 | -56.21 |
|  | OXA-235_rev |  | ACSGGTACAGCAGAAGC |  | 17 | 99 | 62.39 | False | 42.56 | -313.74 |
|  | OXA-235_detection |  | GACATGACTCTCGG | TTT | 14 | 99 | 52.50 | False | 0.00 | 0.00 |
|  | OXA-235_lnc3 | GCAGCGTGGGCTCGCGTGCCGGGGTTTTTT | CAGCCTGGGAAAAA |  | 14 | 99 | 53.13 | False | 0.00 | 0.00 |
| lmrC | lmrC_fwd |  | ATTATTATTGCTCAAAAGATTTC |  | 23 | 104 | 55.76 | False | 0.00 | 0.00 |
|  | lmrC_rev |  | GCAACTAATTCTTGGTGAGTTCC |  | 23 | 104 | 64.10 | False | 0.00 | 0.00 |
|  | lmrC_detection |  | TATTTTGGTTCTTGA | TTT | 15 | 104 | 46.09 | False | 0.00 | 0.00 |
|  | lmrC_lnc3 | GCAGCGTGGGCTCGCGTGCCGGGGTTTTTT | GTTCATGCAGATAA |  | 14 | 104 | 45.99 | False | 0.00 | 0.00 |
| IMP-1 | IMP-1_fwd |  | CACACTCAAGATAACGTAG |  | 19 | 120 | 56.20 | False | 0.00 | 0.00 |
|  | IMP-1_rev |  | CTTTGGCCAAGCTTCTAAAT |  | 20 | 120 | 60.23 | False | 0.00 | 0.00 |
|  | IMP-1_lnc3 | GCAGCGTGGGCTCGCGTGCCGGGGTTTTTT | TACCTGAAAAGAAAA |  | 15 | 120 | 45.99 | False | 0.00 | 0.00 |
|  | IMP-1_detection |  | TTTTATTCGGTGGT | TTT | 14 | 120 | 48.13 | False | 0.00 | 0.00 |
| SRT-1 | SRT-1_fwd |  | GACGAAAATGAACCGCCTG |  | 19 | 120 | 63.20 | False | 0.00 | 0.00 |
|  | SRT-1_rev |  | ACGCCRTATTTTTTCATCA |  | 19 | 120 | 57.94 | False | 0.00 | 0.00 |
|  | SRT-1_detection |  | GACGCCGTTATTCA | TTT | 14 | 120 | 53.33 | False | 0.00 | 0.00 |
|  | SRT-1_lnc3 | GCAGCGTGGGCTCGCGTGCCGGGGTTTTTT | AGCAGCAGGATATC |  | 14 | 120 | 51.38 | False | 0.00 | 0.00 |
| mecR1 | mecR1_fwd |  | TGGTCAACAATCATCTGTTACGAA |  | 24 | 132 | 64.95 | False | 42.99 | -447.30 |
|  | mecR1_rev |  | GCCTTAATCATATAAAATAAGCA |  | 23 | 132 | 56.97 | False | 0.00 | 0.00 |
|  | mecR1_lnc3 | GCAGCGTGGGCTCGCGTGCCGGGGTTTTTT | CGTATATGATTCTTTTGA |  | 18 | 132 | 51.00 | False | 0.00 | 0.00 |
|  | mecR1_detection |  | TATGGGTATTTGGTAGTT | TTT | 18 | 132 | 53.97 | False | 0.00 | 0.00 |
| AAC(6')-IIa | AAC(6')-IIa_fwd |  | ATCGTTGAGTGGTGGGGT |  | 18 | 98 | 64.31 | False | 0.00 | 0.00 |
|  | AAC(6')-IIa_rev |  | GGTGTTACGGACTCTTC |  | 17 | 98 | 57.03 | False | 0.00 | 0.00 |
|  | AAC(6')-IIa_lnc3 | GCAGCGTGGGCTCGCGTGCCGGGGTTTTTT | GACCGACTCTTGAT |  | 14 | 98 | 51.30 | False | 0.00 | 0.00 |
|  | AAC(6')-IIa_detection |  | GAAGTGCTGGAACA | TTT | 14 | 98 | 52.84 | False | 0.00 | 0.00 |
| ACC-1 | ACC-1_fwd |  | CAGGAAAATGTGTGGATTAATA |  | 22 | 96 | 58.31 | False | 0.00 | 0.00 |
|  | ACC-1_rev |  | CAGCATCACGATCCCCA |  | 17 | 96 | 62.16 | False | 0.00 | 0.00 |
|  | ACC-1_lnc3 | GCAGCGTGGGCTCGCGTGCCGGGGTTTTTT | GGATCAACTAACGGC |  | 15 | 96 | 54.94 | False | 0.00 | 0.00 |
|  | ACC-1_detection |  | TTCGGTGCCTATATTG | TTT | 16 | 96 | 54.28 | False | 0.00 | 0.00 |
| PER-1 | PER-1_fwd |  | CTGCTGCAATACTCGGTCTC |  | 20 | 121 | 64.42 | False | 0.00 | 0.00 |
|  | PER-1_rev |  | CAGCGGTCTCCTTTATAC |  | 18 | 121 | 57.72 | False | 0.00 | 0.00 |
|  | PER-1_lnc3 | GCAGCGTGGGCTCGCGTGCCGGGGTTTTTT | GATAACGTGGCCTG |  | 14 | 121 | 53.81 | False | 0.00 | 0.00 |
|  | PER-1_detection |  | TGATTTGTTATTTGAAC | TTT | 17 | 121 | 48.77 | False | 0.00 | 0.00 |
| BlaA2 | BlaA2_fwd |  | ATAAAACCCCTATGGCTGT |  | 19 | 148 | 59.53 | False | 40.85 | -176.52 |
|  | BlaA2_rev |  | TTTTGTAAAACTCCWGTTGG |  | 20 | 148 | 57.81 | False | 42.33 | -380.10 |
|  | BlaA2_lnc3 | GCAGCGTGGGCTCGCGTGCCGGGGTTTTTT | AAAAAAGGCGAATTA |  | 15 | 148 | 48.09 | False | 0.00 | 0.00 |
|  | BlaA2_detection |  | AAAAGAGAAACTATTGT | TTT | 17 | 148 | 49.09 | False | 0.00 | 0.00 |
| ACT-28 | ACT-28_fwd |  | TTCAAGCCACTCAAACTGGAC |  | 21 | 111 | 64.87 | False | 0.00 | 0.00 |
|  | ACT-28_rev |  | CATTCCCGGTGAAACGT |  | 17 | 111 | 60.54 | False | 41.92 | -179.52 |
|  | ACT-28_detection |  | AAAGAAGAAGAGGC | TTT | 14 | 111 | 48.78 | False | 0.00 | 0.00 |
|  | ACT-28_lnc3 | GCAGCGTGGGCTCGCGTGCCGGGGTTTTTT | GGATTAACGTTCCG |  | 14 | 111 | 50.88 | False | 0.00 | 0.00 |
| TriA | TriA_fwd |  | GTGCCGATCTTCACCCTG |  | 18 | 110 | 63.75 | False | 0.00 | 0.00 |
|  | TriA_rev |  | AGCAGGCTGACGGTGAT |  | 17 | 110 | 64.24 | False | 0.00 | 0.00 |
|  | TriA_detection |  | GAGTCGTTGTTCAG | TTT | 14 | 110 | 50.71 | False | 0.00 | 0.00 |
|  | TriA_lnc3 | GCAGCGTGGGCTCGCGTGCCGGGGTTTTTT | TGTTCAACGTCTAC |  | 14 | 110 | 49.11 | False | 0.00 | 0.00 |
| DfrA21 | DfrA21_fwd |  | AGTGGTCGTTATGGGCC |  | 17 | 124 | 62.09 | False | 0.00 | 0.00 |
|  | DfrA21_rev |  | CAGCGTTGAAACAACTGCA |  | 19 | 124 | 63.29 | False | 0.00 | 0.00 |
|  | DfrA21_detection |  | CCCTTACCAAACCG | TTT | 14 | 124 | 53.50 | False | 0.00 | 0.00 |
|  | DfrA21_lnc3 | GCAGCGTGGGCTCGCGTGCCGGGGTTTTTT | GAGTCCATAGGCAAG |  | 15 | 124 | 53.58 | False | 0.00 | 0.00 |
| VanC2 | VanC2_fwd |  | GGCTTTTGGHTAAGTGAAG |  | 19 | 86 | 58.50 | False | 44.18 | -538.75 |
|  | VanC2_rev |  | CTGCCATCTTCCCCRTATT |  | 19 | 86 | 61.72 | False | 0.00 | 0.00 |
|  | VanC2_detection |  | TTTATTTCCCATTATGC | TTT | 17 | 86 | 50.68 | False | 0.00 | 0.00 |
|  | VanC2_lnc3 | GCAGCGTGGGCTCGCGTGCCGGGGTTTTTT | GTTGGTACCTGATGT |  | 15 | 86 | 52.78 | False | 0.00 | 0.00 |
| AphA15 | AphA15_fwd |  | TTTCACCCTTGTTGGATGAAT |  | 21 | 115 | 61.93 | False | 41.05 | -208.67 |
|  | AphA15_rev |  | GAGGCGTACTTTAGATACG |  | 19 | 115 | 58.31 | True | 49.21 | -1253.80 |
|  | AphA15_detection |  | ATTTCCTGATCGCAA | TTT | 15 | 115 | 53.28 | False | 0.00 | 0.00 |
|  | AphA15_lnc3 | GCAGCGTGGGCTCGCGTGCCGGGGTTTTTT | AGTGATTCGAGTTCT |  | 15 | 115 | 51.50 | False | 0.00 | 0.00 |
| GOB-5 | GOB-5_fwd |  | TGAACCMTTCAGAATTGCAGG |  | 21 | 100 | 63.82 | False | 42.04 | -456.99 |
|  | GOB-5_rev |  | TGTATTAATRAGAATATTGCCTT |  | 23 | 100 | 56.78 | False | 37.99 | -96.09 |
|  | GOB-5_detection |  | CTTACCTTATTGTGA | TTT | 15 | 100 | 46.10 | False | 0.00 | 0.00 |
|  | GOB-5_lnc3 | GCAGCGTGGGCTCGCGTGCCGGGGTTTTTT | CTATGATTTGGCTT |  | 14 | 100 | 45.80 | False | 0.00 | 0.00 |
| RmtC | RmtC_fwd |  | ATCTTCTACTTATGTTCAAGCTAT |  | 24 | 93 | 59.61 | False | 0.00 | 0.00 |
|  | RmtC_rev |  | CATTTTTGTATTTCCACTCTT |  | 21 | 93 | 56.29 | False | 0.00 | 0.00 |
|  | RmtC_detection |  | GCCTCTGGTTTGAAA | TTT | 15 | 93 | 54.39 | False | 0.00 | 0.00 |
|  | RmtC_lnc3 | GCAGCGTGGGCTCGCGTGCCGGGGTTTTTT | ATCGGCAGGAAGAG |  | 14 | 93 | 54.87 | False | 0.00 | 0.00 |
| Aph6-I | Aph6-I_fwd |  | AGAACGTGCTCGACTTCG |  | 18 | 211 | 63.28 | False | 42.43 | -418.45 |
|  | Aph6-I_rev |  | CAGCGMAGAAGCCGTTC |  | 17 | 211 | 63.32 | False | 40.36 | -198.05 |
|  | Aph6-I_lnc3 | GCAGCGTGGGCTCGCGTGCCGGGGTTTTTT | CACCTTCGACTATG |  | 14 | 211 | 49.45 | False | 0.00 | 0.00 |
|  | Aph6-I_detection |  | CCAACATCTTCACG | TTT | 14 | 211 | 51.14 | False | 0.00 | 0.00 |
| RmtB | RmtB_fwd |  | GCATAAATCCCCCAAACAG |  | 19 | 139 | 60.02 | False | 0.00 | 0.00 |
|  | RmtB_rev |  | CAGCGACAATGCCTTTTTTACAT |  | 23 | 139 | 64.89 | False | 37.27 | -14.02 |
|  | RmtB_detection |  | CGGAATCGCTCAAG | TTT | 14 | 139 | 54.42 | False | 0.00 | 0.00 |
|  | RmtB_lnc3 | GCAGCGTGGGCTCGCGTGCCGGGGTTTTTT | GGCATATGTCACCC |  | 14 | 139 | 53.26 | False | 0.00 | 0.00 |
| QnrB38 | QnrB38_fwd |  | GCAAAYTTCACGCACTG |  | 17 | 85 | 59.18 | False | 0.00 | 0.00 |
|  | QnrB38_rev |  | CCAATTTGACRCCTTGTAAAT |  | 21 | 85 | 60.06 | False | 0.00 | 0.00 |
|  | QnrB38_lnc3 | GCAGCGTGGGCTCGCGTGCCGGGGTTTTTT | TGACCAATTCAGAAC |  | 15 | 85 | 50.71 | False | 0.00 | 0.00 |
|  | QnrB38_detection |  | TGGGTGATTTAGATATT | TTT | 17 | 85 | 50.33 | False | 0.00 | 0.00 |
| CTX-M-39 | CTX-M-39_fwd |  | ACGTCAATGGCACGATGAC |  | 19 | 121 | 64.49 | False | 44.74 | -924.67 |
|  | CTX-M-39_rev |  | GCAAATGCCGTCACTTT |  | 17 | 121 | 59.45 | False | 36.61 | 22.91 |
|  | CTX-M-39_detection |  | GCCATGAATAAGCTG | TTT | 15 | 121 | 52.51 | False | 0.00 | 0.00 |
|  | CTX-M-39_lnc3 | GCAGCGTGGGCTCGCGTGCCGGGGTTTTTT | GTACAGCGATAATACT |  | 16 | 121 | 50.52 | False | 0.00 | 0.00 |
| tetX | tetX_fwd |  | AAATTTAAYAGCATTGAAGAGGC |  | 23 | 137 | 61.17 | False | 36.94 | 0.00 |
|  | tetX_rev |  | TTTAACAATTGCTGAAACGTAAAGT |  | 25 | 137 | 62.91 | False | 0.00 | 0.00 |
|  | tetX_detection |  | TCAACTCAAAACGA | TTT | 14 | 137 | 48.68 | False | 0.00 | 0.00 |
|  | tetX_lnc3 | GCAGCGTGGGCTCGCGTGCCGGGGTTTTTT | AAGCACAAGAAGAA |  | 14 | 137 | 48.49 | False | 0.00 | 0.00 |
| dfrF | dfrF_fwd |  | TATCAAGGAGATAATTTAGTTTCAG |  | 25 | 157 | 58.91 | False | 41.46 | -356.98 |
|  | dfrF_rev |  | CAATATTTAAATCTACTTCTGTGAT |  | 25 | 157 | 57.67 | False | 0.00 | 0.00 |
|  | dfrF_detection |  | TGTATACATATCTGGT | TTT | 16 | 157 | 47.91 | False | 0.00 | 0.00 |
|  | dfrF_lnc3 | GCAGCGTGGGCTCGCGTGCCGGGGTTTTTT | GCTAAAGGACGAGA |  | 14 | 157 | 51.33 | False | 0.00 | 0.00 |
| OXA-421 | OXA-421_fwd |  | ARCTCTTTTCTTTGCCATTG |  | 20 | 134 | 59.68 | False | 0.00 | 0.00 |
|  | OXA-421_rev |  | TTTGTGCTTGATCAAACA |  | 18 | 134 | 56.13 | False | 0.00 | 0.00 |
|  | OXA-421_lnc3 | GCAGCGTGGGCTCGCGTGCCGGGGTTTTTT | AATACAGTCAATTTCT |  | 16 | 134 | 47.39 | False | 0.00 | 0.00 |
|  | OXA-421_detection |  | ACCAATAAAAACTCAG | TTT | 16 | 134 | 49.18 | False | 0.00 | 0.00 |
| AadA7 | AadA7_fwd |  | TTCAGCAGCGAAGGATCT |  | 18 | 135 | 61.97 | False | 0.00 | 0.00 |
|  | AadA7_rev |  | CGAGACAAAGTAAGCACT |  | 18 | 135 | 57.80 | False | 38.87 | -96.26 |
|  | AadA7_lnc3 | GCAGCGTGGGCTCGCGTGCCGGGGTTTTTT | ATCTATTCAAGGCAC |  | 15 | 135 | 50.05 | False | 0.00 | 0.00 |
|  | AadA7_detection |  | TGGCCGATACTCTG | TTT | 14 | 135 | 54.48 | False | 0.00 | 0.00 |
| DfrB5 | DfrB5_fwd |  | TGAAGTCAGTAATCCAGT |  | 18 | 169 | 55.47 | False | 0.00 | 0.00 |
|  | DfrB5_rev |  | GTGAGCCTCAGACTCGA |  | 17 | 169 | 60.96 | False | 40.71 | -272.21 |
|  | DfrB5_lnc3 | GCAGCGTGGGCTCGCGTGCCGGGGTTTTTT | ATGGGAGATCGCG |  | 13 | 169 | 54.40 | False | 0.00 | 0.00 |
|  | DfrB5_detection |  | TGCGCAAGAAATC | TTT | 13 | 169 | 50.05 | True | 43.02 | -184.60 |
| OXA-134 | OXA-134_fwd |  | GGTTGGGGCATGGATGT |  | 17 | 168 | 62.81 | False | 0.00 | 0.00 |
|  | OXA-134_rev |  | ATAAAGCCCTAATTCGGCC |  | 19 | 168 | 61.10 | True | 52.59 | -1081.79 |
|  | OXA-134_lnc3 | GCAGCGTGGGCTCGCGTGCCGGGGTTTTTT | GGCTGGCTCACC |  | 12 | 168 | 54.80 | False | 0.00 | 0.00 |
|  | OXA-134_detection |  | GGCTGGGTTGAAAC | TTT | 14 | 168 | 54.75 | False | 0.00 | 0.00 |
| opcM | opcM_fwd |  | TGCCGCTGTTCGACG |  | 15 | 280 | 62.61 | False | 44.20 | -399.08 |
|  | opcM_rev |  | TGCGACTGCARCACC |  | 15 | 280 | 61.12 | True | 55.78 | -1164.49 |
|  | opcM_lnc3 | GCAGCGTGGGCTCGCGTGCCGGGGTTTTTT | CTCGTCGCGTTC |  | 12 | 280 | 52.51 | False | 0.00 | 0.00 |
|  | opcM_detection |  | CGCGAGGTCGAG | TTT | 12 | 280 | 54.87 | True | 44.81 | -459.12 |
| smeD | smeD_fwd |  | CGGTCAGCCAGCAGGA |  | 16 | 307 | 64.43 | False | 39.89 | -133.10 |
|  | smeD_rev |  | TGGTTGTCCTGCAGGC |  | 16 | 307 | 62.39 | False | 36.69 | 19.15 |
|  | smeD_lnc3 | GCAGCGTGGGCTCGCGTGCCGGGGTTTTTT | ATCAGCGGCCG |  | 11 | 307 | 53.07 | False | 0.00 | 0.00 |
|  | smeD_detection |  | CATCGGCAAGTCC | TTT | 13 | 307 | 52.97 | False | 0.00 | 0.00 |
| Cat-86 | Cat-86_fwd |  | TAACCCGATGCWCATATAG |  | 19 | 389 | 57.24 | False | 0.00 | 0.00 |
|  | Cat-86_rev |  | AAAAGAAGTRAAATCAATCCA |  | 21 | 389 | 56.44 | False | 0.00 | 0.00 |
|  | Cat-86_lnc3 | GCAGCGTGGGCTCGCGTGCCGGGGTTTTTT | AAAGTATATCCTGTG |  | 15 | 389 | 45.73 | False | 0.00 | 0.00 |
|  | Cat-86_detection |  | CAAATTTATTTGTTAGC | TTT | 17 | 389 | 47.69 | True | 44.54 | -799.50 |
| VanR-D | VanR-D_fwd |  | GTTTCTACGGAGGAATTATT |  | 20 | 116 | 56.26 | False | 0.00 | 0.00 |
|  | VanR-D_rev |  | TTTCTTGACGGTTCCTTCATTTT |  | 23 | 116 | 63.40 | False | 0.00 | 0.00 |
|  | VanR-D_lnc3 | GCAGCGTGGGCTCGCGTGCCGGGGTTTTTT | ACAGCAATAATACY |  | 14 | 116 | 44.53 | False | 0.00 | 0.00 |
|  | VanR-D_detection |  | GTGATGGCGCATAT | TTT | 14 | 116 | 53.42 | False | 0.00 | 0.00 |

### ABR-9

Supplementary Table S12: Primer and probe sequences and additional information of ABR-9.

| Gene | Primer/Probe | Hybridisation Sequence | Sequence | Spacer | Length | Product Size | Tm | Hairpin | Hairpin Tm | Hairpin delta G |
| --- | --- | --- | --- | --- | --- | --- | --- | --- | --- | --- |
| aadA16 | aadA16_fwd |  | CCCGGAAAGCGATCTAT |  | 17 | 112 | 59.01 | False | 0.00 | 0.00 |
|  | aadA16_rev |  | CCAAATGCGAGACAAAGT |  | 18 | 112 | 59.15 | False | 0.00 | 0.00 |
|  | aadA16_lnc3 | GCAGCGTGGGCTCGCGTGCCGGGGTTTTTT | CCTTGAAACTATGGAA |  | 16 | 112 | 51.13 | False | 0.00 | 0.00 |
|  | aadA16_detection |  | CTCACAACCGGATT | TTT | 14 | 112 | 52.52 | False | 0.00 | 0.00 |
| SME-2 | SME-2_fwd |  | GGCAAAACAAATAAAAAAATTAG |  | 23 | 126 | 56.22 | False | 0.00 | 0.00 |
|  | SME-2_rev |  | AATGAACTGCATAAAGGGAA |  | 20 | 126 | 58.88 | False | 40.69 | -206.79 |
|  | SME-2_detection |  | TTTGGGTATAGATCAGA | TTT | 17 | 126 | 52.22 | False | 0.00 | 0.00 |
|  | SME-2_lnc3 | GCAGCGTGGGCTCGCGTGCCGGGGTTTTTT | AGGATCGGGTAATACA |  | 16 | 126 | 54.36 | False | 0.00 | 0.00 |
| TetB-P | TetB-P_fwd |  | GGAAGCAAAGCTGTTTAT |  | 18 | 158 | 56.39 | False | 44.59 | -425.40 |
|  | TetB-P_rev |  | TATAATGAAAGCTTTTCTTGTA |  | 22 | 158 | 55.32 | False | 38.79 | -119.20 |
|  | TetB-P_detection |  | GAAGCATTTTTAGAAAGAT | TTT | 19 | 158 | 53.16 | False | 0.00 | 0.00 |
|  | TetB-P_lnc3 | GCAGCGTGGGCTCGCGTGCCGGGGTTTTTT | ATGTTTTATCAGACTTAGAC |  | 20 | 158 | 54.71 | False | 0.00 | 0.00 |
| ApmA | ApmA_fwd |  | GGCGAACTTCCGTTTGAAC |  | 19 | 106 | 63.41 | False | 41.48 | -394.39 |
|  | ApmA_rev |  | TGCTCTTGATATACCCG |  | 17 | 106 | 55.67 | False | 0.00 | 0.00 |
|  | ApmA_detection |  | GCAGGCAAACGTAT | TTT | 14 | 106 | 54.07 | False | 0.00 | 0.00 |
|  | ApmA_lnc3 | GCAGCGTGGGCTCGCGTGCCGGGGTTTTTT | CAATGTCCCCGTTG |  | 14 | 106 | 54.49 | False | 0.00 | 0.00 |
| NMC-A | NMC-A_fwd |  | AGCGACAATGGTGCTACTAATAT |  | 23 | 119 | 64.04 | False | 41.52 | -286.11 |
|  | NMC-A_rev |  | TCTAACTCCCAACGATCGA |  | 19 | 119 | 61.25 | False | 0.00 | 0.00 |
|  | NMC-A_detection |  | GGTCGATTGGAGAT | TTT | 14 | 119 | 51.31 | False | 0.00 | 0.00 |
|  | NMC-A_lnc3 | GCAGCGTGGGCTCGCGTGCCGGGGTTTTTT | TGACTAAATTCATGC |  | 15 | 119 | 48.05 | False | 0.00 | 0.00 |
| LnuF | LnuF_fwd |  | TCCCCCGAWACGTGAAG |  | 17 | 99 | 61.68 | False | 0.00 | 0.00 |
|  | LnuF_rev |  | TACTCTCCCCGATTTAAAA |  | 19 | 99 | 56.47 | False | 0.00 | 0.00 |
|  | LnuF_detection |  | GATGCTCTTTGGGG | TTT | 14 | 99 | 53.55 | False | 0.00 | 0.00 |
|  | LnuF_lnc3 | GCAGCGTGGGCTCGCGTGCCGGGGTTTTTT | AACCTCATCAGCCT |  | 14 | 99 | 53.50 | False | 0.00 | 0.00 |
| HERA-7 | HERA-7_fwd |  | GATTATCGCGGCGGTCTA |  | 18 | 94 | 62.62 | False | 0.00 | 0.00 |
|  | HERA-7_rev |  | TTCATTTGCCATTTTCATYGAGG |  | 23 | 94 | 63.58 | False | 0.00 | 0.00 |
|  | HERA-7_lnc3 | GCAGCGTGGGCTCGCGTGCCGGGGTTTTTT | CCGCCGTTTTATGT |  | 14 | 94 | 54.09 | False | 0.00 | 0.00 |
|  | HERA-7_detection |  | CGCGATTTTTATTACG | TTT | 16 | 94 | 52.20 | False | 0.00 | 0.00 |
| lnuC | lnuC_fwd |  | ATGGTCAATATAACAGATGT |  | 20 | 119 | 55.20 | False | 0.00 | 0.00 |
|  | lnuC_rev |  | TGGGCTCTTGACTGATAT |  | 18 | 119 | 58.10 | False | 0.00 | 0.00 |
|  | lnuC_detection |  | CGGAGATTAAAGTC | TTT | 14 | 119 | 46.37 | False | 0.00 | 0.00 |
|  | lnuC_lnc3 | GCAGCGTGGGCTCGCGTGCCGGGGTTTTTT | AATTTGCAATAGATG |  | 15 | 119 | 45.54 | False | 0.00 | 0.00 |
| VatD | VatD_fwd |  | TATTATGAATGGAGCAAATCATA |  | 23 | 141 | 57.70 | False | 38.58 | -160.84 |
|  | VatD_rev |  | ATCCATACATCATTACCTATTATTG |  | 25 | 141 | 58.84 | False | 0.00 | 0.00 |
|  | VatD_lnc3 | GCAGCGTGGGCTCGCGTGCCGGGGTTTTTT | CATTTAATTTATTTGGTA |  | 18 | 141 | 46.48 | False | 0.00 | 0.00 |
|  | VatD_detection |  | ATGGATGGGAGAAA | TTT | 14 | 141 | 49.72 | False | 0.00 | 0.00 |
| OXA-229 | OXA-229_fwd |  | AAGTTTAAAATGAAAGGTTTATTT |  | 24 | 98 | 56.37 | False | 0.00 | 0.00 |
|  | OXA-229_rev |  | TCTGATATGACTGGGCGT |  | 18 | 98 | 61.00 | False | 0.00 | 0.00 |
|  | OXA-229_lnc3 | GCAGCGTGGGCTCGCGTGCCGGGGTTTTTT | TTGGCATTTTCAGG |  | 14 | 98 | 50.58 | False | 0.00 | 0.00 |
|  | OXA-229_detection |  | TTGTGTTTATGATTCA | TTT | 16 | 98 | 48.25 | False | 0.00 | 0.00 |
| B-2 | B-2_fwd |  | AAAAACTTATTCTACTAAAATGAC |  | 24 | 134 | 55.80 | False | 0.00 | 0.00 |
|  | B-2_rev |  | CTTTTCCGGGATAGTAAACC |  | 20 | 134 | 59.43 | False | 0.00 | 0.00 |
|  | B-2_detection |  | TTTCAAAGTAGGAAA | TTT | 15 | 134 | 45.99 | False | 34.61 | 199.45 |
|  | B-2_lnc3 | GCAGCGTGGGCTCGCGTGCCGGGGTTTTTT | TTTGACAATAATAAATC |  | 17 | 134 | 45.13 | False | 0.00 | 0.00 |
| ErmG | ErmG_fwd |  | TAGTCAAAATTTTATTACTTCAAAA |  | 25 | 127 | 56.34 | False | 0.00 | 0.00 |
|  | ErmG_rev |  | CTTTACCAATTCAGCAGTA |  | 19 | 127 | 55.94 | False | 0.00 | 0.00 |
|  | ErmG_lnc3 | GCAGCGTGGGCTCGCGTGCCGGGGTTTTTT | TGAATTGCATAAGTTT |  | 16 | 127 | 49.10 | False | 0.00 | 0.00 |
|  | ErmG_detection |  | AGATGAAAAAGATAACA | TTT | 17 | 127 | 48.82 | False | 0.00 | 0.00 |
| dfrA5 | dfrA5_fwd |  | GCCGCAAGACGTTCGAA |  | 17 | 111 | 64.10 | False | 44.07 | -374.46 |
|  | dfrA5_rev |  | CGATCGACGGGAATACT |  | 17 | 111 | 58.70 | False | 40.40 | -243.64 |
|  | dfrA5_lnc3 | GCAGCGTGGGCTCGCGTGCCGGGGTTTTTT | GGAAATACGCGGTC |  | 14 | 111 | 54.41 | False | 0.00 | 0.00 |
|  | dfrA5_detection |  | GTTACTCGCTCAGC | TTT | 14 | 111 | 53.92 | False | 0.00 | 0.00 |
| ACT-12 | ACT-12_fwd |  | GTGACGGTAAAGCGGTTCA |  | 19 | 114 | 64.06 | False | 0.00 | 0.00 |
|  | ACT-12_rev |  | CATCGGGGGCCATGTTAG |  | 18 | 114 | 63.56 | False | 0.00 | 0.00 |
|  | ACT-12_detection |  | AACGTGAAGGATATG | TTT | 15 | 114 | 50.18 | False | 0.00 | 0.00 |
|  | ACT-12_lnc3 | GCAGCGTGGGCTCGCGTGCCGGGGTTTTTT | ATGGCGTGAAAACC |  | 14 | 114 | 54.33 | False | 0.00 | 0.00 |
| VanXYc3 | VanXYc3_fwd |  | TTATTTTACGCTATCCCGC |  | 19 | 128 | 58.88 | False | 0.00 | 0.00 |
|  | VanXYc3_rev |  | GTATTCTTCCAAKGTCCACTGT |  | 22 | 128 | 63.12 | False | 0.00 | 0.00 |
|  | VanXYc3_lnc3 | GCAGCGTGGGCTCGCGTGCCGGGGTTTTTT | ATTACAGGGATTGGC |  | 15 | 128 | 53.31 | False | 0.00 | 0.00 |
|  | VanXYc3_detection |  | TATGAACCATGGCA | TTT | 14 | 128 | 50.88 | False | 0.00 | 0.00 |
| smeE | smeE_fwd |  | CCGGCGGTGGAAATCAG |  | 17 | 160 | 63.94 | False | 0.00 | 0.00 |
|  | smeE_rev |  | AGGTCAGGGTGATGGTG |  | 17 | 160 | 61.25 | False | 0.00 | 0.00 |
|  | smeE_lnc3 | GCAGCGTGGGCTCGCGTGCCGGGGTTTTTT | AGATCATCGAGCAG |  | 14 | 160 | 51.54 | False | 0.00 | 0.00 |
|  | smeE_detection |  | AACATGAAGGGCCT | TTT | 14 | 160 | 54.34 | False | 0.00 | 0.00 |
| Cmr | Cmr_fwd |  | CATTGATCGTTTGCCTT |  | 17 | 133 | 55.90 | False | 0.00 | 0.00 |
|  | Cmr_rev |  | CTCAGTGCTACGGCGAG |  | 17 | 133 | 62.90 | False | 44.46 | -474.46 |
|  | Cmr_detection |  | TTCTCTCTCCTGCT | TTT | 14 | 133 | 52.13 | False | 0.00 | 0.00 |
|  | Cmr_lnc3 | GCAGCGTGGGCTCGCGTGCCGGGGTTTTTT | CGATGACACCAGTG |  | 14 | 133 | 53.76 | False | 0.00 | 0.00 |
| OXA-114a | OXA-114a_fwd |  | CCGCCTATCCCGACTGG |  | 17 | 122 | 64.82 | False | 0.00 | 0.00 |
|  | OXA-114a_rev |  | GAAGCGGTCCTGTCCCA |  | 17 | 122 | 64.55 | False | 0.00 | 0.00 |
|  | OXA-114a_detection |  | TGGTATTCACAGCTG | TTT | 15 | 122 | 53.34 | False | 0.00 | 0.00 |
|  | OXA-114a_lnc3 | GCAGCGTGGGCTCGCGTGCCGGGGTTTTTT | AGTATTCGGTGGTC |  | 14 | 122 | 51.25 | False | 0.00 | 0.00 |
| CatA3 | CatA3_fwd |  | AAATTTGATGTAAAAAATTGGGTTC |  | 25 | 159 | 60.22 | False | 41.58 | -487.63 |
|  | CatA3_rev |  | CAGATAGATCATTACCGGATAAAA |  | 24 | 159 | 60.30 | False | 0.00 | 0.00 |
|  | CatA3_lnc3 | GCAGCGTGGGCTCGCGTGCCGGGGTTTTTT | GTGGTTTTAGCTTAACA |  | 17 | 159 | 53.48 | False | 0.00 | 0.00 |
|  | CatA3_detection |  | AGCAAAATTGATATCAC | TTT | 17 | 159 | 51.14 | False | 0.00 | 0.00 |
| dfrA25 | dfrA25_fwd |  | GGGCGCAAAACATTTGAGT |  | 19 | 150 | 63.41 | False | 0.00 | 0.00 |
|  | dfrA25_rev |  | ATGGTCAGTGATCTCGC |  | 17 | 150 | 59.27 | False | 0.00 | 0.00 |
|  | dfrA25_lnc3 | GCAGCGTGGGCTCGCGTGCCGGGGTTTTTT | CGCTCAAACTGGAC |  | 14 | 150 | 54.58 | False | 0.00 | 0.00 |
|  | dfrA25_detection |  | AGCGGCTAATGAAAA | TTT | 15 | 150 | 53.70 | False | 0.00 | 0.00 |
| QnrVC4 | QnrVC4_fwd |  | GAATTAGAGGGGTGTGAT |  | 18 | 96 | 56.39 | False | 0.00 | 0.00 |
|  | QnrVC4_rev |  | ACAATTTGCACCTTTGA |  | 17 | 96 | 55.11 | False | 0.00 | 0.00 |
|  | QnrVC4_detection |  | TGCAGTCTTTCAAT | TTT | 14 | 96 | 48.59 | False | 0.00 | 0.00 |
|  | QnrVC4_lnc3 | GCAGCGTGGGCTCGCGTGCCGGGGTTTTTT | GCATCTTTTAAAAAC |  | 15 | 96 | 45.00 | False | 0.00 | 0.00 |
| DfrA15 | DfrA15_fwd |  | TGGTGGTGGTGAAATATA |  | 18 | 129 | 55.84 | False | 0.00 | 0.00 |
|  | DfrA15_rev |  | CTAAAAACTGGCCTAAAACTAC |  | 22 | 129 | 59.14 | False | 0.00 | 0.00 |
|  | DfrA15_detection |  | AGAAGGTGATGTCTATT | TTT | 17 | 129 | 52.98 | False | 0.00 | 0.00 |
|  | DfrA15_lnc3 | GCAGCGTGGGCTCGCGTGCCGGGGTTTTTT | AATCGACATTGAGCC |  | 15 | 129 | 54.38 | False | 0.00 | 0.00 |
| OCH-1 | OCH-1_fwd |  | CGACGTTTTGATGGAGCAA |  | 19 | 93 | 62.48 | False | 0.00 | 0.00 |
|  | OCH-1_rev |  | TAGGCGTAGTTCTTCATCTGG |  | 21 | 93 | 63.07 | False | 0.00 | 0.00 |
|  | OCH-1_lnc3 | GCAGCGTGGGCTCGCGTGCCGGGGTTTTTT | CCTGAAGAACACCT |  | 14 | 93 | 51.33 | False | 0.00 | 0.00 |
|  | OCH-1_detection |  | TCATCAATGTGCCG | TTT | 14 | 93 | 53.64 | False | 0.00 | 0.00 |
| lmrB | lmrB_fwd |  | AAAGTGATGCCGATTATG |  | 18 | 119 | 55.93 | False | 0.00 | 0.00 |
|  | lmrB_rev |  | GTTGCCGCTGTAATGTT |  | 17 | 119 | 58.78 | False | 0.00 | 0.00 |
|  | lmrB_detection |  | AACCGACCTTATGA | TTT | 14 | 119 | 49.99 | False | 0.00 | 0.00 |
|  | lmrB_lnc3 | GCAGCGTGGGCTCGCGTGCCGGGGTTTTTT | CTGAATATTGCGTT |  | 14 | 119 | 47.10 | False | 0.00 | 0.00 |
| VanTc3 | VanTc3_fwd |  | TTTCCGATCATYCAGCAAT |  | 19 | 141 | 59.63 | False | 0.00 | 0.00 |
|  | VanTc3_rev |  | ATTGTTTTGMAGCAAGGG |  | 18 | 141 | 58.52 | False | 37.72 | -58.49 |
|  | VanTc3_lnc3 | GCAGCGTGGGCTCGCGTGCCGGGGTTTTTT | CTCTATTTGCTCCA |  | 14 | 141 | 48.03 | False | 0.00 | 0.00 |
|  | VanTc3_detection |  | TCCTTATACCATTGC | TTT | 15 | 141 | 49.60 | False | 0.00 | 0.00 |
| tet(41) | tet(41)_fwd |  | ATGGCGCTGGTTTCR |  | 15 | 134 | 59.14 | False | 0.00 | 0.00 |
|  | tet(41)_rev |  | GCRAAGGCCTGCGAC |  | 15 | 134 | 62.29 | False | 35.71 | 0.00 |
|  | tet(41)_detection |  | TGGAGCATGATGGT | TTT | 14 | 134 | 54.07 | False | 0.00 | 0.00 |
|  | tet(41)_lnc3 | GCAGCGTGGGCTCGCGTGCCGGGGTTTTTT | GATCGTTTCGGC |  | 12 | 134 | 48.90 | False | 0.00 | 0.00 |
| ileS_type | ileS_type_fwd |  | CCAAGCTCACCGTCGTG |  | 17 | 132 | 63.80 | False | 43.65 | -483.11 |
|  | ileS_type_rev |  | CGATCTTCAGCCCCTGC |  | 17 | 132 | 63.30 | False | 37.95 | -51.15 |
|  | ileS_type_detection |  | AGCTTAATATAAAGGA | TTT | 16 | 132 | 45.81 | False | 0.00 | 0.00 |
|  | ileS_type_lnc3 | GCAGCGTGGGCTCGCGTGCCGGGGTTTTTT | AAATTCTCAAGTCAG |  | 15 | 132 | 47.38 | False | 0.00 | 0.00 |
| tetS | tetS_fwd |  | CAATTCAGATAAACTTTGTGGAA |  | 23 | 137 | 59.67 | False | 0.00 | 0.00 |
|  | tetS_rev |  | TTATTTTTTCCTTTTCTGATATATT |  | 25 | 137 | 55.69 | False | 0.00 | 0.00 |
|  | tetS_lnc3 | GCAGCGTGGGCTCGCGTGCCGGGGTTTTTT | GTACGTCTTTATAGTG |  | 16 | 137 | 48.74 | False | 0.00 | 0.00 |
|  | tetS_detection |  | GAACGCTACATTTG | TTT | 14 | 137 | 48.74 | False | 0.00 | 0.00 |
| DfrB4 | DfrB4_fwd |  | AAGTCTGGCGCTGCTTG |  | 17 | 94 | 63.79 | False | 0.00 | 0.00 |
|  | DfrB4_rev |  | CTGGGTGAGATTCGGACTC |  | 19 | 94 | 62.98 | False | 36.74 | 24.59 |
|  | DfrB4_lnc3 | GCAGCGTGGGCTCGCGTGCCGGGGTTTTTT | GGTACTGCACAACA |  | 14 | 94 | 52.87 | False | 0.00 | 0.00 |
|  | DfrB4_detection |  | CTTACCCCTGAAGG | TTT | 14 | 94 | 51.56 | False | 0.00 | 0.00 |
| AAC(6')-Iu | AAC(6')-Iu_fwd |  | ATGAATATTWTGCCGATATCT |  | 21 | 79 | 56.34 | False | 0.00 | 0.00 |
|  | AAC(6')-Iu_rev |  | CKTCATGATCAGGCCAGAG |  | 19 | 79 | 62.40 | False | 0.00 | 0.00 |
|  | AAC(6')-Iu_lnc3 | GCAGCGTGGGCTCGCGTGCCGGGGTTTTTT | CACAATTATCAGATTG |  | 16 | 79 | 47.43 | True | 43.62 | -699.71 |
|  | AAC(6')-Iu_detection |  | GCTAGCATTAAGAA | TTT | 14 | 79 | 45.55 | False | 0.00 | 0.00 |
| OXA-137 | OXA-137_fwd |  | TCAAATGAAACTACATTAATAGAT |  | 24 | 143 | 56.36 | False | 43.72 | -619.67 |
|  | OXA-137_rev |  | AATGTTGATGCTGGATAAAAT |  | 21 | 143 | 57.87 | False | 0.00 | 0.00 |
|  | OXA-137_detection |  | GTTATATATAATTTAAATGA | TTT | 20 | 143 | 45.01 | False | 0.00 | 0.00 |
|  | OXA-137_lnc3 | GCAGCGTGGGCTCGCGTGCCGGGGTTTTTT | GCTGAAGGAACATTA |  | 15 | 143 | 50.40 | False | 0.00 | 0.00 |
| VatE | VatE_fwd |  | TATCAAGAACGTGATCAAAAGTC |  | 23 | 101 | 61.13 | False | 43.40 | -617.04 |
|  | VatE_rev |  | GATGGGTAACGTGTTTCT |  | 18 | 101 | 57.77 | False | 37.60 | -19.37 |
|  | VatE_detection |  | ACCCAGTAAATCCCA | TTT | 15 | 101 | 54.13 | False | 0.00 | 0.00 |
|  | VatE_lnc3 | GCAGCGTGGGCTCGCGTGCCGGGGTTTTTT | ACTACACCTATTATGATG |  | 18 | 101 | 51.88 | False | 0.00 | 0.00 |
| vgaALC | vgaALC_fwd |  | GGAAAAACAACGTTACTTCAC |  | 21 | 111 | 59.87 | False | 0.00 | 0.00 |
|  | vgaALC_rev |  | TGATTCTATGAGCTTCAATTG |  | 21 | 111 | 57.70 | False | 0.00 | 0.00 |
|  | vgaALC_lnc3 | GCAGCGTGGGCTCGCGTGCCGGGGTTTTTT | AAAAAATTGTGCCTGA |  | 16 | 111 | 52.89 | False | 0.00 | 0.00 |
|  | vgaALC_detection |  | AGAAGGTATTGTAAAAC | TTT | 17 | 111 | 49.76 | False | 0.00 | 0.00 |
| fusB | fusB_fwd |  | TAAGTTCATTTGTAGGATGGA |  | 21 | 145 | 58.07 | False | 0.00 | 0.00 |
|  | fusB_rev |  | GATTCTTTATTACAAATTGTGC |  | 22 | 145 | 56.20 | False | 0.00 | 0.00 |
|  | fusB_lnc3 | GCAGCGTGGGCTCGCGTGCCGGGGTTTTTT | TATTACGATGAAAAAAAACA |  | 20 | 145 | 53.62 | False | 0.00 | 0.00 |
|  | fusB_detection |  | ATTAAAAGGACTTTATGGAG | TTT | 20 | 145 | 54.94 | False | 34.45 | 183.10 |
| smeB | smeB_fwd |  | CGAGGTGCTGTCGTTCA |  | 17 | 125 | 62.68 | False | 36.09 | 71.59 |
|  | smeB_rev |  | CGATCGGCTGGATGTTGC |  | 18 | 125 | 64.84 | False | 39.57 | -148.52 |
|  | smeB_detection |  | GTCGCCAACTTCAT | TTT | 14 | 125 | 53.54 | False | 0.00 | 0.00 |
|  | smeB_lnc3 | GCAGCGTGGGCTCGCGTGCCGGGGTTTTTT | TGGACAACTTCGAT |  | 14 | 125 | 50.94 | False | 0.00 | 0.00 |
| IND2a | IND2a_fwd |  | AGGATGTCTTGTAAAAAGC |  | 19 | 153 | 56.55 | False | 0.00 | 0.00 |
|  | IND2a_rev |  | CCACCGCCTTTCCATTC |  | 17 | 153 | 61.38 | False | 0.00 | 0.00 |
|  | IND2a_detection |  | GAAACCATGCGAAAA | TTT | 15 | 153 | 53.00 | False | 0.00 | 0.00 |
|  | IND2a_lnc3 | GCAGCGTGGGCTCGCGTGCCGGGGTTTTTT | GTAAAACAATGGCCG |  | 15 | 153 | 53.65 | False | 0.00 | 0.00 |
| GIM-1 | GIM-1_fwd |  | TGAAAAATGTATTAGTGTTTTTAAT |  | 25 | 160 | 56.64 | False | 39.90 | -314.60 |
|  | GIM-1_rev |  | ACCAACCCATTCGAATCA |  | 18 | 160 | 59.77 | False | 0.00 | 0.00 |
|  | GIM-1_lnc3 | GCAGCGTGGGCTCGCGTGCCGGGGTTTTTT | CTAGAAGTTATAAAAATT |  | 18 | 160 | 45.39 | False | 0.00 | 0.00 |
|  | GIM-1_detection |  | GAAGATGGAGTATATC | TTT | 16 | 160 | 47.40 | False | 0.00 | 0.00 |
| CblA-1 | CblA-1_fwd |  | ACGGTATGAAAACTGCAGA |  | 19 | 132 | 60.48 | False | 0.00 | 0.00 |
|  | CblA-1_rev |  | ATATGCGGGCGATGATG |  | 17 | 132 | 60.29 | False | 0.00 | 0.00 |
|  | CblA-1_lnc3 | GCAGCGTGGGCTCGCGTGCCGGGGTTTTTT | GTTATCCTTCCCGAC |  | 15 | 132 | 53.62 | False | 0.00 | 0.00 |
|  | CblA-1_detection |  | GGCCGGAAATACTAC | TTT | 15 | 132 | 54.12 | False | 0.00 | 0.00 |
| smeR | smeR_fwd |  | CTGCTGGACCTGATGCT |  | 17 | 147 | 62.09 | False | 0.00 | 0.00 |
|  | smeR_rev |  | CCTTGCCGTTCCAGGT |  | 16 | 147 | 62.03 | False | 0.00 | 0.00 |
|  | smeR_lnc3 | GCAGCGTGGGCTCGCGTGCCGGGGTTTTTT | CCGACGACTACATC |  | 14 | 147 | 52.72 | False | 0.00 | 0.00 |
|  | smeR_detection |  | TGCAAGCCGTTC | TTT | 12 | 147 | 51.73 | False | 0.00 | 0.00 |
| smeS | smeS_fwd |  | TCAAATTCGGCCTGACC |  | 17 | 147 | 60.20 | False | 0.00 | 0.00 |
|  | smeS_rev |  | GCTGCATGCGCAGGT |  | 15 | 147 | 63.50 | True | 65.05 | -2803.61 |
|  | smeS_lnc3 | GCAGCGTGGGCTCGCGTGCCGGGGTTTTTT | CACCGCCTGCC |  | 11 | 147 | 54.63 | False | 0.00 | 0.00 |
|  | smeS_detection |  | TGCTGGTGCTGG | TTT | 12 | 147 | 54.01 | True | 41.98 | -347.47 |
| smeC | smeC_lnc3 | GCAGCGTGGGCTCGCGTGCCGGGGTTTTTT | CGCGTTGTCGAC |  | 12 | 76 | 53.43 | True | 41.34 | -277.35 |
|  | smeC_detection |  | GCTGTTCTCGGC | TTT | 12 | 76 | 52.11 | True | 40.19 | -245.40 |
|  | smeC_fwd |  | GCCAAYTACGGCCACAG |  | 17 | 76 | 62.82 | True | 51.11 | -1001.06 |
|  | smeC_rev |  | TGGGYGCGAACGAC |  | 14 | 76 | 59.95 | True | 53.46 | -896.82 |
| smeF | smeF_fwd |  | AGCGCCCGGACATCAT |  | 16 | 199 | 64.20 | False | 0.00 | 0.00 |
|  | smeF_rev |  | CCCTGGAAGATCGGCAG |  | 17 | 199 | 62.57 | False | 38.71 | -50.57 |
|  | smeF_lnc3 | GCAGCGTGGGCTCGCGTGCCGGGGTTTTTT | TTCTTCCCGAGCAT |  | 14 | 199 | 54.14 | False | 0.00 | 0.00 |
|  | smeF_detection |  | CTCGCTGACCGG | TTT | 12 | 199 | 54.63 | True | 39.19 | -141.64 |
| RbpA | RbpA_fwd |  | ATGGCTGATCGYGTCCTG |  | 18 | 229 | 64.32 | False | 42.96 | -330.10 |
|  | RbpA_rev |  | GCTTGACCTTCTTGGGCTC |  | 19 | 229 | 64.50 | True | 51.23 | -890.81 |
|  | RbpA_lnc3 | GCAGCGTGGGCTCGCGTGCCGGGGTTTTTT | CCGTGAGCTACGA |  | 13 | 229 | 53.83 | True | 58.83 | -1643.78 |
|  | RbpA_detection |  | GACCGACCGCAA | TTT | 12 | 229 | 54.32 | False | 0.00 | 0.00 |
| ramA | ramA_fwd |  | CAAGCTGCTGCTGGCG |  | 16 | 131 | 64.79 | True | 55.45 | -1196.07 |
|  | ramA_rev |  | CMGGCGGCTGGTTGAA |  | 16 | 131 | 64.35 | False | 0.00 | 0.00 |
|  | ramA_lnc3 | GCAGCGTGGGCTCGCGTGCCGGGGTTTTTT | ATCTGCCTGCGTTA |  | 14 | 131 | 54.75 | False | 0.00 | 0.00 |
|  | ramA_detection |  | CGGGTTTGACTCG | TTT | 13 | 131 | 52.96 | True | 38.83 | -95.84 |
| OXA-12 | OXA-12_fwd |  | CCGCTGGGAAACCTA |  | 15 | 111 | 57.24 | True | 58.51 | -1284.04 |
|  | OXA-12_rev |  | AGATCCCGRTTGCCGTA |  | 17 | 111 | 62.14 | True | 51.08 | -937.81 |
|  | OXA-12_lnc3 | GCAGCGTGGGCTCGCGTGCCGGGGTTTTTT | GTCTGGTTCTCCC |  | 13 | 111 | 50.64 | False | 0.00 | 0.00 |
|  | OXA-12_detection |  | AGCAGATCACCGA | TTT | 13 | 111 | 52.44 | False | 0.00 | 0.00 |

## Virulence factor genes (VF) – primers and probes

### VF-1

Supplementary Table S13: Primer and probe sequences and additional information of VF-1.

| Gene | Primer/Probe | Hybridization Sequence | Sequence | Spacer | Length | Product Size | Tm | Hairpin | Hairpin Tm | Hairpin delta G |
| --- | --- | --- | --- | --- | --- | --- | --- | --- | --- | --- |
| sak | VFG001328(gi:21283614)_fwd |  | GCGCAAAGATCGAAGTCAC |  | 19 | 103 | 63.23 | False | 0.00 | 0.00 |
|  | VFG001328(gi:21283614)_rev |  | TCTGATAAATCTGGGACAACAA |  | 22 | 103 | 61.36 | False | 43.14 | -192.30 |
|  | VFG001328(gi:21283614)_lnc3 | GCAGCGTGGGCTCGCGTGCCGGGGTTTTTT | GAATAAGAAAAAAGAAGAAA |  | 20 | 103 | 51.70 | False | 0.00 | 0.00 |
|  | VFG001328(gi:21283614)_detection |  | CGAAGTCTTTCCCT | TTT | 14 | 103 | 51.54 | False | 0.00 | 0.00 |
| fimI | VFG000444(gi:16763924)_fwd |  | TTTACCGGTCCTGGCAG |  | 17 | 85 | 62.00 | False | 0.00 | 0.00 |
|  | VFG000444(gi:16763924)_rev |  | CATGACGCCAGACCTCC |  | 17 | 85 | 62.88 | False | 0.00 | 0.00 |
|  | VFG000444(gi:16763924)_detection |  | CGTTACGGTTAATCTC | TTT | 16 | 85 | 52.64 | False | 0.00 | 0.00 |
|  | VFG000444(gi:16763924)_lnc3 | GCAGCGTGGGCTCGCGTGCCGGGGTTTTTT | TCAGCGTTCCATTTT |  | 15 | 85 | 54.00 | False | 0.00 | 0.00 |
| cbpD | VFG001355(gi:15902008)_fwd |  | TAGTGTCTATGCCTATTCC |  | 19 | 158 | 56.43 | False | 0.00 | 0.00 |
|  | VFG001355(gi:15902008)_rev |  | AACCATTGACATTACTCAAA |  | 20 | 158 | 56.59 | False | 38.85 | -152.19 |
|  | VFG001355(gi:15902008)_detection |  | GAGCCAGGAGATTG | TTT | 14 | 158 | 52.75 | False | 0.00 | 0.00 |
|  | VFG001355(gi:15902008)_lnc3 | GCAGCGTGGGCTCGCGTGCCGGGGTTTTTT | CTGCTTATTATAAAAATGG |  | 19 | 158 | 51.13 | False | 0.00 | 0.00 |
| csgB | VFG000457(gi:16764499)_fwd |  | TGAATTAAGCAAGTCTTCATTTA |  | 23 | 116 | 58.57 | False | 39.66 | -215.07 |
|  | VFG000457(gi:16764499)_rev |  | CTTCTTGTGAAATAACGGACAAT |  | 23 | 116 | 61.64 | False | 0.00 | 0.00 |
|  | VFG000457(gi:16764499)_lnc3 | GCAGCGTGGGCTCGCGTGCCGGGGTTTTTT | ATTGGTCAAGTCGG |  | 14 | 116 | 52.52 | False | 0.00 | 0.00 |
|  | VFG000457(gi:16764499)_detection |  | CACGGATAATAGTGC | TTT | 15 | 116 | 51.45 | False | 0.00 | 0.00 |
| ideR | VFG001406(gi:15609848)_fwd |  | ATGAACGAGTTGGTTGA |  | 17 | 72 | 56.34 | False | 0.00 | 0.00 |
|  | VFG001406(gi:15609848)_rev |  | CGTCACGCCCTCTTCCT |  | 17 | 72 | 64.58 | False | 0.00 | 0.00 |
|  | VFG001406(gi:15609848)_detection |  | CGGACCATCTACGA | TTT | 14 | 72 | 54.13 | False | 0.00 | 0.00 |
|  | VFG001406(gi:15609848)_lnc3 | GCAGCGTGGGCTCGCGTGCCGGGGTTTTTT | CCGAGATGTACCTG |  | 14 | 72 | 52.02 | False | 0.00 | 0.00 |
| lytC | VFG001377(gi:15903474)_fwd |  | TACAAGAGTTGGTTTTATCT |  | 20 | 139 | 55.30 | False | 36.46 | 31.37 |
|  | VFG001377(gi:15903474)_rev |  | CATTCAAGAAATAACTTCCTTGC |  | 23 | 139 | 60.63 | False | 38.40 | -91.38 |
|  | VFG001377(gi:15903474)_detection |  | ATTGATTGGAAATAAG | TTT | 16 | 139 | 46.13 | False | 0.00 | 0.00 |
|  | VFG001377(gi:15903474)_lnc3 | GCAGCGTGGGCTCGCGTGCCGGGGTTTTTT | CATCAAGAATGGCA |  | 14 | 139 | 49.88 | False | 0.00 | 0.00 |
| icl | VFG001381(gi:57116734)_fwd |  | TTCTACCGCACCAAGAAC |  | 18 | 157 | 60.50 | False | 0.00 | 0.00 |
|  | VFG001381(gi:57116734)_rev |  | GCATCTGGTCCGGGTACT |  | 18 | 157 | 64.49 | False | 0.00 | 0.00 |
|  | VFG001381(gi:57116734)_detection |  | GATGGAGACCGGTA | TTT | 14 | 157 | 53.47 | False | 0.00 | 0.00 |
|  | VFG001381(gi:57116734)_lnc3 | GCAGCGTGGGCTCGCGTGCCGGGGTTTTTT | GCCGACTTGATCTG |  | 14 | 157 | 53.85 | False | 0.00 | 0.00 |
| hlgC | VFG001274(gi:21284072)_fwd |  | TCCAATCAGCCCCATCAC |  | 18 | 119 | 62.73 | False | 0.00 | 0.00 |
|  | VFG001274(gi:21284072)_rev |  | CCATAAAACACTTTTTGAGTT |  | 21 | 119 | 56.79 | False | 41.20 | -227.09 |
|  | VFG001274(gi:21284072)_detection |  | ACACAACAAAATTATG | TTT | 16 | 119 | 47.76 | False | 0.00 | 0.00 |
|  | VFG001274(gi:21284072)_lnc3 | GCAGCGTGGGCTCGCGTGCCGGGGTTTTTT | AAATCGATTAGCTAT |  | 15 | 119 | 45.40 | False | 0.00 | 0.00 |
| csgF | VFG000461(gi:16764496)_fwd |  | GGACGTATGGTGACCAA |  | 17 | 141 | 59.09 | False | 41.95 | -273.52 |
|  | VFG000461(gi:16764496)_rev |  | TTAAAAATCGGTTGACTGA |  | 19 | 141 | 55.77 | False | 37.39 | -27.47 |
|  | VFG000461(gi:16764496)_detection |  | GGACAGAAAAACGG | TTT | 14 | 141 | 51.40 | False | 0.00 | 0.00 |
|  | VFG000461(gi:16764496)_lnc3 | GCAGCGTGGGCTCGCGTGCCGGGGTTTTTT | CAGCTCAACGTCAC |  | 14 | 141 | 54.63 | False | 0.00 | 0.00 |
| icaC | VFG001288(gi:21284318)_fwd |  | TCATTTTTAAAATTAACTATAACCT |  | 25 | 137 | 55.64 | False | 0.00 | 0.00 |
|  | VFG001288(gi:21284318)_rev |  | TAATGGATAATAGTGTAGCACGGT |  | 24 | 137 | 63.65 | False | 43.50 | -469.95 |
|  | VFG001288(gi:21284318)_detection |  | ATATTACTTTACGAACAA | TTT | 18 | 137 | 49.95 | False | 0.00 | 0.00 |
|  | VFG001288(gi:21284318)_lnc3 | GCAGCGTGGGCTCGCGTGCCGGGGTTTTTT | ACAGCAATCATTTTT |  | 15 | 137 | 48.42 | False | 0.00 | 0.00 |
| csgA | VFG000458(gi:16764500)_fwd |  | GTACTATTGAACTGACTCAGAAT |  | 23 | 125 | 59.80 | False | 38.71 | -133.11 |
|  | VFG000458(gi:16764500)_rev |  | GGTCTGATTAACCAGCGC |  | 18 | 125 | 61.76 | False | 40.21 | -256.23 |
|  | VFG000458(gi:16764500)_detection |  | TCCGATATTACTGTC | TTT | 15 | 125 | 48.58 | False | 0.00 | 0.00 |
|  | VFG000458(gi:16764500)_lnc3 | GCAGCGTGGGCTCGCGTGCCGGGGTTTTTT | GGAACGCTAAAAAC |  | 14 | 125 | 48.99 | False | 0.00 | 0.00 |
| nanB | VFG001360(gi:15901522)_fwd |  | GAAATAAGAGACGCCCAA |  | 18 | 133 | 57.85 | False | 0.00 | 0.00 |
|  | VFG001360(gi:15901522)_rev |  | TTGTGTATGTTTTATCTTTAGAA |  | 23 | 133 | 56.04 | False | 0.00 | 0.00 |
|  | VFG001360(gi:15901522)_lnc3 | GCAGCGTGGGCTCGCGTGCCGGGGTTTTTT | CATAAAGGACAGGC |  | 14 | 133 | 50.10 | False | 0.00 | 0.00 |
|  | VFG001360(gi:15901522)_detection |  | AGTTGAAAATACACT | TTT | 15 | 133 | 46.15 | False | 0.00 | 0.00 |
| sspC | VFG001294(gi:21282659)_fwd |  | CAATTTATAAATTTAGTTTACGACA |  | 25 | 158 | 56.88 | False | 41.69 | -375.59 |
|  | VFG001294(gi:21282659)_rev |  | AAAAGAATATGATATTGATTGTGAC |  | 25 | 158 | 58.44 | False | 0.00 | 0.00 |
|  | VFG001294(gi:21282659)_lnc3 | GCAGCGTGGGCTCGCGTGCCGGGGTTTTTT | AATTGGAGTAATCATC |  | 16 | 158 | 48.17 | False | 0.00 | 0.00 |
|  | VFG001294(gi:21282659)_detection |  | AATTACAAAAATCAATTT | TTT | 18 | 158 | 47.21 | False | 0.00 | 0.00 |
| lytA | VFG001358(gi:15901761)_fwd |  | TTAAGAACAGATTTGCCTCA |  | 20 | 112 | 58.52 | False | 0.00 | 0.00 |
|  | VFG001358(gi:15901761)_rev |  | TCCGCCAGTGATARTCC |  | 17 | 112 | 59.99 | False | 0.00 | 0.00 |
|  | VFG001358(gi:15901761)_detection |  | GCACACTCAACTGG | TTT | 14 | 112 | 54.42 | False | 0.00 | 0.00 |
|  | VFG001358(gi:15901761)_lnc3 | GCAGCGTGGGCTCGCGTGCCGGGGTTTTTT | CATATAGGCAAGTACAC |  | 17 | 112 | 52.85 | False | 0.00 | 0.00 |
| icaD | VFG001286(gi:21284316)_fwd |  | CGAAAGTATCAATACAATACG |  | 21 | 136 | 56.22 | False | 39.35 | -236.88 |
|  | VFG001286(gi:21284316)_rev |  | CTGCCATTTTTGAATCAATATGC |  | 23 | 136 | 61.57 | False | 0.00 | 0.00 |
|  | VFG001286(gi:21284316)_lnc3 | GCAGCGTGGGCTCGCGTGCCGGGGTTTTTT | TGAAAATACTGAAATTT |  | 17 | 136 | 47.28 | False | 0.00 | 0.00 |
|  | VFG001286(gi:21284316)_detection |  | TAGATATATTTGAAACTATG | TTT | 20 | 136 | 49.14 | False | 0.00 | 0.00 |
| pce | VFG001354(gi:15900810)_fwd |  | TCAAGCTGGTTGGCATAAGAG |  | 21 | 123 | 64.47 | False | 40.45 | -255.10 |
|  | VFG001354(gi:15900810)_rev |  | CCCGTTTGGTTAAAGTAATAC |  | 21 | 123 | 58.56 | False | 0.00 | 0.00 |
|  | VFG001354(gi:15900810)_lnc3 | GCAGCGTGGGCTCGCGTGCCGGGGTTTTTT | AACTGGTGGTATCAA |  | 15 | 123 | 51.62 | False | 0.00 | 0.00 |
|  | VFG001354(gi:15900810)_detection |  | GCGCCTGATTCTAC | TTT | 14 | 123 | 53.96 | False | 0.00 | 0.00 |
| fbpA | VFG001419(gi:15610940)_fwd |  | GTCTTTCGATGGCTGCT |  | 17 | 99 | 60.29 | False | 0.00 | 0.00 |
|  | VFG001419(gi:15610940)_rev |  | GGGAGGGGTCCAACAGG |  | 17 | 99 | 64.64 | False | 0.00 | 0.00 |
|  | VFG001419(gi:15610940)_detection |  | AGCAGTTCGTCTAC | TTT | 14 | 99 | 51.82 | False | 0.00 | 0.00 |
|  | VFG001419(gi:15610940)_lnc3 | GCAGCGTGGGCTCGCGTGCCGGGGTTTTTT | GATCTATCACCCCC |  | 14 | 99 | 50.90 | False | 0.00 | 0.00 |
| hysA | VFG001315(gi:21283858)_fwd |  | GATGACCAACATGCTAGT |  | 18 | 96 | 57.51 | False | 43.47 | -461.49 |
|  | VFG001315(gi:21283858)_rev |  | GACAATTTTATCGTTTAATATGAA |  | 24 | 96 | 56.27 | False | 0.00 | 0.00 |
|  | VFG001315(gi:21283858)_detection |  | CAGGACAAAACTTTAAC | TTT | 17 | 96 | 52.14 | False | 0.00 | 0.00 |
|  | VFG001315(gi:21283858)_lnc3 | GCAGCGTGGGCTCGCGTGCCGGGGTTTTTT | AATGGATTTTGAAAAT |  | 16 | 96 | 46.51 | False | 0.00 | 0.00 |
| sspB | VFG001295(gi:21282660)_fwd |  | TAAAATTGTTTATACTTTAACACTT |  | 25 | 144 | 56.10 | False | 39.06 | -175.97 |
|  | VFG001295(gi:21282660)_rev |  | GTGATATTTGAATTTTTATCTTTAA |  | 25 | 144 | 55.07 | False | 0.00 | 0.00 |
|  | VFG001295(gi:21282660)_lnc3 | GCAGCGTGGGCTCGCGTGCCGGGGTTTTTT | TTAAACAAATCAAAAGA |  | 17 | 144 | 47.60 | False | 0.00 | 0.00 |
|  | VFG001295(gi:21282660)_detection |  | AGACATGAATTACAGTG | TTT | 17 | 144 | 52.50 | False | 0.00 | 0.00 |
| fbpC | VFG001811(gi:57116693)_fwd |  | CTTCCGGGACACCTACG |  | 17 | 121 | 62.12 | False | 35.84 | 62.01 |
|  | VFG001811(gi:57116693)_rev |  | CTGGATATCGGCCTTCATGG |  | 20 | 121 | 64.01 | False | 39.35 | -144.38 |
|  | VFG001811(gi:57116693)_detection |  | CCAACGGAACACAC | TTT | 14 | 121 | 54.86 | False | 0.00 | 0.00 |
|  | VFG001811(gi:57116693)_lnc3 | GCAGCGTGGGCTCGCGTGCCGGGGTTTTTT | GTTTAACTTCCCGC |  | 14 | 121 | 51.52 | False | 0.00 | 0.00 |
| hlgB | VFG001275(gi:21284073)_fwd |  | AAACTTCATAGCGCAACAC |  | 19 | 128 | 60.24 | False | 0.00 | 0.00 |
|  | VFG001275(gi:21284073)_rev |  | CACGTTGATAAGTTACTGTA |  | 20 | 128 | 56.25 | False | 0.00 | 0.00 |
|  | VFG001275(gi:21284073)_detection |  | CAAGATGGCGCTAAA | TTT | 15 | 128 | 54.79 | False | 0.00 | 0.00 |
|  | VFG001275(gi:21284073)_lnc3 | GCAGCGTGGGCTCGCGTGCCGGGGTTTTTT | CGTACTATCACACAGA |  | 16 | 128 | 53.08 | False | 0.00 | 0.00 |
| fimD | VFG000446(gi:16763926)_fwd |  | TTACCGATCTTAATCCTACCTCTTC |  | 25 | 106 | 63.79 | False | 0.00 | 0.00 |
|  | VFG000446(gi:16763926)_rev |  | AGCGGAACGGTAGAGTAAG |  | 19 | 106 | 62.29 | True | 74.41 | -2174.08 |
|  | VFG000446(gi:16763926)_detection |  | GAAAAAGACGGTAGC | TTT | 15 | 106 | 52.44 | False | 0.00 | 0.00 |
|  | VFG000446(gi:16763926)_lnc3 | GCAGCGTGGGCTCGCGTGCCGGGGTTTTTT | AGGTGACGGTAGAT |  | 14 | 106 | 52.29 | False | 0.00 | 0.00 |
| csgE | VFG000460(gi:16764497)_fwd |  | CCGGAAACCTGCATGCC |  | 17 | 110 | 64.62 | False | 0.00 | 0.00 |
|  | VFG000460(gi:16764497)_rev |  | CCATTTGTCGCTGAATGC |  | 18 | 110 | 60.73 | False | 37.17 | -16.76 |
|  | VFG000460(gi:16764497)_lnc3 | GCAGCGTGGGCTCGCGTGCCGGGGTTTTTT | GAGGTTCCCGGATT |  | 14 | 110 | 54.69 | False | 0.00 | 0.00 |
|  | VFG000460(gi:16764497)_detection |  | GTTAACCGACCATA | TTT | 14 | 110 | 48.15 | False | 0.00 | 0.00 |
| fimH | VFG000447(gi:16763927)_fwd |  | CACCCAATAATTTGTCGA |  | 18 | 75 | 55.75 | False | 0.00 | 0.00 |
|  | VFG000447(gi:16763927)_rev |  | AGGCGCGAATACCTACG |  | 17 | 75 | 62.27 | False | 38.24 | -69.57 |
|  | VFG000447(gi:16763927)_detection |  | TGATGATAACGCCG | TTT | 14 | 75 | 53.00 | False | 0.00 | 0.00 |
|  | VFG000447(gi:16763927)_lnc3 | GCAGCGTGGGCTCGCGTGCCGGGGTTTTTT | ATTCCGTTTCATCT |  | 14 | 75 | 47.81 | False | 0.00 | 0.00 |
| fimC | VFG000445(gi:16763925)_fwd |  | ATTTATCCCTCGGCGGC |  | 17 | 116 | 62.66 | False | 0.00 | 0.00 |
|  | VFG000445(gi:16763925)_rev |  | AACGTTTTTTCTTTCTGC |  | 18 | 116 | 55.28 | False | 0.00 | 0.00 |
|  | VFG000445(gi:16763925)_lnc3 | GCAGCGTGGGCTCGCGTGCCGGGGTTTTTT | CAGTAATAGCGATAC |  | 15 | 116 | 47.47 | False | 0.00 | 0.00 |
|  | VFG000445(gi:16763925)_detection |  | TCAAGAACGTTACCT | TTT | 15 | 116 | 51.80 | False | 0.00 | 0.00 |
| geh | VFG001316(gi:21282026)_fwd |  | GTAACAAAGAAGAAATTGCC |  | 20 | 139 | 56.85 | False | 0.00 | 0.00 |
|  | VFG001316(gi:21282026)_rev |  | AACTTATCAGCTGCTTGTG |  | 19 | 139 | 59.26 | False | 0.00 | 0.00 |
|  | VFG001316(gi:21282026)_detection |  | TCAATCACAACATT | TTT | 14 | 139 | 45.39 | False | 0.00 | 0.00 |
|  | VFG001316(gi:21282026)_lnc3 | GCAGCGTGGGCTCGCGTGCCGGGGTTTTTT | ATAACAATATGGTTGCA |  | 17 | 139 | 52.35 | False | 34.80 | 177.55 |
| phoP | VFG001386(gi:15607897)_fwd |  | CAAAGCCCTTCAGTTTGGAG |  | 20 | 148 | 62.98 | False | 0.00 | 0.00 |
|  | VFG001386(gi:15607897)_rev |  | CCCGCCTTCCACACTTC |  | 17 | 148 | 63.47 | False | 0.00 | 0.00 |
|  | VFG001386(gi:15607897)_lnc3 | GCAGCGTGGGCTCGCGTGCCGGGGTTTTTT | CAACAAGGAACCAC |  | 14 | 148 | 51.20 | False | 0.00 | 0.00 |
|  | VFG001386(gi:15607897)_detection |  | GTAATGTTCGACTGA | TTT | 15 | 148 | 50.20 | False | 0.00 | 0.00 |
| ebp | VFG001278(gi:21283098)_fwd |  | TGTCTAATAATTTTAAAGATGACTT |  | 25 | 106 | 57.22 | False | 40.09 | -209.80 |
|  | VFG001278(gi:21283098)_rev |  | TCTAATTCTGATTGGTCTTTTTC |  | 23 | 106 | 58.90 | False | 0.00 | 0.00 |
|  | VFG001278(gi:21283098)_detection |  | ACATCAAGACCATA | TTT | 14 | 106 | 46.23 | False | 0.00 | 0.00 |
|  | VFG001278(gi:21283098)_lnc3 | GCAGCGTGGGCTCGCGTGCCGGGGTTTTTT | GATAGACACAAATTC |  | 15 | 106 | 45.43 | False | 0.00 | 0.00 |
| csgC | VFG000459(gi:16764501)_fwd |  | GGACAAAGCCATACACAGC |  | 19 | 99 | 62.58 | False | 0.00 | 0.00 |
|  | VFG000459(gi:16764501)_rev |  | CGAGTCCTCTGAAGATATATTTAC |  | 24 | 99 | 60.35 | False | 0.00 | 0.00 |
|  | VFG000459(gi:16764501)_lnc3 | GCAGCGTGGGCTCGCGTGCCGGGGTTTTTT | TATCTTTACCTGCTAATCA |  | 19 | 99 | 54.73 | False | 0.00 | 0.00 |
|  | VFG000459(gi:16764501)_detection |  | ACCGATTGAGTTGTC | TTT | 15 | 99 | 53.80 | False | 0.00 | 0.00 |
| hbhA | VFG001383(gi:15607616)_fwd |  | TCAAGGCTCCGTTGCTTG |  | 18 | 109 | 63.92 | False | 0.00 | 0.00 |
|  | VFG001383(gi:15607616)_rev |  | GTGTCCGTACGAGTCTC |  | 17 | 109 | 59.30 | False | 0.00 | 0.00 |
|  | VFG001383(gi:15607616)_detection |  | GATCACGAACCTGC | TTT | 14 | 109 | 54.27 | False | 0.00 | 0.00 |
|  | VFG001383(gi:15607616)_lnc3 | GCAGCGTGGGCTCGCGTGCCGGGGTTTTTT | CACTGTCAACGAGTT |  | 15 | 109 | 54.15 | False | 0.00 | 0.00 |
| hlb | VFG001798(gi:57650692)_fwd |  | CACATACACAATCTGAAGA |  | 19 | 111 | 55.64 | False | 0.00 | 0.00 |
|  | VFG001798(gi:57650692)_rev |  | CTTTWGGGATATTTTTCTTTTTAA |  | 24 | 111 | 56.26 | False | 0.00 | 0.00 |
|  | VFG001798(gi:57650692)_detection |  | ATGAAAGAAATCAGTG | TTT | 16 | 111 | 49.32 | False | 34.87 | 139.21 |
|  | VFG001798(gi:57650692)_lnc3 | GCAGCGTGGGCTCGCGTGCCGGGGTTTTTT | TTAGAGCTGAACAA |  | 14 | 111 | 47.19 | False | 0.00 | 0.00 |
| cps4A | VFG001365(gi:15900275)_fwd |  | AAGTGAAGCGAAGTGTTAATAT |  | 22 | 115 | 60.00 | False | 0.00 | 0.00 |
|  | VFG001365(gi:15900275)_rev |  | ACTAGRTTAAGATATCTAAAAGCA |  | 24 | 115 | 58.26 | False | 42.13 | -536.60 |
|  | VFG001365(gi:15900275)_detection |  | TCTTAATCTTTAAGTACAA | TTT | 19 | 115 | 50.18 | False | 0.00 | 0.00 |
|  | VFG001365(gi:15900275)_lnc3 | GCAGCGTGGGCTCGCGTGCCGGGGTTTTTT | TAGTTTGTTTTTTATTGT |  | 18 | 115 | 48.92 | False | 0.00 | 0.00 |
| hld | VFG001292(gi:21283688)_fwd |  | AGTTGTTTAATTTTAAGAATTTTTA |  | 25 | 104 | 55.03 | False | 0.00 | 0.00 |
|  | VFG001292(gi:21283688)_rev |  | TCGATAATCCATTTTACTAAGT |  | 22 | 104 | 56.61 | False | 0.00 | 0.00 |
|  | VFG001292(gi:21283688)_lnc3 | GCAGCGTGGGCTCGCGTGCCGGGGTTTTTT | AGTGATTTCAATGG |  | 14 | 104 | 46.14 | False | 0.00 | 0.00 |
|  | VFG001292(gi:21283688)_detection |  | CACAAGATATCATTTC | TTT | 16 | 104 | 47.05 | False | 0.00 | 0.00 |
| fimF | VFG000448(gi:16763928)_fwd |  | GCCGTCGATATTGACAAC |  | 18 | 101 | 59.49 | False | 42.65 | -411.77 |

### VF-2

Supplementary Table S14: Primer and probe sequences and additional information of VF-2.

| Gene | Primer/Probe | Hybridization Sequence | Sequence | Spacer | Length | Product Size | Tm | Hairpin | Hairpin Tm | Hairpin delta G |
| --- | --- | --- | --- | --- | --- | --- | --- | --- | --- | --- |
| misL | VFG002304(gi:16767041)_fwd |  | CACCATCAATGAAGATTATG |  | 20 | 106 | 55.51 | False | 0.00 | 0.00 |
|  | VFG002304(gi:16767041)_rev |  | AGTATTGCCGAGAACCTG |  | 18 | 106 | 59.94 | False | 0.00 | 0.00 |
|  | VFG002304(gi:16767041)_detection |  | TTAATACTGTGCTC | TTT | 14 | 106 | 45.03 | False | 0.00 | 0.00 |
|  | VFG002304(gi:16767041)_lnc3 | GCAGCGTGGGCTCGCGTGCCGGGGTTTTTT | CGGTAAGCTGGTCT |  | 14 | 106 | 54.79 | False | 0.00 | 0.00 |
| lpfA | VFG000456(gi:16766926)_fwd |  | TTCCACTTCTGCTTTCGCTG |  | 20 | 127 | 64.82 | False | 39.15 | -136.49 |
|  | VFG000456(gi:16766926)_rev |  | CTTAACCTGACCCAGCACA |  | 19 | 127 | 63.10 | False | 0.00 | 0.00 |
|  | VFG000456(gi:16766926)_lnc3 | GCAGCGTGGGCTCGCGTGCCGGGGTTTTTT | ACCATTAAATTCACC |  | 15 | 127 | 47.74 | False | 0.00 | 0.00 |
|  | VFG000456(gi:16766926)_detection |  | GGTGAAATCGTTGA | TTT | 14 | 127 | 49.95 | False | 0.00 | 0.00 |
| stx2B | VFG000838(gi:15800961)_fwd |  | AGTCGCTGGAATCTGCA |  | 17 | 104 | 61.65 | False | 40.75 | -335.37 |
|  | VFG000838(gi:15800961)_rev |  | ACTTCAGCAAATCCGGAGC |  | 19 | 104 | 64.19 | False | 0.00 | 0.00 |
|  | VFG000838(gi:15800961)_lnc3 | GCAGCGTGGGCTCGCGTGCCGGGGTTTTTT | ACTGTCACAATCAA |  | 14 | 104 | 48.06 | False | 0.00 | 0.00 |
|  | VFG000838(gi:15800961)_detection |  | ATCCAGTACCTGTGA | TTT | 15 | 104 | 53.40 | False | 0.00 | 0.00 |
| yagW/ecpD | VFG002416(gi:15799995)_fwd |  | AACTTTCGCTTTCAGAACAC |  | 20 | 130 | 60.73 | False | 0.00 | 0.00 |
|  | VFG002416(gi:15799995)_rev |  | ACGGTGTCTGAGTATATTC |  | 19 | 130 | 57.10 | False | 0.00 | 0.00 |
|  | VFG002416(gi:15799995)_detection |  | GATTTCAGCATCAGTAT | TTT | 17 | 130 | 52.67 | False | 0.00 | 0.00 |
|  | VFG002416(gi:15799995)_lnc3 | GCAGCGTGGGCTCGCGTGCCGGGGTTTTTT | TTATCAAACCCCGT |  | 14 | 130 | 50.80 | False | 0.00 | 0.00 |
| srtG1 | VFG042974(gi:225860992)_fwd |  | AAGTTCGAAGACTTTAATACCATTA |  | 25 | 152 | 60.82 | False | 39.13 | -60.72 |
|  | VFG042974(gi:225860992)_rev |  | ATCTCGAGCCCTTTTTCC |  | 18 | 152 | 60.27 | False | 0.00 | 0.00 |
|  | VFG042974(gi:225860992)_lnc3 | GCAGCGTGGGCTCGCGTGCCGGGGTTTTTT | TTTGGTGATGTGGC |  | 14 | 152 | 53.74 | False | 0.00 | 0.00 |
|  | VFG042974(gi:225860992)_detection |  | TAAGTTTGCTGATCAG | TTT | 16 | 152 | 51.74 | False | 0.00 | 0.00 |
| vexA | VFG000427(gi:16763117)_fwd |  | GGAGCCATGGTGACGGT |  | 17 | 139 | 64.98 | False | 0.00 | 0.00 |
|  | VFG000427(gi:16763117)_rev |  | TCACGTGAACTTCCATCA |  | 18 | 139 | 59.37 | False | 0.00 | 0.00 |
|  | VFG000427(gi:16763117)_detection |  | AGTATGAATCGTCT | TTT | 14 | 139 | 46.14 | False | 0.00 | 0.00 |
|  | VFG000427(gi:16763117)_lnc3 | GCAGCGTGGGCTCGCGTGCCGGGGTTTTTT | ACCCTCTCGAACAG |  | 14 | 139 | 54.25 | False | 0.00 | 0.00 |
| clfA | VFG001289(gi:21282493)_fwd |  | AGTGAATCAAACAAGTAATGAAACG |  | 25 | 99 | 63.01 | False | 0.00 | 0.00 |
|  | VFG001289(gi:21282493)_rev |  | GTTGAAACATTTTCCGCATT |  | 20 | 99 | 59.58 | False | 0.00 | 0.00 |
|  | VFG001289(gi:21282493)_lnc3 | GCAGCGTGGGCTCGCGTGCCGGGGTTTTTT | CTAATACAGTATCATCT |  | 17 | 99 | 47.40 | False | 0.00 | 0.00 |
|  | VFG001289(gi:21282493)_detection |  | GTAAATTCACCTCAAAATT | TTT | 19 | 99 | 53.37 | False | 0.00 | 0.00 |
| yagY/ecpB | VFG002412(gi:15799997)_fwd |  | TGAACAGTGACAGCAGCAC |  | 19 | 154 | 64.31 | False | 38.26 | -100.41 |
|  | VFG002412(gi:15799997)_rev |  | GCGGGAGTGAGTAGCAAC |  | 18 | 154 | 63.72 | False | 0.00 | 0.00 |
|  | VFG002412(gi:15799997)_lnc3 | GCAGCGTGGGCTCGCGTGCCGGGGTTTTTT | CAATATCCGTCTCG |  | 14 | 154 | 49.77 | False | 0.00 | 0.00 |
|  | VFG002412(gi:15799997)_detection |  | AACGGCTCTCTTCA | TTT | 14 | 154 | 54.10 | False | 0.00 | 0.00 |
| selk | VFG001327(gi:21283667)_fwd |  | TAATTTCAGAATCAAAAGATTTTAA |  | 25 | 141 | 56.39 | False | 0.00 | 0.00 |
|  | VFG001327(gi:21283667)_rev |  | TATCTAGATATTCGTTAGTAGCT |  | 23 | 141 | 57.52 | False | 0.00 | 0.00 |
|  | VFG001327(gi:21283667)_detection |  | TAGTTATAATGGCCAGT | TTT | 17 | 141 | 53.05 | False | 0.00 | 0.00 |
|  | VFG001327(gi:21283667)_lnc3 | GCAGCGTGGGCTCGCGTGCCGGGGTTTTTT | TGATGTTTTTGGTAT |  | 15 | 141 | 46.59 | False | 0.00 | 0.00 |
| lytB | VFG001356(gi:15900842)_fwd |  | TGGCTAGATAAGGATAGAAAA |  | 21 | 135 | 57.26 | False | 0.00 | 0.00 |
|  | VFG001356(gi:15900842)_rev |  | ATCACTCTCATAATAAGGGATA |  | 22 | 135 | 57.22 | False | 38.32 | -104.35 |
|  | VFG001356(gi:15900842)_lnc3 | GCAGCGTGGGCTCGCGTGCCGGGGTTTTTT | GGCTATATGAAAACAGA |  | 17 | 135 | 52.20 | False | 0.00 | 0.00 |
|  | VFG001356(gi:15900842)_detection |  | AGATTTACAAGCGC | TTT | 14 | 135 | 50.64 | False | 0.00 | 0.00 |
| tviD | VFG000429(gi:16763121)_fwd |  | AGGTTATTATGAACAGGCTT |  | 20 | 111 | 57.98 | False | 0.00 | 0.00 |
|  | VFG000429(gi:16763121)_rev |  | CAACGCGCTAATCCATG |  | 17 | 111 | 59.47 | False | 0.00 | 0.00 |
|  | VFG000429(gi:16763121)_detection |  | GAAAATTATCGGTAC | TTT | 15 | 111 | 45.61 | False | 0.00 | 0.00 |
|  | VFG000429(gi:16763121)_lnc3 | GCAGCGTGGGCTCGCGTGCCGGGGTTTTTT | ATATTAGTGATGTTGA |  | 16 | 111 | 46.33 | False | 0.00 | 0.00 |
| hlyA | VFG000840(gi:75994494)_fwd |  | TATCGACAACAGCTGCA |  | 17 | 105 | 59.10 | False | 0.00 | 0.00 |
|  | VFG000840(gi:75994494)_rev |  | GCTTAGCTCGCTCAAATT |  | 18 | 105 | 59.00 | False | 0.00 | 0.00 |
|  | VFG000840(gi:75994494)_lnc3 | GCAGCGTGGGCTCGCGTGCCGGGGTTTTTT | GGCTGTTATGCTGG |  | 14 | 105 | 54.15 | False | 0.00 | 0.00 |
|  | VFG000840(gi:75994494)_detection |  | CTATCAGTCCTCTT | TTT | 14 | 105 | 46.08 | False | 0.00 | 0.00 |
| ebpC | VFG042978(gi:29375669)_fwd |  | CAAAAATGATACCTCTAAAGT |  | 21 | 155 | 55.14 | False | 0.00 | 0.00 |
|  | VFG042978(gi:29375669)_rev |  | AATTGAATTTGACGTATTTATTAG |  | 24 | 155 | 56.26 | False | 0.00 | 0.00 |
|  | VFG042978(gi:29375669)_detection |  | TTTCTGTAAATATTCCATT | TTT | 19 | 155 | 51.23 | False | 0.00 | 0.00 |
|  | VFG042978(gi:29375669)_lnc3 | GCAGCGTGGGCTCGCGTGCCGGGGTTTTTT | GAAAAAATTAAATATCAAA |  | 19 | 155 | 46.47 | False | 0.00 | 0.00 |
| cps4D | VFG001368(gi:15900278)_fwd |  | GCCGACATTAGAAATAGCACAAA |  | 23 | 156 | 63.90 | False | 0.00 | 0.00 |
|  | VFG001368(gi:15900278)_rev |  | GAAGTAGTTGTTTTTCCTTCC |  | 21 | 156 | 58.69 | False | 0.00 | 0.00 |
|  | VFG001368(gi:15900278)_detection |  | TGAGCGGAGATAAA | TTT | 14 | 156 | 50.18 | False | 0.00 | 0.00 |
|  | VFG001368(gi:15900278)_lnc3 | GCAGCGTGGGCTCGCGTGCCGGGGTTTTTT | TGTACAAATATACAGT |  | 16 | 156 | 46.28 | False | 34.75 | 203.23 |
| paa | VFG000839(gi:15801498)_fwd |  | TGATGTCGTGGAAATAGC |  | 18 | 147 | 57.95 | False | 0.00 | 0.00 |
|  | VFG000839(gi:15801498)_rev |  | GTCCAGCCATATTTTTTGA |  | 19 | 147 | 56.93 | False | 0.00 | 0.00 |
|  | VFG000839(gi:15801498)_detection |  | TGAAACCCGTCGAT | TTT | 14 | 147 | 54.66 | False | 0.00 | 0.00 |
|  | VFG000839(gi:15801498)_lnc3 | GCAGCGTGGGCTCGCGTGCCGGGGTTTTTT | CAGGATGCAAATGA |  | 14 | 147 | 49.88 | False | 0.00 | 0.00 |
| srtC | VFG042979(gi:29375670)_fwd |  | TTGATGGTTTTATGATTCTTT |  | 21 | 116 | 55.30 | False | 0.00 | 0.00 |
|  | VFG042979(gi:29375670)_rev |  | TGCTTGATAATGAGCGATAATTT |  | 23 | 116 | 61.02 | True | 45.32 | -653.22 |
|  | VFG042979(gi:29375670)_detection |  | TAGCGATGCATTAAATAA | TTT | 18 | 116 | 53.36 | False | 0.00 | 0.00 |
|  | VFG042979(gi:29375670)_lnc3 | GCAGCGTGGGCTCGCGTGCCGGGGTTTTTT | TTGCGTATCCTTTTGT |  | 16 | 116 | 54.71 | False | 0.00 | 0.00 |
| tviE | VFG000428(gi:16763118)_fwd |  | TCAAGCCGGAATATGAGC |  | 18 | 143 | 60.43 | False | 0.00 | 0.00 |
|  | VFG000428(gi:16763118)_rev |  | GCCGTTATATACAACCTGGAAG |  | 22 | 143 | 62.77 | False | 0.00 | 0.00 |
|  | VFG000428(gi:16763118)_lnc3 | GCAGCGTGGGCTCGCGTGCCGGGGTTTTTT | GGCGTTGATTTTAT |  | 14 | 143 | 47.45 | False | 0.00 | 0.00 |
|  | VFG000428(gi:16763118)_detection |  | GAGTAACAACCATTG | TTT | 15 | 143 | 48.97 | False | 0.00 | 0.00 |
| sdrD | VFG001280(gi:21282246)_fwd |  | GTTGAATTCGAAACACCATC |  | 20 | 114 | 59.09 | False | 0.00 | 0.00 |
|  | VFG001280(gi:21282246)_rev |  | GTTATCTTTATCTTTAATGACACCT |  | 25 | 114 | 59.39 | False | 0.00 | 0.00 |
|  | VFG001280(gi:21282246)_lnc3 | GCAGCGTGGGCTCGCGTGCCGGGGTTTTTT | AACACAAGTAGGTTCA |  | 16 | 114 | 53.23 | False | 0.00 | 0.00 |
|  | VFG001280(gi:21282246)_detection |  | GGAACTGATGAAGGT | TTT | 15 | 114 | 52.76 | False | 0.00 | 0.00 |
| rrgB | VFG005303(gi:15900379)_fwd |  | AAAAGAAATTGCCGGTGT |  | 18 | 117 | 58.97 | False | 0.00 | 0.00 |
|  | VFG005303(gi:15900379)_rev |  | GCCCCTGAGAGTTTAAATGT |  | 20 | 117 | 61.48 | False | 0.00 | 0.00 |
|  | VFG005303(gi:15900379)_detection |  | AATGGCCAAACTCT | TTT | 14 | 117 | 51.60 | False | 0.00 | 0.00 |
|  | VFG005303(gi:15900379)_lnc3 | GCAGCGTGGGCTCGCGTGCCGGGGTTTTTT | ATGAAATTATTGATGAA |  | 17 | 117 | 46.68 | False | 0.00 | 0.00 |
| pitB | VFG042973(gi:225860991)_fwd |  | CTAACGCCCCTAAAGTAGA |  | 19 | 109 | 59.32 | False | 0.00 | 0.00 |
|  | VFG042973(gi:225860991)_rev |  | ACTTGACTCGGATCATCAG |  | 19 | 109 | 60.02 | False | 0.00 | 0.00 |
|  | VFG042973(gi:225860991)_detection |  | CATATACCAAAGACG | TTT | 15 | 109 | 48.07 | False | 0.00 | 0.00 |
|  | VFG042973(gi:225860991)_lnc3 | GCAGCGTGGGCTCGCGTGCCGGGGTTTTTT | TTATAATTTATTTGATAACA |  | 20 | 109 | 47.22 | False | 0.00 | 0.00 |
| vexE | VFG000423(gi:16763113)_fwd |  | CAGGCCGCTGAGCATTG |  | 17 | 125 | 64.66 | False | 0.00 | 0.00 |
|  | VFG000423(gi:16763113)_rev |  | TGCTGCAGTAACGGAAT |  | 17 | 125 | 59.43 | False | 0.00 | 0.00 |
|  | VFG000423(gi:16763113)_lnc3 | GCAGCGTGGGCTCGCGTGCCGGGGTTTTTT | CCCTGTATGCCGAT |  | 14 | 125 | 54.95 | False | 0.00 | 0.00 |
|  | VFG000423(gi:16763113)_detection |  | GCCTTAATCAGAAC | TTT | 14 | 125 | 47.09 | False | 0.00 | 0.00 |
| ebpB | VFG042977(gi:29375668)_fwd |  | CATGGATTTACGCTTGATTTTT |  | 22 | 134 | 60.07 | False | 0.00 | 0.00 |
|  | VFG042977(gi:29375668)_rev |  | ACTAATTGTCCTTCGTTG |  | 18 | 134 | 55.37 | False | 0.00 | 0.00 |
|  | VFG042977(gi:29375668)_lnc3 | GCAGCGTGGGCTCGCGTGCCGGGGTTTTTT | TATCAAATGCGTTT |  | 14 | 134 | 46.25 | False | 0.00 | 0.00 |
|  | VFG042977(gi:29375668)_detection |  | AGAAAAGACCGCTG | TTT | 14 | 134 | 53.00 | False | 0.00 | 0.00 |
| hlyB | VFG000841(gi:75994495)_fwd |  | AATGGTTACGACGCCAGATT |  | 20 | 128 | 63.73 | False | 42.13 | -369.20 |
|  | VFG000841(gi:75994495)_rev |  | AAGTCTGGCTGCCTCAAT |  | 18 | 128 | 62.25 | False | 0.00 | 0.00 |
|  | VFG000841(gi:75994495)_detection |  | TATCGATAATATTACATT | TTT | 18 | 128 | 45.90 | False | 0.00 | 0.00 |
|  | VFG000841(gi:75994495)_lnc3 | GCAGCGTGGGCTCGCGTGCCGGGGTTTTTT | CTATTAAATCGTAGTAT |  | 17 | 128 | 45.39 | False | 0.00 | 0.00 |
| stx2A | VFG000837(gi:15800960)_fwd |  | CAACGGACAGCAGTTATACC |  | 20 | 166 | 62.23 | False | 36.08 | 75.72 |
|  | VFG000837(gi:15800960)_rev |  | ACAGTGACAAAACGCAGAAC |  | 20 | 166 | 63.14 | False | 0.00 | 0.00 |
|  | VFG000837(gi:15800960)_detection |  | CATCATATCTGGCGTT | TTT | 16 | 166 | 54.76 | False | 0.00 | 0.00 |
|  | VFG000837(gi:15800960)_lnc3 | GCAGCGTGGGCTCGCGTGCCGGGGTTTTTT | GTCACTCACTGGTTT |  | 15 | 166 | 53.54 | False | 0.00 | 0.00 |
| sipA | VFG042972(gi:225860990)_fwd |  | AAGGAAAGAGTGGCGCG |  | 17 | 119 | 63.45 | False | 0.00 | 0.00 |
|  | VFG042972(gi:225860990)_rev |  | AGCAGCGTTTCTTTGTAGATATC |  | 23 | 119 | 63.15 | False | 0.00 | 0.00 |
|  | VFG042972(gi:225860990)_lnc3 | GCAGCGTGGGCTCGCGTGCCGGGGTTTTTT | GTCTCATCATCAAC |  | 14 | 119 | 46.75 | False | 0.00 | 0.00 |
|  | VFG042972(gi:225860990)_detection |  | GGTTCTCCTCAACA | TTT | 14 | 119 | 51.37 | False | 0.00 | 0.00 |
| lpfE | VFG000452(gi:16766922)_fwd |  | CCCTCCAGTACCGACTCTAC |  | 20 | 142 | 64.45 | False | 37.21 | -10.35 |
|  | VFG000452(gi:16766922)_rev |  | TGACGCTAAAATTCACC |  | 17 | 142 | 55.15 | False | 0.00 | 0.00 |
|  | VFG000452(gi:16766922)_lnc3 | GCAGCGTGGGCTCGCGTGCCGGGGTTTTTT | AACAAGCTGAACTA |  | 14 | 142 | 47.62 | False | 0.00 | 0.00 |
|  | VFG000452(gi:16766922)_detection |  | TATCGCCAGCTATGT | TTT | 15 | 142 | 54.74 | False | 0.00 | 0.00 |
| cps4B | VFG001366(gi:15900276)_fwd |  | GGGGGTGMGAACCATT |  | 16 | 106 | 60.29 | False | 0.00 | 0.00 |
|  | VFG001366(gi:15900276)_rev |  | CTTAGCTATTTCCCGAACCTG |  | 21 | 106 | 62.21 | False | 0.00 | 0.00 |
|  | VFG001366(gi:15900276)_detection |  | AAGGGCATGTTTGAA | TTT | 15 | 106 | 53.78 | False | 0.00 | 0.00 |
|  | VFG001366(gi:15900276)_lnc3 | GCAGCGTGGGCTCGCGTGCCGGGGTTTTTT | TCTCACCGTCGC |  | 12 | 106 | 53.42 | False | 0.00 | 0.00 |
| lpfD | VFG000453(gi:16766923)_fwd |  | CAAAATGCAAGTAGTAAAGA |  | 20 | 136 | 55.20 | False | 0.00 | 0.00 |
|  | VFG000453(gi:16766923)_rev |  | GTTTCTTTGTCACAAAAAGGT |  | 21 | 136 | 59.38 | False | 39.78 | -281.80 |
|  | VFG000453(gi:16766923)_lnc3 | GCAGCGTGGGCTCGCGTGCCGGGGTTTTTT | TTCTCCATCCGTTT |  | 14 | 136 | 50.85 | False | 0.00 | 0.00 |
|  | VFG000453(gi:16766923)_detection |  | GTGGGTGAAAGTAT | TTT | 14 | 136 | 48.28 | False | 0.00 | 0.00 |
| lmb | VFG001330(gi:22537388)_fwd |  | TCAAAAATTTAAAAAGGTGCGCT |  | 23 | 142 | 62.85 | False | 0.00 | 0.00 |
|  | VFG001330(gi:22537388)_rev |  | TTTCAATTGGCGAGGAGA |  | 18 | 142 | 59.78 | False | 0.00 | 0.00 |
|  | VFG001330(gi:22537388)_detection |  | ATCTGGCTAAACGAT | TTT | 15 | 142 | 52.05 | False | 0.00 | 0.00 |
|  | VFG001330(gi:22537388)_lnc3 | GCAGCGTGGGCTCGCGTGCCGGGGTTTTTT | ACACGGCATTTTCTT |  | 15 | 142 | 54.39 | False | 0.00 | 0.00 |
| sdrE | VFG001281(gi:21282247)_fwd |  | TGACGATTAATTATGATAAGAATGT |  | 25 | 134 | 58.97 | False | 0.00 | 0.00 |
|  | VFG001281(gi:21282247)_rev |  | ATATGTGATTTGCTTAGTYGCTT |  | 23 | 134 | 62.01 | False | 0.00 | 0.00 |
|  | VFG001281(gi:21282247)_detection |  | ATCGATATTACTGATC | TTT | 16 | 134 | 47.03 | False | 0.00 | 0.00 |
|  | VFG001281(gi:21282247)_lnc3 | GCAGCGTGGGCTCGCGTGCCGGGGTTTTTT | CAGATAAAAATGATCCT |  | 17 | 134 | 49.51 | False | 0.00 | 0.00 |
| lukF-PV | VFG001276(gi:21283107)_fwd |  | TTGATGCAGCTCAACATATCAC |  | 22 | 126 | 63.24 | False | 35.41 | 127.51 |
|  | VFG001276(gi:21283107)_rev |  | AATTAAAAGTTAAAATCTGAGAAA |  | 24 | 126 | 55.63 | False | 0.00 | 0.00 |
|  | VFG001276(gi:21283107)_lnc3 | GCAGCGTGGGCTCGCGTGCCGGGGTTTTTT | AATTACTTTGTACAAA |  | 16 | 126 | 45.34 | False | 0.00 | 0.00 |
|  | VFG001276(gi:21283107)_detection |  | ACAACTGCAACATC | TTT | 14 | 126 | 50.75 | False | 0.00 | 0.00 |
| tviC | VFG000430(gi:16763122)_fwd |  | GAAGAACTACGGACCAA |  | 17 | 118 | 56.12 | False | 0.00 | 0.00 |
|  | VFG000430(gi:16763122)_rev |  | CAATGACAGTCTGGTTG |  | 17 | 118 | 55.50 | False | 0.00 | 0.00 |
|  | VFG000430(gi:16763122)_detection |  | GCAGGCTTTATTGG | TTT | 14 | 118 | 51.48 | False | 0.00 | 0.00 |
|  | VFG000430(gi:16763122)_lnc3 | GCAGCGTGGGCTCGCGTGCCGGGGTTTTTT | TGATCACTGGCGTA |  | 14 | 118 | 53.80 | False | 0.00 | 0.00 |
| yagX/ecpC | VFG002415(gi:15799996)_fwd |  | AAGAGAGTGAAGATAACGCCA |  | 21 | 135 | 62.95 | False | 0.00 | 0.00 |
|  | VFG002415(gi:15799996)_rev |  | GTTGGCGCAAGCTGAGAT |  | 18 | 135 | 64.37 | True | 69.22 | -2258.48 |
|  | VFG002415(gi:15799996)_detection |  | TTACCATCCCCCTG | TTT | 14 | 135 | 53.59 | False | 0.00 | 0.00 |
|  | VFG002415(gi:15799996)_lnc3 | GCAGCGTGGGCTCGCGTGCCGGGGTTTTTT | GTTCAATGAGGCCC |  | 14 | 135 | 53.99 | False | 0.00 | 0.00 |
| sed | VFG001807(gi:758691)_fwd |  | AATATGAAACATTCTTATGCA |  | 21 | 147 | 55.18 | False | 38.91 | -135.18 |
|  | VFG001807(gi:758691)_rev |  | TGAATTGAAGTTTATTAATAAATCT |  | 25 | 147 | 55.72 | False | 39.42 | -226.54 |
|  | VFG001807(gi:758691)_detection |  | TACAGGAGATCAATTTTTA | TTT | 19 | 147 | 52.91 | False | 0.00 | 0.00 |
|  | VFG001807(gi:758691)_lnc3 | GCAGCGTGGGCTCGCGTGCCGGGGTTTTTT | ATAATAGGAGAAAATAAAAG |  | 20 | 147 | 49.91 | False | 0.00 | 0.00 |
| lukS-PV | VFG001277(gi:21283108)_fwd |  | GTTATTTAGAAGGATCTAGAATAC |  | 24 | 99 | 56.36 | False | 44.14 | -614.69 |
|  | VFG001277(gi:21283108)_rev |  | CTTTCACTTTAATTTCATGAGT |  | 22 | 99 | 56.79 | False | 0.00 | 0.00 |
|  | VFG001277(gi:21283108)_lnc3 | GCAGCGTGGGCTCGCGTGCCGGGGTTTTTT | GCATTTGTAAACAGAAATT |  | 19 | 99 | 54.44 | False | 0.00 | 0.00 |
|  | VFG001277(gi:21283108)_detection |  | ACACAGTTAAATATGA | TTT | 16 | 99 | 47.06 | False | 0.00 | 0.00 |
| vexB | VFG000426(gi:16763116)_fwd |  | TGGGGTTATCGTTTGGTTACT |  | 21 | 100 | 63.20 | False | 0.00 | 0.00 |
|  | VFG000426(gi:16763116)_rev |  | GAAATCAAAAACATCGGTCTTAGC |  | 24 | 100 | 63.23 | False | 0.00 | 0.00 |
|  | VFG000426(gi:16763116)_lnc3 | GCAGCGTGGGCTCGCGTGCCGGGGTTTTTT | TGTCTGAACGGTTTC |  | 15 | 100 | 54.12 | False | 0.00 | 0.00 |
|  | VFG000426(gi:16763116)_detection |  | CGCTGGTCTACAAAG | TTT | 15 | 100 | 54.87 | False | 0.00 | 0.00 |
| lpfB | VFG000455(gi:16766925)_fwd |  | TGATTGCTCAAAACAGTTTT |  | 20 | 114 | 58.10 | False | 0.00 | 0.00 |
|  | VFG000455(gi:16766925)_rev |  | CATCCGTGGTGCTTTTAT |  | 18 | 114 | 58.58 | False | 0.00 | 0.00 |
|  | VFG000455(gi:16766925)_detection |  | AAGGAAGCTTCTCT | TTT | 14 | 114 | 49.76 | False | 0.00 | 0.00 |
|  | VFG000455(gi:16766925)_lnc3 | GCAGCGTGGGCTCGCGTGCCGGGGTTTTTT | TTTATGACGGTAGTAGA |  | 17 | 114 | 52.39 | False | 0.00 | 0.00 |
| yagZ/ecpA | VFG002414(gi:15799998)_fwd |  | YGTGGGCGTGGATTAT |  | 16 | 92 | 58.53 | False | 0.00 | 0.00 |
|  | VFG002414(gi:15799998)_rev |  | TRAGGTTGCCGCCCA |  | 15 | 92 | 62.09 | False | 0.00 | 0.00 |
|  | VFG002414(gi:15799998)_detection |  | TCGATACCGCCA | TTT | 12 | 92 | 51.11 | True | 36.92 | 4.98 |
|  | VFG002414(gi:15799998)_lnc3 | GCAGCGTGGGCTCGCGTGCCGGGGTTTTTT | CGATACCGTGATGA |  | 14 | 92 | 51.64 | False | 0.00 | 0.00 |
| selq | VFG001326(gi:21283666)_fwd |  | CTCACTGTTAGCTTGTTT |  | 18 | 132 | 55.60 | False | 0.00 | 0.00 |
|  | VFG001326(gi:21283666)_rev |  | TCCAGAACTAACTCCTTGAA |  | 20 | 132 | 59.41 | False | 0.00 | 0.00 |
|  | VFG001326(gi:21283666)_detection |  | CATATGCTGATGTAG | TTT | 15 | 132 | 47.69 | False | 0.00 | 0.00 |
|  | VFG001326(gi:21283666)_lnc3 | GCAGCGTGGGCTCGCGTGCCGGGGTTTTTT | TTTAATAAAAAACAATCTAG |  | 20 | 132 | 48.94 | False | 0.00 | 0.00 |
| efaA | VFG002165(gi:29376585)_fwd |  | ACGAATATGAACCGTTACCAGAA |  | 23 | 146 | 64.14 | False | 36.48 | 31.56 |
|  | VFG002165(gi:29376585)_rev |  | GTAATCTTTATTCTCAACTTTTTT |  | 24 | 146 | 56.30 | False | 0.00 | 0.00 |
|  | VFG002165(gi:29376585)_lnc3 | GCAGCGTGGGCTCGCGTGCCGGGGTTTTTT | ATTTTATTCTTTAACGG |  | 17 | 146 | 48.14 | False | 0.00 | 0.00 |
|  | VFG002165(gi:29376585)_detection |  | CTTGAACTTAGAAAC | TTT | 15 | 146 | 45.65 | False | 0.00 | 0.00 |
| lpfC | VFG000454(gi:16766924)_fwd |  | AATTCGGAAGGTCAGTCTTATCG |  | 23 | 127 | 64.49 | False | 0.00 | 0.00 |
|  | VFG000454(gi:16766924)_rev |  | GCACATCCGTCGCTTCC |  | 17 | 127 | 64.28 | False | 0.00 | 0.00 |
|  | VFG000454(gi:16766924)_lnc3 | GCAGCGTGGGCTCGCGTGCCGGGGTTTTTT | GTTACAAGTATTCGA |  | 15 | 127 | 46.91 | False | 0.00 | 0.00 |
|  | VFG000454(gi:16766924)_detection |  | CCAGCGGCTATTAC | TTT | 14 | 127 | 53.50 | False | 0.00 | 0.00 |
| map | VFG001799(gi:8648965)_fwd |  | ATTTAACTAGTCAAATAAAATCAGT |  | 25 | 116 | 57.46 | False | 0.00 | 0.00 |
|  | VFG001799(gi:8648965)_rev |  | TCTTTTACCACCATTTTTAAAA |  | 22 | 116 | 56.53 | False | 0.00 | 0.00 |
|  | VFG001799(gi:8648965)_lnc3 | GCAGCGTGGGCTCGCGTGCCGGGGTTTTTT | AGCATGATAGAGGTATTA |  | 18 | 116 | 53.16 | False | 0.00 | 0.00 |
|  | VFG001799(gi:8648965)_detection |  | GTGAACAAGATTTAAAATAT | TTT | 20 | 116 | 51.54 | False | 0.00 | 0.00 |
| sinH | VFG002307(gi:16765837)_fwd |  | AAGATTTCGACTTTTATGAAGA |  | 22 | 145 | 57.65 | False | 0.00 | 0.00 |
|  | VFG002307(gi:16765837)_rev |  | TTATCGGTGCCAAACAG |  | 17 | 145 | 57.66 | False | 0.00 | 0.00 |
|  | VFG002307(gi:16765837)_lnc3 | GCAGCGTGGGCTCGCGTGCCGGGGTTTTTT | TTTATCCGCAGCTT |  | 14 | 145 | 52.02 | False | 0.00 | 0.00 |
|  | VFG002307(gi:16765837)_detection |  | GGCGCTAAATTAGT | TTT | 14 | 145 | 49.71 | False | 0.00 | 0.00 |
| srtC-3/srtD | VFG005310(gi:15900384)_fwd |  | TTGGAGATGCTCTTTATTATGATA |  | 24 | 104 | 59.67 | False | 0.00 | 0.00 |
|  | VFG005310(gi:15900384)_rev |  | AACCGATTCTAATTTTTCCCAT |  | 22 | 104 | 60.27 | False | 0.00 | 0.00 |
|  | VFG005310(gi:15900384)_detection |  | GATGATGGACACAGAG | TTT | 16 | 104 | 54.70 | False | 0.00 | 0.00 |
|  | VFG005310(gi:15900384)_lnc3 | GCAGCGTGGGCTCGCGTGCCGGGGTTTTTT | GGAAATTGTAGAATATCA |  | 18 | 104 | 50.42 | False | 0.00 | 0.00 |
| hly | VFG000074(gi:16802248)_fwd |  | ACTGGAGCGAAAACAATAA |  | 19 | 126 | 58.47 | False | 0.00 | 0.00 |
|  | VFG000074(gi:16802248)_rev |  | TTCTCCACCATTCCCAAG |  | 18 | 126 | 59.77 | False | 0.00 | 0.00 |
|  | VFG000074(gi:16802248)_lnc3 | GCAGCGTGGGCTCGCGTGCCGGGGTTTTTT | GCTCATTTCACATCG |  | 15 | 126 | 53.04 | False | 0.00 | 0.00 |
|  | VFG000074(gi:16802248)_detection |  | TCCATCTATTTGCC | TTT | 14 | 126 | 48.49 | False | 0.00 | 0.00 |

### VF-3

Supplementary Table S15: Primer and probe sequences and additional information of VF-3.

| Gene | Primer/Probe | Hybridization Sequence | Sequence | Spacer | Length | Product Size | Tm | Hairpin | Hairpin Tm | Hairpin delta G |
| --- | --- | --- | --- | --- | --- | --- | --- | --- | --- | --- |
| cfa/cfb | VFG001333(gi:22538178)_fwd |  | TATTGATATGGGATTTGGG |  | 19 | 109 | 55.03 | False | 0.00 | 0.00 |
|  | VFG001333(gi:22538178)_rev |  | TTGTTCTAATGCCTTTACA |  | 19 | 109 | 55.41 | False | 0.00 | 0.00 |
|  | VFG001333(gi:22538178)_detection |  | CATTTGCTTCAGTTGA | TTT | 16 | 109 | 53.24 | False | 0.00 | 0.00 |
|  | VFG001333(gi:22538178)_lnc3 | GCAGCGTGGGCTCGCGTGCCGGGGTTTTTT | GTTATTCGCATTTTAGATC |  | 19 | 109 | 53.95 | False | 0.00 | 0.00 |
| hlyB | VFG000907(gi:26249408)_fwd |  | GTCAACGCATCGCAATTGC |  | 19 | 140 | 64.80 | False | 38.48 | -117.51 |
|  | VFG000907(gi:26249408)_rev |  | AACCGTTCTGCCCTTAC |  | 17 | 140 | 59.78 | False | 0.00 | 0.00 |
|  | VFG000907(gi:26249408)_detection |  | AGTGCTCTGGATTAT | TTT | 15 | 140 | 51.01 | False | 0.00 | 0.00 |
|  | VFG000907(gi:26249408)_lnc3 | GCAGCGTGGGCTCGCGTGCCGGGGTTTTTT | TTTGATGAAGCAACC |  | 15 | 140 | 52.02 | False | 0.00 | 0.00 |
| cpsA | VFG001352(gi:22537333)_fwd |  | AAGAAAAAGTGATTGCAGCTATT |  | 23 | 118 | 61.60 | False | 0.00 | 0.00 |
|  | VFG001352(gi:22537333)_rev |  | TTATCAATAGTCATATTAGTTTGA |  | 24 | 118 | 55.60 | False | 35.25 | 141.52 |
|  | VFG001352(gi:22537333)_detection |  | CAGTAACAAAATTAAAT | TTT | 17 | 118 | 45.76 | False | 0.00 | 0.00 |
|  | VFG001352(gi:22537333)_lnc3 | GCAGCGTGGGCTCGCGTGCCGGGGTTTTTT | TAGCTTCTAGTCAGT |  | 15 | 118 | 49.68 | False | 0.00 | 0.00 |
| nleC | VFG002122(gi:15800521)_fwd |  | GCAGGAGAAAACGAKAAC |  | 18 | 117 | 57.94 | False | 0.00 | 0.00 |
|  | VFG002122(gi:15800521)_rev |  | AACATGATGAATAATCTCGTG |  | 21 | 117 | 57.79 | False | 39.76 | -210.74 |
|  | VFG002122(gi:15800521)_lnc3 | GCAGCGTGGGCTCGCGTGCCGGGGTTTTTT | CTTATGTCAGTTTTAGTG |  | 18 | 117 | 51.96 | False | 0.00 | 0.00 |
|  | VFG002122(gi:15800521)_detection |  | TTGCGCCAGATACT | TTT | 14 | 117 | 54.75 | False | 0.00 | 0.00 |
| algB | VFG000116(gi:15600676)_fwd |  | CCACCTCGGTTTCAGCG |  | 17 | 147 | 63.82 | False | 43.03 | -409.80 |
|  | VFG000116(gi:15600676)_rev |  | ATACCGAGGGTCTTGGCG |  | 18 | 147 | 64.85 | False | 43.98 | -537.06 |
|  | VFG000116(gi:15600676)_detection |  | AGCCCATATCACGG | TTT | 14 | 147 | 54.95 | False | 0.00 | 0.00 |
|  | VFG000116(gi:15600676)_lnc3 | GCAGCGTGGGCTCGCGTGCCGGGGTTTTTT | GGAAGACCTGGAGAA |  | 15 | 147 | 54.78 | False | 0.00 | 0.00 |
| pefB | VFG000436(gi:17233484)_fwd |  | CAGGCACTCAGGGACTA |  | 17 | 140 | 60.55 | False | 39.61 | -127.46 |
|  | VFG000436(gi:17233484)_rev |  | TAAACAACCATCTGCGC |  | 17 | 140 | 58.45 | False | 0.00 | 0.00 |
|  | VFG000436(gi:17233484)_lnc3 | GCAGCGTGGGCTCGCGTGCCGGGGTTTTTT | CTATGAAGTGAACAAC |  | 16 | 140 | 50.01 | False | 0.00 | 0.00 |
|  | VFG000436(gi:17233484)_detection |  | GGGTATTTCAGTAC | TTT | 14 | 140 | 45.59 | False | 0.00 | 0.00 |
| mpl | VFG000071(gi:16802249)_fwd |  | TTCGCCGATGTYTTTGG |  | 17 | 124 | 59.87 | False | 0.00 | 0.00 |
|  | VFG000071(gi:16802249)_rev |  | CTTGATTATATTTGTCRGGATCTTT |  | 25 | 124 | 60.72 | False | 0.00 | 0.00 |
|  | VFG000071(gi:16802249)_detection |  | CATTGGTTGATTGG | TTT | 14 | 124 | 48.26 | False | 0.00 | 0.00 |
|  | VFG000071(gi:16802249)_lnc3 | GCAGCGTGGGCTCGCGTGCCGGGGTTTTTT | TTATTGCGCCAAAT |  | 14 | 124 | 50.29 | False | 0.00 | 0.00 |
| neuA | VFG001335(gi:22537316)_fwd |  | ATTGTACAGAGAGATTTGTTTAGAA |  | 25 | 179 | 60.76 | False | 0.00 | 0.00 |
|  | VFG001335(gi:22537316)_rev |  | TTATATGCCATGATTTTCTTAGTG |  | 24 | 179 | 59.43 | False | 0.00 | 0.00 |
|  | VFG001335(gi:22537316)_lnc3 | GCAGCGTGGGCTCGCGTGCCGGGGTTTTTT | CAACTTCGTATGATATGTTA |  | 20 | 179 | 54.91 | False | 0.00 | 0.00 |
|  | VFG001335(gi:22537316)_detection |  | AAAGATTTTTTATCTGACT | TTT | 19 | 179 | 51.51 | False | 0.00 | 0.00 |
| fliM | VFG001256(gi:15596640)_fwd |  | CGCTGTTCATCCTCGAC |  | 17 | 83 | 60.77 | False | 0.00 | 0.00 |
|  | VFG001256(gi:15596640)_rev |  | CTCGATCTTGGCGTGGC |  | 17 | 83 | 63.97 | False | 0.00 | 0.00 |
|  | VFG001256(gi:15596640)_lnc3 | GCAGCGTGGGCTCGCGTGCCGGGGTTTTTT | GTTCAAGCTGGTGG |  | 14 | 83 | 54.37 | False | 0.00 | 0.00 |
|  | VFG001256(gi:15596640)_detection |  | ACAACTTCTTCGGC | TTT | 14 | 83 | 53.44 | False | 0.00 | 0.00 |
| fliQ | VFG001260(gi:15596644)_fwd |  | TGGCACTCGACCTGTTC |  | 17 | 124 | 62.31 | False | 0.00 | 0.00 |
|  | VFG001260(gi:15596644)_rev |  | TCGTTGATCTGGGTRGC |  | 17 | 124 | 60.63 | False | 0.00 | 0.00 |
|  | VFG001260(gi:15596644)_lnc3 | GCAGCGTGGGCTCGCGTGCCGGGGTTTTTT | TGATCGTCGGCG |  | 12 | 124 | 54.20 | True | 39.92 | -191.38 |
|  | VFG001260(gi:15596644)_detection |  | TGCTGGTGGTTC | TTT | 12 | 124 | 49.78 | False | 0.00 | 0.00 |
| stx1B | VFG000836(gi:15802645)_fwd |  | TTTCATTTYTTTCAGCAAGT |  | 20 | 156 | 56.84 | False | 37.23 | -17.70 |
|  | VFG000836(gi:15802645)_rev |  | CACTKAGAAGAAGAGACTGAAGA |  | 23 | 156 | 62.42 | False | 0.00 | 0.00 |
|  | VFG000836(gi:15802645)_detection |  | TAATGATGACGATAC | TTT | 15 | 156 | 46.25 | False | 0.00 | 0.00 |
|  | VFG000836(gi:15802645)_lnc3 | GCAGCGTGGGCTCGCGTGCCGGGGTTTTTT | TGGAGTATACAAAATA |  | 16 | 156 | 45.78 | False | 0.00 | 0.00 |
| flgG | VFG001240(gi:15596279)_fwd |  | CGCCCAGGACATGAACC |  | 17 | 104 | 63.56 | False | 0.00 | 0.00 |
|  | VFG001240(gi:15596279)_rev |  | TCTGGTACAGCAGGTCCTG |  | 19 | 104 | 64.50 | True | 63.47 | -1824.46 |
|  | VFG001240(gi:15596279)_detection |  | CTGGCCAACGTATC | TTT | 14 | 104 | 53.81 | False | 0.00 | 0.00 |
|  | VFG001240(gi:15596279)_lnc3 | GCAGCGTGGGCTCGCGTGCCGGGGTTTTTT | CCATTTCCAACAAC |  | 14 | 104 | 48.62 | False | 0.00 | 0.00 |
| rhlI | VFG000153(gi:15598672)_fwd |  | ATTCTGGTCCAGCCTGCA |  | 18 | 141 | 64.73 | False | 40.53 | -224.84 |
|  | VFG000153(gi:15598672)_rev |  | TCGCCCTTGACCTTCTG |  | 17 | 141 | 61.99 | False | 0.00 | 0.00 |
|  | VFG000153(gi:15598672)_lnc3 | GCAGCGTGGGCTCGCGTGCCGGGGTTTTTT | CCATGGAGCGCTA |  | 13 | 141 | 54.24 | False | 0.00 | 0.00 |
|  | VFG000153(gi:15598672)_detection |  | TTTCGTTCGCAAC | TTT | 13 | 141 | 50.55 | False | 0.00 | 0.00 |
| neuC | VFG001337(gi:22537318)_fwd |  | TGACCAACTTTGACAATCCCTA |  | 22 | 102 | 63.60 | False | 0.00 | 0.00 |
|  | VFG001337(gi:22537318)_rev |  | ATGGTTGGGAAATTGAAGGTT |  | 21 | 102 | 62.22 | False | 0.00 | 0.00 |
|  | VFG001337(gi:22537318)_lnc3 | GCAGCGTGGGCTCGCGTGCCGGGGTTTTTT | GAATGCTTCATCTATT |  | 16 | 102 | 48.73 | False | 0.00 | 0.00 |
|  | VFG001337(gi:22537318)_detection |  | GCATATGAAGCAATA | TTT | 15 | 102 | 47.83 | False | 0.00 | 0.00 |
| ebpA | VFG042976(gi:29375667)_fwd |  | TGTTCAGGTTCAAACATTACCAGA |  | 24 | 134 | 64.89 | False | 37.89 | -68.07 |
|  | VFG042976(gi:29375667)_rev |  | TGAAGTTTTCTGACTCTGTTTGA |  | 23 | 134 | 62.50 | False | 0.00 | 0.00 |
|  | VFG042976(gi:29375667)_detection |  | TCAAATTCATTATCAA | TTT | 16 | 134 | 45.09 | False | 0.00 | 0.00 |
|  | VFG042976(gi:29375667)_lnc3 | GCAGCGTGGGCTCGCGTGCCGGGGTTTTTT | AAAGGGCAAGAAAT |  | 14 | 134 | 48.80 | False | 0.00 | 0.00 |
| cpsF | VFG001347(gi:22537328)_fwd |  | TTGATAAAGAAGATGCTAGG |  | 20 | 94 | 55.95 | False | 0.00 | 0.00 |
|  | VFG001347(gi:22537328)_rev |  | TTTTTTACCAAGTTTTTGACA |  | 21 | 94 | 56.56 | False | 40.34 | -231.42 |
|  | VFG001347(gi:22537328)_detection |  | TGTATATCATTGCTT | TTT | 15 | 94 | 45.61 | False | 0.00 | 0.00 |
|  | VFG001347(gi:22537328)_lnc3 | GCAGCGTGGGCTCGCGTGCCGGGGTTTTTT | TCTAAGAGAAGAGAT |  | 15 | 94 | 45.73 | False | 0.00 | 0.00 |
| pilG | VFG001225(gi:15595605)_fwd |  | TGGAACAGCAATCCGACG |  | 18 | 92 | 63.66 | False | 43.81 | -496.27 |
|  | VFG001225(gi:15595605)_rev |  | ACCGACCTTTTTCAGCA |  | 17 | 92 | 59.60 | False | 0.00 | 0.00 |
|  | VFG001225(gi:15595605)_lnc3 | GCAGCGTGGGCTCGCGTGCCGGGGTTTTTT | AAAGTGATGGTGATC |  | 15 | 92 | 50.36 | False | 0.00 | 0.00 |
|  | VFG001225(gi:15595605)_detection |  | GACGATTCGAAAACG | TTT | 15 | 92 | 53.59 | False | 0.00 | 0.00 |
| espD | VFG000799(gi:15804216)_fwd |  | TACTCAATCTGAAACGGG |  | 18 | 114 | 57.12 | False | 0.00 | 0.00 |
|  | VFG000799(gi:15804216)_rev |  | AATGAGTGACCTGCCGG |  | 17 | 114 | 62.41 | False | 0.00 | 0.00 |
|  | VFG000799(gi:15804216)_detection |  | GGCTGGACAGAAAG | TTT | 14 | 114 | 53.51 | False | 0.00 | 0.00 |
|  | VFG000799(gi:15804216)_lnc3 | GCAGCGTGGGCTCGCGTGCCGGGGTTTTTT | TGAACTCGTCAGCA |  | 14 | 114 | 54.60 | False | 0.00 | 0.00 |
| nleA/espI | VFG002121(gi:15801940)_fwd |  | ATCTCTGGTAAATATGGTGT |  | 20 | 128 | 57.06 | False | 0.00 | 0.00 |
|  | VFG002121(gi:15801940)_rev |  | CCAGGAAGGTCTTTAAAAT |  | 19 | 128 | 55.79 | False | 0.00 | 0.00 |
|  | VFG002121(gi:15801940)_lnc3 | GCAGCGTGGGCTCGCGTGCCGGGGTTTTTT | ACATCAGGTAATTATT |  | 16 | 128 | 46.59 | False | 0.00 | 0.00 |
|  | VFG002121(gi:15801940)_detection |  | TTTTACAGATATTCCC | TTT | 16 | 128 | 47.65 | False | 0.00 | 0.00 |
| pilT | VFG000232(gi:15675990)_fwd |  | ATGCAGATTACCGACTT |  | 17 | 95 | 55.80 | False | 0.00 | 0.00 |
|  | VFG000232(gi:15675990)_rev |  | CCGTGAACCCGAATCATAGG |  | 20 | 95 | 64.18 | False | 0.00 | 0.00 |
|  | VFG000232(gi:15675990)_detection |  | CGACCTTCACCTGA | TTT | 14 | 95 | 54.69 | False | 0.00 | 0.00 |
|  | VFG000232(gi:15675990)_lnc3 | GCAGCGTGGGCTCGCGTGCCGGGGTTTTTT | AAAAACAAAGCATC |  | 14 | 95 | 45.37 | False | 0.00 | 0.00 |
| waaG | VFG000139(gi:15600203)_fwd |  | CCAAGCCGTTCCTGCTA |  | 17 | 87 | 62.41 | False | 43.11 | -355.47 |
|  | VFG000139(gi:15600203)_rev |  | GGAAGCGCGGGATATCG |  | 17 | 87 | 63.47 | False | 0.00 | 0.00 |
|  | VFG000139(gi:15600203)_lnc3 | GCAGCGTGGGCTCGCGTGCCGGGGTTTTTT | TCTCAACGACCAGG |  | 14 | 87 | 54.69 | False | 0.00 | 0.00 |
|  | VFG000139(gi:15600203)_detection |  | TACAGATCCTCAAG | TTT | 14 | 87 | 46.58 | False | 0.00 | 0.00 |
| lasB | VFG000146(gi:15598919)_fwd |  | ATGAAGAAGGTTTCTACGC |  | 19 | 98 | 58.65 | False | 44.36 | -431.41 |
|  | VFG000146(gi:15598919)_rev |  | GGGAGTTTGGACACGTC |  | 17 | 98 | 60.54 | False | 0.00 | 0.00 |
|  | VFG000146(gi:15598919)_detection |  | GATCATGGGTGTTT | TTT | 14 | 98 | 49.25 | False | 0.00 | 0.00 |
|  | VFG000146(gi:15598919)_lnc3 | GCAGCGTGGGCTCGCGTGCCGGGGTTTTTT | CTGTTGTTCGTTGC |  | 14 | 98 | 52.88 | False | 0.00 | 0.00 |
| pilF | VFG000233(gi:15676245)_fwd |  | CATCGCTGCTTCAGGAG |  | 17 | 108 | 60.78 | False | 38.35 | -100.78 |
|  | VFG000233(gi:15676245)_rev |  | TATGGATAAACCTCGGAACAGG |  | 22 | 108 | 63.56 | False | 41.84 | -339.88 |
|  | VFG000233(gi:15676245)_detection |  | CACACCCTGTATATCG | TTT | 16 | 108 | 54.45 | False | 0.00 | 0.00 |
|  | VFG000233(gi:15676245)_lnc3 | GCAGCGTGGGCTCGCGTGCCGGGGTTTTTT | AGGAAGAGGAAAGC |  | 14 | 108 | 51.43 | False | 0.00 | 0.00 |
| hlyD | VFG000843(gi:75994496)_fwd |  | ATAGTGGAAGAAGCAAAGAAATAA |  | 24 | 167 | 60.80 | False | 0.00 | 0.00 |
|  | VFG000843(gi:75994496)_rev |  | TTTTTCATACAACAAAGATGCT |  | 22 | 167 | 58.99 | False | 0.00 | 0.00 |
|  | VFG000843(gi:75994496)_detection |  | ATCCATTAGTTGAAT | TTT | 15 | 167 | 45.33 | False | 0.00 | 0.00 |
|  | VFG000843(gi:75994496)_lnc3 | GCAGCGTGGGCTCGCGTGCCGGGGTTTTTT | CTTTTGTAAAAAAAAATG |  | 18 | 167 | 47.71 | False | 0.00 | 0.00 |
| xcpA/pilD | VFG000114(gi:15599724)_fwd |  | YTTCGGTCCCTATCTGGC |  | 18 | 80 | 62.28 | False | 0.00 | 0.00 |
|  | VFG000114(gi:15599724)_rev |  | CGAATTGCAGATAGGTCCGG |  | 20 | 80 | 64.51 | False | 0.00 | 0.00 |
|  | VFG000114(gi:15599724)_detection |  | CTCTGGGGTGATCA | TTT | 14 | 80 | 53.69 | False | 0.00 | 0.00 |
|  | VFG000114(gi:15599724)_lnc3 | GCAGCGTGGGCTCGCGTGCCGGGGTTTTTT | GGTGGATTGCTTTG |  | 14 | 80 | 51.81 | False | 0.00 | 0.00 |
| fliE | VFG001250(gi:15596297)_fwd |  | GACGTGATGATCGCCAG |  | 17 | 85 | 60.86 | False | 0.00 | 0.00 |
|  | VFG001250(gi:15596297)_rev |  | CTTGGTAGGCCTGGACC |  | 17 | 85 | 62.09 | False | 38.01 | -75.97 |
|  | VFG001250(gi:15596297)_detection |  | CATGACCCAGGTG | TTT | 13 | 85 | 51.66 | False | 0.00 | 0.00 |
|  | VFG001250(gi:15596297)_lnc3 | GCAGCGTGGGCTCGCGTGCCGGGGTTTTTT | TGTCGTTCCAGGC |  | 13 | 85 | 54.93 | False | 0.00 | 0.00 |
| pilD | VFG000234(gi:15676247)_fwd |  | GGCAATACGGCTGGTCT |  | 17 | 109 | 62.50 | False | 39.35 | -148.90 |
|  | VFG000234(gi:15676247)_rev |  | GTCATCGAGTCGGGCAG |  | 17 | 109 | 62.92 | False | 0.00 | 0.00 |
|  | VFG000234(gi:15676247)_lnc3 | GCAGCGTGGGCTCGCGTGCCGGGGTTTTTT | CGTTTCTGATTTCC |  | 14 | 109 | 48.09 | False | 0.00 | 0.00 |
|  | VFG000234(gi:15676247)_detection |  | CTGACCTTTATCGA | TTT | 14 | 109 | 47.75 | False | 0.00 | 0.00 |
| hlyA | VFG000906(gi:26249405)_fwd |  | GATATCTTCCATGGCGCG |  | 18 | 131 | 61.67 | False | 0.00 | 0.00 |
|  | VFG000906(gi:26249405)_rev |  | CCATCACCGCCATAGAG |  | 17 | 131 | 59.78 | False | 0.00 | 0.00 |
|  | VFG000906(gi:26249405)_detection |  | TGGTGATAAAGGTAATG | TTT | 17 | 131 | 52.06 | False | 0.00 | 0.00 |
|  | VFG000906(gi:26249405)_lnc3 | GCAGCGTGGGCTCGCGTGCCGGGGTTTTTT | GAATGACCGCTTATA |  | 15 | 131 | 49.90 | False | 0.00 | 0.00 |
| pilG | VFG000235(gi:15676248)_fwd |  | GTATCCGTCATCGCCGT |  | 17 | 124 | 62.27 | False | 37.49 | -34.42 |
|  | VFG000235(gi:15676248)_rev |  | CCATCACTGTTTGGGTCA |  | 18 | 124 | 60.43 | False | 0.00 | 0.00 |
|  | VFG000235(gi:15676248)_lnc3 | GCAGCGTGGGCTCGCGTGCCGGGGTTTTTT | ATGATGATTTTCGTACTG |  | 18 | 124 | 54.09 | False | 0.00 | 0.00 |
|  | VFG000235(gi:15676248)_detection |  | CCCGCCTTTAAAGA | TTT | 14 | 124 | 52.47 | False | 0.00 | 0.00 |
| pilT | VFG001223(gi:15595592)_fwd |  | GATCTACGACATCATGAACG |  | 20 | 119 | 59.66 | False | 0.00 | 0.00 |
|  | VFG001223(gi:15595592)_rev |  | GGTTCTGGTTGAAGGCGT |  | 18 | 119 | 63.55 | False | 39.07 | -140.06 |
|  | VFG001223(gi:15595592)_lnc3 | GCAGCGTGGGCTCGCGTGCCGGGGTTTTTT | GAATTCCTCGAGACC |  | 15 | 119 | 53.65 | False | 0.00 | 0.00 |
|  | VFG001223(gi:15595592)_detection |  | GACTTCTCCTTCGA | TTT | 14 | 119 | 50.78 | False | 0.00 | 0.00 |
| pilH | VFG001226(gi:15595606)_fwd |  | GAGATGTACAAGCTGACC |  | 18 | 107 | 57.95 | False | 0.00 | 0.00 |
|  | VFG001226(gi:15595606)_rev |  | ACRTCGGGCTTTTCCTG |  | 17 | 107 | 61.63 | False | 44.78 | -582.17 |
|  | VFG001226(gi:15595606)_lnc3 | GCAGCGTGGGCTCGCGTGCCGGGGTTTTTT | AAAAGCATGGTCACC |  | 15 | 107 | 54.88 | False | 0.00 | 0.00 |
|  | VFG001226(gi:15595606)_detection |  | AGGTACTCAAGGCC | TTT | 14 | 107 | 54.14 | False | 0.00 | 0.00 |
| neuD | VFG001336(gi:22537317)_fwd |  | GATGGTTATCCTGTTCTTGG |  | 20 | 127 | 59.42 | False | 36.20 | 55.43 |
|  | VFG001336(gi:22537317)_rev |  | TTGCTACATATTCAAATAGTTCT |  | 23 | 127 | 57.85 | False | 0.00 | 0.00 |
|  | VFG001336(gi:22537317)_detection |  | TGAAGATGGCTCAATA | TTT | 16 | 127 | 52.72 | False | 0.00 | 0.00 |
|  | VFG001336(gi:22537317)_lnc3 | GCAGCGTGGGCTCGCGTGCCGGGGTTTTTT | TGTGCTACCTTACCT |  | 15 | 127 | 53.83 | False | 0.00 | 0.00 |
| cpsD | VFG001349(gi:22537330)_fwd |  | GTTGATGCCGCAATAAT |  | 17 | 125 | 56.08 | False | 0.00 | 0.00 |
|  | VFG001349(gi:22537330)_rev |  | AAGAACTTTGAACCACTTTGTT |  | 22 | 125 | 61.07 | False | 0.00 | 0.00 |
|  | VFG001349(gi:22537330)_detection |  | TGAAAAAGCAAAAGAAC | TTT | 17 | 125 | 52.69 | False | 0.00 | 0.00 |
|  | VFG001349(gi:22537330)_lnc3 | GCAGCGTGGGCTCGCGTGCCGGGGTTTTTT | AATAAAACGTAATTATGT |  | 18 | 125 | 48.21 | False | 0.00 | 0.00 |
| flgH | VFG001241(gi:15596280)_fwd |  | CGAGAAGACCCAGGCGA |  | 17 | 82 | 64.24 | False | 0.00 | 0.00 |
|  | VFG001241(gi:15596280)_rev |  | GAACAGCGAGGTCAGCC |  | 17 | 82 | 63.51 | False | 37.36 | -21.26 |
|  | VFG001241(gi:15596280)_lnc3 | GCAGCGTGGGCTCGCGTGCCGGGGTTTTTT | GCCAACTCCGATATC |  | 15 | 82 | 54.24 | False | 0.00 | 0.00 |
|  | VFG001241(gi:15596280)_detection |  | CAGAAGGACAGCAA | TTT | 14 | 82 | 52.40 | False | 0.00 | 0.00 |
| cpsB | VFG001351(gi:22537332)_fwd |  | AGTGATATGTTAGAAAAACTGAAGT |  | 25 | 120 | 61.00 | False | 0.00 | 0.00 |
|  | VFG001351(gi:22537332)_rev |  | ATTTGACAAAGCTGTATGAAT |  | 21 | 120 | 57.81 | False | 40.25 | -180.47 |
|  | VFG001351(gi:22537332)_lnc3 | GCAGCGTGGGCTCGCGTGCCGGGGTTTTTT | CGAAATTTGCTTTAAT |  | 16 | 120 | 48.43 | False | 0.00 | 0.00 |
|  | VFG001351(gi:22537332)_detection |  | TGAATTTTCTATGCAA | TTT | 16 | 120 | 48.72 | False | 0.00 | 0.00 |
| fliG | VFG001252(gi:15596299)_fwd |  | CTGAACACCGTRCAGCC |  | 17 | 129 | 62.55 | False | 39.52 | -181.60 |
|  | VFG001252(gi:15596299)_rev |  | GTAGTTCATGATGTCCGCCG |  | 20 | 129 | 64.75 | False | 0.00 | 0.00 |
|  | VFG001252(gi:15596299)_lnc3 | GCAGCGTGGGCTCGCGTGCCGGGGTTTTTT | GGAACTCAACCTGATC |  | 16 | 129 | 54.95 | False | 0.00 | 0.00 |
|  | VFG001252(gi:15596299)_detection |  | CTGGAGAAGCAGTTC | TTT | 15 | 129 | 54.31 | False | 0.00 | 0.00 |
| pilR | VFG001214(gi:15599743)_fwd |  | TGTGCCTCACCGACATG |  | 17 | 104 | 62.72 | False | 36.94 | 3.93 |
|  | VFG001214(gi:15599743)_rev |  | GTACGCGGTGATCATGGC |  | 18 | 104 | 64.52 | False | 0.00 | 0.00 |
|  | VFG001214(gi:15599743)_lnc3 | GCAGCGTGGGCTCGCGTGCCGGGGTTTTTT | CTCGATCTGGTCCA |  | 14 | 104 | 53.94 | False | 0.00 | 0.00 |
|  | VFG001214(gi:15599743)_detection |  | GTACATCCAGCAGC | TTT | 14 | 104 | 53.79 | False | 0.00 | 0.00 |
| cpsL | VFG001339(gi:22537320)_fwd |  | GTAGGTAATATATGGTTTCTTATT |  | 24 | 169 | 55.98 | False | 0.00 | 0.00 |
|  | VFG001339(gi:22537320)_rev |  | TCCTCTGTTGTTGTATAAAATA |  | 22 | 169 | 56.73 | False | 0.00 | 0.00 |
|  | VFG001339(gi:22537320)_detection |  | AATCTATTTTTTTATTAATG | TTT | 20 | 169 | 46.79 | False | 0.00 | 0.00 |
|  | VFG001339(gi:22537320)_lnc3 | GCAGCGTGGGCTCGCGTGCCGGGGTTTTTT | AGTTGGTTTTTCTG |  | 14 | 169 | 46.86 | False | 0.00 | 0.00 |
| cpsC | VFG001350(gi:22537331)_fwd |  | ATGYTAATCCAAAAATGTCTCA |  | 22 | 128 | 59.21 | False | 0.00 | 0.00 |
|  | VFG001350(gi:22537331)_rev |  | TGGTGMTTTAGGTAAATTTCC |  | 21 | 128 | 58.87 | False | 40.96 | -338.75 |
|  | VFG001350(gi:22537331)_lnc3 | GCAGCGTGGGCTCGCGTGCCGGGGTTTTTT | TAGCAAATTCAGTTAGA |  | 17 | 128 | 51.15 | False | 0.00 | 0.00 |
|  | VFG001350(gi:22537331)_detection |  | GAAGCAGCAGTTTC | TTT | 14 | 128 | 51.95 | False | 0.00 | 0.00 |
| espG | VFG000833(gi:15804250)_fwd |  | CCGGGCCTACACCATTC |  | 17 | 138 | 62.91 | False | 0.00 | 0.00 |
|  | VFG000833(gi:15804250)_rev |  | GTGTGTCTTTAAGTCCTAGTGC |  | 22 | 138 | 62.82 | False | 0.00 | 0.00 |
|  | VFG000833(gi:15804250)_detection |  | CTTTCACTCAATGGT | TTT | 15 | 138 | 50.67 | False | 0.00 | 0.00 |
|  | VFG000833(gi:15804250)_lnc3 | GCAGCGTGGGCTCGCGTGCCGGGGTTTTTT | TCGGCAATGCAAAG |  | 14 | 138 | 54.90 | False | 0.00 | 0.00 |
| pilC | VFG005291(gi:25011516)_fwd |  | AATCAATTACAGATAAATCCTAAG |  | 24 | 136 | 56.75 | False | 0.00 | 0.00 |
|  | VFG005291(gi:25011516)_rev |  | CTGGCTGATTCTGGTTA |  | 17 | 136 | 56.15 | False | 0.00 | 0.00 |
|  | VFG005291(gi:25011516)_detection |  | TAAAGAAAAGGCTAT | TTT | 15 | 136 | 45.29 | False | 0.00 | 0.00 |
|  | VFG005291(gi:25011516)_lnc3 | GCAGCGTGGGCTCGCGTGCCGGGGTTTTTT | ATACAAAAGAAGGAAAGA |  | 18 | 136 | 52.46 | False | 0.00 | 0.00 |
| nspA | VFG037153(gi:15676561)_fwd |  | GGCTACCGCATCAACGA |  | 17 | 92 | 62.86 | False | 40.58 | -200.58 |
|  | VFG037153(gi:15676561)_rev |  | ATGCTGTAAAGTTTGAAATCGG |  | 22 | 92 | 61.71 | False | 0.00 | 0.00 |
|  | VFG037153(gi:15676561)_detection |  | GCGCTACAAAAACTATAA | TTT | 18 | 92 | 54.79 | False | 0.00 | 0.00 |
|  | VFG037153(gi:15676561)_lnc3 | GCAGCGTGGGCTCGCGTGCCGGGGTTTTTT | GCCGTCGATTACAC |  | 14 | 92 | 54.46 | False | 0.00 | 0.00 |
| hlyD | VFG000908(gi:26249409)_fwd |  | ACTGCGTTTAACTTCTTTG |  | 19 | 93 | 57.53 | False | 0.00 | 0.00 |
|  | VFG000908(gi:26249409)_rev |  | TCTGCTCTTTTCTTATCCA |  | 19 | 93 | 56.80 | False | 0.00 | 0.00 |
|  | VFG000908(gi:26249409)_detection |  | TGGCAAAATCAGAAGTA | TTT | 17 | 93 | 54.83 | False | 0.00 | 0.00 |
|  | VFG000908(gi:26249409)_lnc3 | GCAGCGTGGGCTCGCGTGCCGGGGTTTTTT | GAACAGTTTTCCACA |  | 15 | 93 | 51.44 | False | 0.00 | 0.00 |
| neuB | VFG001338(gi:22537319)_fwd |  | TTTAAAACCAATTAAAAAAGGCGA |  | 24 | 151 | 60.91 | False | 38.42 | -146.75 |
|  | VFG001338(gi:22537319)_rev |  | TGAATCACGAATAACTTCAT |  | 20 | 151 | 56.18 | False | 0.00 | 0.00 |
|  | VFG001338(gi:22537319)_lnc3 | GCAGCGTGGGCTCGCGTGCCGGGGTTTTTT | ATGGTATTTCTCCT |  | 14 | 151 | 45.58 | False | 0.00 | 0.00 |
|  | VFG001338(gi:22537319)_detection |  | ATGAACTGGTATGA | TTT | 14 | 151 | 46.23 | False | 0.00 | 0.00 |
| algU | VFG000121(gi:15595959)_fwd |  | GAACAGGATCAGCAACT |  | 17 | 109 | 56.87 | False | 0.00 | 0.00 |
|  | VFG000121(gi:15595959)_rev |  | CGAACCGCACGATCAAT |  | 17 | 109 | 61.10 | False | 0.00 | 0.00 |
|  | VFG000121(gi:15595959)_lnc3 | GCAGCGTGGGCTCGCGTGCCGGGGTTTTTT | TCTGCTGGTACTGA |  | 14 | 109 | 52.63 | False | 0.00 | 0.00 |
|  | VFG000121(gi:15595959)_detection |  | AATACCAGCACAAGA | TTT | 15 | 109 | 52.20 | False | 0.00 | 0.00 |

### VF-4

Supplementary Table S16: Primer and probe sequences and additional information of VF-4.

| Gene | Primer/Probe | Hybridization Sequence | Sequence | Spacer | Length | Product Size | Tm | Hairpin | Hairpin Tm | Hairpin delta G |
| --- | --- | --- | --- | --- | --- | --- | --- | --- | --- | --- |
| fliJ | VFG001255(gi:15596302)_fwd |  | CTACCAGCAGCAATGGATCA |  | 20 | 123 | 63.82 | False | 44.72 | -512.24 |
|  | VFG001255(gi:15596302)_rev |  | CAGGTCACGCTGTTGGC |  | 17 | 123 | 64.48 | False | 37.64 | -41.75 |
|  | VFG001255(gi:15596302)_detection |  | CTTCCTCTCGCAAC | TTT | 14 | 123 | 53.75 | False | 0.00 | 0.00 |
|  | VFG001255(gi:15596302)_lnc3 | GCAGCGTGGGCTCGCGTGCCGGGGTTTTTT | GATGAACTACCAGCG |  | 15 | 123 | 54.58 | False | 0.00 | 0.00 |
| pilF | VFG001217(gi:15599000)_fwd |  | TCTACAAGGAGCGCGAG |  | 17 | 101 | 61.78 | False | 0.00 | 0.00 |
|  | VFG001217(gi:15599000)_rev |  | GAGACGAATGCCCAGCA |  | 17 | 101 | 62.81 | True | 47.25 | -608.10 |
|  | VFG001217(gi:15599000)_detection |  | TCTCTTTGCCCAGG | TTT | 14 | 101 | 55.00 | False | 0.00 | 0.00 |
|  | VFG001217(gi:15599000)_lnc3 | GCAGCGTGGGCTCGCGTGCCGGGGTTTTTT | TCGCCAGTACTATGA |  | 15 | 101 | 53.75 | False | 0.00 | 0.00 |
| chpD | VFG001234(gi:15595613)_fwd |  | TTCGAGAAGCACAGCCAC |  | 18 | 121 | 63.59 | False | 0.00 | 0.00 |
|  | VFG001234(gi:15595613)_rev |  | CCGGGTTGTAGAGGGTC |  | 17 | 121 | 61.73 | False | 0.00 | 0.00 |
|  | VFG001234(gi:15595613)_detection |  | ATGTCTGGCTGGAT | TTT | 14 | 121 | 53.61 | False | 0.00 | 0.00 |
|  | VFG001234(gi:15595613)_lnc3 | GCAGCGTGGGCTCGCGTGCCGGGGTTTTTT | CTGCGGATTGGAAG |  | 14 | 121 | 54.20 | False | 0.00 | 0.00 |
| flhF | VFG001264(gi:15596650)_fwd |  | ACGTGAAGAATTACCTGGTGAT |  | 22 | 112 | 63.70 | False | 0.00 | 0.00 |
|  | VFG001264(gi:15596650)_rev |  | TCCAGCTTAGTCAGGATG |  | 18 | 112 | 58.88 | False | 37.54 | -20.50 |
|  | VFG001264(gi:15596650)_detection |  | CAGACCTATCGGCA | TTT | 14 | 112 | 54.48 | False | 0.00 | 0.00 |
|  | VFG001264(gi:15596650)_lnc3 | GCAGCGTGGGCTCGCGTGCCGGGGTTTTTT | TCAAGTCGGCCTAC |  | 14 | 112 | 54.81 | False | 0.00 | 0.00 |
| pilI | VFG001227(gi:15595607)_fwd |  | CATGCAGCACTTCCCGG |  | 17 | 145 | 64.27 | False | 0.00 | 0.00 |
|  | VFG001227(gi:15595607)_rev |  | GAAGCCCTGGTGTTGCG |  | 17 | 145 | 64.51 | False | 0.00 | 0.00 |
|  | VFG001227(gi:15595607)_detection |  | ATTCATGGCGTCTT | TTT | 14 | 145 | 51.99 | False | 0.00 | 0.00 |
|  | VFG001227(gi:15595607)_lnc3 | GCAGCGTGGGCTCGCGTGCCGGGGTTTTTT | CACTGCAACCCTTC |  | 14 | 145 | 54.37 | False | 0.00 | 0.00 |
| pilK | VFG001229(gi:15595609)_fwd |  | CGGCTGAATGTGCTGGAC |  | 18 | 111 | 64.72 | False | 36.67 | 28.73 |
|  | VFG001229(gi:15595609)_rev |  | GTTGAGGATCTCGCGCC |  | 17 | 111 | 63.64 | False | 38.06 | -46.44 |
|  | VFG001229(gi:15595609)_lnc3 | GCAGCGTGGGCTCGCGTGCCGGGGTTTTTT | CGTGATTTTTTGTCAGA |  | 17 | 111 | 54.35 | False | 0.00 | 0.00 |
|  | VFG001229(gi:15595609)_detection |  | ACCTGCTGATCTACT | TTT | 15 | 111 | 53.53 | False | 0.00 | 0.00 |
| fliP | VFG001259(gi:15596643)_fwd |  | CATGATGATGCTGTCGCC |  | 18 | 85 | 62.22 | False | 36.94 | 3.18 |
|  | VFG001259(gi:15596643)_rev |  | GATGATCAGCGCCCAGC |  | 17 | 85 | 64.09 | True | 48.95 | -723.52 |
|  | VFG001259(gi:15596643)_detection |  | CATGCTGTTCGTCC | TTT | 14 | 85 | 54.68 | False | 0.00 | 0.00 |
|  | VFG001259(gi:15596643)_lnc3 | GCAGCGTGGGCTCGCGTGCCGGGGTTTTTT | CTGCCGTTCAAGAT |  | 14 | 85 | 53.10 | False | 0.00 | 0.00 |
| alg44 | VFG000124(gi:15598738)_fwd |  | CTGAGCACCGCGATCTT |  | 17 | 125 | 62.86 | False | 0.00 | 0.00 |
|  | VFG000124(gi:15598738)_rev |  | GTGATCTGCTGGTTGGG |  | 17 | 125 | 60.62 | False | 0.00 | 0.00 |
|  | VFG000124(gi:15598738)_detection |  | GTACTTCGTCACCC | TTT | 14 | 125 | 52.80 | False | 0.00 | 0.00 |
|  | VFG000124(gi:15598738)_lnc3 | GCAGCGTGGGCTCGCGTGCCGGGGTTTTTT | CCAGATGTACAACCT |  | 15 | 125 | 52.36 | False | 0.00 | 0.00 |
| fleQ | VFG001248(gi:15596294)_fwd |  | AAGATGATCGAGGACGGCA |  | 19 | 124 | 64.62 | False | 42.03 | -387.43 |
|  | VFG001248(gi:15596294)_rev |  | AGATCAGTTCGTTGAGC |  | 17 | 124 | 57.01 | False | 37.21 | -16.66 |
|  | VFG001248(gi:15596294)_lnc3 | GCAGCGTGGGCTCGCGTGCCGGGGTTTTTT | ACCTCTACTACCGC |  | 14 | 124 | 53.53 | False | 0.00 | 0.00 |
|  | VFG001248(gi:15596294)_detection |  | CTCAACGTATTCCCC | TTT | 15 | 124 | 54.01 | False | 0.00 | 0.00 |
| algF | VFG000132(gi:15598746)_fwd |  | TCCAGCGACTTCAAGTT |  | 17 | 161 | 58.92 | False | 0.00 | 0.00 |
|  | VFG000132(gi:15598746)_rev |  | TTCTGCTTGTTCTTGAACGG |  | 20 | 161 | 62.60 | False | 0.00 | 0.00 |
|  | VFG000132(gi:15598746)_detection |  | TACACCCTGGTCAG | TTT | 14 | 161 | 53.62 | False | 0.00 | 0.00 |
|  | VFG000132(gi:15598746)_lnc3 | GCAGCGTGGGCTCGCGTGCCGGGGTTTTTT | CCCGGACAGCTAC |  | 13 | 161 | 54.75 | False | 0.00 | 0.00 |
| flgD | VFG001237(gi:15596276)_fwd |  | AGTTCAGCACCGTGGAA |  | 17 | 124 | 61.83 | False | 0.00 | 0.00 |
|  | VFG001237(gi:15596276)_rev |  | GTCGCCACGATGACCTT |  | 17 | 124 | 62.79 | False | 42.79 | -368.26 |
|  | VFG001237(gi:15596276)_detection |  | GCATTCTCTCCAAC | TTT | 14 | 124 | 50.60 | False | 0.00 | 0.00 |
|  | VFG001237(gi:15596276)_lnc3 | GCAGCGTGGGCTCGCGTGCCGGGGTTTTTT | ACAAGAGCATGGAGA |  | 15 | 124 | 54.77 | False | 0.00 | 0.00 |
| xcpX | VFG000176(gi:15598293)_fwd |  | CGCCAACCGCAGTTTCA |  | 17 | 87 | 64.41 | False | 40.30 | -152.46 |
|  | VFG000176(gi:15598293)_rev |  | CTGACGAACGGCAGCAG |  | 17 | 87 | 64.18 | True | 50.85 | -1008.67 |
|  | VFG000176(gi:15598293)_detection |  | GGAAGCCGACTATC | TTT | 14 | 87 | 52.55 | False | 0.00 | 0.00 |
|  | VFG000176(gi:15598293)_lnc3 | GCAGCGTGGGCTCGCGTGCCGGGGTTTTTT | CTGCTGAAATTGTC |  | 14 | 87 | 48.47 | False | 0.00 | 0.00 |
| xcpZ | VFG000174(gi:15598291)_fwd |  | AGTACTTCACCGAACAG |  | 17 | 132 | 56.14 | False | 0.00 | 0.00 |
|  | VFG000174(gi:15598291)_rev |  | CCGAAGCCGTGACCATG |  | 17 | 132 | 63.91 | False | 0.00 | 0.00 |
|  | VFG000174(gi:15598291)_lnc3 | GCAGCGTGGGCTCGCGTGCCGGGGTTTTTT | GCTGCATGCCTAC |  | 13 | 132 | 53.51 | True | 36.87 | 7.61 |
|  | VFG000174(gi:15598291)_detection |  | ATCCAGCAGCAGG | TTT | 13 | 132 | 54.48 | False | 0.00 | 0.00 |
| fleN | VFG001265(gi:15596651)_fwd |  | GCCAAACTGACCAAGGT |  | 17 | 108 | 60.37 | False | 0.00 | 0.00 |
|  | VFG001265(gi:15596651)_rev |  | GGCTCGCTGTTTCTGCA |  | 17 | 108 | 63.78 | False | 0.00 | 0.00 |
|  | VFG001265(gi:15596651)_lnc3 | GCAGCGTGGGCTCGCGTGCCGGGGTTTTTT | TCGCACTACAGTAT |  | 14 | 108 | 49.85 | False | 0.00 | 0.00 |
|  | VFG001265(gi:15596651)_detection |  | GTAGGTGTCATCCC | TTT | 14 | 108 | 51.77 | False | 0.00 | 0.00 |
| algL | VFG000129(gi:15598743)_fwd |  | GAAATCGAGGACTGGTT |  | 17 | 113 | 56.53 | False | 0.00 | 0.00 |
|  | VFG000129(gi:15598743)_rev |  | GACATCACCGACCAGGC |  | 17 | 113 | 63.20 | True | 49.19 | -650.40 |
|  | VFG000129(gi:15598743)_lnc3 | GCAGCGTGGGCTCGCGTGCCGGGGTTTTTT | GCTGAAGAAGATCA |  | 14 | 113 | 48.56 | False | 0.00 | 0.00 |
|  | VFG000129(gi:15598743)_detection |  | ACAACCATTCCTAC | TTT | 14 | 113 | 48.28 | False | 0.00 | 0.00 |
| fliN | VFG001257(gi:15596641)_fwd |  | AGTGACCACCGAGGAACA |  | 18 | 162 | 63.87 | False | 44.31 | -331.60 |
|  | VFG001257(gi:15596641)_rev |  | GCGCCGAACTCTTCCATC |  | 18 | 162 | 64.46 | False | 0.00 | 0.00 |
|  | VFG001257(gi:15596641)_detection |  | CGATGCGCTGATG | TTT | 13 | 162 | 54.35 | False | 0.00 | 0.00 |
|  | VFG001257(gi:15596641)_lnc3 | GCAGCGTGGGCTCGCGTGCCGGGGTTTTTT | GCCAGGACGACAT |  | 13 | 162 | 54.57 | False | 0.00 | 0.00 |
| algE | VFG000126(gi:15598740)_fwd |  | CCGGGCGAGAAGGACAT |  | 17 | 92 | 64.70 | False | 0.00 | 0.00 |
|  | VFG000126(gi:15598740)_rev |  | ACGTACTGGCTCATCGA |  | 17 | 92 | 60.63 | False | 0.00 | 0.00 |
|  | VFG000126(gi:15598740)_detection |  | GGTGACCAAGTACTTC | TTT | 16 | 92 | 54.87 | False | 0.00 | 0.00 |
|  | VFG000126(gi:15598740)_lnc3 | GCAGCGTGGGCTCGCGTGCCGGGGTTTTTT | GAACTCGACCTGGT |  | 14 | 92 | 54.69 | False | 0.00 | 0.00 |
| pilJ | VFG001228(gi:15595608)_fwd |  | TCGTCTCGATCGTGTTG |  | 17 | 80 | 59.63 | False | 0.00 | 0.00 |
|  | VFG001228(gi:15595608)_rev |  | ATGGCCGATGTACTGCTTG |  | 19 | 80 | 63.97 | False | 0.00 | 0.00 |
|  | VFG001228(gi:15595608)_lnc3 | GCAGCGTGGGCTCGCGTGCCGGGGTTTTTT | ACTTTGCCTACCTC |  | 14 | 80 | 51.46 | False | 0.00 | 0.00 |
|  | VFG001228(gi:15595608)_detection |  | AACACCCAGTCGAA | TTT | 14 | 80 | 54.39 | False | 0.00 | 0.00 |
| flgF | VFG001239(gi:15596278)_fwd |  | GAAAGCAGCAACGTGAAC |  | 18 | 84 | 60.88 | False | 0.00 | 0.00 |
|  | VFG001239(gi:15596278)_rev |  | CATCATCTTCACGTGCAG |  | 18 | 84 | 59.40 | False | 0.00 | 0.00 |
|  | VFG001239(gi:15596278)_lnc3 | GCAGCGTGGGCTCGCGTGCCGGGGTTTTTT | GAAGAGATGACCGC |  | 14 | 84 | 53.44 | False | 0.00 | 0.00 |
|  | VFG001239(gi:15596278)_detection |  | GATCCTCTCGCTGT | TTT | 14 | 84 | 54.50 | False | 0.00 | 0.00 |
| waaC | VFG000142(gi:15600204)_fwd |  | AAYCTCTGGCAGACCCTG |  | 18 | 116 | 63.34 | False | 37.44 | -23.33 |
|  | VFG000142(gi:15600204)_rev |  | GTCAGCCAGGCACTCTT |  | 17 | 116 | 62.34 | False | 40.75 | -83.65 |
|  | VFG000142(gi:15600204)_detection |  | ACCTGGTGATCGAC | TTT | 14 | 116 | 54.79 | False | 0.00 | 0.00 |
|  | VFG000142(gi:15600204)_lnc3 | GCAGCGTGGGCTCGCGTGCCGGGGTTTTTT | GGAAGTCGACTATG |  | 14 | 116 | 49.03 | False | 0.00 | 0.00 |
| aprA | VFG000144(gi:15596446)_fwd |  | ACCTACGCCGAGGACAC |  | 17 | 76 | 64.23 | False | 43.58 | -413.27 |
|  | VFG000144(gi:15596446)_rev |  | CCTTGAAGTCCTGGCCG |  | 17 | 76 | 63.15 | False | 0.00 | 0.00 |
|  | VFG000144(gi:15596446)_lnc3 | GCAGCGTGGGCTCGCGTGCCGGGGTTTTTT | CCTATTCGGTGATGAG |  | 16 | 76 | 54.44 | False | 0.00 | 0.00 |
|  | VFG000144(gi:15596446)_detection |  | CTACTGGGAAGAGCA | TTT | 15 | 76 | 54.92 | False | 0.00 | 0.00 |
| flgI | VFG001242(gi:15596281)_fwd |  | GACCAGACCACGCAGAC |  | 17 | 107 | 63.10 | False | 0.00 | 0.00 |
|  | VFG001242(gi:15596281)_rev |  | ACGTTCTTCAACTGCAC |  | 17 | 107 | 58.29 | False | 0.00 | 0.00 |
|  | VFG001242(gi:15596281)_detection |  | CAGTTCGGCATCAA | TTT | 14 | 107 | 53.55 | False | 0.00 | 0.00 |
|  | VFG001242(gi:15596281)_lnc3 | GCAGCGTGGGCTCGCGTGCCGGGGTTTTTT | CAACATGCTGGCG |  | 13 | 107 | 54.90 | False | 0.00 | 0.00 |
| fliR | VFG001261(gi:15596645)_fwd |  | CGCTGCTGGTGGTGAAC |  | 17 | 113 | 64.48 | False | 0.00 | 0.00 |
|  | VFG001261(gi:15596645)_rev |  | CCAGAGGATCACCAGGC |  | 17 | 113 | 62.17 | False | 41.52 | -169.37 |
|  | VFG001261(gi:15596645)_detection |  | GCTTTCCGTTGACC | TTT | 14 | 113 | 54.96 | False | 0.00 | 0.00 |
|  | VFG001261(gi:15596645)_lnc3 | GCAGCGTGGGCTCGCGTGCCGGGGTTTTTT | ACATCTTCTCCATCG |  | 15 | 113 | 52.68 | False | 0.00 | 0.00 |
| mucB | VFG000135(gi:15595961)_fwd |  | TGCTGCTGAACGAGAAG |  | 17 | 90 | 60.20 | False | 0.00 | 0.00 |
|  | VFG000135(gi:15595961)_rev |  | CCTGCAACTGGTCTTCG |  | 17 | 90 | 60.95 | False | 0.00 | 0.00 |
|  | VFG000135(gi:15595961)_lnc3 | GCAGCGTGGGCTCGCGTGCCGGGGTTTTTT | CGCTTCCAGTTCAC |  | 14 | 90 | 54.58 | False | 0.00 | 0.00 |
|  | VFG000135(gi:15595961)_detection |  | CCAGTTGAATACCGG | TTT | 15 | 90 | 54.40 | False | 0.00 | 0.00 |
| waaP | VFG000140(gi:15600202)_fwd |  | AACAAGGATCTGGCGGC |  | 17 | 85 | 63.17 | False | 0.00 | 0.00 |
|  | VFG000140(gi:15600202)_rev |  | AATAGGTGCGCAGGAAGC |  | 18 | 85 | 63.77 | False | 40.87 | -222.95 |
|  | VFG000140(gi:15600202)_lnc3 | GCAGCGTGGGCTCGCGTGCCGGGGTTTTTT | ATTTCTCTGCGCTG |  | 14 | 85 | 53.67 | False | 0.00 | 0.00 |
|  | VFG000140(gi:15600202)_detection |  | GACATCGGACTGAC | TTT | 14 | 85 | 52.94 | False | 0.00 | 0.00 |
| xcpV | VFG000178(gi:15598295)_fwd |  | CAGGGCGAGCTGGAGTT |  | 17 | 139 | 64.96 | False | 0.00 | 0.00 |
|  | VFG000178(gi:15598295)_rev |  | GTTCCTCGATGCTGCCAC |  | 18 | 139 | 64.42 | False | 0.00 | 0.00 |
|  | VFG000178(gi:15598295)_lnc3 | GCAGCGTGGGCTCGCGTGCCGGGGTTTTTT | CAGGATATGCGGC |  | 13 | 139 | 52.74 | False | 0.00 | 0.00 |
|  | VFG000178(gi:15598295)_detection |  | GGGTGATCGTCTG | TTT | 13 | 139 | 51.51 | False | 0.00 | 0.00 |
| flgJ | VFG001243(gi:15596282)_fwd |  | CGCCTCAACCAGCTCAAG |  | 18 | 148 | 64.35 | False | 0.00 | 0.00 |
|  | VFG001243(gi:15596282)_rev |  | TCATGAAATTGCCGTCG |  | 17 | 148 | 58.54 | False | 0.00 | 0.00 |
|  | VFG001243(gi:15596282)_detection |  | TTCCTCAACGAGATG | TTT | 15 | 148 | 53.00 | False | 0.00 | 0.00 |
|  | VFG001243(gi:15596282)_lnc3 | GCAGCGTGGGCTCGCGTGCCGGGGTTTTTT | GAATTCGAATCGCTG |  | 15 | 148 | 53.21 | False | 0.00 | 0.00 |
| flgE | VFG001238(gi:15596277)_fwd |  | AACGGCTTCTTCGTCACC |  | 18 | 103 | 63.58 | False | 44.15 | -502.66 |
|  | VFG001238(gi:15596277)_rev |  | GGTAGCCGTTGTTGTCG |  | 17 | 103 | 61.35 | False | 0.00 | 0.00 |
|  | VFG001238(gi:15596277)_lnc3 | GCAGCGTGGGCTCGCGTGCCGGGGTTTTTT | GGCTACTTCAATACC |  | 15 | 103 | 50.69 | False | 0.00 | 0.00 |
|  | VFG001238(gi:15596277)_detection |  | GACAAGCAGGATTTC | TTT | 15 | 103 | 52.33 | False | 0.00 | 0.00 |
| alg8 | VFG000123(gi:15598737)_fwd |  | GGTCTACCCGTACTACCG |  | 18 | 129 | 61.46 | False | 0.00 | 0.00 |
|  | VFG000123(gi:15598737)_rev |  | ATCACCGAGCGATAGAC |  | 17 | 129 | 58.69 | False | 39.76 | -84.60 |
|  | VFG000123(gi:15598737)_detection |  | GGTCACCAGTTTCC | TTT | 14 | 129 | 53.38 | False | 0.00 | 0.00 |
|  | VFG000123(gi:15598737)_lnc3 | GCAGCGTGGGCTCGCGTGCCGGGGTTTTTT | CACGTGTTCCTGAT |  | 14 | 129 | 52.59 | False | 0.00 | 0.00 |
| algR | VFG000119(gi:15600454)_fwd |  | GCAATGGCGAAGAAGCG |  | 17 | 119 | 62.87 | False | 40.40 | -212.52 |
|  | VFG000119(gi:15600454)_rev |  | TTCCCGCTCGCAGAGTC |  | 17 | 119 | 64.95 | True | 63.37 | -1873.14 |
|  | VFG000119(gi:15600454)_detection |  | GATATCCGCATGCC | TTT | 14 | 119 | 54.11 | False | 0.00 | 0.00 |
|  | VFG000119(gi:15600454)_lnc3 | GCAGCGTGGGCTCGCGTGCCGGGGTTTTTT | ATATCGTCCTGCTG |  | 14 | 119 | 51.50 | False | 0.00 | 0.00 |
| pilN | VFG001221(gi:15600236)_fwd |  | TCAAGGCGGTGACCCAG |  | 17 | 112 | 64.89 | False | 0.00 | 0.00 |
|  | VFG001221(gi:15600236)_rev |  | CCTTGCGCAACCCCATG |  | 17 | 112 | 64.95 | False | 0.00 | 0.00 |
|  | VFG001221(gi:15600236)_lnc3 | GCAGCGTGGGCTCGCGTGCCGGGGTTTTTT | GGCCAACGTGTTC |  | 13 | 112 | 54.22 | False | 0.00 | 0.00 |
|  | VFG001221(gi:15600236)_detection |  | CAACTGACCGTGC | TTT | 13 | 112 | 53.83 | False | 0.00 | 0.00 |
| flgC | VFG001236(gi:15596275)_fwd |  | CGTGGTCGAGGAAATGGC |  | 18 | 98 | 64.40 | False | 41.90 | -312.43 |
|  | VFG001236(gi:15596275)_rev |  | GGACCTTCTGCATCATCTGTT |  | 21 | 98 | 64.20 | False | 0.00 | 0.00 |
|  | VFG001236(gi:15596275)_detection |  | GCGGAAATGATGAA | TTT | 14 | 98 | 50.56 | False | 0.00 | 0.00 |
|  | VFG001236(gi:15596275)_lnc3 | GCAGCGTGGGCTCGCGTGCCGGGGTTTTTT | CCTTCCAGACCAAC |  | 14 | 98 | 52.93 | False | 0.00 | 0.00 |
| chpC | VFG001233(gi:15595612)_fwd |  | CCCTGCTGTTGCCCAAC |  | 17 | 91 | 64.50 | False | 43.13 | -527.10 |
|  | VFG001233(gi:15595612)_rev |  | TGGCCCAGGTACCACTG |  | 17 | 91 | 64.20 | False | 39.93 | -236.78 |
|  | VFG001233(gi:15595612)_lnc3 | GCAGCGTGGGCTCGCGTGCCGGGGTTTTTT | GTGGCCGAACTGA |  | 13 | 91 | 54.93 | False | 0.00 | 0.00 |
|  | VFG001233(gi:15595612)_detection |  | TCGCCTATCGCAA | TTT | 13 | 91 | 53.71 | True | 39.53 | -117.38 |
| fleR | VFG001249(gi:15596296)_fwd |  | GTCGAGATTCCGTCGCC |  | 17 | 146 | 63.29 | False | 40.85 | -304.35 |
|  | VFG001249(gi:15596296)_rev |  | CTGATGCCAAGGCGCTC |  | 17 | 146 | 64.33 | True | 47.72 | -688.37 |
|  | VFG001249(gi:15596296)_lnc3 | GCAGCGTGGGCTCGCGTGCCGGGGTTTTTT | GCGAATTCCAGGTG |  | 14 | 146 | 54.63 | False | 0.00 | 0.00 |
|  | VFG001249(gi:15596296)_detection |  | ATCATCGATACCCTG | TTT | 15 | 146 | 51.91 | False | 0.00 | 0.00 |
| xcpS | VFG000181(gi:15598298)_fwd |  | CGCAACCAGGAGAACGAC |  | 18 | 134 | 64.30 | False | 0.00 | 0.00 |
|  | VFG000181(gi:15598298)_rev |  | TTGAGAGAAAGGATCGGA |  | 18 | 134 | 58.04 | False | 0.00 | 0.00 |
|  | VFG000181(gi:15598298)_detection |  | CTGATATTCATGGGC | TTT | 15 | 134 | 51.24 | False | 0.00 | 0.00 |
|  | VFG000181(gi:15598298)_lnc3 | GCAGCGTGGGCTCGCGTGCCGGGGTTTTTT | TCGAACCGTTCATG |  | 14 | 134 | 53.22 | False | 0.00 | 0.00 |
| algZ | VFG000120(gi:15600455)_fwd |  | AACCCCTACGACGAGGC |  | 17 | 145 | 64.26 | False | 38.95 | -135.27 |
|  | VFG000120(gi:15600455)_rev |  | GACAGGTGTAGTGCCGT |  | 17 | 145 | 61.97 | False | 0.00 | 0.00 |
|  | VFG000120(gi:15600455)_detection |  | GATGCACGACTTGG | TTT | 14 | 145 | 54.68 | False | 0.00 | 0.00 |
|  | VFG000120(gi:15600455)_lnc3 | GCAGCGTGGGCTCGCGTGCCGGGGTTTTTT | GGCGTTACACAATATC |  | 16 | 145 | 53.39 | False | 0.00 | 0.00 |
| fliI | VFG001254(gi:15596301)_fwd |  | GGCATCCGCTCGATCAA |  | 17 | 188 | 62.95 | False | 40.90 | -235.92 |
|  | VFG001254(gi:15596301)_rev |  | AGGATCTCGTCGATGAA |  | 17 | 188 | 57.37 | False | 35.43 | 92.65 |
|  | VFG001254(gi:15596301)_lnc3 | GCAGCGTGGGCTCGCGTGCCGGGGTTTTTT | CAAGTCGGTGCTG |  | 13 | 188 | 53.37 | False | 0.00 | 0.00 |
|  | VFG001254(gi:15596301)_detection |  | CTGGGGATGATGA | TTT | 13 | 188 | 48.98 | False | 0.00 | 0.00 |
| xcpW | VFG000177(gi:15598294)_fwd |  | GTGCTGGACGGGGTTAC |  | 17 | 93 | 63.13 | False | 0.00 | 0.00 |
|  | VFG000177(gi:15598294)_rev |  | CTCCTCGCTGCCTTCGT |  | 17 | 93 | 64.96 | False | 0.00 | 0.00 |
|  | VFG000177(gi:15598294)_lnc3 | GCAGCGTGGGCTCGCGTGCCGGGGTTTTTT | CTCGACAAGGAGC |  | 13 | 93 | 52.03 | False | 0.00 | 0.00 |
|  | VFG000177(gi:15598294)_detection |  | ACAACTGGCAGGG | TTT | 13 | 93 | 54.68 | False | 0.00 | 0.00 |
| algA | VFG000133(gi:15598747)_fwd |  | GCCTCTTTCCCGCAAGC |  | 17 | 135 | 64.59 | False | 0.00 | 0.00 |
|  | VFG000133(gi:15598747)_rev |  | CGGTGCTCCTTGTTGCA |  | 17 | 135 | 63.72 | False | 38.74 | -123.52 |
|  | VFG000133(gi:15598747)_detection |  | GCAGACCATCAAGC | TTT | 14 | 135 | 54.61 | False | 0.00 | 0.00 |
|  | VFG000133(gi:15598747)_lnc3 | GCAGCGTGGGCTCGCGTGCCGGGGTTTTTT | GACACCCTGTTCCA |  | 14 | 135 | 54.92 | False | 0.00 | 0.00 |
| xcpU | VFG000179(gi:15598296)_fwd |  | GCTGACCTTCGAGCTGG |  | 17 | 117 | 63.19 | False | 35.64 | 105.61 |
|  | VFG000179(gi:15598296)_rev |  | CTGAGTTCGCCACTGGA |  | 17 | 117 | 61.98 | False | 40.47 | -174.64 |
|  | VFG000179(gi:15598296)_detection |  | AGCAGAAGAAAGG | TTT | 13 | 117 | 47.07 | False | 0.00 | 0.00 |
|  | VFG000179(gi:15598296)_lnc3 | GCAGCGTGGGCTCGCGTGCCGGGGTTTTTT | AAGGGCGAGAAGG |  | 13 | 117 | 53.93 | False | 0.00 | 0.00 |
| chpE | VFG001235(gi:15595614)_fwd |  | ATGCTCGCCATCTTCCT |  | 17 | 78 | 61.41 | False | 0.00 | 0.00 |
|  | VFG001235(gi:15595614)_rev |  | CGTTTCGCTGAACACCG |  | 17 | 78 | 62.37 | False | 0.00 | 0.00 |
|  | VFG001235(gi:15595614)_lnc3 | GCAGCGTGGGCTCGCGTGCCGGGGTTTTTT | TGCTGTTCGGTTTC |  | 14 | 78 | 53.88 | False | 0.00 | 0.00 |
|  | VFG001235(gi:15595614)_detection |  | GCCTTCAATGTTTC | TTT | 14 | 78 | 48.81 | False | 0.00 | 0.00 |
| flhB | VFG001262(gi:15596646)_fwd |  | AGCTGGAYCAGGAGATTCC |  | 19 | 121 | 63.34 | False | 39.02 | -114.49 |
|  | VFG001262(gi:15596646)_rev |  | AGGTCCTTCAACGGGCT |  | 17 | 121 | 63.78 | False | 0.00 | 0.00 |
|  | VFG001262(gi:15596646)_detection |  | TCAAGCAGTACCAG | TTT | 14 | 121 | 51.55 | False | 0.00 | 0.00 |
|  | VFG001262(gi:15596646)_lnc3 | GCAGCGTGGGCTCGCGTGCCGGGGTTTTTT | CCTATGTCTACCAGC |  | 15 | 121 | 52.79 | False | 0.00 | 0.00 |
| plcH | VFG000157(gi:15596041)_fwd |  | CGGTATTCCAGGTGCAGC |  | 18 | 128 | 64.13 | False | 0.00 | 0.00 |
|  | VFG000157(gi:15596041)_rev |  | GAKCAGGTCGTCGAGTT |  | 17 | 128 | 60.45 | False | 0.00 | 0.00 |
|  | VFG000157(gi:15596041)_lnc3 | GCAGCGTGGGCTCGCGTGCCGGGGTTTTTT | GTGACCAGCTATCC |  | 14 | 128 | 52.36 | False | 0.00 | 0.00 |
|  | VFG000157(gi:15596041)_detection |  | GGTGGTCCAGGAAA | TTT | 14 | 128 | 54.87 | False | 0.00 | 0.00 |
| algX | VFG000128(gi:15598742)_fwd |  | CGACATCCTCAACAACG |  | 17 | 140 | 58.56 | False | 0.00 | 0.00 |
|  | VFG000128(gi:15598742)_rev |  | TCTTCTGCGCCATGTCG |  | 17 | 140 | 63.18 | False | 0.00 | 0.00 |
|  | VFG000128(gi:15598742)_lnc3 | GCAGCGTGGGCTCGCGTGCCGGGGTTTTTT | CTGGCGTACATGAC |  | 14 | 140 | 53.87 | False | 0.00 | 0.00 |
|  | VFG000128(gi:15598742)_detection |  | CAGCGAGGAATTCC | TTT | 14 | 140 | 53.79 | False | 0.00 | 0.00 |
| tir | VFG000805(gi:15804222)_fwd |  | GATTCGCAGGTGCCGAC |  | 17 | 89 | 64.28 | False | 40.34 | -190.43 |
|  | VFG000805(gi:15804222)_rev |  | GGATGTTGAATGGTGCTATA |  | 20 | 89 | 58.83 | False | 0.00 | 0.00 |
|  | VFG000805(gi:15804222)_lnc3 | GCAGCGTGGGCTCGCGTGCCGGGGTTTTTT | TACGTCTGTTCAGA |  | 14 | 89 | 49.65 | False | 0.00 | 0.00 |
|  | VFG000805(gi:15804222)_detection |  | ATATGGGGAATACAGAT | TTT | 17 | 89 | 52.30 | False | 0.00 | 0.00 |

### VF-5

Supplementary Table S17: Primer and probe sequences and additional information of VF-5.

| Gene | Primer/Probe | Hybridization Sequence | Sequence | Spacer | Length | Product Size | Tm | Hairpin | Hairpin Tm | Hairpin delta G |
| --- | --- | --- | --- | --- | --- | --- | --- | --- | --- | --- |
| fliO | VFG001258(gi:15596642)_fwd |  | CGCAACTGGCCCAACTG |  | 17 | 117 | 64.83 | False | 43.10 | -304.91 |
|  | VFG001258(gi:15596642)_rev |  | TGCGGATCAAGCGGTTG |  | 17 | 117 | 63.85 | False | 39.65 | -166.00 |
|  | VFG001258(gi:15596642)_detection |  | TCTTCCTGCTCGC | TTT | 13 | 117 | 54.63 | False | 0.00 | 0.00 |
|  | VFG001258(gi:15596642)_lnc3 | GCAGCGTGGGCTCGCGTGCCGGGGTTTTTT | GTGATCGGCCTGA |  | 13 | 117 | 54.13 | False | 0.00 | 0.00 |
| fliF | VFG001251(gi:15596298)_fwd |  | GATCGACAGCCAGGTTC |  | 17 | 124 | 60.37 | False | 0.00 | 0.00 |
|  | VFG001251(gi:15596298)_rev |  | GACCAGCAGGCCGATCT |  | 17 | 124 | 64.71 | False | 41.89 | -240.62 |
|  | VFG001251(gi:15596298)_detection |  | GACAACCTTTCCGAG | TTT | 15 | 124 | 54.74 | False | 0.00 | 0.00 |
|  | VFG001251(gi:15596298)_lnc3 | GCAGCGTGGGCTCGCGTGCCGGGGTTTTTT | GACTGTCGTTCCTC |  | 14 | 124 | 52.84 | False | 0.00 | 0.00 |
| speB | VFG000973(gi:15675811)_fwd |  | CAAAACTTTGCTCGTAACGA |  | 20 | 120 | 60.79 | False | 0.00 | 0.00 |
|  | VFG000973(gi:15675811)_rev |  | GTTAACTTTGTCAAGCTTAATAT |  | 23 | 120 | 57.31 | False | 36.88 | 7.69 |
|  | VFG000973(gi:15675811)_lnc3 | GCAGCGTGGGCTCGCGTGCCGGGGTTTTTT | ATAGCGCTATCACATTT |  | 17 | 120 | 54.89 | False | 0.00 | 0.00 |
|  | VFG000973(gi:15675811)_detection |  | ATCCAAAAATCAGCA | TTT | 15 | 120 | 50.51 | False | 0.00 | 0.00 |
| chpA | VFG001231(gi:15595610)_fwd |  | CTGCCGTTCACCGTGTC |  | 17 | 118 | 64.11 | False | 36.21 | 42.65 |
|  | VFG001231(gi:15595610)_rev |  | CCTCCAGTTCGTAGGGC |  | 17 | 118 | 62.14 | False | 0.00 | 0.00 |
|  | VFG001231(gi:15595610)_detection |  | GGCGAGGATCTCTA | TTT | 14 | 118 | 53.19 | False | 0.00 | 0.00 |
|  | VFG001231(gi:15595610)_lnc3 | GCAGCGTGGGCTCGCGTGCCGGGGTTTTTT | CTGATGGTGCTATCC |  | 15 | 118 | 53.67 | False | 0.00 | 0.00 |
| pilV | VFG001207(gi:15599747)_fwd |  | TGTACGACGTCAAGGAC |  | 17 | 137 | 59.12 | False | 0.00 | 0.00 |
|  | VFG001207(gi:15599747)_rev |  | CTGTTCCGCCCAGCATC |  | 17 | 137 | 63.95 | False | 0.00 | 0.00 |
|  | VFG001207(gi:15599747)_detection |  | TTCAAGGCCAAGG | TTT | 13 | 137 | 51.28 | False | 0.00 | 0.00 |
|  | VFG001207(gi:15599747)_lnc3 | GCAGCGTGGGCTCGCGTGCCGGGGTTTTTT | GCAATCCGACTTC |  | 13 | 137 | 49.80 | False | 0.00 | 0.00 |
| lasI | VFG000154(gi:15596629)_fwd |  | TTCAAGGAGCGCAAAGGC |  | 18 | 99 | 64.62 | False | 0.00 | 0.00 |
|  | VFG000154(gi:15596629)_rev |  | ATCTTCCTGGATCAACATGTAAT |  | 23 | 99 | 61.76 | False | 0.00 | 0.00 |
|  | VFG000154(gi:15596629)_detection |  | CGAGATGGAAATCG | TTT | 14 | 99 | 50.59 | False | 0.00 | 0.00 |
|  | VFG000154(gi:15596629)_lnc3 | GCAGCGTGGGCTCGCGTGCCGGGGTTTTTT | CGTTAGTGTCATCGA |  | 15 | 99 | 53.26 | False | 0.00 | 0.00 |
| pilP | VFG001219(gi:15600234)_fwd |  | CATCAGCGAAGGAAAAAT |  | 18 | 92 | 56.50 | False | 0.00 | 0.00 |
|  | VFG001219(gi:15600234)_rev |  | GTTCCTTGAGAGTCAGGCTG |  | 20 | 92 | 63.99 | False | 0.00 | 0.00 |
|  | VFG001219(gi:15600234)_lnc3 | GCAGCGTGGGCTCGCGTGCCGGGGTTTTTT | GAAATCGTTCCTGAC |  | 15 | 92 | 52.03 | False | 0.00 | 0.00 |
|  | VFG001219(gi:15600234)_detection |  | GGAGAGGGGAACTG | TTT | 14 | 92 | 54.72 | False | 0.00 | 0.00 |
| algK | VFG000125(gi:15598739)_fwd |  | GCCAAGCTGCTCTACGA |  | 17 | 132 | 62.46 | False | 0.00 | 0.00 |
|  | VFG000125(gi:15598739)_rev |  | CCACTTGCCGTCGTAGTA |  | 18 | 132 | 62.32 | False | 0.00 | 0.00 |
|  | VFG000125(gi:15598739)_detection |  | TACCTGAAGAACGC | TTT | 14 | 132 | 52.20 | False | 0.00 | 0.00 |
|  | VFG000125(gi:15598739)_lnc3 | GCAGCGTGGGCTCGCGTGCCGGGGTTTTTT | AAAAGATGCTCGGC |  | 14 | 132 | 54.05 | False | 0.00 | 0.00 |
| hasC | VFG000964(gi:15675935)_fwd |  | CCAAAGTCAGAAAAGCCATTATTC |  | 24 | 127 | 62.97 | False | 0.00 | 0.00 |
|  | VFG000964(gi:15675935)_rev |  | TCTTCGACGATAAACTGGAT |  | 20 | 127 | 60.01 | False | 0.00 | 0.00 |
|  | VFG000964(gi:15675935)_detection |  | TACCTGCTACCAAA | TTT | 14 | 127 | 49.79 | False | 0.00 | 0.00 |
|  | VFG000964(gi:15675935)_lnc3 | GCAGCGTGGGCTCGCGTGCCGGGGTTTTTT | CTAGGAACACGTTTTT |  | 16 | 127 | 52.44 | False | 0.00 | 0.00 |
| pilE | VFG001212(gi:15599752)_fwd |  | ATGAGGACAAGACAGAAG |  | 18 | 104 | 56.68 | False | 0.00 | 0.00 |
|  | VFG001212(gi:15599752)_rev |  | ACGTAGTTCTGGTARCTGGG |  | 20 | 104 | 63.85 | True | 46.13 | -151.51 |
|  | VFG001212(gi:15599752)_detection |  | TGGTGGTGGTAGTG | TTT | 14 | 104 | 54.49 | False | 0.00 | 0.00 |
|  | VFG001212(gi:15599752)_lnc3 | GCAGCGTGGGCTCGCGTGCCGGGGTTTTTT | CACGTTGCTGGAAA |  | 14 | 104 | 54.29 | False | 0.00 | 0.00 |
| mucC | VFG000136(gi:15595962)_fwd |  | TGAGCGACCTGAGCCTG |  | 17 | 92 | 64.59 | False | 36.94 | 3.55 |
|  | VFG000136(gi:15595962)_rev |  | GAAAAGATAGAAGAGGACGGA |  | 21 | 92 | 60.63 | False | 0.00 | 0.00 |
|  | VFG000136(gi:15595962)_lnc3 | GCAGCGTGGGCTCGCGTGCCGGGGTTTTTT | CCTAGGAATTCATG |  | 14 | 92 | 45.60 | False | 0.00 | 0.00 |
|  | VFG000136(gi:15595962)_detection |  | AAGACCTGTTGTTG | TTT | 14 | 92 | 49.58 | False | 0.00 | 0.00 |
| gbs0628 | VFG005277(gi:25010688)_fwd |  | GACAATGTAAAAGGTTTGC |  | 19 | 130 | 56.34 | False | 0.00 | 0.00 |
|  | VFG005277(gi:25010688)_rev |  | CTTCAAGAATCGTTCCAACT |  | 20 | 130 | 59.58 | False | 0.00 | 0.00 |
|  | VFG005277(gi:25010688)_detection |  | TTGATGAATTGAAAAAA | TTT | 17 | 130 | 48.40 | False | 0.00 | 0.00 |
|  | VFG005277(gi:25010688)_lnc3 | GCAGCGTGGGCTCGCGTGCCGGGGTTTTTT | GACGGATATTTCTG |  | 14 | 130 | 46.47 | False | 0.00 | 0.00 |
| pilO | VFG001220(gi:15600235)_fwd |  | GGCACAGATGAARGAGATG |  | 19 | 139 | 60.40 | False | 0.00 | 0.00 |
|  | VFG001220(gi:15600235)_rev |  | CAGCTTGATYTCCTCGAACT |  | 20 | 139 | 62.48 | False | 0.00 | 0.00 |
|  | VFG001220(gi:15600235)_lnc3 | GCAGCGTGGGCTCGCGTGCCGGGGTTTTTT | CTGCTCGAGGACA |  | 13 | 139 | 53.61 | False | 0.00 | 0.00 |
|  | VFG001220(gi:15600235)_detection |  | TCACTCGTACCGG | TTT | 13 | 139 | 53.22 | False | 0.00 | 0.00 |
| pilU | VFG001224(gi:15595593)_fwd |  | GTATTGCGCCGGATCGA |  | 17 | 102 | 63.00 | False | 0.00 | 0.00 |
|  | VFG001224(gi:15595593)_rev |  | GAAGATCACCAGGCCGC |  | 17 | 102 | 63.62 | False | 0.00 | 0.00 |
|  | VFG001224(gi:15595593)_lnc3 | GCAGCGTGGGCTCGCGTGCCGGGGTTTTTT | TGGAAGAGCTGAAG |  | 14 | 102 | 51.52 | False | 0.00 | 0.00 |
|  | VFG001224(gi:15595593)_detection |  | CTCCCGGAAATCCT | TTT | 14 | 102 | 54.24 | False | 0.00 | 0.00 |
| pilX | VFG001209(gi:15599749)_fwd |  | GCGCTGAACCTGAAGCT |  | 17 | 138 | 63.46 | False | 40.91 | -297.58 |
|  | VFG001209(gi:15599749)_rev |  | CCACTGCACGCTGTAGA |  | 17 | 138 | 62.37 | False | 38.91 | -162.81 |
|  | VFG001209(gi:15599749)_detection |  | TGGATGAGTTACCG | TTT | 14 | 138 | 51.25 | False | 0.00 | 0.00 |
|  | VFG001209(gi:15599749)_lnc3 | GCAGCGTGGGCTCGCGTGCCGGGGTTTTTT | GCATCGCCAATACC |  | 14 | 138 | 54.84 | False | 0.00 | 0.00 |
| waaF | VFG000143(gi:15600205)_fwd |  | GAGGCGAAGATCCGTGC |  | 17 | 129 | 63.64 | False | 0.00 | 0.00 |
|  | VFG000143(gi:15600205)_rev |  | CTCCCCGGCAAGATTGA |  | 17 | 129 | 62.09 | False | 0.00 | 0.00 |
|  | VFG000143(gi:15600205)_detection |  | GGTGGAGAGGAGATT | TTT | 15 | 129 | 54.45 | False | 0.00 | 0.00 |
|  | VFG000143(gi:15600205)_lnc3 | GCAGCGTGGGCTCGCGTGCCGGGGTTTTTT | AGAACGACCATCCC |  | 14 | 129 | 54.74 | False | 0.00 | 0.00 |
| exoS | VFG000147(gi:15599036)_fwd |  | CACTACGCGGACCTGAATC |  | 19 | 128 | 63.95 | False | 0.00 | 0.00 |
|  | VFG000147(gi:15599036)_rev |  | AAAGTCTTCACTACCTGTTCA |  | 21 | 128 | 60.64 | False | 34.98 | 112.58 |
|  | VFG000147(gi:15599036)_lnc3 | GCAGCGTGGGCTCGCGTGCCGGGGTTTTTT | GACAAAAGCTGATC |  | 14 | 128 | 48.05 | False | 0.00 | 0.00 |
|  | VFG000147(gi:15599036)_detection |  | GACCAAGGTATGTC | TTT | 14 | 128 | 49.15 | False | 0.00 | 0.00 |
| pilQ | VFG001218(gi:15600233)_fwd |  | GACAATCGCCTGACCGTC |  | 18 | 112 | 64.07 | False | 0.00 | 0.00 |
|  | VFG001218(gi:15600233)_rev |  | TGTCCTGGAAGTTCAGCG |  | 18 | 112 | 62.90 | False | 36.92 | 6.83 |
|  | VFG001218(gi:15600233)_detection |  | TTCGCCTATACCGG | TTT | 14 | 112 | 54.61 | False | 0.00 | 0.00 |
|  | VFG001218(gi:15600233)_lnc3 | GCAGCGTGGGCTCGCGTGCCGGGGTTTTTT | GCAAGAAGGACAAT |  | 14 | 112 | 49.77 | False | 0.00 | 0.00 |
| algJ | VFG000131(gi:15598745)_fwd |  | CTCAACGGCAAGCTGGC |  | 17 | 110 | 64.89 | False | 41.82 | -341.38 |
|  | VFG000131(gi:15598745)_rev |  | TCGTGGAACAGCGTGTA |  | 17 | 110 | 61.55 | True | 46.58 | -281.59 |
|  | VFG000131(gi:15598745)_lnc3 | GCAGCGTGGGCTCGCGTGCCGGGGTTTTTT | CACTACGACAAGGA |  | 14 | 110 | 51.24 | False | 0.00 | 0.00 |
|  | VFG000131(gi:15598745)_detection |  | ATTCCCGATCAAGC | TTT | 14 | 110 | 52.71 | False | 0.00 | 0.00 |
| toxA | VFG000115(gi:15596345)_fwd |  | GGGTCTACAACTACCTC |  | 17 | 107 | 55.49 | False | 39.35 | -191.76 |
|  | VFG000115(gi:15596345)_rev |  | GATGTCCAGGTCATGCTT |  | 18 | 107 | 60.27 | False | 0.00 | 0.00 |
|  | VFG000115(gi:15596345)_detection |  | GCAAGATCTACCGG | TTT | 14 | 107 | 52.97 | False | 0.00 | 0.00 |
|  | VFG000115(gi:15596345)_lnc3 | GCAGCGTGGGCTCGCGTGCCGGGGTTTTTT | CGATACCTGGGAAG |  | 14 | 107 | 51.94 | False | 0.00 | 0.00 |
| algI | VFG000130(gi:15598744)_fwd |  | TCACCTCGTTCGGCATG |  | 17 | 142 | 62.78 | False | 0.00 | 0.00 |
|  | VFG000130(gi:15598744)_rev |  | AAGTCGATCAGGTTGTGGG |  | 19 | 142 | 63.16 | False | 0.00 | 0.00 |
|  | VFG000130(gi:15598744)_detection |  | TCAGCTACATCATCG | TTT | 15 | 142 | 52.86 | False | 0.00 | 0.00 |
|  | VFG000130(gi:15598744)_lnc3 | GCAGCGTGGGCTCGCGTGCCGGGGTTTTTT | CACCTTCGAGTCGA |  | 14 | 142 | 54.91 | False | 0.00 | 0.00 |
| mucA | VFG000134(gi:15595960)_fwd |  | ATGAGTCGTGAAGCCCT |  | 17 | 113 | 60.89 | False | 0.00 | 0.00 |
|  | VFG000134(gi:15595960)_rev |  | GAACGCAGCTCGGCATC |  | 17 | 113 | 64.65 | True | 46.10 | -572.96 |
|  | VFG000134(gi:15595960)_lnc3 | GCAGCGTGGGCTCGCGTGCCGGGGTTTTTT | AAACTCTGTCCGCT |  | 14 | 113 | 54.52 | False | 0.00 | 0.00 |
|  | VFG000134(gi:15595960)_detection |  | GTGATGGATAACGA | TTT | 14 | 113 | 48.32 | False | 0.00 | 0.00 |
| stx1A | VFG000835(gi:15802646)_fwd |  | GGGATTTCGTACAACACT |  | 18 | 132 | 57.77 | False | 0.00 | 0.00 |
|  | VFG000835(gi:15802646)_rev |  | CGAACAGAGTCTTGTCCA |  | 18 | 132 | 59.78 | False | 0.00 | 0.00 |
|  | VFG000835(gi:15802646)_lnc3 | GCAGCGTGGGCTCGCGTGCCGGGGTTTTTT | TCTTATGTAATGACTG |  | 16 | 132 | 47.44 | False | 0.00 | 0.00 |
|  | VFG000835(gi:15802646)_detection |  | CTGAAGATGTTGAT | TTT | 14 | 132 | 45.36 | False | 0.00 | 0.00 |
| pilW | VFG001208(gi:15599748)_fwd |  | CTGTCCATGATCGAACTAC |  | 19 | 129 | 58.51 | False | 0.00 | 0.00 |
|  | VFG001208(gi:15599748)_rev |  | GTTTTCCTGGTTGCCTG |  | 17 | 129 | 59.02 | False | 0.00 | 0.00 |
|  | VFG001208(gi:15599748)_lnc3 | GCAGCGTGGGCTCGCGTGCCGGGGTTTTTT | CTTCCTGATTCTCGG |  | 15 | 129 | 53.63 | False | 0.00 | 0.00 |
|  | VFG001208(gi:15599748)_detection |  | GATTACCCAGATCTAC | TTT | 16 | 129 | 50.41 | False | 0.00 | 0.00 |
| xcpQ | VFG000184(gi:15598301)_fwd |  | TCGAAGACGTGATCCGC |  | 17 | 104 | 62.50 | False | 44.07 | -374.46 |
|  | VFG000184(gi:15598301)_rev |  | GTTGTTGAGCACCTCGGC |  | 18 | 104 | 64.94 | False | 36.61 | 24.79 |
|  | VFG000184(gi:15598301)_detection |  | CGTGATCAACCTGC | TTT | 14 | 104 | 54.68 | False | 0.00 | 0.00 |
|  | VFG000184(gi:15598301)_lnc3 | GCAGCGTGGGCTCGCGTGCCGGGGTTTTTT | AGCCACGACTACAG |  | 14 | 104 | 54.84 | False | 0.00 | 0.00 |
| exoT | VFG000148(gi:15595242)_fwd |  | CGATAACCACCCTGTTCGG |  | 19 | 133 | 63.92 | False | 0.00 | 0.00 |
|  | VFG000148(gi:15595242)_rev |  | ACACCCTGCCCATCCTT |  | 17 | 133 | 63.85 | False | 0.00 | 0.00 |
|  | VFG000148(gi:15595242)_detection |  | GATGAGCAGGAGAT | TTT | 14 | 133 | 50.82 | False | 0.00 | 0.00 |
|  | VFG000148(gi:15595242)_lnc3 | GCAGCGTGGGCTCGCGTGCCGGGGTTTTTT | TATCGATCGAGGGC |  | 14 | 133 | 54.33 | False | 0.00 | 0.00 |
| iap/cwhA | VFG000070(gi:16802625)_fwd |  | GCTCAAAAACACCTTGGAAAA |  | 21 | 116 | 61.81 | False | 0.00 | 0.00 |
|  | VFG000070(gi:16802625)_rev |  | GTACGTGGAAGGGAGAT |  | 17 | 116 | 58.05 | False | 0.00 | 0.00 |
|  | VFG000070(gi:16802625)_detection |  | CGGACCAACTACAT | TTT | 14 | 116 | 51.67 | False | 0.00 | 0.00 |
|  | VFG000070(gi:16802625)_lnc3 | GCAGCGTGGGCTCGCGTGCCGGGGTTTTTT | TCATGGGGTGGTAA |  | 14 | 116 | 52.89 | False | 0.00 | 0.00 |
| pefA | VFG000435(gi:17233483)_fwd |  | CCGTTCAGGCTAACCAG |  | 17 | 105 | 60.29 | False | 41.89 | -240.62 |
|  | VFG000435(gi:17233483)_rev |  | AAACAGTGGCCGTTTTC |  | 17 | 105 | 58.94 | False | 38.63 | -113.56 |
|  | VFG000435(gi:17233483)_lnc3 | GCAGCGTGGGCTCGCGTGCCGGGGTTTTTT | TTGTTGACTTCGCC |  | 14 | 105 | 53.88 | False | 0.00 | 0.00 |
|  | VFG000435(gi:17233483)_detection |  | ATGAAACCGGTTGAC | TTT | 15 | 105 | 54.55 | False | 0.00 | 0.00 |
| algG | VFG000127(gi:15598741)_fwd |  | AAGTCAACGACAGCTTC |  | 17 | 125 | 57.61 | False | 38.70 | -113.75 |
|  | VFG000127(gi:15598741)_rev |  | GTCGGAATGGTTGCGRTAG |  | 19 | 125 | 63.33 | True | 58.68 | -1927.66 |
|  | VFG000127(gi:15598741)_detection |  | AAGCTTTCCGGGAT | TTT | 14 | 125 | 54.52 | False | 0.00 | 0.00 |
|  | VFG000127(gi:15598741)_lnc3 | GCAGCGTGGGCTCGCGTGCCGGGGTTTTTT | GCAGCTACGAGAAC |  | 14 | 125 | 53.92 | False | 0.00 | 0.00 |
| chpB | VFG001232(gi:15595611)_fwd |  | TTCGAGCATGCCGGACA |  | 17 | 89 | 64.93 | False | 0.00 | 0.00 |
|  | VFG001232(gi:15595611)_rev |  | GATGCTTGACCAATGCC |  | 17 | 89 | 59.28 | False | 0.00 | 0.00 |
|  | VFG001232(gi:15595611)_lnc3 | GCAGCGTGGGCTCGCGTGCCGGGGTTTTTT | GAAGCCGGTTACAG |  | 14 | 89 | 53.72 | False | 0.00 | 0.00 |
|  | VFG001232(gi:15595611)_detection |  | CAGTTTCAATGCCA | TTT | 14 | 89 | 50.67 | False | 0.00 | 0.00 |
| fimU | VFG001216(gi:15599746)_fwd |  | TCGTTCCAACTCGACCG |  | 17 | 117 | 62.35 | False | 0.00 | 0.00 |
|  | VFG001216(gi:15599746)_rev |  | AGTTCGTTGCGTTCTGTCA |  | 19 | 117 | 63.63 | False | 0.00 | 0.00 |
|  | VFG001216(gi:15599746)_lnc3 | GCAGCGTGGGCTCGCGTGCCGGGGTTTTTT | TGATCGAGTTGCTG |  | 14 | 117 | 52.76 | False | 0.00 | 0.00 |
|  | VFG001216(gi:15599746)_detection |  | ATCATCGTCGTCCT | TTT | 14 | 117 | 53.51 | False | 0.00 | 0.00 |
| lasA | VFG000145(gi:15597068)_fwd |  | GCACCAGCAACTACGACA |  | 18 | 76 | 63.58 | False | 43.81 | -429.53 |
|  | VFG000145(gi:15597068)_rev |  | AAAGCGCAATGGGTGGT |  | 17 | 76 | 63.38 | False | 42.24 | -317.32 |
|  | VFG000145(gi:15597068)_lnc3 | GCAGCGTGGGCTCGCGTGCCGGGGTTTTTT | TCGCCGCTACTATT |  | 14 | 76 | 54.08 | False | 0.00 | 0.00 |
|  | VFG000145(gi:15597068)_detection |  | TCTACAACCAGAGC | TTT | 14 | 76 | 51.12 | False | 0.00 | 0.00 |
| fimV | VFG001230(gi:15598311)_fwd |  | TCTCGACTTCGGCCTGG |  | 17 | 117 | 64.24 | False | 42.27 | -372.39 |
|  | VFG001230(gi:15598311)_rev |  | TCGTCCGAAAGCTTGTCTTC |  | 20 | 117 | 64.22 | False | 37.54 | -31.41 |
|  | VFG001230(gi:15598311)_detection |  | CTGGGCGATTTCTC | TTT | 14 | 117 | 53.79 | False | 0.00 | 0.00 |
|  | VFG001230(gi:15598311)_lnc3 | GCAGCGTGGGCTCGCGTGCCGGGGTTTTTT | CCAAGCATGACGAC |  | 14 | 117 | 54.68 | False | 0.00 | 0.00 |
| pilA | VFG005299(gi:25011520)_fwd |  | ACCAAAAATTTTCTAAAATATTGA |  | 24 | 116 | 56.18 | False | 42.74 | -465.44 |
|  | VFG005299(gi:25011520)_rev |  | TAACTTTCCTTTAGCACCATTTTCC |  | 25 | 116 | 64.31 | False | 0.00 | 0.00 |
|  | VFG005299(gi:25011520)_detection |  | GCTTAATACCAATGTTTTA | TTT | 19 | 116 | 52.84 | False | 0.00 | 0.00 |
|  | VFG005299(gi:25011520)_lnc3 | GCAGCGTGGGCTCGCGTGCCGGGGTTTTTT | TTGTCGCAAATACC |  | 14 | 116 | 50.49 | False | 0.00 | 0.00 |
| seb | VFG001802(gi:153000)_fwd |  | TTCACTGGTTTGATGGAAAATATG |  | 24 | 179 | 62.14 | False | 0.00 | 0.00 |
|  | VFG001802(gi:153000)_rev |  | TTATCAGCTAAATCTTTGTTTTTAA |  | 25 | 179 | 58.19 | False | 0.00 | 0.00 |
|  | VFG001802(gi:153000)_lnc3 | GCAGCGTGGGCTCGCGTGCCGGGGTTTTTT | TAGATCAATTTCTATACT |  | 18 | 179 | 47.69 | False | 0.00 | 0.00 |
|  | VFG001802(gi:153000)_detection |  | TTGACTTAATATATTCTAT | TTT | 19 | 179 | 46.61 | False | 0.00 | 0.00 |
| algQ | VFG000118(gi:15600448)_fwd |  | CCAAGCAGATCTTCCCCC |  | 18 | 78 | 63.12 | False | 35.99 | 58.44 |
|  | VFG000118(gi:15600448)_rev |  | AATCGCCGTTGTCGCAG |  | 17 | 78 | 64.19 | False | 37.49 | -34.42 |
|  | VFG000118(gi:15600448)_lnc3 | GCAGCGTGGGCTCGCGTGCCGGGGTTTTTT | CCATCACCGAATCC |  | 14 | 78 | 53.32 | False | 0.00 | 0.00 |
|  | VFG000118(gi:15600448)_detection |  | GCGCTGAACTTCAA | TTT | 14 | 78 | 54.44 | False | 0.00 | 0.00 |
| xcpY | VFG000175(gi:15598292)_fwd |  | GTRTTGCAATGGGGCTT |  | 17 | 149 | 59.81 | False | 36.18 | 0.00 |
|  | VFG000175(gi:15598292)_rev |  | AGGTGCTGGTCGAACTG |  | 17 | 149 | 62.31 | False | 0.00 | 0.00 |
|  | VFG000175(gi:15598292)_detection |  | CGCAAGCTGATCAA | TTT | 14 | 149 | 54.11 | False | 0.00 | 0.00 |
|  | VFG000175(gi:15598292)_lnc3 | GCAGCGTGGGCTCGCGTGCCGGGGTTTTTT | GTTTCCCGAGGAC |  | 13 | 149 | 51.80 | False | 0.00 | 0.00 |
| pilY2 | VFG001211(gi:15599751)_fwd |  | GTGGTGTCCTACTCTGG |  | 17 | 98 | 58.75 | False | 0.00 | 0.00 |
|  | VFG001211(gi:15599751)_rev |  | ATCATCTCGGCCAGCTC |  | 17 | 98 | 61.90 | False | 0.00 | 0.00 |
|  | VFG001211(gi:15599751)_lnc3 | GCAGCGTGGGCTCGCGTGCCGGGGTTTTTT | CACCGATATCTACATTC |  | 17 | 98 | 52.64 | False | 0.00 | 0.00 |
|  | VFG001211(gi:15599751)_detection |  | ACAAACAGATGAGCG | TTT | 15 | 98 | 54.72 | False | 0.00 | 0.00 |
| waaA | VFG000141(gi:15600181)_fwd |  | GCCGACATCGCTTTCGT |  | 17 | 167 | 63.87 | False | 40.31 | -216.19 |
|  | VFG000141(gi:15600181)_rev |  | GCATCCGTCACCTCGAG |  | 17 | 167 | 62.92 | False | 0.00 | 0.00 |
|  | VFG000141(gi:15600181)_lnc3 | GCAGCGTGGGCTCGCGTGCCGGGGTTTTTT | TCCGCACCTGTTC |  | 13 | 167 | 54.93 | False | 0.00 | 0.00 |
|  | VFG000141(gi:15600181)_detection |  | AACTTCCTCGACA | TTT | 13 | 167 | 48.81 | False | 0.00 | 0.00 |
| sea | VFG001325(gi:21283618)_fwd |  | GATTAATCGTGTTTCATACTTCT |  | 23 | 134 | 58.79 | False | 0.00 | 0.00 |
|  | VFG001325(gi:21283618)_rev |  | AATATGCATGTTTTCAGAGT |  | 20 | 134 | 56.75 | False | 0.00 | 0.00 |
|  | VFG001325(gi:21283618)_lnc3 | GCAGCGTGGGCTCGCGTGCCGGGGTTTTTT | GGTTAATTACGATTTAT |  | 17 | 134 | 47.15 | False | 0.00 | 0.00 |
|  | VFG001325(gi:21283618)_detection |  | TTGGTGCTCAAGGA | TTT | 14 | 134 | 54.32 | False | 0.00 | 0.00 |
| hylB | VFG001334(gi:22537355)_fwd |  | CACTATTGATATTGAACGC |  | 19 | 105 | 55.09 | False | 0.00 | 0.00 |
|  | VFG001334(gi:22537355)_rev |  | TGGCTTATAGTGATAAAAGGAT |  | 22 | 105 | 58.62 | False | 0.00 | 0.00 |
|  | VFG001334(gi:22537355)_detection |  | TTCTAAAAATACCTCAAT | TTT | 18 | 105 | 49.66 | False | 0.00 | 0.00 |
|  | VFG001334(gi:22537355)_lnc3 | GCAGCGTGGGCTCGCGTGCCGGGGTTTTTT | AGCATTAATCGTAC |  | 14 | 105 | 45.83 | False | 0.00 | 0.00 |
| xcpR | VFG000182(gi:15598299)_fwd |  | TGTTCGACGACCACATGC |  | 18 | 124 | 63.93 | False | 0.00 | 0.00 |
|  | VFG000182(gi:15598299)_rev |  | ACGCCTTCCAGCACCTT |  | 17 | 124 | 64.85 | False | 0.00 | 0.00 |
|  | VFG000182(gi:15598299)_detection |  | CGAGCAGGAAATGA | TTT | 14 | 124 | 52.69 | False | 0.00 | 0.00 |
|  | VFG000182(gi:15598299)_lnc3 | GCAGCGTGGGCTCGCGTGCCGGGGTTTTTT | CCACAACGAATCCTC |  | 15 | 124 | 54.83 | False | 0.00 | 0.00 |
| fimT | VFG001215(gi:15599745)_fwd |  | TTGCAAAGAAGGAAAGGCT |  | 19 | 134 | 61.34 | False | 0.00 | 0.00 |
|  | VFG001215(gi:15599745)_rev |  | GTTGAGGATCAGTTGCC |  | 17 | 134 | 58.10 | True | 56.82 | -1021.07 |
|  | VFG001215(gi:15599745)_lnc3 | GCAGCGTGGGCTCGCGTGCCGGGGTTTTTT | ACATTCACAACGGG |  | 14 | 134 | 53.38 | False | 0.00 | 0.00 |
|  | VFG001215(gi:15599745)_detection |  | CGGTTTCTGCTCTG | TTT | 14 | 134 | 54.16 | False | 0.00 | 0.00 |
| hlyC | VFG000842(gi:75994493)_fwd |  | ATGGGGAAGGTTGCATG |  | 17 | 146 | 60.20 | False | 0.00 | 0.00 |
|  | VFG000842(gi:75994493)_rev |  | CTACAAAAAGCTACAGGAA |  | 19 | 146 | 55.86 | False | 0.00 | 0.00 |
|  | VFG000842(gi:75994493)_detection |  | AATGTCATACCTGC | TTT | 14 | 146 | 49.02 | False | 0.00 | 0.00 |
|  | VFG000842(gi:75994493)_lnc3 | GCAGCGTGGGCTCGCGTGCCGGGGTTTTTT | TCTGTTTTTGCTATT |  | 15 | 146 | 47.11 | False | 0.00 | 0.00 |
| algD | VFG000122(gi:15598736)_fwd |  | CTTTGGTTTGGGCTATG |  | 17 | 117 | 55.87 | False | 0.00 | 0.00 |
|  | VFG000122(gi:15598736)_rev |  | GACTTGCCCTGGTTGATC |  | 18 | 117 | 61.31 | False | 0.00 | 0.00 |
|  | VFG000122(gi:15598736)_detection |  | TCCAGCACCAAGAT | TTT | 14 | 117 | 53.96 | False | 0.00 | 0.00 |
|  | VFG000122(gi:15598736)_lnc3 | GCAGCGTGGGCTCGCGTGCCGGGGTTTTTT | TTGGTGTGGATGTC |  | 14 | 117 | 52.35 | False | 0.00 | 0.00 |

### VF-6

Supplementary Table S18: Primer and probe sequences and additional information of VF-6.

| Gene | Primer/Probe | Hybridization Sequence | Sequence | Spacer | Length | Product Size | Tm | Hairpin | Hairpin Tm | Hairpin delta G |
| --- | --- | --- | --- | --- | --- | --- | --- | --- | --- | --- |
| tsst-1 | VFG001809(gi:15927587)_fwd |  | CGGATCCACATATCAAAGTGATTTA |  | 25 | 106 | 63.52 | False | 43.25 | -521.08 |
|  | VFG001809(gi:15927587)_rev |  | TTAATTAATTTCTGCTTCTATAGTT |  | 25 | 106 | 57.04 | False | 0.00 | 0.00 |
|  | VFG001809(gi:15927587)_detection |  | CACCTATAAATATTGATGA | TTT | 19 | 106 | 50.94 | False | 0.00 | 0.00 |
|  | VFG001809(gi:15927587)_lnc3 | GCAGCGTGGGCTCGCGTGCCGGGGTTTTTT | TGAATACAATACTGAAAAAC |  | 20 | 106 | 53.72 | False | 0.00 | 0.00 |
| mf3 | VFG000971(gi:15675346)_fwd |  | TTTAGCTGAATTGGATTACC |  | 20 | 93 | 56.84 | False | 0.00 | 0.00 |
|  | VFG000971(gi:15675346)_rev |  | GCTTCTTCAAACTCTTCG |  | 18 | 93 | 56.90 | False | 0.00 | 0.00 |
|  | VFG000971(gi:15675346)_detection |  | TCAAAACGAAGAAG | TTT | 14 | 93 | 46.82 | False | 0.00 | 0.00 |
|  | VFG000971(gi:15675346)_lnc3 | GCAGCGTGGGCTCGCGTGCCGGGGTTTTTT | ATGCTAGATTCAAC |  | 14 | 93 | 45.50 | False | 0.00 | 0.00 |
| mf2 | VFG000970(gi:15674770)_fwd |  | TTCAGGAGCAAATGACAGCA |  | 20 | 149 | 63.90 | False | 0.00 | 0.00 |
|  | VFG000970(gi:15674770)_rev |  | ATTCAATTTGTCGAGGCACTAC |  | 22 | 149 | 63.16 | True | 46.70 | -263.92 |
|  | VFG000970(gi:15674770)_detection |  | GGCTAGATTATAAAGTCAC | TTT | 19 | 149 | 54.44 | False | 0.00 | 0.00 |
|  | VFG000970(gi:15674770)_lnc3 | GCAGCGTGGGCTCGCGTGCCGGGGTTTTTT | CTTCACCCTGACTTTT |  | 16 | 149 | 54.58 | False | 0.00 | 0.00 |
| pilB | VFG005297(gi:25011519)_fwd |  | ACAAAGGTGTTGTCTCAG |  | 18 | 124 | 57.92 | False | 38.78 | -78.21 |
|  | VFG005297(gi:25011519)_rev |  | AACGTCAAACCTTTAGTC |  | 18 | 124 | 55.62 | False | 0.00 | 0.00 |
|  | VFG005297(gi:25011519)_detection |  | AAACTGGTTTGGAC | TTT | 14 | 124 | 49.95 | False | 0.00 | 0.00 |
|  | VFG005297(gi:25011519)_lnc3 | GCAGCGTGGGCTCGCGTGCCGGGGTTTTTT | AAGGCTCAGACTATAAG |  | 17 | 124 | 53.63 | False | 0.00 | 0.00 |
| nleD | VFG002123(gi:15800523)_fwd |  | AAATCAGAAACAGTGGTAATAC |  | 22 | 149 | 58.26 | False | 0.00 | 0.00 |
|  | VFG002123(gi:15800523)_rev |  | CTAATCCCCTCCCCACAG |  | 18 | 149 | 61.75 | False | 0.00 | 0.00 |
|  | VFG002123(gi:15800523)_lnc3 | GCAGCGTGGGCTCGCGTGCCGGGGTTTTTT | CACATAGAGATATAGA |  | 16 | 149 | 45.14 | False | 0.00 | 0.00 |
|  | VFG002123(gi:15800523)_detection |  | TGCTGAGAACCATC | TTT | 14 | 149 | 52.07 | False | 0.00 | 0.00 |
| cif | VFG042218(gi:37547384)_fwd |  | AAATATCGACTCTAACATCAAACAG |  | 25 | 151 | 61.68 | False | 0.00 | 0.00 |
|  | VFG042218(gi:37547384)_rev |  | ATCCTTGTCCAGAAATAGTTTCAT |  | 24 | 151 | 62.43 | True | 45.63 | -687.25 |
|  | VFG042218(gi:37547384)_detection |  | ATAGGGCGAGAGAA | TTT | 14 | 151 | 52.39 | False | 0.00 | 0.00 |
|  | VFG042218(gi:37547384)_lnc3 | GCAGCGTGGGCTCGCGTGCCGGGGTTTTTT | GGAGGTTGAGGTTATT |  | 16 | 151 | 53.49 | False | 0.00 | 0.00 |
| fbsB | VFG005215(gi:25010905)_fwd |  | TCCCCAATATTCCATATAAAAAT |  | 23 | 126 | 57.59 | False | 0.00 | 0.00 |
|  | VFG005215(gi:25010905)_rev |  | GTAAAACTTCATTCTTAAGCTTATT |  | 25 | 126 | 58.57 | False | 0.00 | 0.00 |
|  | VFG005215(gi:25010905)_lnc3 | GCAGCGTGGGCTCGCGTGCCGGGGTTTTTT | GTTTTGAAGATGATG |  | 15 | 126 | 46.62 | False | 0.00 | 0.00 |
|  | VFG005215(gi:25010905)_detection |  | AAGAAGAGTTTGGAAATG | TTT | 18 | 126 | 54.41 | False | 0.00 | 0.00 |
| acm | VFG043518(gi:29134931)_fwd |  | TTGATTTTGATCCACTAAGTTCAA |  | 24 | 128 | 61.23 | False | 0.00 | 0.00 |
|  | VFG043518(gi:29134931)_rev |  | GTTCCAAATTTTAGTTGCTG |  | 20 | 128 | 57.14 | False | 0.00 | 0.00 |
|  | VFG043518(gi:29134931)_detection |  | TTATAACAAGAAAAAGGTTA | TTT | 20 | 128 | 52.13 | False | 0.00 | 0.00 |
|  | VFG043518(gi:29134931)_lnc3 | GCAGCGTGGGCTCGCGTGCCGGGGTTTTTT | GAGTCAGTTTACCTAT |  | 16 | 128 | 49.66 | False | 0.00 | 0.00 |
| emm | VFG000950(gi:21911263)_fwd |  | AAGAAAGTTACAGAAGCAACTG |  | 22 | 163 | 60.99 | False | 44.24 | -698.44 |
|  | VFG000950(gi:21911263)_rev |  | CTTCTTTAGCATTTTGTTTAGT |  | 22 | 163 | 57.07 | False | 0.00 | 0.00 |
|  | VFG000950(gi:21911263)_lnc3 | GCAGCGTGGGCTCGCGTGCCGGGGTTTTTT | AAAAAGTTGAAAGTAC |  | 16 | 163 | 47.20 | False | 0.00 | 0.00 |
|  | VFG000950(gi:21911263)_detection |  | TATGCAAGATTTAACTGAA | TTT | 19 | 163 | 54.01 | False | 0.00 | 0.00 |
| emm | VFG000968(gi:15675799)_fwd |  | TAAAAATAACACGAATAGACACTA |  | 24 | 121 | 58.20 | False | 0.00 | 0.00 |
|  | VFG000968(gi:15675799)_rev |  | GTTAGCCTTAACCTCTGT |  | 18 | 121 | 56.67 | False | 38.75 | -164.51 |
|  | VFG000968(gi:15675799)_lnc3 | GCAGCGTGGGCTCGCGTGCCGGGGTTTTTT | CTTCAGTAGCGGTA |  | 14 | 121 | 50.91 | False | 0.00 | 0.00 |
|  | VFG000968(gi:15675799)_detection |  | GCTTTGACTGTTTT | TTT | 14 | 121 | 47.96 | False | 0.00 | 0.00 |
| pilB | VFG000112(gi:15599722)_fwd |  | GCAAGAACGGTTACAAG |  | 17 | 81 | 56.32 | False | 0.00 | 0.00 |
|  | VFG000112(gi:15599722)_rev |  | CCTCCATGATAATCCGC |  | 17 | 81 | 57.33 | False | 0.00 | 0.00 |
|  | VFG000112(gi:15599722)_lnc3 | GCAGCGTGGGCTCGCGTGCCGGGGTTTTTT | CGGTATTTATGAAGTA |  | 16 | 81 | 47.60 | False | 0.00 | 0.00 |
|  | VFG000112(gi:15599722)_detection |  | GTTAAAAACACCCCG | TTT | 15 | 81 | 53.02 | False | 0.00 | 0.00 |
| plcB | VFG000069(gi:16802251)_fwd |  | ATCATATGCGAGCTAATTTAATGAA |  | 25 | 162 | 61.58 | False | 0.00 | 0.00 |
|  | VFG000069(gi:16802251)_rev |  | CAAAACCCGGCAAATAAGTA |  | 20 | 162 | 60.23 | False | 0.00 | 0.00 |
|  | VFG000069(gi:16802251)_lnc3 | GCAGCGTGGGCTCGCGTGCCGGGGTTTTTT | CGGATCATAAAAATC |  | 15 | 162 | 46.05 | False | 0.00 | 0.00 |
|  | VFG000069(gi:16802251)_detection |  | CATATTATGATACTAGTAC | TTT | 19 | 162 | 47.98 | False | 0.00 | 0.00 |
| eltA | VFG002037(gi:148028)_fwd |  | AACAGGAGGTTTCTGCGTTA |  | 20 | 108 | 63.24 | False | 41.56 | -219.00 |
|  | VFG002037(gi:148028)_rev |  | TATATTCCCTGTTACGATGTAAT |  | 23 | 108 | 58.81 | False | 0.00 | 0.00 |
|  | VFG002037(gi:148028)_lnc3 | GCAGCGTGGGCTCGCGTGCCGGGGTTTTTT | AATACCATATTCTCAGAT |  | 18 | 108 | 50.93 | False | 0.00 | 0.00 |
|  | VFG002037(gi:148028)_detection |  | ATATGGATGGTATCG | TTT | 15 | 108 | 48.63 | False | 0.00 | 0.00 |
| hylP | VFG000965(gi:15674762)_fwd |  | GSCACAATCGATTTAGTGA |  | 19 | 122 | 59.03 | False | 37.64 | -48.05 |
|  | VFG000965(gi:15674762)_rev |  | TTTTTGTTGTAGTTTCTGAGCG |  | 22 | 122 | 61.83 | False | 0.00 | 0.00 |
|  | VFG000965(gi:15674762)_detection |  | AACAAGCCAAATAT | TTT | 14 | 122 | 45.34 | False | 0.00 | 0.00 |
|  | VFG000965(gi:15674762)_lnc3 | GCAGCGTGGGCTCGCGTGCCGGGGTTTTTT | ATTACAATCTACTGACT |  | 17 | 122 | 50.00 | False | 0.00 | 0.00 |
| cpsH | VFG001345(gi:22537326)_fwd |  | TCTAAAAAAAGCTTTCTCTATAATA |  | 25 | 150 | 56.74 | False | 0.00 | 0.00 |
|  | VFG001345(gi:22537326)_rev |  | CCAAAACCTAAGTCACTTCTCA |  | 22 | 150 | 62.14 | False | 0.00 | 0.00 |
|  | VFG001345(gi:22537326)_detection |  | TTTGTGAAATTAGATT | TTT | 16 | 150 | 45.38 | False | 0.00 | 0.00 |
|  | VFG001345(gi:22537326)_lnc3 | GCAGCGTGGGCTCGCGTGCCGGGGTTTTTT | GTTCTATTAAATCAAATT |  | 18 | 150 | 46.53 | False | 0.00 | 0.00 |
| cpsM | VFG001344(gi:22537325)_fwd |  | TGTCTGACTAGTGTTACC |  | 18 | 106 | 55.97 | False | 0.00 | 0.00 |
|  | VFG001344(gi:22537325)_rev |  | AATCAATTATGAGCCTTACT |  | 20 | 106 | 55.50 | False | 43.78 | -430.29 |
|  | VFG001344(gi:22537325)_lnc3 | GCAGCGTGGGCTCGCGTGCCGGGGTTTTTT | TATACATCATTACGAAGGA |  | 19 | 106 | 54.54 | False | 0.00 | 0.00 |
|  | VFG001344(gi:22537325)_detection |  | AGTTGGAAAAGTTC | TTT | 14 | 106 | 46.42 | False | 0.00 | 0.00 |
| cps4L | VFG001376(gi:15900289)_fwd |  | TAAACGTGTGATTCAGCAT |  | 19 | 124 | 58.56 | False | 0.00 | 0.00 |
|  | VFG001376(gi:15900289)_rev |  | TTGACTAGTAAAGAAACTACT |  | 21 | 124 | 55.24 | False | 36.94 | 6.17 |
|  | VFG001376(gi:15900289)_detection |  | TCGGGAACTTTACC | TTT | 14 | 124 | 51.52 | False | 0.00 | 0.00 |
|  | VFG001376(gi:15900289)_lnc3 | GCAGCGTGGGCTCGCGTGCCGGGGTTTTTT | GTTGTAGTATCTGAT |  | 15 | 124 | 45.44 | False | 0.00 | 0.00 |
| bont/a | VFG002297(gi:123229205)_fwd |  | ATTATGGTGAAATAATCTGGACT |  | 23 | 109 | 59.53 | False | 35.12 | 154.02 |
|  | VFG002297(gi:123229205)_rev |  | ATCCATCTGTTTATATAATCTGAT |  | 24 | 109 | 57.33 | False | 0.00 | 0.00 |
|  | VFG002297(gi:123229205)_lnc3 | GCAGCGTGGGCTCGCGTGCCGGGGTTTTTT | ACAAAGAGTAGTTTT |  | 15 | 109 | 46.05 | False | 0.00 | 0.00 |
|  | VFG002297(gi:123229205)_detection |  | TAAATACAGTCAAATG | TTT | 16 | 109 | 45.83 | False | 0.00 | 0.00 |
| espD | VFG000743(gi:2865303)_fwd |  | GCWGTGGCAAATGGTTTAAC |  | 20 | 131 | 62.09 | False | 42.98 | -370.70 |
|  | VFG000743(gi:2865303)_rev |  | TCACTTTGTCGAGTTAAATTTTC |  | 23 | 131 | 59.93 | False | 0.00 | 0.00 |
|  | VFG000743(gi:2865303)_detection |  | AACTGTGCTGAATA | TTT | 14 | 131 | 47.72 | False | 0.00 | 0.00 |
|  | VFG000743(gi:2865303)_lnc3 | GCAGCGTGGGCTCGCGTGCCGGGGTTTTTT | CGCTCTATTGGTAC |  | 14 | 131 | 49.58 | False | 0.00 | 0.00 |
| siaB/synB | VFG000253(gi:15676005)_fwd |  | CCGATACAGCCAGCTCTATTTC |  | 22 | 156 | 64.79 | False | 0.00 | 0.00 |
|  | VFG000253(gi:15676005)_rev |  | ATCCTTTTATTTTCTCATCAAAT |  | 23 | 156 | 56.65 | False | 0.00 | 0.00 |
|  | VFG000253(gi:15676005)_lnc3 | GCAGCGTGGGCTCGCGTGCCGGGGTTTTTT | CACAGTAACCCTATT |  | 15 | 156 | 48.97 | False | 0.00 | 0.00 |
|  | VFG000253(gi:15676005)_detection |  | ACAACCAACCAGTC | TTT | 14 | 156 | 52.67 | False | 0.00 | 0.00 |
| hylP | VFG000966(gi:15675005)_fwd |  | TCGTCTTACTGGAGGGTG |  | 18 | 143 | 61.24 | False | 0.00 | 0.00 |
|  | VFG000966(gi:15675005)_rev |  | AGTTTTACCTTGGAGACCAGT |  | 21 | 143 | 62.79 | False | 38.68 | -94.38 |
|  | VFG000966(gi:15675005)_lnc3 | GCAGCGTGGGCTCGCGTGCCGGGGTTTTTT | GCTAAGTTTGGCGAT |  | 15 | 143 | 54.78 | False | 0.00 | 0.00 |
|  | VFG000966(gi:15675005)_detection |  | GGTCAAAACACTTTTAGT | TTT | 18 | 143 | 54.69 | False | 0.00 | 0.00 |
| llsG | VFG045332(gi:588561365)_fwd |  | ATATAACGCATTACTTTGAGG |  | 21 | 95 | 57.50 | False | 0.00 | 0.00 |
|  | VFG045332(gi:588561365)_rev |  | TTCTGATATTGTTCCCCAAAA |  | 21 | 95 | 59.21 | False | 40.70 | -239.50 |
|  | VFG045332(gi:588561365)_lnc3 | GCAGCGTGGGCTCGCGTGCCGGGGTTTTTT | GAAAACTGGGCAAC |  | 14 | 95 | 52.15 | False | 0.00 | 0.00 |
|  | VFG045332(gi:588561365)_detection |  | GAAAGTTTTGATTCTTCA | TTT | 18 | 95 | 52.35 | False | 0.00 | 0.00 |
| llsH | VFG045333(gi:588561366)_fwd |  | TGCCAGTAGGAATGTGTT |  | 18 | 142 | 59.36 | False | 39.89 | -133.10 |
|  | VFG045333(gi:588561366)_rev |  | CAAACATTAAATAAATCATTCATTA |  | 25 | 142 | 55.37 | False | 0.00 | 0.00 |
|  | VFG045333(gi:588561366)_lnc3 | GCAGCGTGGGCTCGCGTGCCGGGGTTTTTT | ATTTTTACAGGAACATT |  | 17 | 142 | 49.85 | False | 0.00 | 0.00 |
|  | VFG045333(gi:588561366)_detection |  | TTCATCTTTTTCGGA | TTT | 15 | 142 | 49.73 | False | 0.00 | 0.00 |
| siaC/synC | VFG000252(gi:15676004)_fwd |  | TGGTTGATGCTGCCTATA |  | 18 | 103 | 58.86 | False | 0.00 | 0.00 |
|  | VFG000252(gi:15676004)_rev |  | TTGCCTGGAATGACTTGTT |  | 19 | 103 | 61.03 | False | 0.00 | 0.00 |
|  | VFG000252(gi:15676004)_detection |  | ACACATCGTTGAAG | TTT | 14 | 103 | 50.00 | False | 0.00 | 0.00 |
|  | VFG000252(gi:15676004)_lnc3 | GCAGCGTGGGCTCGCGTGCCGGGGTTTTTT | TTGTTAAACATCAAAC |  | 16 | 103 | 47.71 | False | 0.00 | 0.00 |
| spej | VFG000980(gi:15674561)_fwd |  | GATTATACGAATTGTAATATTGAT |  | 24 | 119 | 55.13 | False | 40.70 | -239.50 |
|  | VFG000980(gi:15674561)_rev |  | TTTGTTGTAATATAGCTCTCGA |  | 22 | 119 | 58.92 | False | 0.00 | 0.00 |
|  | VFG000980(gi:15674561)_lnc3 | GCAGCGTGGGCTCGCGTGCCGGGGTTTTTT | TTTCCAGTTATAAAAAG |  | 17 | 119 | 47.49 | False | 0.00 | 0.00 |
|  | VFG000980(gi:15674561)_detection |  | AAAAATTTTTCAGTTGATTC | TTT | 20 | 119 | 53.29 | False | 0.00 | 0.00 |
| spea | VFG000951(gi:21910837)_fwd |  | TATTTGCTCAACAAGACCC |  | 19 | 91 | 58.86 | False | 44.36 | -477.47 |
|  | VFG000951(gi:21910837)_rev |  | TCACCCTCATAAAGAAAATAT |  | 21 | 91 | 55.97 | False | 0.00 | 0.00 |
|  | VFG000951(gi:21910837)_lnc3 | GCAGCGTGGGCTCGCGTGCCGGGGTTTTTT | TCACAGATCTAGTTT |  | 15 | 91 | 47.48 | False | 0.00 | 0.00 |
|  | VFG000951(gi:21910837)_detection |  | AGTTAAAAACCTTCAA | TTT | 16 | 91 | 48.35 | False | 0.00 | 0.00 |
| llsX | VFG045334(gi:588561367)_fwd |  | ATTTTCGGACTAACTATTTTTGAT |  | 24 | 170 | 59.24 | False | 0.00 | 0.00 |
|  | VFG045334(gi:588561367)_rev |  | CATTTTGCTGTTTCCTTTCTATGT |  | 24 | 170 | 62.62 | False | 0.00 | 0.00 |
|  | VFG045334(gi:588561367)_lnc3 | GCAGCGTGGGCTCGCGTGCCGGGGTTTTTT | CTAGCATGATGTTTATAGG |  | 19 | 170 | 54.50 | False | 0.00 | 0.00 |
|  | VFG045334(gi:588561367)_detection |  | GGTTATTATTTCTATGATAC | TTT | 20 | 170 | 50.30 | False | 0.00 | 0.00 |
| cpsJ | VFG001341(gi:22537322)_fwd |  | CAATCTTTCCTAAACGAGT |  | 19 | 104 | 55.96 | False | 0.00 | 0.00 |
|  | VFG001341(gi:22537322)_rev |  | ATATCCCCGGAATTATCTGTAG |  | 22 | 104 | 60.61 | False | 0.00 | 0.00 |
|  | VFG001341(gi:22537322)_lnc3 | GCAGCGTGGGCTCGCGTGCCGGGGTTTTTT | TCAAACTTATTCAAATTTAG |  | 20 | 104 | 51.48 | False | 0.00 | 0.00 |
|  | VFG001341(gi:22537322)_detection |  | AAATTATTCTAGTTAACGA | TTT | 19 | 104 | 50.74 | False | 0.00 | 0.00 |
| sic | VFG000972(gi:15675798)_fwd |  | AACACTACTATTTACATCCC |  | 20 | 94 | 55.56 | False | 0.00 | 0.00 |
|  | VFG000972(gi:15675798)_rev |  | CCAGTCAAAATTGCGTG |  | 17 | 94 | 57.40 | False | 0.00 | 0.00 |
|  | VFG000972(gi:15675798)_lnc3 | GCAGCGTGGGCTCGCGTGCCGGGGTTTTTT | GCTACACAACCAGTT |  | 15 | 94 | 54.05 | False | 0.00 | 0.00 |
|  | VFG000972(gi:15675798)_detection |  | TCAGCCGAAACGTA | TTT | 14 | 94 | 54.74 | False | 0.00 | 0.00 |
| gbs0632 | VFG005289(gi:25010692)_fwd |  | GTGTAAACGGAGATCCT |  | 17 | 149 | 55.84 | False | 0.00 | 0.00 |
|  | VFG005289(gi:25010692)_rev |  | TTCCTCAACAATTGTTTTAAA |  | 21 | 149 | 56.05 | False | 40.25 | -202.47 |
|  | VFG005289(gi:25010692)_detection |  | AGTAAAACAGAAAATTA | TTT | 17 | 149 | 46.47 | False | 0.00 | 0.00 |
|  | VFG005289(gi:25010692)_lnc3 | GCAGCGTGGGCTCGCGTGCCGGGGTTTTTT | TTTATGCAATCAATATCA |  | 18 | 149 | 50.70 | False | 0.00 | 0.00 |
| cna | VFG001291(gi:21284341)_fwd |  | GTGATGTTTCGGGATTTGC |  | 19 | 113 | 60.99 | False | 0.00 | 0.00 |
|  | VFG001291(gi:21284341)_rev |  | AACATTCGTTGATTTATTCCCAG |  | 23 | 113 | 61.71 | False | 37.44 | -25.96 |
|  | VFG001291(gi:21284341)_detection |  | ATTTAACGCAAACAAA | TTT | 16 | 113 | 50.78 | False | 0.00 | 0.00 |
|  | VFG001291(gi:21284341)_lnc3 | GCAGCGTGGGCTCGCGTGCCGGGGTTTTTT | AAGTACAAGGAAGAA |  | 15 | 113 | 48.14 | False | 0.00 | 0.00 |
| exoU | VFG000149(gi:2429143)_fwd |  | CCATCGCCAGCAGATTG |  | 17 | 86 | 61.53 | False | 0.00 | 0.00 |
|  | VFG000149(gi:2429143)_rev |  | CGTCTTAATCTGGGGAA |  | 17 | 86 | 55.81 | False | 0.00 | 0.00 |
|  | VFG000149(gi:2429143)_lnc3 | GCAGCGTGGGCTCGCGTGCCGGGGTTTTTT | GAGTCACCTTTGGC |  | 14 | 86 | 53.95 | False | 0.00 | 0.00 |
|  | VFG000149(gi:2429143)_detection |  | GATCTAGATCGGTT | TTT | 14 | 86 | 46.93 | False | 0.00 | 0.00 |
| actA | VFG000068(gi:16802250)_fwd |  | ATGAATGGGAAGAAGAAAAAACAG |  | 24 | 123 | 62.33 | False | 0.00 | 0.00 |
|  | VFG000068(gi:16802250)_rev |  | TCACTTTATTCGATTTTTCTAGTT |  | 24 | 123 | 58.94 | False | 0.00 | 0.00 |
|  | VFG000068(gi:16802250)_detection |  | TACGAAACTGCACG | TTT | 14 | 123 | 53.71 | False | 0.00 | 0.00 |
|  | VFG000068(gi:16802250)_lnc3 | GCAGCGTGGGCTCGCGTGCCGGGGTTTTTT | ATACGGGACCAAGA |  | 14 | 123 | 52.68 | False | 0.00 | 0.00 |
| ctrB | VFG000256(gi:15676008)_fwd |  | GAGCCTGCCGGATTTGG |  | 17 | 127 | 63.98 | False | 0.00 | 0.00 |
|  | VFG000256(gi:15676008)_rev |  | TCAGTTTTTATGCTCACG |  | 18 | 127 | 56.14 | False | 0.00 | 0.00 |
|  | VFG000256(gi:15676008)_detection |  | TGATTATCGGCTTGA | TTT | 15 | 127 | 52.09 | False | 0.00 | 0.00 |
|  | VFG000256(gi:15676008)_lnc3 | GCAGCGTGGGCTCGCGTGCCGGGGTTTTTT | ACATTGTTGCCACTC |  | 15 | 127 | 54.93 | False | 0.00 | 0.00 |
| spec | VFG000978(gi:15674769)_fwd |  | AGTTTTCATAATTACAGTCATAC |  | 23 | 153 | 56.53 | False | 0.00 | 0.00 |
|  | VFG000978(gi:15674769)_rev |  | GTTGAAAAATTTACCCTGCAA |  | 21 | 153 | 59.52 | False | 0.00 | 0.00 |
|  | VFG000978(gi:15674769)_detection |  | ATTTCGAATGTTAAAAGT | TTT | 18 | 153 | 51.63 | False | 0.00 | 0.00 |
|  | VFG000978(gi:15674769)_lnc3 | GCAGCGTGGGCTCGCGTGCCGGGGTTTTTT | GACTCTAAGAAAGAC |  | 15 | 153 | 46.92 | False | 0.00 | 0.00 |
| toxA | VFG002287(gi:126698240)_fwd |  | GTAAAGATGAATTCAACACAAG |  | 22 | 172 | 57.77 | False | 0.00 | 0.00 |
|  | VFG002287(gi:126698240)_rev |  | TAAGTTTCTTCAACATTAAAATCA |  | 24 | 172 | 57.51 | False | 42.03 | -340.06 |
|  | VFG002287(gi:126698240)_detection |  | CATAAAATTAGATATATCAC | TTT | 20 | 172 | 48.19 | False | 0.00 | 0.00 |
|  | VFG002287(gi:126698240)_lnc3 | GCAGCGTGGGCTCGCGTGCCGGGGTTTTTT | AAGTTCATTTTTAGATAC |  | 18 | 172 | 48.60 | False | 0.00 | 0.00 |
| ctrC | VFG000257(gi:15676009)_fwd |  | CTTGGCTTTTGATGGCTTTTTTT |  | 23 | 120 | 63.68 | False | 0.00 | 0.00 |
|  | VFG000257(gi:15676009)_rev |  | ATAACGGCATCATCACAAAA |  | 20 | 120 | 59.64 | False | 0.00 | 0.00 |
|  | VFG000257(gi:15676009)_detection |  | GCCTTTAATTTCGA | TTT | 14 | 120 | 46.93 | False | 0.00 | 0.00 |
|  | VFG000257(gi:15676009)_lnc3 | GCAGCGTGGGCTCGCGTGCCGGGGTTTTTT | TGATTTGTTCGATT |  | 14 | 120 | 45.67 | False | 0.00 | 0.00 |
| cpsO | VFG001342(gi:22537323)_fwd |  | CTTATTCAATTGAGGTTATGTACA |  | 24 | 142 | 59.06 | False | 0.00 | 0.00 |
|  | VFG001342(gi:22537323)_rev |  | TCTAAAGTATAAAGTTTAATCTCT |  | 24 | 142 | 55.21 | False | 37.04 | -4.36 |
|  | VFG001342(gi:22537323)_lnc3 | GCAGCGTGGGCTCGCGTGCCGGGGTTTTTT | ATATTGTTTTAACTAGTATT |  | 20 | 142 | 49.08 | False | 0.00 | 0.00 |
|  | VFG001342(gi:22537323)_detection |  | GGTAACTTCAACAATACTTA | TTT | 20 | 142 | 54.93 | False | 0.00 | 0.00 |
| espB | VFG000744(gi:2865304)_fwd |  | GCTGAAGGTACGAAAACA |  | 18 | 76 | 58.44 | False | 0.00 | 0.00 |
|  | VFG000744(gi:2865304)_rev |  | CACGCTGCTCAYTGATA |  | 17 | 76 | 58.57 | False | 0.00 | 0.00 |
|  | VFG000744(gi:2865304)_lnc3 | GCAGCGTGGGCTCGCGTGCCGGGGTTTTTT | AACAACGATATCTG |  | 14 | 76 | 45.69 | False | 0.00 | 0.00 |
|  | VFG000744(gi:2865304)_detection |  | AATCTGTCAAGTCC | TTT | 14 | 76 | 48.35 | False | 0.00 | 0.00 |
| sspA | VFG001296(gi:21282661)_fwd |  | ACAGCAAACACCTAAGAT |  | 18 | 122 | 56.89 | False | 0.00 | 0.00 |
|  | VFG001296(gi:21282661)_rev |  | AATGACCATTCGTTGTATCT |  | 20 | 122 | 58.38 | False | 34.58 | 180.62 |
|  | VFG001296(gi:21282661)_detection |  | AGAACAACGTGAAC | TTT | 14 | 122 | 50.35 | False | 0.00 | 0.00 |
|  | VFG001296(gi:21282661)_lnc3 | GCAGCGTGGGCTCGCGTGCCGGGGTTTTTT | TAACCTTAAACCATT |  | 15 | 122 | 45.53 | False | 0.00 | 0.00 |
| cpsN | VFG001343(gi:22537324)_fwd |  | CAAGTGATTATCATGTATGTGTAG |  | 24 | 120 | 59.50 | False | 0.00 | 0.00 |
|  | VFG001343(gi:22537324)_rev |  | GATAAACAAGGCCTACATATC |  | 21 | 120 | 57.57 | False | 0.00 | 0.00 |
|  | VFG001343(gi:22537324)_detection |  | TGAAATTATTTTTAAAGAT | TTT | 19 | 120 | 47.16 | False | 0.00 | 0.00 |
|  | VFG001343(gi:22537324)_lnc3 | GCAGCGTGGGCTCGCGTGCCGGGGTTTTTT | GTTATTAGAAGATGCAGT |  | 18 | 120 | 53.29 | False | 0.00 | 0.00 |
| prgB/asc10_EF0485_asa1 | VFG002164_2172_2173_fwd |  | ACGTTTCTTCTAAAGATTTAGC |  | 22 | 116 | 58.61 | False | 43.94 | -718.08 |
|  | VFG002164_2172_2173_rev |  | AATTTTTTCATTTTTGGCTTT |  | 21 | 116 | 55.77 | False | 0.00 | 0.00 |
|  | VFG002164_2172_2173_detection |  | ACAAAAAGAACAAGCG | TTT | 16 | 116 | 54.42 | False | 0.00 | 0.00 |
|  | VFG002164_2172_2173_lnc3 | GCAGCGTGGGCTCGCGTGCCGGGGTTTTTT | AAAAGAAGTAGACCAACT |  | 18 | 116 | 54.89 | False | 0.00 | 0.00 |
| wzz | VFG000137(gi:15598356)_fwd |  | AGCTATCTACGCTTTTCTCA |  | 20 | 83 | 60.01 | False | 38.16 | -61.30 |
|  | VFG000137(gi:15598356)_rev |  | AACCTGCCACATCACTCAA |  | 19 | 83 | 63.05 | False | 0.00 | 0.00 |
|  | VFG000137(gi:15598356)_detection |  | GCATAGCGATTTTG | TTT | 14 | 83 | 49.34 | False | 0.00 | 0.00 |
|  | VFG000137(gi:15598356)_lnc3 | GCAGCGTGGGCTCGCGTGCCGGGGTTTTTT | TGTCTATGAGGCCC |  | 14 | 83 | 53.82 | False | 0.00 | 0.00 |
| inlA | VFG000075(gi:16802477)_fwd |  | AAATTTAAATCGGCTAGAAC |  | 20 | 116 | 55.35 | False | 0.00 | 0.00 |
|  | VFG000075(gi:16802477)_rev |  | ATGGTTTTAAATCTGTCACTTGAT |  | 24 | 116 | 61.29 | False | 0.00 | 0.00 |
|  | VFG000075(gi:16802477)_lnc3 | GCAGCGTGGGCTCGCGTGCCGGGGTTTTTT | AGTAACACGATTAGT |  | 15 | 116 | 48.17 | False | 0.00 | 0.00 |
|  | VFG000075(gi:16802477)_detection |  | GATATTAGTGCGCTTTC | TTT | 17 | 116 | 54.51 | False | 0.00 | 0.00 |
| wzy | VFG000138(gi:15598350)_fwd |  | TGAGCTTTCGTCTGGGGA |  | 18 | 94 | 63.95 | False | 0.00 | 0.00 |
|  | VFG000138(gi:15598350)_rev |  | ACATAACCCAACTGCATAT |  | 19 | 94 | 57.57 | False | 0.00 | 0.00 |
|  | VFG000138(gi:15598350)_detection |  | GCGAATGTAAACTTC | TTT | 15 | 94 | 50.16 | False | 0.00 | 0.00 |
|  | VFG000138(gi:15598350)_lnc3 | GCAGCGTGGGCTCGCGTGCCGGGGTTTTTT | CAATAATCCCGATATGAAT |  | 19 | 94 | 54.11 | False | 0.00 | 0.00 |

### VF-7

Supplementary Table S19: Primer and probe sequences and additional information of VF-7.

| Gene | Primer/Probe | Hybridization Sequence | Sequence | Spacer | Length | Product Size | Tm | Hairpin | Hairpin Tm | Hairpin delta G |
| --- | --- | --- | --- | --- | --- | --- | --- | --- | --- | --- |
| plcA | VFG000073(gi:16802247)_fwd |  | TTAAACAACAAAGTAGAGCAATTCG |  | 25 | 107 | 63.00 | False | 0.00 | 0.00 |
|  | VFG000073(gi:16802247)_rev |  | ATGTTTTTAATTGTTTGTTTTTCGG |  | 25 | 107 | 60.92 | False | 0.00 | 0.00 |
|  | VFG000073(gi:16802247)_detection |  | GGCATACTAATCATGGA | TTT | 17 | 107 | 54.50 | False | 0.00 | 0.00 |
|  | VFG000073(gi:16802247)_lnc3 | GCAGCGTGGGCTCGCGTGCCGGGGTTTTTT | GAAAAAGTTCGAGGATTA |  | 18 | 107 | 53.91 | False | 0.00 | 0.00 |
| rfaE | VFG000331(gi:16273426)_fwd |  | ATAATGTAGATTGCAAGGAT |  | 20 | 131 | 55.58 | False | 0.00 | 0.00 |
|  | VFG000331(gi:16273426)_rev |  | TTTGCGTGCAATTTGAAT |  | 18 | 131 | 57.71 | False | 39.35 | -177.47 |
|  | VFG000331(gi:16273426)_detection |  | TTGATTCTTTCTGAT | TTT | 15 | 131 | 45.79 | False | 0.00 | 0.00 |
|  | VFG000331(gi:16273426)_lnc3 | GCAGCGTGGGCTCGCGTGCCGGGGTTTTTT | AAAATTACGGTGCT |  | 14 | 131 | 49.19 | False | 0.00 | 0.00 |
| espH | VFG000808(gi:15804225)_fwd |  | TGTTTCTCAACGCTATTTAG |  | 20 | 147 | 57.20 | False | 39.54 | -168.82 |
|  | VFG000808(gi:15804225)_rev |  | AGATAATCCTCTTTCAGCTAAG |  | 22 | 147 | 58.98 | False | 0.00 | 0.00 |
|  | VFG000808(gi:15804225)_lnc3 | GCAGCGTGGGCTCGCGTGCCGGGGTTTTTT | TGGAAAGAAAATTAAATGAG |  | 20 | 147 | 53.96 | False | 0.00 | 0.00 |
|  | VFG000808(gi:15804225)_detection |  | AGACATATAACTCCACTTT | TTT | 19 | 147 | 54.95 | False | 0.00 | 0.00 |
| lspD | VFG001877(gi:52841551)_fwd |  | TCTCTCGAATTACAGGTAA |  | 19 | 126 | 55.88 | False | 0.00 | 0.00 |
|  | VFG001877(gi:52841551)_rev |  | ATACCTGCAGTACTGAGAGAA |  | 21 | 126 | 61.78 | False | 37.18 | -10.55 |
|  | VFG001877(gi:52841551)_detection |  | GTTTCATCAACGCC | TTT | 14 | 126 | 52.51 | False | 0.00 | 0.00 |
|  | VFG001877(gi:52841551)_lnc3 | GCAGCGTGGGCTCGCGTGCCGGGGTTTTTT | CAAGGAAAAGTATCGATT |  | 18 | 126 | 53.97 | False | 0.00 | 0.00 |
| cbpG | VFG001353(gi:15900312)_fwd |  | GGAGTGCATACTTTAGGAG |  | 19 | 114 | 58.10 | False | 41.84 | -291.75 |
|  | VFG001353(gi:15900312)_rev |  | CCATCCTTCAAGAGAGTA |  | 18 | 114 | 55.66 | False | 0.00 | 0.00 |
|  | VFG001353(gi:15900312)_detection |  | GTTAAATTAAATGAACG | TTT | 17 | 114 | 47.47 | False | 0.00 | 0.00 |
|  | VFG001353(gi:15900312)_lnc3 | GCAGCGTGGGCTCGCGTGCCGGGGTTTTTT | TCAAATTAACAGTGCA |  | 16 | 114 | 51.83 | False | 0.00 | 0.00 |
| xcpP | VFG000183(gi:15598300)_fwd |  | ATGATCCCTCGGCGATCT |  | 18 | 127 | 63.62 | False | 41.70 | -474.85 |
|  | VFG000183(gi:15598300)_rev |  | CGATGATCAGGGCAACAGC |  | 19 | 127 | 64.95 | False | 39.74 | -88.36 |
|  | VFG000183(gi:15598300)_lnc3 | GCAGCGTGGGCTCGCGTGCCGGGGTTTTTT | AACGATAAAGACCAGG |  | 16 | 127 | 54.08 | False | 0.00 | 0.00 |
|  | VFG000183(gi:15598300)_detection |  | AGTGATGTATTGCCTT | TTT | 16 | 127 | 53.47 | False | 0.00 | 0.00 |
| hmw1B | VFG001152(gi:475772)_fwd |  | CATTTAACCTTGGHATGGAAGA |  | 22 | 121 | 61.23 | False | 38.42 | -104.73 |
|  | VFG001152(gi:475772)_rev |  | CCTGAYACTGCAAATTTTTTCTT |  | 23 | 121 | 61.55 | False | 0.00 | 0.00 |
|  | VFG001152(gi:475772)_lnc3 | GCAGCGTGGGCTCGCGTGCCGGGGTTTTTT | AAATTAATTTAGGCTAC |  | 17 | 121 | 47.29 | False | 0.00 | 0.00 |
|  | VFG001152(gi:475772)_detection |  | AACTACCGCCATAT | TTT | 14 | 121 | 50.18 | False | 0.00 | 0.00 |
| siaA/synA | VFG000254(gi:15676006)_fwd |  | AAATTTGAAGCAGATGATACCTTT |  | 24 | 126 | 61.60 | False | 0.00 | 0.00 |
|  | VFG000254(gi:15676006)_rev |  | ATTCAAATCGATAAAACGTTTT |  | 22 | 126 | 57.42 | False | 39.75 | -321.00 |
|  | VFG000254(gi:15676006)_detection |  | GAAGTAATCAACAATC | TTT | 16 | 126 | 47.37 | False | 0.00 | 0.00 |
|  | VFG000254(gi:15676006)_lnc3 | GCAGCGTGGGCTCGCGTGCCGGGGTTTTTT | CACAGAAAGATTTGCT |  | 16 | 126 | 52.85 | False | 0.00 | 0.00 |
| map | VFG000806(gi:15804223)_fwd |  | GACGACTTATATATCCAGAACA |  | 22 | 118 | 58.80 | False | 0.00 | 0.00 |
|  | VFG000806(gi:15804223)_rev |  | TAACGCATTTTCAACAGCTCTA |  | 22 | 118 | 62.52 | False | 0.00 | 0.00 |
|  | VFG000806(gi:15804223)_detection |  | TATTGCAGGAGTAATAATTC | TTT | 20 | 118 | 54.83 | False | 34.99 | 192.16 |
|  | VFG000806(gi:15804223)_lnc3 | GCAGCGTGGGCTCGCGTGCCGGGGTTTTTT | GAACTCTGGATGAC |  | 14 | 118 | 49.21 | False | 0.00 | 0.00 |
| rrgC | VFG005305(gi:15900380)_fwd |  | TATCGTTTCAAGGAGGTGGA |  | 20 | 136 | 62.09 | False | 0.00 | 0.00 |
|  | VFG005305(gi:15900380)_rev |  | TAAAGTCAACATTGCCACGT |  | 20 | 136 | 61.98 | False | 0.00 | 0.00 |
|  | VFG005305(gi:15900380)_lnc3 | GCAGCGTGGGCTCGCGTGCCGGGGTTTTTT | TGGTAGATCATCAG |  | 14 | 136 | 46.68 | False | 0.00 | 0.00 |
|  | VFG005305(gi:15900380)_detection |  | CTGGTGACGATTAC | TTT | 14 | 136 | 49.90 | False | 0.00 | 0.00 |
| spei | VFG000952(gi:15675011)_fwd |  | GGGTGGATATATTTACTTTGG |  | 21 | 140 | 57.46 | False | 0.00 | 0.00 |
|  | VFG000952(gi:15675011)_rev |  | AACGCCGTCTATGAAAAT |  | 18 | 140 | 57.85 | False | 0.00 | 0.00 |
|  | VFG000952(gi:15675011)_detection |  | CGGTAATAGAATTGATA | TTT | 17 | 140 | 48.67 | False | 0.00 | 0.00 |
|  | VFG000952(gi:15675011)_lnc3 | GCAGCGTGGGCTCGCGTGCCGGGGTTTTTT | GTATTACTTTAAGCGA |  | 16 | 140 | 48.48 | False | 0.00 | 0.00 |
| licD | VFG000329(gi:16273440)_fwd |  | CTTTYTATTTTCCAAAATGCA |  | 21 | 99 | 56.31 | False | 43.35 | -595.52 |
|  | VFG000329(gi:16273440)_rev |  | CCGTAGTCAGATAATACRAGA |  | 21 | 99 | 58.99 | True | 48.15 | -1069.02 |
|  | VFG000329(gi:16273440)_detection |  | TAAAACAATGCGATTAT | TTT | 17 | 99 | 50.32 | False | 0.00 | 0.00 |
|  | VFG000329(gi:16273440)_lnc3 | GCAGCGTGGGCTCGCGTGCCGGGGTTTTTT | CAAATAAAATATCCGA |  | 16 | 99 | 46.04 | False | 0.00 | 0.00 |
| aap/aspU | VFG000865(gi:387604907)_fwd |  | ATCTTGGGTATCAGCCTGAATG |  | 22 | 142 | 64.18 | False | 40.66 | -244.76 |
|  | VFG000865(gi:387604907)_rev |  | CATTAAGGCCTTGCATACATACTG |  | 24 | 142 | 64.18 | False | 0.00 | 0.00 |
|  | VFG000865(gi:387604907)_detection |  | TCCCAATGTATAAAAC | TTT | 16 | 142 | 48.45 | False | 0.00 | 0.00 |
|  | VFG000865(gi:387604907)_lnc3 | GCAGCGTGGGCTCGCGTGCCGGGGTTTTTT | GATAATGTGGACCCG |  | 15 | 142 | 54.10 | False | 0.00 | 0.00 |
| pefD | VFG000433(gi:17233481)_fwd |  | CTTTGTGCTGAACGGCA |  | 17 | 119 | 61.53 | False | 42.08 | -366.10 |
|  | VFG000433(gi:17233481)_rev |  | GGGTGGTGTTGTCTATC |  | 17 | 119 | 56.97 | False | 0.00 | 0.00 |
|  | VFG000433(gi:17233481)_detection |  | GGAGAAAGAACACCT | TTT | 15 | 119 | 52.66 | False | 0.00 | 0.00 |
|  | VFG000433(gi:17233481)_lnc3 | GCAGCGTGGGCTCGCGTGCCGGGGTTTTTT | TTTATCTATGAGGAAG |  | 16 | 119 | 46.52 | False | 0.00 | 0.00 |
| hmw1C | VFG001153(gi:475773)_fwd |  | GGTGGCTTTCATTTAGCAA |  | 19 | 107 | 59.86 | False | 44.00 | -545.52 |
|  | VFG001153(gi:475773)_rev |  | TTCCCTGCCCATAACGC |  | 17 | 107 | 62.84 | False | 0.00 | 0.00 |
|  | VFG001153(gi:475773)_lnc3 | GCAGCGTGGGCTCGCGTGCCGGGGTTTTTT | AACTCTTCTATTGCTAAA |  | 18 | 107 | 52.25 | False | 0.00 | 0.00 |
|  | VFG001153(gi:475773)_detection |  | TTCTGTATTTTTTACTT | TTT | 17 | 107 | 46.47 | False | 0.00 | 0.00 |
| eae | VFG000739(gi:2865299)_fwd |  | ATCTGGTTCAGCGTAAT |  | 17 | 121 | 55.80 | False | 0.00 | 0.00 |
|  | VFG000739(gi:2865299)_rev |  | ACGATCAATTGAATCTTCT |  | 19 | 121 | 55.28 | False | 0.00 | 0.00 |
|  | VFG000739(gi:2865299)_lnc3 | GCAGCGTGGGCTCGCGTGCCGGGGTTTTTT | AATATTCCGCATGATA |  | 16 | 121 | 50.26 | False | 0.00 | 0.00 |
|  | VFG000739(gi:2865299)_detection |  | TTAATGGTACTGAAC | TTT | 15 | 121 | 46.55 | False | 0.00 | 0.00 |
| srtC-2/srtC | VFG005308(gi:15900383)_fwd |  | CGGAATCGAGCGGTGAG |  | 17 | 134 | 63.29 | False | 41.38 | -256.22 |
|  | VFG005308(gi:15900383)_rev |  | TCCAATTGCTTTTCTAGTCCTTTG |  | 24 | 134 | 63.92 | False | 0.00 | 0.00 |
|  | VFG005308(gi:15900383)_detection |  | CGGTCATCCTTCTC | TTT | 14 | 134 | 52.43 | False | 0.00 | 0.00 |
|  | VFG005308(gi:15900383)_lnc3 | GCAGCGTGGGCTCGCGTGCCGGGGTTTTTT | TACTAGGAGCGATGG |  | 15 | 134 | 54.39 | False | 0.00 | 0.00 |
| yagV/ecpE | VFG002417(gi:15799994)_fwd |  | TTTCGGTCGGCAATCTGA |  | 18 | 104 | 62.59 | False | 0.00 | 0.00 |
|  | VFG002417(gi:15799994)_rev |  | GATGGCAATACGGTATATC |  | 19 | 104 | 56.15 | False | 0.00 | 0.00 |
|  | VFG002417(gi:15799994)_lnc3 | GCAGCGTGGGCTCGCGTGCCGGGGTTTTTT | CCGAGACTGACTTT |  | 14 | 104 | 51.62 | False | 0.00 | 0.00 |
|  | VFG002417(gi:15799994)_detection |  | GTCAGCAAACGTGT | TTT | 14 | 104 | 54.34 | False | 0.00 | 0.00 |
| mip | VFG001864(gi:52841028)_fwd |  | AATGGCTGCAACCGATG |  | 17 | 139 | 61.38 | False | 0.00 | 0.00 |
|  | VFG001864(gi:52841028)_rev |  | GTCTTGCATGCCTTTAG |  | 17 | 139 | 56.01 | False | 0.00 | 0.00 |
|  | VFG001864(gi:52841028)_detection |  | TTGGTGCCGATTTG | TTT | 14 | 139 | 54.34 | False | 0.00 | 0.00 |
|  | VFG001864(gi:52841028)_lnc3 | GCAGCGTGGGCTCGCGTGCCGGGGTTTTTT | GTTGTCTTATAGCA |  | 14 | 139 | 45.03 | False | 0.00 | 0.00 |
| east1_astA | VFG045328_VFG000863_fwd |  | ATCAACACAGTATATCCGR |  | 19 | 104 | 56.91 | False | 0.00 | 0.00 |
|  | VFG045328_VFG000863_rev |  | CGAGTGACGGCYTTGTA |  | 17 | 104 | 60.95 | False | 40.40 | -243.64 |
|  | VFG045328_VFG000863_detection |  | CATCGTGCATATG | TTT | 13 | 104 | 46.39 | False | 0.00 | 0.00 |
|  | VFG045328_VFG000863_lnc3 | GCAGCGTGGGCTCGCGTGCCGGGGTTTTTT | GCATCCAGTTATG |  | 13 | 104 | 45.41 | False | 0.00 | 0.00 |
| ace | VFG002166(gi:29375675)_fwd |  | CTCAATAAATCCGATGTCA |  | 19 | 133 | 55.80 | False | 0.00 | 0.00 |
|  | VFG002166(gi:29375675)_rev |  | ACTCGGCAAGTGAAATAT |  | 18 | 133 | 57.08 | False | 41.39 | -255.47 |
|  | VFG002166(gi:29375675)_lnc3 | GCAGCGTGGGCTCGCGTGCCGGGGTTTTTT | AAAGAGAGTTTTACA |  | 15 | 133 | 45.65 | False | 0.00 | 0.00 |
|  | VFG002166(gi:29375675)_detection |  | TTTGATATTGTGAATG | TTT | 16 | 133 | 46.68 | False | 0.00 | 0.00 |
| licB | VFG000327(gi:16273438)_fwd |  | TTTATCTGTTAAAACCAATCAA |  | 22 | 110 | 56.06 | False | 0.00 | 0.00 |
|  | VFG000327(gi:16273438)_rev |  | TAAGGTAATGGGCTTAATTGG |  | 21 | 110 | 59.43 | False | 0.00 | 0.00 |
|  | VFG000327(gi:16273438)_lnc3 | GCAGCGTGGGCTCGCGTGCCGGGGTTTTTT | TGGCATTAAATATCA |  | 15 | 110 | 45.95 | False | 0.00 | 0.00 |
|  | VFG000327(gi:16273438)_detection |  | CATATTCTGTATGGG | TTT | 15 | 110 | 46.98 | False | 0.00 | 0.00 |
| llsY | VFG045336(gi:588561369)_fwd |  | TTAATACAAATAGAATCCAAAGTTG |  | 25 | 116 | 58.32 | False | 0.00 | 0.00 |
|  | VFG045336(gi:588561369)_rev |  | ATCACTACTCATCAGCTCC |  | 19 | 116 | 59.40 | False | 0.00 | 0.00 |
|  | VFG045336(gi:588561369)_detection |  | ATAATATATTATTTGAAGAG | TTT | 20 | 116 | 46.90 | False | 33.14 | 279.46 |
|  | VFG045336(gi:588561369)_lnc3 | GCAGCGTGGGCTCGCGTGCCGGGGTTTTTT | CCTGTGCTACTTTCA |  | 15 | 116 | 53.24 | False | 0.00 | 0.00 |
| inlB | VFG000076(gi:16802478)_fwd |  | AATTAAAATCACTTTCTTTGGAG |  | 23 | 124 | 57.74 | False | 0.00 | 0.00 |
|  | VFG000076(gi:16802478)_rev |  | CGTGAAAGAACCGTTATATC |  | 20 | 124 | 57.74 | False | 0.00 | 0.00 |
|  | VFG000076(gi:16802478)_lnc3 | GCAGCGTGGGCTCGCGTGCCGGGGTTTTTT | CACAGCTGGAAAGT |  | 14 | 124 | 52.81 | False | 0.00 | 0.00 |
|  | VFG000076(gi:16802478)_detection |  | TTGTATTTGGGAAATA | TTT | 16 | 124 | 47.27 | False | 34.90 | 169.00 |
| rfaD | VFG000332(gi:16273039)_fwd |  | TTCTAAATTCTTGTTCGAYCA |  | 21 | 119 | 58.07 | False | 0.00 | 0.00 |
|  | VFG000332(gi:16273039)_rev |  | CCATWGAACCTTTATGATTTTCAC |  | 24 | 119 | 60.66 | False | 36.06 | 0.00 |
|  | VFG000332(gi:16273039)_detection |  | TTCAATGTTTACGG | TTT | 14 | 119 | 46.85 | False | 0.00 | 0.00 |
|  | VFG000332(gi:16273039)_lnc3 | GCAGCGTGGGCTCGCGTGCCGGGGTTTTTT | GTGGTTTCCGTTAT |  | 14 | 119 | 49.42 | False | 0.00 | 0.00 |
| pilC | VFG000113(gi:15599723)_fwd |  | ACTCGACAGAACGATCC |  | 17 | 136 | 58.86 | False | 44.70 | -652.00 |
|  | VFG000113(gi:15599723)_rev |  | GGAGACGGAGTCGAGCG |  | 17 | 136 | 65.00 | True | 69.37 | -2325.02 |
|  | VFG000113(gi:15599723)_lnc3 | GCAGCGTGGGCTCGCGTGCCGGGGTTTTTT | GTTATGCACGGACC |  | 14 | 136 | 54.24 | False | 0.00 | 0.00 |
|  | VFG000113(gi:15599723)_detection |  | TTGTCCACGACCTT | TTT | 14 | 136 | 54.39 | False | 0.00 | 0.00 |
| nleA | VFG045339(gi:391314561)_fwd |  | TCTGGTAAATATGGTGTTCAG |  | 21 | 149 | 59.09 | False | 0.00 | 0.00 |
|  | VFG045339(gi:391314561)_rev |  | TGAGAGAGAAACTTCTGGA |  | 19 | 149 | 58.36 | False | 42.12 | -323.33 |
|  | VFG045339(gi:391314561)_detection |  | CCCTACATGATGAG | TTT | 14 | 149 | 48.33 | False | 0.00 | 0.00 |
|  | VFG045339(gi:391314561)_lnc3 | GCAGCGTGGGCTCGCGTGCCGGGGTTTTTT | TTTTTACAGTTATTCC |  | 16 | 149 | 46.05 | False | 0.00 | 0.00 |
| espF | VFG000794(gi:15804211)_fwd |  | GTAACGCTGCTTCTACACTAG |  | 21 | 86 | 62.17 | False | 0.00 | 0.00 |
|  | VFG000794(gi:15804211)_rev |  | GGCTACAGAAAATCCAGTTCC |  | 21 | 86 | 62.71 | False | 0.00 | 0.00 |
|  | VFG000794(gi:15804211)_detection |  | GCAAGTCGAGTGAG | TTT | 14 | 86 | 53.81 | False | 0.00 | 0.00 |
|  | VFG000794(gi:15804211)_lnc3 | GCAGCGTGGGCTCGCGTGCCGGGGTTTTTT | AGCTTGTAGGTATC |  | 14 | 86 | 47.15 | False | 0.00 | 0.00 |
| pilY1 | VFG001210(gi:15599750)_fwd |  | CCAACCTGGCGTTCTACAG |  | 19 | 130 | 64.16 | False | 35.27 | 87.39 |
|  | VFG001210(gi:15599750)_rev |  | AAGTCCCGTAGATAGTTG |  | 18 | 130 | 56.12 | False | 0.00 | 0.00 |
|  | VFG001210(gi:15599750)_detection |  | CTCGAACTGCAACC | TTT | 14 | 130 | 54.58 | False | 0.00 | 0.00 |
|  | VFG001210(gi:15599750)_lnc3 | GCAGCGTGGGCTCGCGTGCCGGGGTTTTTT | CAATTGCTGAACGA |  | 14 | 130 | 50.97 | False | 0.00 | 0.00 |
| pitA | VFG042971(gi:225860989)_fwd |  | CTAGAACGGGATATCATGTCAC |  | 22 | 123 | 61.98 | False | 0.00 | 0.00 |
|  | VFG042971(gi:225860989)_rev |  | AAGCACCTGTAACTTTTTTTGTGA |  | 24 | 123 | 64.23 | False | 0.00 | 0.00 |
|  | VFG042971(gi:225860989)_detection |  | GTTCAAACAATCGT | TTT | 14 | 123 | 47.79 | False | 0.00 | 0.00 |
|  | VFG042971(gi:225860989)_lnc3 | GCAGCGTGGGCTCGCGTGCCGGGGTTTTTT | ACTTTGTCAGAGGAT |  | 15 | 123 | 51.23 | False | 0.00 | 0.00 |
| licA | VFG000326(gi:16273437)_fwd |  | GTATTTAGCGTATTCGATGAGT |  | 22 | 113 | 60.00 | False | 41.90 | -312.43 |
|  | VFG000326(gi:16273437)_rev |  | AATTGCCAAAATACAGCTGAAAG |  | 23 | 113 | 62.73 | False | 0.00 | 0.00 |
|  | VFG000326(gi:16273437)_lnc3 | GCAGCGTGGGCTCGCGTGCCGGGGTTTTTT | CAATATTTTTCATTGCTAGA |  | 20 | 113 | 53.84 | False | 0.00 | 0.00 |
|  | VFG000326(gi:16273437)_detection |  | AAACAAATCCGCTTTTT | TTT | 17 | 113 | 54.83 | False | 0.00 | 0.00 |
| icmT | VFG001830(gi:52840686)_fwd |  | TATAAGRGTATGGACAGGTGTTT |  | 23 | 119 | 61.96 | False | 0.00 | 0.00 |
|  | VFG001830(gi:52840686)_rev |  | AACCAGCTAAAAAACTTCTGAG |  | 22 | 119 | 61.00 | False | 0.00 | 0.00 |
|  | VFG001830(gi:52840686)_detection |  | ACTGTCCCGGTTTT | TTT | 14 | 119 | 54.76 | False | 0.00 | 0.00 |
|  | VFG001830(gi:52840686)_lnc3 | GCAGCGTGGGCTCGCGTGCCGGGGTTTTTT | TGAACATTATGGTTTC |  | 16 | 119 | 49.30 | False | 0.00 | 0.00 |
| lic2A | VFG000333(gi:16272494)_fwd |  | ACAAACTAAAATTCCACCTTTT |  | 22 | 150 | 58.84 | False | 0.00 | 0.00 |
|  | VFG000333(gi:16272494)_rev |  | ACTGCAACAATTTCATCAG |  | 19 | 150 | 57.58 | False | 0.00 | 0.00 |
|  | VFG000333(gi:16272494)_lnc3 | GCAGCGTGGGCTCGCGTGCCGGGGTTTTTT | GGTTATATTATTTCTCAA |  | 18 | 150 | 47.57 | False | 0.00 | 0.00 |
|  | VFG000333(gi:16272494)_detection |  | GGTGCGGCTAAATA | TTT | 14 | 150 | 52.77 | False | 0.00 | 0.00 |
| fctA | VFG042968(gi:71909923)_fwd |  | GTTACAAAAAACCTTGATTTAG |  | 22 | 134 | 55.98 | False | 0.00 | 0.00 |
|  | VFG042968(gi:71909923)_rev |  | ATCGGTGTGTTCAAAGCT |  | 18 | 134 | 60.43 | False | 0.00 | 0.00 |
|  | VFG042968(gi:71909923)_lnc3 | GCAGCGTGGGCTCGCGTGCCGGGGTTTTTT | CAGATTTTACATTTAAA |  | 17 | 134 | 45.41 | False | 0.00 | 0.00 |
|  | VFG042968(gi:71909923)_detection |  | ATCGAACCTGATAC | TTT | 14 | 134 | 47.84 | False | 0.00 | 0.00 |
| hlyC | VFG000905(gi:26249404)_fwd |  | ATGGTTCATTGWCTGGATTGCT |  | 22 | 147 | 64.61 | False | 0.00 | 0.00 |
|  | VFG000905(gi:26249404)_rev |  | TTACCTCCRTGAAATTCTGATACTT |  | 25 | 147 | 63.39 | False | 0.00 | 0.00 |
|  | VFG000905(gi:26249404)_detection |  | TCCCTGATGAACTATTC | TTT | 17 | 147 | 53.96 | False | 0.00 | 0.00 |
|  | VFG000905(gi:26249404)_lnc3 | GCAGCGTGGGCTCGCGTGCCGGGGTTTTTT | TATATGCGAAAAAAAT |  | 16 | 147 | 46.23 | False | 0.00 | 0.00 |
| eltB | VFG002038(gi:145831)_fwd |  | ATTGAAAGGATGAAGGACACAT |  | 22 | 107 | 61.97 | False | 0.00 | 0.00 |
|  | VFG002038(gi:145831)_rev |  | CTGATTGCCGCAATTGA |  | 17 | 107 | 58.86 | False | 39.61 | -187.81 |
|  | VFG002038(gi:145831)_detection |  | GGAATAATAAAACCC | TTT | 15 | 107 | 45.09 | False | 0.00 | 0.00 |
|  | VFG002038(gi:145831)_lnc3 | GCAGCGTGGGCTCGCGTGCCGGGGTTTTTT | ATTGATAAATTATGTGTAT |  | 19 | 107 | 48.37 | False | 0.00 | 0.00 |
| kdtA | VFG000330(gi:16272595)_fwd |  | TTTTTTTATACCAGCTTGCTTTT |  | 23 | 118 | 60.12 | False | 0.00 | 0.00 |
|  | VFG000330(gi:16272595)_rev |  | CATAAAAACCATAACGTTCA |  | 20 | 118 | 55.59 | False | 0.00 | 0.00 |
|  | VFG000330(gi:16272595)_detection |  | CTTGCTCAGTGTTA | TTT | 14 | 118 | 48.93 | False | 0.00 | 0.00 |
|  | VFG000330(gi:16272595)_lnc3 | GCAGCGTGGGCTCGCGTGCCGGGGTTTTTT | TGATATTGTGTTTTATTGG |  | 19 | 118 | 52.82 | False | 0.00 | 0.00 |
| iga | VFG000261(gi:15676598)_fwd |  | CTTCGGTGATAATTCAAAACAT |  | 22 | 111 | 59.20 | False | 36.98 | 1.30 |
|  | VFG000261(gi:15676598)_rev |  | GCAAATAATGGTGAACCGC |  | 19 | 111 | 61.36 | False | 41.89 | -276.52 |
|  | VFG000261(gi:15676598)_detection |  | TTTTATCTCAAAACGCA | TTT | 17 | 111 | 53.48 | False | 0.00 | 0.00 |
|  | VFG000261(gi:15676598)_lnc3 | GCAGCGTGGGCTCGCGTGCCGGGGTTTTTT | AAAAACTCAAAGAAG |  | 15 | 111 | 45.58 | False | 0.00 | 0.00 |
| lspE | VFG001876(gi:52841550)_fwd |  | CCACTTGGGTTATAARGG |  | 18 | 111 | 56.43 | False | 0.00 | 0.00 |
|  | VFG001876(gi:52841550)_rev |  | AAATAATTTTCCATBGTTTGAA |  | 22 | 111 | 56.38 | False | 0.00 | 0.00 |
|  | VFG001876(gi:52841550)_lnc3 | GCAGCGTGGGCTCGCGTGCCGGGGTTTTTT | GGAATTTATGAATTAATT |  | 18 | 111 | 46.50 | True | 39.43 | -198.18 |
|  | VFG001876(gi:52841550)_detection |  | ATCGTAGATGAAAC | TTT | 14 | 111 | 45.26 | True | 47.73 | -434.86 |
| pavA | VFG005197(gi:15902912)_fwd |  | AATGCCCAACGCTATTTTA |  | 19 | 107 | 58.93 | False | 41.21 | -240.81 |
|  | VFG005197(gi:15902912)_rev |  | ACACTTTCCAGATAGAGAAT |  | 20 | 107 | 57.00 | False | 0.00 | 0.00 |
|  | VFG005197(gi:15902912)_lnc3 | GCAGCGTGGGCTCGCGTGCCGGGGTTTTTT | TCAGAAACTCAAAGA |  | 15 | 107 | 48.66 | False | 0.00 | 0.00 |
|  | VFG005197(gi:15902912)_detection |  | AGCTGTCAAATACTT | TTT | 15 | 107 | 49.25 | False | 0.00 | 0.00 |
| srtC-1/srtB | VFG005307(gi:15900382)_fwd |  | GTACGTAGCAGAGGTTG |  | 17 | 119 | 57.13 | False | 0.00 | 0.00 |
|  | VFG005307(gi:15900382)_rev |  | AGCGTCGAATAATCCATAA |  | 19 | 119 | 57.48 | False | 0.00 | 0.00 |
|  | VFG005307(gi:15900382)_detection |  | TGTTTTATGTGGCAG | TTT | 15 | 119 | 51.63 | False | 0.00 | 0.00 |
|  | VFG005307(gi:15900382)_lnc3 | GCAGCGTGGGCTCGCGTGCCGGGGTTTTTT | TCTCTATCGCTACC |  | 14 | 119 | 49.70 | False | 0.00 | 0.00 |
| vexC | VFG000425(gi:16763115)_fwd |  | AGAAAGGGTTGATTGTGCTA |  | 20 | 91 | 60.75 | False | 0.00 | 0.00 |
|  | VFG000425(gi:16763115)_rev |  | ATTGTAATTTTTCCATGCAGCAG |  | 23 | 91 | 62.79 | False | 0.00 | 0.00 |
|  | VFG000425(gi:16763115)_detection |  | TAAAGAACACTGTCAT | TTT | 16 | 91 | 50.17 | False | 0.00 | 0.00 |
|  | VFG000425(gi:16763115)_lnc3 | GCAGCGTGGGCTCGCGTGCCGGGGTTTTTT | AACCCTCGGCTTAT |  | 14 | 91 | 53.65 | False | 0.00 | 0.00 |
| tviB | VFG000431(gi:16763123)_fwd |  | ACCTTTAAAGAAAACTGTCCGG |  | 22 | 128 | 63.00 | False | 0.00 | 0.00 |
|  | VFG000431(gi:16763123)_rev |  | TCTCGTCTTACCTCTTCG |  | 18 | 128 | 58.59 | False | 0.00 | 0.00 |
|  | VFG000431(gi:16763123)_lnc3 | GCAGCGTGGGCTCGCGTGCCGGGGTTTTTT | CATTATTGATGTGGTAAA |  | 18 | 128 | 51.46 | False | 0.00 | 0.00 |
|  | VFG000431(gi:16763123)_detection |  | GGAACTCGGTAAATATA | TTT | 17 | 128 | 51.16 | False | 0.00 | 0.00 |
| set1A | VFG000859(gi:387610010)_fwd |  | ACGGTTTTCCCAGTCTTTC |  | 19 | 141 | 61.39 | False | 38.81 | -104.34 |
|  | VFG000859(gi:387610010)_rev |  | TTTGATGGTAGCGTGAACC |  | 19 | 141 | 61.54 | False | 39.35 | -178.98 |
|  | VFG000859(gi:387610010)_detection |  | CTGGAATATCAGGGT | TTT | 15 | 141 | 51.54 | False | 0.00 | 0.00 |
|  | VFG000859(gi:387610010)_lnc3 | GCAGCGTGGGCTCGCGTGCCGGGGTTTTTT | GATAACCGGATGTCC |  | 15 | 141 | 53.71 | False | 0.00 | 0.00 |
| icaA | VFG001285(gi:21284315)_fwd |  | AGACTATACATTTATGACATATAG |  | 24 | 145 | 55.32 | False | 37.15 | -18.37 |
|  | VFG001285(gi:21284315)_rev |  | TACAAATATGAGTCCAGCCATA |  | 22 | 145 | 60.68 | False | 0.00 | 0.00 |
|  | VFG001285(gi:21284315)_detection |  | TGACTTTTATAAACGTTAT | TTT | 19 | 145 | 51.40 | False | 0.00 | 0.00 |
|  | VFG001285(gi:21284315)_lnc3 | GCAGCGTGGGCTCGCGTGCCGGGGTTTTTT | TACTATCATCATTTACTA |  | 18 | 145 | 47.38 | False | 0.00 | 0.00 |

### VF-8

Supplementary Table S20: Primer and probe sequences and additional information of VF-8.

| Gene | Primer/Probe | Hybridization Sequence | Sequence | Spacer | Length | Product Size | Tm | Hairpin | Hairpin Tm | Hairpin delta G |
| --- | --- | --- | --- | --- | --- | --- | --- | --- | --- | --- |
| vWbp | VFG002418(gi:151220969)_fwd |  | GTTAGAAAATAAAAAAGAAGACTTA |  | 25 | 156 | 56.44 | False | 39.08 | -141.57 |
|  | VFG002418(gi:151220969)_rev |  | GCTTCAGTGTCAGATTTTAATTG |  | 23 | 156 | 60.62 | False | 0.00 | 0.00 |
|  | VFG002418(gi:151220969)_lnc3 | GCAGCGTGGGCTCGCGTGCCGGGGTTTTTT | CCTAATAATATTCCTGTT |  | 18 | 156 | 49.63 | False | 0.00 | 0.00 |
|  | VFG002418(gi:151220969)_detection |  | TTAGAAGATGAAAAACAAG | TTT | 19 | 156 | 52.70 | False | 0.00 | 0.00 |
| icmC/dotE | VFG001841(gi:52840698)_fwd |  | ATTTGTAAGAGGGTGGGTATT |  | 21 | 156 | 60.50 | False | 0.00 | 0.00 |
|  | VFG001841(gi:52840698)_rev |  | TAAGTACCATATAAAGTATTGTTAA |  | 25 | 156 | 56.00 | False | 0.00 | 0.00 |
|  | VFG001841(gi:52840698)_detection |  | GGGGAATATTGGCAA | TTT | 15 | 156 | 53.68 | False | 0.00 | 0.00 |
|  | VFG001841(gi:52840698)_lnc3 | GCAGCGTGGGCTCGCGTGCCGGGGTTTTTT | GTTTGATTCATGTATTTG |  | 18 | 156 | 51.03 | False | 0.00 | 0.00 |
| hasA | VFG000962(gi:15675933)_fwd |  | TTTGATATCTATCTTGATTTATCTA |  | 25 | 114 | 55.62 | False | 37.08 | -6.98 |
|  | VFG000962(gi:15675933)_rev |  | AGGAAAGATAATCCAAGTTTAATA |  | 24 | 114 | 57.90 | False | 43.29 | -469.01 |
|  | VFG000962(gi:15675933)_lnc3 | GCAGCGTGGGCTCGCGTGCCGGGGTTTTTT | TGGAACATCAACTG |  | 14 | 114 | 49.26 | False | 0.00 | 0.00 |
|  | VFG000962(gi:15675933)_detection |  | TAGGAATTTATGGAGT | TTT | 16 | 114 | 48.50 | False | 0.00 | 0.00 |
| hylP | VFG000967(gi:15675354)_fwd |  | TTAAAAAACAGAAAGGCGGAAA |  | 22 | 94 | 61.47 | False | 0.00 | 0.00 |
|  | VFG000967(gi:15675354)_rev |  | TTTCTGATTCTGAGCATTTT |  | 20 | 94 | 56.97 | False | 0.00 | 0.00 |
|  | VFG000967(gi:15675354)_lnc3 | GCAGCGTGGGCTCGCGTGCCGGGGTTTTTT | GAATCTACATTAACTC |  | 16 | 94 | 45.84 | False | 0.00 | 0.00 |
|  | VFG000967(gi:15675354)_detection |  | AACATCAGGCACAG | TTT | 14 | 94 | 52.91 | False | 0.00 | 0.00 |
| flhA | VFG001263(gi:15596649)_fwd |  | CTGATTCTCTTCATCAACCTGAT |  | 23 | 92 | 62.31 | False | 42.27 | -315.81 |
|  | VFG001263(gi:15596649)_rev |  | AGGGCGTAGACCTTGCC |  | 17 | 92 | 64.64 | True | 110.77 | -2459.60 |
|  | VFG001263(gi:15596649)_detection |  | GCATTCGATGTCCTT | TTT | 15 | 92 | 54.38 | False | 0.00 | 0.00 |
|  | VFG001263(gi:15596649)_lnc3 | GCAGCGTGGGCTCGCGTGCCGGGGTTTTTT | ATCGGCATGATCCA |  | 14 | 92 | 54.35 | False | 33.37 | 149.09 |
| xcpT | VFG000180(gi:15598297)_fwd |  | GACATCAAGGCCATCGCT |  | 18 | 122 | 63.47 | False | 36.73 | 15.96 |
|  | VFG000180(gi:15598297)_rev |  | TTCTTCGGCTGCGGATTG |  | 18 | 122 | 64.05 | False | 0.00 | 0.00 |
|  | VFG000180(gi:15598297)_detection |  | AACTTCGCCTATCC | TTT | 14 | 122 | 51.77 | False | 0.00 | 0.00 |
|  | VFG000180(gi:15598297)_lnc3 | GCAGCGTGGGCTCGCGTGCCGGGGTTTTTT | TGTACAAGCTGGAC |  | 14 | 122 | 52.00 | False | 0.00 | 0.00 |
| icmG/dotF | VFG001840(gi:52840697)_fwd |  | ACAAGCAGTAATTCCAGGTCG |  | 21 | 132 | 64.43 | False | 0.00 | 0.00 |
|  | VFG001840(gi:52840697)_rev |  | GTTAAAATACGACCTTGCAAAGA |  | 23 | 132 | 61.89 | True | 56.20 | -997.01 |
|  | VFG001840(gi:52840697)_detection |  | GGTTCTACACTTACAG | TTT | 16 | 132 | 51.46 | False | 0.00 | 0.00 |
|  | VFG001840(gi:52840697)_lnc3 | GCAGCGTGGGCTCGCGTGCCGGGGTTTTTT | GCTAATTGGCTCAAAT |  | 16 | 132 | 52.98 | False | 0.00 | 0.00 |
| clfB | VFG001290(gi:21284280)_fwd |  | ACTTATAACTATAGTTCGCC |  | 20 | 147 | 55.47 | False | 42.57 | -475.97 |
|  | VFG001290(gi:21284280)_rev |  | ACCTAAAACTCGTTGCT |  | 17 | 147 | 56.34 | False | 0.00 | 0.00 |
|  | VFG001290(gi:21284280)_detection |  | CAGCTTCAGGTCAA | TTT | 14 | 147 | 52.40 | False | 0.00 | 0.00 |
|  | VFG001290(gi:21284280)_lnc3 | GCAGCGTGGGCTCGCGTGCCGGGGTTTTTT | AAATTATTGGTGTAGATA |  | 18 | 147 | 49.06 | False | 0.00 | 0.00 |
| nanA | VFG001378(gi:15903579)_fwd |  | GGATATAGATATAGAGAGGAACAG |  | 24 | 150 | 58.87 | False | 0.00 | 0.00 |
|  | VFG001378(gi:15903579)_rev |  | GCCCCTTCTTGAGCTAAAACA |  | 21 | 150 | 64.69 | False | 0.00 | 0.00 |
|  | VFG001378(gi:15903579)_lnc3 | GCAGCGTGGGCTCGCGTGCCGGGGTTTTTT | AGTGTCGTTATAGCATTA |  | 18 | 150 | 54.65 | False | 0.00 | 0.00 |
|  | VFG001378(gi:15903579)_detection |  | GGAAACTATCGGTA | TTT | 14 | 150 | 47.25 | False | 0.00 | 0.00 |
| llsD | VFG045337(gi:588561370)_fwd |  | AGTATGGAAAACCTGAATTGCAATA |  | 25 | 155 | 63.63 | False | 35.61 | 111.25 |
|  | VFG045337(gi:588561370)_rev |  | ATTCATATATCTAAACTCTGAAGGT |  | 25 | 155 | 59.68 | False | 0.00 | 0.00 |
|  | VFG045337(gi:588561370)_detection |  | GAAGGCGCTTTTAGA | TTT | 15 | 155 | 54.30 | False | 0.00 | 0.00 |
|  | VFG045337(gi:588561370)_lnc3 | GCAGCGTGGGCTCGCGTGCCGGGGTTTTTT | AGAAAAAGTTACAAATCT |  | 18 | 155 | 50.68 | False | 0.00 | 0.00 |
| ctrA | VFG000255(gi:15676007)_fwd |  | GATGTGGTTACCATGATTA |  | 19 | 148 | 55.35 | False | 40.12 | -177.28 |
|  | VFG000255(gi:15676007)_rev |  | CATCAGAACGGCGATCTT |  | 18 | 148 | 60.74 | False | 0.00 | 0.00 |
|  | VFG000255(gi:15676007)_lnc3 | GCAGCGTGGGCTCGCGTGCCGGGGTTTTTT | TGGGGAGAACACAA |  | 14 | 148 | 53.72 | False | 0.00 | 0.00 |
|  | VFG000255(gi:15676007)_detection |  | GAAATCGGTTTTTC | TTT | 14 | 148 | 45.91 | False | 0.00 | 0.00 |
| cylS | VFG045473(gi:21693303)_fwd |  | GAAATGGAAGCGATTCAAGG |  | 20 | 86 | 61.02 | False | 0.00 | 0.00 |
|  | VFG045473(gi:21693303)_rev |  | AATGCACCTACTCCTAAGC |  | 19 | 86 | 60.32 | False | 0.00 | 0.00 |
|  | VFG045473(gi:21693303)_lnc3 | GCAGCGTGGGCTCGCGTGCCGGGGTTTTTT | TGTTCAGGCTGAGA |  | 14 | 86 | 53.49 | False | 0.00 | 0.00 |
|  | VFG045473(gi:21693303)_detection |  | CAACTCCAGCATGT | TTT | 14 | 86 | 52.91 | False | 0.00 | 0.00 |
| fliH | VFG001253(gi:15596300)_fwd |  | ATCCATGTCAACCCGCA |  | 17 | 131 | 62.02 | False | 0.00 | 0.00 |
|  | VFG001253(gi:15596300)_rev |  | ATGCGACTGTGCTCGGT |  | 17 | 131 | 64.91 | False | 37.61 | -47.11 |
|  | VFG001253(gi:15596300)_lnc3 | GCAGCGTGGGCTCGCGTGCCGGGGTTTTTT | ACGAAGAGAGCTGG |  | 14 | 131 | 54.82 | False | 0.00 | 0.00 |
|  | VFG001253(gi:15596300)_detection |  | CGGATTCTCGAGGA | TTT | 14 | 131 | 54.11 | False | 0.00 | 0.00 |
| icmL/dotI | VFG001837(gi:52840694)_fwd |  | TGCCAATATTAGTSACCTA |  | 19 | 90 | 56.22 | False | 0.00 | 0.00 |
|  | VFG001837(gi:52840694)_rev |  | TCAACGTTGAAACTCTRGTTA |  | 21 | 90 | 60.07 | False | 41.62 | -292.32 |
|  | VFG001837(gi:52840694)_lnc3 | GCAGCGTGGGCTCGCGTGCCGGGGTTTTTT | GCCAGTGAATTTACA |  | 15 | 90 | 51.23 | False | 0.00 | 0.00 |
|  | VFG001837(gi:52840694)_detection |  | CAACAAAATAACGTAG | TTT | 16 | 90 | 48.28 | False | 0.00 | 0.00 |
| srtG2 | VFG042975(gi:225860993)_fwd |  | TTGGCGATTATACGATAAC |  | 19 | 96 | 56.01 | False | 37.15 | -9.89 |
|  | VFG042975(gi:225860993)_rev |  | CCATAGAGTGTCCGTAGATG |  | 20 | 96 | 60.52 | False | 0.00 | 0.00 |
|  | VFG042975(gi:225860993)_detection |  | CCAACTTTACTGATTTTAAT | TTT | 20 | 96 | 53.66 | False | 0.00 | 0.00 |
|  | VFG042975(gi:225860993)_lnc3 | GCAGCGTGGGCTCGCGTGCCGGGGTTTTTT | CGACTATCGCTTTAATC |  | 17 | 96 | 53.88 | False | 34.85 | 117.51 |
| fnbA | VFG001283(gi:21284150)_fwd |  | CAGAAACACCAACACCG |  | 17 | 136 | 59.04 | False | 0.00 | 0.00 |
|  | VFG001283(gi:21284150)_rev |  | TTGATTTCAATAACAGGTGTT |  | 21 | 136 | 57.66 | False | 43.38 | -544.20 |
|  | VFG001283(gi:21284150)_lnc3 | GCAGCGTGGGCTCGCGTGCCGGGGTTTTTT | GTAAACCAGTACCA |  | 14 | 136 | 47.80 | False | 0.00 | 0.00 |
|  | VFG001283(gi:21284150)_detection |  | CCCGCAAAAGAAGAA | TTT | 15 | 136 | 54.94 | False | 0.00 | 0.00 |
| llsP | VFG045338(gi:588561371)_fwd |  | CAATATTTACTTTGTTTATTCTTG |  | 24 | 166 | 55.19 | False | 0.00 | 0.00 |
|  | VFG045338(gi:588561371)_rev |  | AAATAGTAAGCAACTTTTTCCACT |  | 24 | 166 | 61.48 | False | 42.40 | -520.14 |
|  | VFG045338(gi:588561371)_detection |  | ATTTTTTTCTTACCATTTA | TTT | 19 | 166 | 49.76 | False | 0.00 | 0.00 |
|  | VFG045338(gi:588561371)_lnc3 | GCAGCGTGGGCTCGCGTGCCGGGGTTTTTT | TTCTCGCTGATATGT |  | 15 | 166 | 51.73 | False | 0.00 | 0.00 |
| fbaB | VFG000948(gi:21909640)_fwd |  | CAACAAGAAATATCAGTAACT |  | 21 | 138 | 55.17 | False | 0.00 | 0.00 |
|  | VFG000948(gi:21909640)_rev |  | TCCATCTTCCTTCTTGATATAAT |  | 23 | 138 | 58.73 | False | 0.00 | 0.00 |
|  | VFG000948(gi:21909640)_lnc3 | GCAGCGTGGGCTCGCGTGCCGGGGTTTTTT | AAGGAATGGCTTATATTTAT |  | 20 | 138 | 54.00 | False | 0.00 | 0.00 |
|  | VFG000948(gi:21909640)_detection |  | TCTGTAAAAGAAGTAG | TTT | 16 | 138 | 47.26 | False | 33.24 | 205.10 |
| llsB | VFG045335(gi:588561368)_fwd |  | ATTTTTGTGTTTTCTTTGGAT |  | 21 | 145 | 56.68 | False | 0.00 | 0.00 |
|  | VFG045335(gi:588561368)_rev |  | GGACAACTTGTATAGTGAACCG |  | 22 | 145 | 62.88 | False | 41.56 | -283.86 |
|  | VFG045335(gi:588561368)_lnc3 | GCAGCGTGGGCTCGCGTGCCGGGGTTTTTT | AGAACTATCTTTACTG |  | 16 | 145 | 46.92 | False | 33.56 | 157.17 |
|  | VFG045335(gi:588561368)_detection |  | AATACGTTTGTGGAA | TTT | 15 | 145 | 50.17 | False | 0.00 | 0.00 |
| spa | VFG001313(gi:21281813)_fwd |  | AGAACTTGTTGTTGATAAGAA |  | 21 | 146 | 57.04 | False | 43.44 | -433.11 |
|  | VFG001313(gi:21281813)_rev |  | CTAATAACGCTGCACCTAAC |  | 20 | 146 | 60.16 | False | 0.00 | 0.00 |
|  | VFG001313(gi:21281813)_lnc3 | GCAGCGTGGGCTCGCGTGCCGGGGTTTTTT | ATTACCAGAAACTG |  | 14 | 146 | 45.17 | False | 0.00 | 0.00 |
|  | VFG001313(gi:21281813)_detection |  | GTGAAGAAAATCCATTCA | TTT | 18 | 146 | 54.51 | False | 0.00 | 0.00 |
| icaB | VFG001287(gi:21284317)_fwd |  | ATGACGATTCACCTAAAAAACTGA |  | 24 | 156 | 63.13 | False | 38.33 | -73.51 |
|  | VFG001287(gi:21284317)_rev |  | CAAATTGTGATTGACTAACACTA |  | 23 | 156 | 59.17 | False | 38.60 | -158.68 |
|  | VFG001287(gi:21284317)_lnc3 | GCAGCGTGGGCTCGCGTGCCGGGGTTTTTT | GCGAATTTTCTGAATAA |  | 17 | 156 | 51.61 | False | 0.00 | 0.00 |
|  | VFG001287(gi:21284317)_detection |  | TTTTATTTACTTCTTTTC | TTT | 18 | 156 | 46.75 | False | 0.00 | 0.00 |
| ratB | VFG002306(gi:16765834)_fwd |  | GCAAAGCACCGGATAAT |  | 17 | 99 | 58.17 | False | 0.00 | 0.00 |
|  | VFG002306(gi:16765834)_rev |  | AGGTTGATTTAGATAAATAGGGATA |  | 25 | 99 | 59.43 | False | 40.96 | -338.75 |
|  | VFG002306(gi:16765834)_lnc3 | GCAGCGTGGGCTCGCGTGCCGGGGTTTTTT | TGTTGACGCTGTATC |  | 15 | 99 | 53.97 | False | 0.00 | 0.00 |
|  | VFG002306(gi:16765834)_detection |  | ATTCAATTAGCTCGTTA | TTT | 17 | 99 | 51.76 | False | 0.00 | 0.00 |
| erp | VFG001420(gi:15610946)_fwd |  | CAGTTCGGGATCAACAT |  | 17 | 147 | 56.96 | False | 0.00 | 0.00 |
|  | VFG001420(gi:15610946)_rev |  | ACTGGTAAGGGCAGGGTC |  | 18 | 147 | 64.39 | False | 0.00 | 0.00 |
|  | VFG001420(gi:15610946)_lnc3 | GCAGCGTGGGCTCGCGTGCCGGGGTTTTTT | GTCTAACCGGTCCT |  | 14 | 147 | 53.79 | False | 0.00 | 0.00 |
|  | VFG001420(gi:15610946)_detection |  | GGCCTGACTAGTCC | TTT | 14 | 147 | 54.89 | False | 0.00 | 0.00 |
| set1B | VFG000860(gi:387610009)_fwd |  | ACATAATTTCCTGTTCCGTCAT |  | 22 | 106 | 62.02 | False | 0.00 | 0.00 |
|  | VFG000860(gi:387610009)_rev |  | CACGTAACGCCTCGCTG |  | 17 | 106 | 64.21 | False | 0.00 | 0.00 |
|  | VFG000860(gi:387610009)_lnc3 | GCAGCGTGGGCTCGCGTGCCGGGGTTTTTT | TCCCAGTGTGATAT |  | 14 | 106 | 48.93 | False | 0.00 | 0.00 |
|  | VFG000860(gi:387610009)_detection |  | GGCTGTTATCCGAC | TTT | 14 | 106 | 53.40 | False | 0.00 | 0.00 |
| icaR | VFG001284(gi:21284314)_fwd |  | AATCAAAATAAATCGAACTATTCAA |  | 25 | 143 | 58.32 | False | 0.00 | 0.00 |
|  | VFG001284(gi:21284314)_rev |  | TGTCCGTAAATATTTCCAGA |  | 20 | 143 | 57.71 | False | 0.00 | 0.00 |
|  | VFG001284(gi:21284314)_lnc3 | GCAGCGTGGGCTCGCGTGCCGGGGTTTTTT | TTTGAGTTTATTTTCGACA |  | 19 | 143 | 54.95 | False | 0.00 | 0.00 |
|  | VFG001284(gi:21284314)_detection |  | TCGAAGAAAGGTATATTAG | TTT | 19 | 143 | 52.98 | False | 0.00 | 0.00 |
| relA | VFG001826(gi:15609720)_fwd |  | GGTTGGTAGCGCTGGAAC |  | 18 | 105 | 64.35 | False | 0.00 | 0.00 |
|  | VFG001826(gi:15609720)_rev |  | CCACGAACTGCTGCCAG |  | 17 | 105 | 64.16 | True | 46.51 | -788.56 |
|  | VFG001826(gi:15609720)_detection |  | GAGGTTTTCACGTC | TTT | 14 | 105 | 51.07 | False | 0.00 | 0.00 |
|  | VFG001826(gi:15609720)_lnc3 | GCAGCGTGGGCTCGCGTGCCGGGGTTTTTT | ACGGAGAAGTTGTC |  | 14 | 105 | 52.07 | False | 0.00 | 0.00 |
| pilS | VFG001213(gi:15599742)_fwd |  | ATCCTCCGTCTGTACCAC |  | 18 | 151 | 61.32 | False | 0.00 | 0.00 |
|  | VFG001213(gi:15599742)_rev |  | CCAGGATGTTGAAGACC |  | 17 | 151 | 57.31 | False | 0.00 | 0.00 |
|  | VFG001213(gi:15599742)_lnc3 | GCAGCGTGGGCTCGCGTGCCGGGGTTTTTT | ATCTCCAGCGAACT |  | 14 | 151 | 53.75 | False | 0.00 | 0.00 |
|  | VFG001213(gi:15599742)_detection |  | GGAAGATCAGGTCC | TTT | 14 | 151 | 51.74 | False | 0.00 | 0.00 |
| EF0149 | VFG002171(gi:29374799)_fwd |  | ACGTAAAAGTTCGCTTAA |  | 18 | 117 | 55.32 | False | 40.66 | -226.53 |
|  | VFG002171(gi:29374799)_rev |  | TTGTTCCTAGACTAGAGTTTAAT |  | 23 | 117 | 58.55 | False | 0.00 | 0.00 |
|  | VFG002171(gi:29374799)_detection |  | GCAAAGAAGTTTTACC | TTT | 16 | 117 | 51.45 | False | 0.00 | 0.00 |
|  | VFG002171(gi:29374799)_lnc3 | GCAGCGTGGGCTCGCGTGCCGGGGTTTTTT | ATTATATGATGCTAATG |  | 17 | 117 | 46.48 | False | 0.00 | 0.00 |
| icmW | VFG001852(gi:52842894)_fwd |  | GCAGGTTTTTTTCTAAAACGC |  | 21 | 111 | 61.09 | False | 44.16 | -744.40 |
|  | VFG001852(gi:52842894)_rev |  | TTCATCCCCTTCGAGTG |  | 17 | 111 | 58.74 | False | 0.00 | 0.00 |
|  | VFG001852(gi:52842894)_detection |  | GTCTAAAACTCGTTT | TTT | 15 | 111 | 48.00 | False | 0.00 | 0.00 |
|  | VFG001852(gi:52842894)_lnc3 | GCAGCGTGGGCTCGCGTGCCGGGGTTTTTT | TTTTTTGCCAATACC |  | 15 | 111 | 49.43 | False | 0.00 | 0.00 |
| cpsE | VFG001348(gi:22537329)_fwd |  | AGTTTAGTAGGAACACGCC |  | 19 | 108 | 60.55 | False | 0.00 | 0.00 |
|  | VFG001348(gi:22537329)_rev |  | TATTTGCCACAAACCAGTGAT |  | 21 | 108 | 62.26 | True | 45.90 | -649.74 |
|  | VFG001348(gi:22537329)_detection |  | CGACGCCTTAGTTTT | TTT | 15 | 108 | 54.75 | False | 0.00 | 0.00 |
|  | VFG001348(gi:22537329)_lnc3 | GCAGCGTGGGCTCGCGTGCCGGGGTTTTTT | TATAATTCAACGCAGAAG |  | 18 | 108 | 54.11 | False | 0.00 | 0.00 |
| aur | VFG001314(gi:21284287)_fwd |  | GGTGACAGTAATAAATACGT |  | 20 | 125 | 56.30 | False | 43.19 | -483.49 |
|  | VFG001314(gi:21284287)_rev |  | TCTTTAACTAAGTTCATTTTTTCT |  | 24 | 125 | 57.19 | False | 38.54 | -129.54 |
|  | VFG001314(gi:21284287)_lnc3 | GCAGCGTGGGCTCGCGTGCCGGGGTTTTTT | GTTAATTACAGTGACACC |  | 18 | 125 | 54.69 | False | 0.00 | 0.00 |
|  | VFG001314(gi:21284287)_detection |  | AGAAATTTCACATTGG | TTT | 16 | 125 | 50.02 | False | 0.00 | 0.00 |
| cpa | VFG000949(gi:21909634)_fwd |  | ATGGAGAATCATTGATTGTT |  | 20 | 150 | 56.41 | False | 40.04 | -110.54 |
|  | VFG000949(gi:21909634)_rev |  | CAGTCTCATCTTCCTTAACTGA |  | 22 | 150 | 61.14 | False | 40.15 | -296.27 |
|  | VFG000949(gi:21909634)_lnc3 | GCAGCGTGGGCTCGCGTGCCGGGGTTTTTT | TGAAATTACAGAAACGG |  | 17 | 150 | 53.61 | False | 0.00 | 0.00 |
|  | VFG000949(gi:21909634)_detection |  | GTGCTTCAGATTATG | TTT | 15 | 150 | 48.81 | False | 0.00 | 0.00 |
| lipF | VFG001415(gi:15610623)_fwd |  | GCCGCTGCCAAGAACAT |  | 17 | 98 | 64.22 | False | 0.00 | 0.00 |
|  | VFG001415(gi:15610623)_rev |  | GAAACGTGAATAAGTGTCG |  | 19 | 98 | 57.82 | False | 0.00 | 0.00 |
|  | VFG001415(gi:15610623)_detection |  | GACCATATCGAATC | TTT | 14 | 98 | 46.14 | False | 0.00 | 0.00 |
|  | VFG001415(gi:15610623)_lnc3 | GCAGCGTGGGCTCGCGTGCCGGGGTTTTTT | TCTATGAGCCGCTC |  | 14 | 98 | 54.62 | False | 0.00 | 0.00 |
| vexD | VFG000424(gi:16763114)_fwd |  | TTTTCTGGAACATCTTTCTTTGGT |  | 24 | 117 | 63.53 | False | 42.33 | -178.96 |
|  | VFG000424(gi:16763114)_rev |  | TAAAAAACCGAGCTCTTTTT |  | 20 | 117 | 57.51 | False | 39.92 | -353.24 |
|  | VFG000424(gi:16763114)_lnc3 | GCAGCGTGGGCTCGCGTGCCGGGGTTTTTT | AGTTTATACTTTCGAA |  | 16 | 117 | 47.20 | False | 0.00 | 0.00 |
|  | VFG000424(gi:16763114)_detection |  | AGAAATGATGGATCG | TTT | 15 | 117 | 50.24 | False | 0.00 | 0.00 |
| rrgA | VFG005301(gi:15900378)_fwd |  | GGAATACTATTAAGTGATTTTATTA |  | 25 | 150 | 55.14 | False | 40.60 | -234.05 |
|  | VFG005301(gi:15900378)_rev |  | ATATTTTTCAGGTTTAACTGGGAAA |  | 25 | 150 | 61.73 | False | 42.28 | -445.05 |
|  | VFG005301(gi:15900378)_detection |  | AGATGGGCAAAGTTA | TTT | 15 | 150 | 52.13 | False | 0.00 | 0.00 |
|  | VFG005301(gi:15900378)_lnc3 | GCAGCGTGGGCTCGCGTGCCGGGGTTTTTT | TACAATTGTACGCGG |  | 15 | 150 | 54.49 | False | 0.00 | 0.00 |
| cps4K | VFG001375(gi:15900288)_fwd |  | GAAAAAATCTTATCTGTACTCTGG |  | 24 | 116 | 59.62 | False | 0.00 | 0.00 |
|  | VFG001375(gi:15900288)_rev |  | TAATAAAATCGGATCTGTATCAC |  | 23 | 116 | 58.07 | False | 37.30 | -21.26 |
|  | VFG001375(gi:15900288)_detection |  | AGATTGGAGAGATTTTT | TTT | 17 | 116 | 51.38 | False | 0.00 | 0.00 |
|  | VFG001375(gi:15900288)_lnc3 | GCAGCGTGGGCTCGCGTGCCGGGGTTTTTT | ACTCGTCCTAATTTAG |  | 16 | 116 | 50.24 | False | 0.00 | 0.00 |
| pilM | VFG001222(gi:15600237)-148-166_fwd |  | TGGAAAAGAACATCGTCG |  | 18 | 133 | 58.53 | False | 0.00 | 0.00 |
|  | VFG001222(gi:15600237)-262-281_rev |  | ATCTCGATGGTCTTGGTGA |  | 19 | 133 | 61.57 | False | 0.00 | 0.00 |
|  | VFG001222(gi:15600237)-218-232_detection |  | CAACCTGAAGTCGG | TTT | 14 | 133 | 53.60 | False | 0.00 | 0.00 |
|  | VFG001222(gi:15600237)-204-218_lnc3 | GCAGCGTGGGCTCGCGTGCCGGGGTTTTTT | GTCAAGGCGAAAAC |  | 14 | 133 | 52.41 | False | 0.00 | 0.00 |
| fHbp | VFG037136(gi:15677705)_fwd |  | CCRGTTCAGAATCGGCG |  | 17 | 105 | 62.53 | False | 40.79 | -295.89 |
|  | VFG037136(gi:15677705)_rev |  | GCATCGTCTGAACYGAA |  | 17 | 105 | 58.82 | False | 41.78 | -344.39 |
|  | VFG037136(gi:15677705)_detection |  | ACAAGCTTCCCGAA | TTT | 14 | 105 | 54.92 | False | 0.00 | 0.00 |
|  | VFG037136(gi:15677705)_lnc3 | GCAGCGTGGGCTCGCGTGCCGGGGTTTTTT | GAACATACATCTTTTG |  | 16 | 105 | 47.75 | False | 0.00 | 0.00 |
| pfbA | VFG043465(gi:15903694)_fwd |  | CGACAATAAACAAGATAATAAGA |  | 23 | 176 | 56.62 | False | 0.00 | 0.00 |
|  | VFG043465(gi:15903694)_rev |  | AACCAATTCCTGTCACTGTT |  | 20 | 176 | 61.27 | False | 0.00 | 0.00 |
|  | VFG043465(gi:15903694)_detection |  | CTAAAGAAATAGTAGATGTA | TTT | 20 | 176 | 50.94 | False | 0.00 | 0.00 |
|  | VFG043465(gi:15903694)_lnc3 | GCAGCGTGGGCTCGCGTGCCGGGGTTTTTT | TGAAAATCATATTAATAAAT |  | 20 | 176 | 47.56 | False | 0.00 | 0.00 |
| ykgK/ecpR | VFG002413(gi:15799999)_fwd |  | GGAAATTATCCGCATGACG |  | 19 | 93 | 60.24 | False | 43.35 | -387.05 |
|  | VFG002413(gi:15799999)_rev |  | CGCCGATGGGTATACACT |  | 18 | 93 | 62.19 | False | 37.38 | -21.45 |
|  | VFG002413(gi:15799999)_detection |  | AATTGAAAATTGTAGT | TTT | 16 | 93 | 45.76 | False | 0.00 | 0.00 |
|  | VFG002413(gi:15799999)_lnc3 | GCAGCGTGGGCTCGCGTGCCGGGGTTTTTT | AAATCGATTGCCAG |  | 14 | 93 | 50.52 | False | 0.00 | 0.00 |
| llsA | VFG045331(gi:588561364)_fwd |  | ATATTAAATCACAATCATCAAATG |  | 24 | 110 | 56.29 | False | 0.00 | 0.00 |
|  | VFG045331(gi:588561364)_rev |  | TGTACATGTGCAAGTTGAAC |  | 20 | 110 | 60.44 | False | 0.00 | 0.00 |
|  | VFG045331(gi:588561364)_detection |  | CTATGCAGCTGGAT | TTT | 14 | 110 | 51.81 | False | 0.00 | 0.00 |
|  | VFG045331(gi:588561364)_lnc3 | GCAGCGTGGGCTCGCGTGCCGGGGTTTTTT | CTCTGAAGCAATGAA |  | 15 | 110 | 50.85 | False | 0.00 | 0.00 |
| scpA | VFG000974(gi:15675796)_fwd |  | CGAAATTGTAAATGGACTAG |  | 20 | 158 | 55.49 | False | 0.00 | 0.00 |
|  | VFG000974(gi:15675796)_rev |  | CATAGTCAAAGGCTTTTTTGGTT |  | 23 | 158 | 62.36 | False | 42.29 | -556.71 |
|  | VFG000974(gi:15675796)_lnc3 | GCAGCGTGGGCTCGCGTGCCGGGGTTTTTT | AACTTGGGAGCTAAG |  | 15 | 158 | 52.76 | False | 0.00 | 0.00 |
|  | VFG000974(gi:15675796)_detection |  | GTGATTAATATGAGCTTTG | TTT | 19 | 158 | 53.49 | False | 0.00 | 0.00 |
